# The long noncoding RNA *CARDINAL* attenuates cardiac hypertrophy by modulating protein translation

**DOI:** 10.1172/JCI169112

**Published:** 2024-05-14

**Authors:** Xin He, Tiqun Yang, Yao Wei Lu, Gengze Wu, Gang Dai, Qing Ma, Mingming Zhang, Huimin Zhou, Tianxin Long, Youchen Yan, Zhuomin Liang, Chen Liu, William T. Pu, Yugang Dong, Jingsong Ou, Hong Chen, John D. Mably, Jiangui He, Da-Zhi Wang, Zhan-Peng Huang

**Affiliations:** 1 Department of Cardiology, Center for Translational Medicine, Institute of Precision Medicine, The First Affiliated Hospital, Sun Yat-sen University, Guangzhou, China.; 2 Department of Cardiology, Boston Children’s Hospital, Harvard Medical School, Boston, Massachusetts, USA.; 3 NHC Key Laboratory of Assisted Circulation, Sun Yat-sen University, Guangzhou, China.; 4 Vascular Biology Program, Department of Surgery, Boston Children’s Hospital, Harvard Medical School, Boston, Massachusetts, USA.; 5 Division of Cardiac Surgery, National-Guangdong Joint Engineering Laboratory for Diagnosis and Treatment of Vascular Diseases, Key Laboratory of Assisted Circulation and Vascular Diseases, Chinese Academy of Medical Sciences, The First Affiliated Hospital, Sun Yat-sen University, Guangzhou, China.; 6 Center for Regenerative Medicine, USF Health Heart Institute and; 7 Departments of Internal Medicine, Molecular Pharmacology and Physiology, Morsani College of Medicine, University of South Florida, Tampa, Florida, USA.

**Keywords:** Cardiology, Development, Cardiovascular disease, Mouse models, Translation

## Abstract

One of the features of pathological cardiac hypertrophy is enhanced translation and protein synthesis. Translational inhibition has been shown to be an effective means of treating cardiac hypertrophy, although system-wide side effects are common. Regulators of translation, such as cardiac-specific long noncoding RNAs (lncRNAs), could provide new, more targeted therapeutic approaches to inhibit cardiac hypertrophy. Therefore, we generated mice lacking a previously identified lncRNA named *CARDINAL* to examine its cardiac function. We demonstrate that *CARDINAL* is a cardiac-specific, ribosome-associated lncRNA and show that its expression was induced in the heart upon pathological cardiac hypertrophy and that its deletion in mice exacerbated stress-induced cardiac hypertrophy and augmented protein translation. In contrast, overexpression of *CARDINAL* attenuated cardiac hypertrophy in vivo and in vitro and suppressed hypertrophy-induced protein translation. Mechanistically, *CARDINAL* interacted with developmentally regulated GTP-binding protein 1 (DRG1) and blocked its interaction with DRG family regulatory protein 1 (DFRP1); as a result, DRG1 was downregulated, thereby modulating the rate of protein translation in the heart in response to stress. This study provides evidence for the therapeutic potential of targeting cardiac-specific lncRNAs to suppress disease-induced translational changes and to treat cardiac hypertrophy and heart failure.

## Abstract 2

The cardiac-specific long noncoding RNA *CARDINAL* suppresses protein translation under stress conditions and provides a treatment target for cardiac hypertrophy and heart failure.

## Introduction

Heart failure is the common end stage for most cardiomyopathies and has both a high prevalence and mortality rate (1, 2). Although previous decades have witnessed great progress in short-term heart failure treatment, its long-term prognosis remains poor (2). Pathological cardiac hypertrophy is one of the driving forces in heart failure. Numerous studies have demonstrated that inhibition of cardiac hypertrophy under stress conditions contributes to the preservation of cardiac function (3).

Translation is enhanced during the development of cardiac hypertrophy (4); however, we have a limited understanding of the mechanisms regulating translation and few approaches for therapeutic intervention. In fact, most published mechanistic studies of cardiac hypertrophy have focused on transcriptional regulation (5). Unfortunately, these studies have limited value for the development of treatments for hypertrophy, since mRNA levels only moderately correlate with protein amounts (6). Our previous studies highlighted the importance of translational regulation in cardiac hypertrophy and the importance of its continued interrogation as a mechanism and potential target for clinical intervention (7, 8).

The imbalance of protein synthesis and degradation is the underlying cause for the increased heart weight reported in cardiac hypertrophy. Studies have confirmed that translational inhibition achieved by modulating regulatory protein activity is efficient in suppressing cardiac hypertrophy, whereas translation promotion exacerbates cardiac hypertrophy (9–11). However, most of these regulatory proteins were constitutively expressed, with functions in multiple tissues; the targeting of these molecules inevitably led to system-wide side effects. For example, mTOR is an important protein kinase that regulates the rate of translation (12). Preclinical and clinical data showed that mTOR inhibitors are effective in reversing cardiac hypertrophy (13–16). However, these therapies have potential to cause serious side effects, such as immunosuppression and thrombocytopenia, which is not an acceptable risk in treatments for heart failure (17). In principle, the characterization of cardiac-specific translational regulators would identify new, safer therapeutic targets for cardiac hypertrophy and heart failure. The underlying concepts that prompted our investigation of long noncoding RNA (lncRNA) molecules as potential cardiac-specific translational regulators included published reports demonstrating that (a) lncRNAs typically demonstrate a higher tissue specificity compared with protein coding genes (18), and (b) many lncRNAs without coding potential have been found to be associated with the ribosome, which indicates a potential role in regulating ribosomal function (19–21).

In this study, we investigate the function of a cardiac-specific, translation-altering lncRNA that was previously identified as a serum response factor–interacting lncRNA and named myocardin-adjacent lncRNA, abbreviated as *CARDINAL* (22). Here, we report that *CARDINAL* was associated with the ribosome to suppress translation in cardiomyocytes under stress conditions. Loss of *CARDINAL* exacerbated cardiac hypertrophy in response to stress. Mechanistically, we show that *CARDINAL* interacted with developmentally regulated GTP-binding protein 1 (DRG1), an enhancer of translation. We also demonstrate that *CARDINAL* promoted the degradation of DRG1 by preventing its interaction with the DRG1-stabilizing partner DRG family regulatory protein 1 (DFRP1).

## Results

### Screening and identification of CARDINAL as a cardiac-specific ribosome–associated lncRNA.

**Figure 1:**
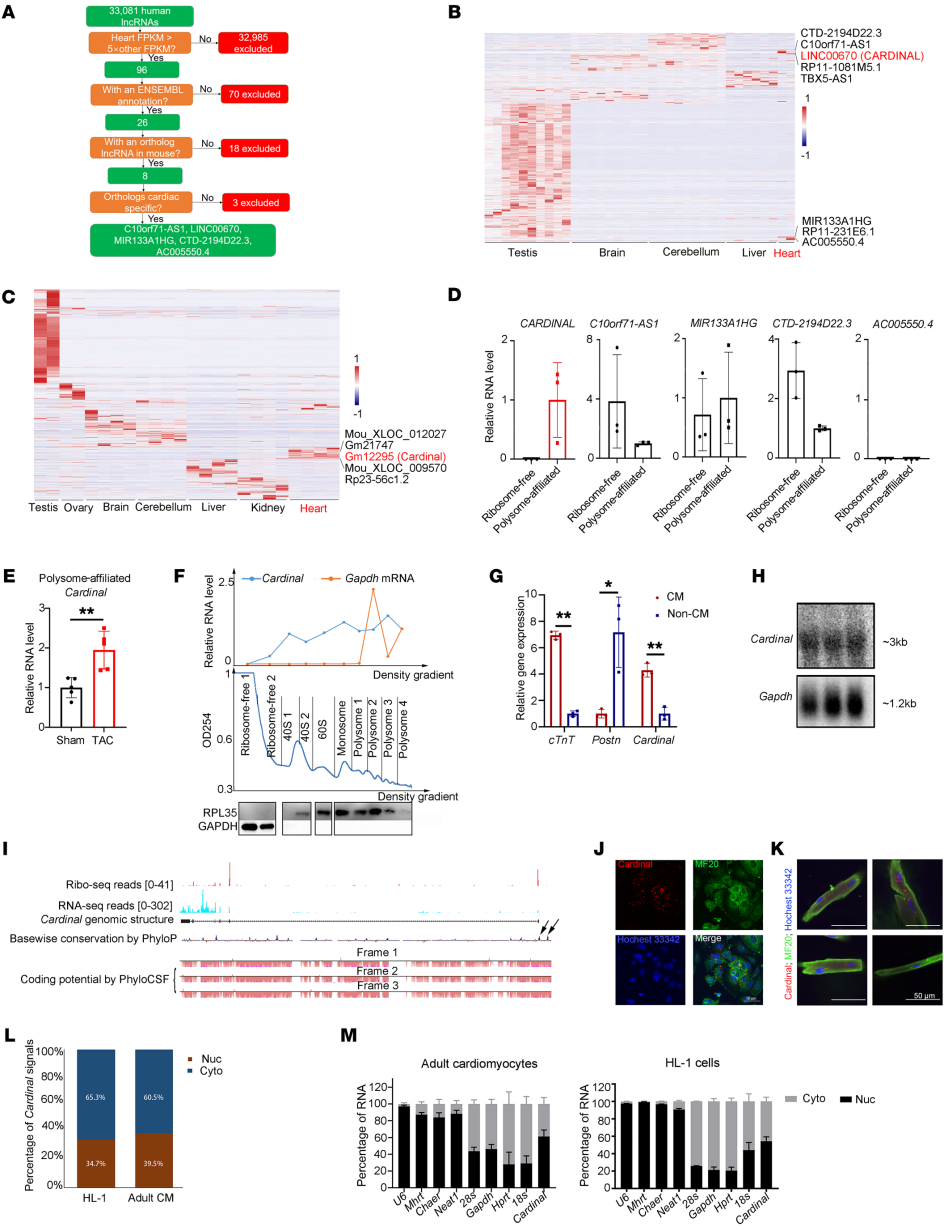
Identification of *CARDINAL* by screening for cardiac-specific, ribosome-associated lncRNAs. (**A**) Flow chart of screening for cardiac-specific lncRNAs in a human multiorgan RNA-Seq database (https://apps.kaessmannlab.org/lncRNA_app). (**B**) Heatmap showing the cardiac specificity of candidate human lncRNAs identified in **A**. (**C**) Heatmap showing the cardiac specificity of mouse orthologs of candidate lncRNAs. (**D**) Relative expression level of 5 lncRNA candidates detected by RNA-Seq in ribosome-free fraction and a polysome fraction following polysome profiling in hESC-CMs (SRP150416) (*n* = 3 for each group). (**E**) Relative expression levels of *Cardinal* in polysome fractions of mouse hearts after sham or TAC surgery (GSE131296) (*n* = 5 for each group). (**F**) Polysome profiling of HL-1 cells and results of RT-qPCR and Western blotting with different fractions. (**G**) Relative expression levels of *Cardinal* in different cell types in hearts, detected by RT-qPCR (*n* = 3 for each group). (**H**) Northern blotting of endogenous *Cardinal* from adult mouse hearts. *Gapdh* serves as a control for loading. (**I**) Genomic structure of *Cardinal* with Ribo-Seq and RNA-Seq read coverage, basewise conservation calculated by PhyloP, and coding potential calculated by PhyloCSF. Black arrows indicate 2 conserved promoter regions. Tracks of Ribo-Seq and RNA-Seq read coverage were obtained from the Hubner Laboratory (http://shiny.mdc-berlin.de/cardiac-translatome/). Tracks of basewise conservation and coding potential were obtained from the UCSC genome browser (https://genome.ucsc.edu/). (**J**) Single-molecule RNA-FISH of *Cardinal* in HL-1 cells. (**K**) Single-molecule RNA-FISH in cardiomyocytes from adult mice. (**L**) Quantification of *Cardinal* RNA-FISH signals in the nucleus (Nuc) and cytoplasm (Cyto) in at least 100 randomly selected adult cardiomyocytes (CM) and HL-1 cells. (**M**) Relative amount of *Cardinal* in the nucleus versus the cytoplasm detected by RT-qPCR following nucleus/cytoplasm fractionation in adult cardiomyocytes and HL-1 cells (*n* = 3 for each group). *P < 0.05 and **P < 0.01, by 2-tailed Student’s *t* test (**E** and **G**). Scale bars: 50 μm (**J** and **K**). Cyto, cytoplasm; Nuc, nucleus.

The objective of this analysis was to identify cardiac-specific, ribosome-associated lncRNAs in the human genome. To identify cardiac-specific lncRNAs, our initial analysis was performed using large RNA-Seq data sets generated from 7 major organs, in 7 species, across multiple developmental time points (23). Since the onset of cardiac hypertrophy and heart failure is more common in the older population, we focused on adult human samples; these included 2 heart samples, 9 brain samples, 9 cerebellum samples, 6 liver samples, and 6 testis samples (there were no kidney or ovary samples from human adults). To ensure cardiac specificity, we focused on lncRNAs in hearts with a fragments per kilobase per million mapped reads (FPKM) value 5 times greater than that in any of the other samples; this initial screening yielded 96 candidate lncRNAs (Figure 1A). Among these lncRNAs, we excluded 70 that did not have an Ensembl annotation and 18 that did not have an ortholog in the mouse (Figure 1, A and B). The remaining 8 lncRNAs were cross-checked for cardiac specificity using adult mice tissues to ensure their conservation in expression between humans and mice. Three lncRNAs were further excluded because of the low cardiac specificity of the mouse orthologs (Figure 1, A and C, and Supplemental Figure 1; supplemental material available online with this article; https://doi.org/10.1172/JCI169112DS1).

We next examined the association of the 5 candidate lncRNAs with the ribosome. Lysates of human embryonic stem cell–derived cardiomyocytes (hESC-CMs) were subjected to polysome profiling to separate ribosome-free fractions and polysome fractions. RNA isolated from these fractions was subjected to RNA-Seq (24, 25). We detected 4 of the 5 candidates in the polysome fractions; among them, *CARDINAL* (LINC00670) exhibited the strongest association (Figure 1D); the ortholog of *CARDINAL* in mouse, Gm12295, was found to be associated with the ribosome and detected in the polysome fraction of heart (Figure 1E). Interestingly, polysome-associated *CARDINAL* increased during cardiac hypertrophy induced by transverse aortic constriction (TAC) (Figure 1E). To verify the association of *CARDINAL* with the ribosome, we performed polysome profiling followed by real-time quantitative PCR (RT-qPCR). We detected *CARDINAL* expression in the 40S, 60S, monosome, and polysome fraction, but not in the ribosome-free fraction (Figure 1F). This distribution pattern was distinct from that of the translated transcript for *Gapdh* mRNA, suggesting that the function of *CARDINAL* is linked to its association with the ribosome (Figure 1F). Together, these data demonstrate that *CARDINAL* is a ribosome-associated lncRNA.

*CARDINAL* was previously described as a cardiac-enriched lncRNA that interacts with SRF to regulate cardiac gene expression (22). We validated cardiac-specific expression of *Cardinal* in mice by RT-qPCR (Supplemental Figure 2). Furthermore, we found that the expression of *Cardinal* in the heart gradually increased from embryonic and postnatal stages to adulthood, with the highest expression detected in 6-month-old mouse hearts (Supplemental Figure 2), indicating a role in adult hearts. We separated cardiomyocyte and noncardiomyocyte fractions from adult mouse hearts using the Langendorff procedure and found that *Cardinal* was predominantly expressed in cardiomyocytes. As expected, the control markers cardiac troponin T (*cTnT*) and periostin (*Postn*) were expressed in cardiomyocyte and fibroblast fractions, respectively (Figure 1G). We confirmed that the full-length *Cardinal* transcript was approximately 3 kb in length using rapid amplification of cDNA ends (RACEs) and Northern blotting approaches (Figure 1H), as previously reported (22). Ribosome-sequencing (Ribo-Seq) data indicated that the first 2 exons were associated with the ribosome (26), but PhyloCSF scoring indicated no coding potential in these regions (Figure 1I); these results suggested that *Cardinal* was a noncoding RNA. This conclusion was supported by a previous large-scale cardiac translatomics study that did not annotate *CARDINAL* as a translated RNA in either mouse or human (26). Moreover, a previous report also described *CARDINAL* as a noncoding RNA expressed in the heart (22). Both mouse and human *CARDINAL* genes have 2 highly conserved regions near the transcriptional start site (Figure 1I), indicating that the transcription of *CARDINAL* is controlled by similar regulatory networks in both species.

The subcellular location of *Cardinal* was evaluated by single-molecule RNA FISH. *Cardinal* was detected in the nuclear and cytoplasmic compartments of both HL-1 cells and isolated adult mouse cardiomyocytes (Figure 1, J and K). As a control, we confirmed that expression of Neat1, a previously reported nuclear lncRNA (27), was restricted to the nucleus (Supplemental Figure 3). Quantification showed that more than 60% of the *Cardinal* signal was in the cytoplasm in both HL-1 cells and adult cardiomyocytes (Figure 1L). To confirm the above observations, we isolated cytosolic and nuclear factions from HL-1 cells and adult cardiomyocytes and detected *Cardinal* transcripts in both (Figure 1M). Cytosolic (*28s*, *Gapdh*, *Hprt*) and nuclear (*U6*, *Mhrt*, *Chaer*, *Neat1*) transcripts were detected in their expected fractions (Figure 1M).

### CARDINAL alters the translation rate in cardiomyocytes.

**Figure 2:**
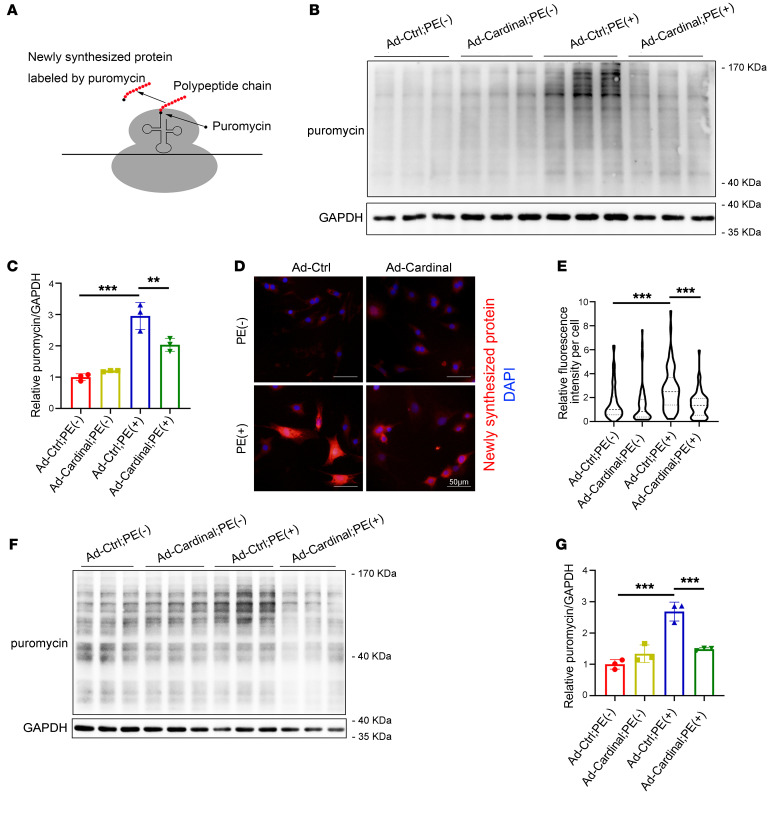
*CARDINAL* modulation alters translation. (**A**) Rationale of the SUnSET measurement. (**B**) Western blot and (**C**) quantification of puromycin-incorporated protein in NRVCs infected with control virus or Ad-*Cardinal* and treated by culture medium with or without PE (50 μM) for 24 hours. Cells were treated with 1 μM puromycin for 30 minutes before harvesting (*n* = 3 for each group). (**D**) Immunofluorescence images (scale bars: 50 μm) and (**E**) fluorescence intensity quantification of NRVCs infected with control virus or Ad-*Cardinal* and treated in culture medium with or without PE (50 μM) for 24 hours by FUNCAT assay. Newly synthesized protein was labeled by Alexa Fluor 594. Violin plots were generated to show the median, 25th and 75th percentiles. At least 100 cells were measured for quantification in each group. (**F**) Western blot and (**G**) quantification of puromycin-incorporated protein in adult cardiomyocytes infected with control virus or Ad-*Cardinal* and treated in culture medium with or without PE (50 μM) for 24 hours. Cells were treated with 1 μM puromycin for 30 minutes before harvesting (*n* = 3 for each group). **P < 0.01 and ***P < 0.001, by 2-way ANOVA with Tukey’s post hoc test (**C**, **E**, and **G**).

We assessed the effect of *CARDINAL* on protein translation in cardiomyocytes. Global translation was assessed by surface sensing of translation (SUnSET) (28). Before harvesting, cells were incubated in medium containing puromycin, which was incorporated into the nascent polypeptide chain. Western blotting of the puromycin-incorporated protein reflected the amount of newly synthesized protein within a period of time (i.e., the translation rate, Figure 2A). In isolated neonatal rat ventricular cardiomyocytes (NRVCs), overexpression of *Cardinal* did not alter the translation rate from the baseline under normal conditions (Figure 2, B and C). We then tested the effect of *Cardinal* under stress conditions using phenylephrine (PE), which is a hypertrophic agonist that also promotes the translation rate in cardiomyocytes (4). As expected, we observed an increase in the translation rate in NRVCs 24 hours after stimulation with PE; this PE-induced enhancement was suppressed by adenovirus-mediated overexpression of *Cardinal* (Figure 2, B and C). This observation was further supported using the FlUorescent Non-Canonical Amino acid Tagging (FUNCAT) assay (Figure 2, D and E). Since *Cardinal* is highly expressed in adult hearts, we also attempted to evaluate its effect on translation in isolated adult cardiomyocytes. As observed with the NRVCs, *Cardinal* was able to suppress the PE-induced translational increase (Figure 2, F and G). These data support a role for *CARDINAL* as a potent suppressor of translation in cardiomyocytes.

### Ribosome-bound CARDINAL is increased in cardiac hypertrophy.

**Figure 3:**
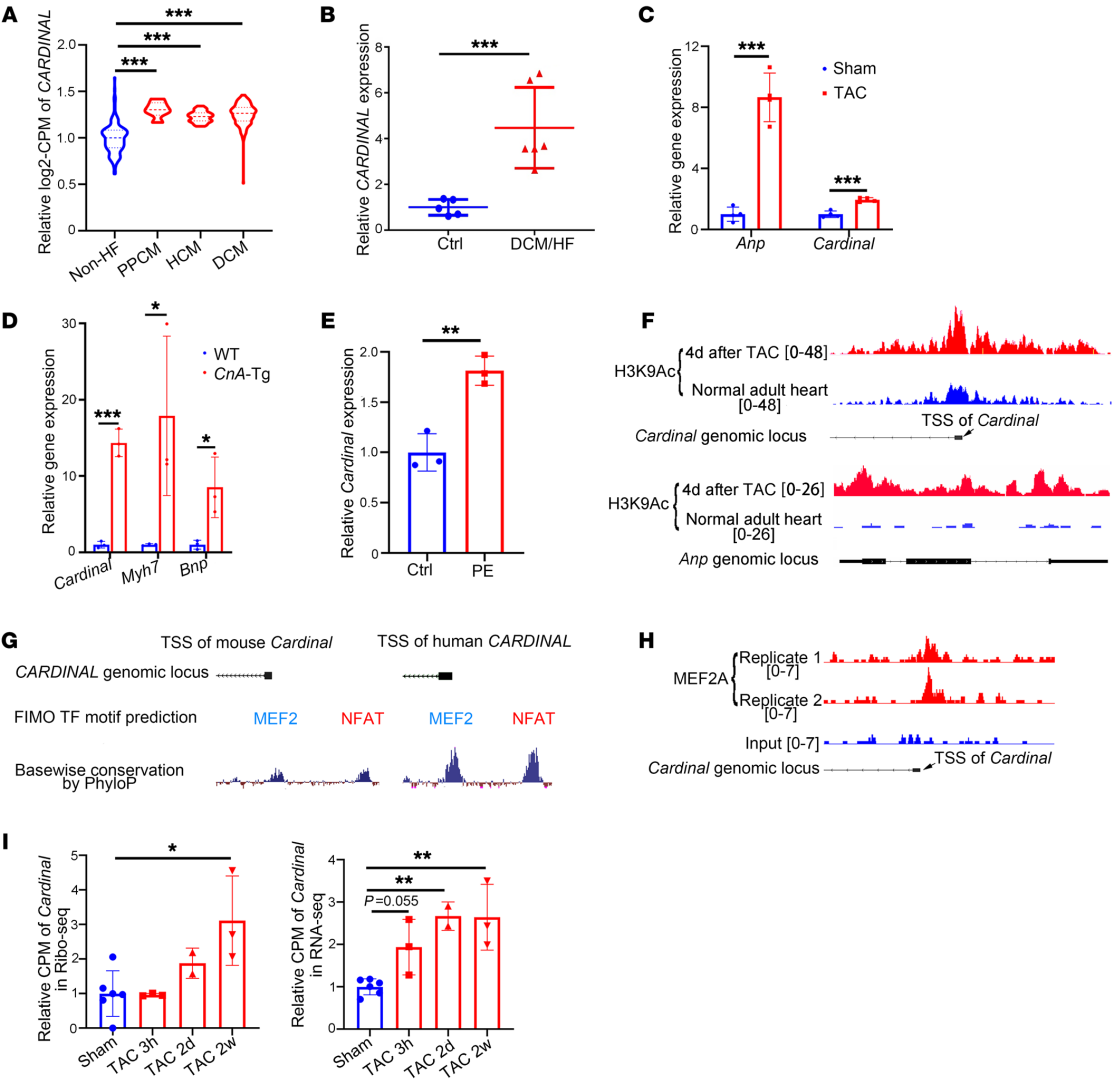
Cardiac hypertrophy upregulates *CARDINAL* and enhances its association with the ribosome. (**A**) RNS-Seq was performed to detect relative expression levels of *CARDINAL* in human heart samples from individuals without heart failure (Non-HF), with peripartum cardiomyopathy (PPCM), hypertrophic cardiomyopathy (HCM), or DCM (GSE141910). Replicate numbers of non-HF, PPCM, HCM, and DCM samples were 166, 6, 28, and 166, respectively. (**B**) Relative expression of *CARDINAL* detected by RT-qPCR in human heart samples from individuals with or without heart failure (HF)/DCM (*n* ≥5 for each group). (**C**) Relative *Anp* and *Cardinal* expression levels detected by RT-qPCR in hearts 2 weeks after sham or TAC surgery (*n* = 4 for each group). (**D**) Relative expression levels of *Cardinal* and the hypertrophic markers *Bnp* and *Myh7* in hearts from WT or *CnA*-Tg mice detected by RT-qPCR (*n* = 3 for each group). (**E**) Relative *Cardinal* expression levels detected by RT-qPCR in isolated adult mouse cardiomyocytes treated in culture medium with or without PE (50 μM) for 24 hours. (*n* = 3 for each group). (**F**) Read coverage of histone H3K9Ac CHIP-Seq near the transcription start sites (TSSs) of *Cardinal* and *Anp* from normal hearts or hearts 4 days after TAC surgery (GSE50637). (**G**) MEF2 and NFAT were predicted to bind the conserved promoter regions of *CARDINAL* in both humans and mice by the software tool Find Individual Motif Occurrences (FIMO). (**H**) Read coverage of MEF2A CHIP-Seq near the TSS of *Cardinal* (GEO GSE124008). (**I**) Ribosome-associated and total *Cardinal* levels detected by Ribo-Seq and RNA-Seq from hearts after sham surgery or 3 hours, 2 days, or 2 weeks after TAC surgery (PRJNA484227). The replicate numbers of sham surgery, 3 hours after TAC (TAC 3h), 2 days after TAC (TAC 2d), and 2 weeks after TAC (TAC 2w) were 6, 3, 2, and 3, respectively. *P < 0.05, **P < 0.01, and ***P < 0.001, by 2-tailed Student’s *t* test (**B**–**E**) or 2-way ANOVA with Tukey’s post hoc test (**A** and **I**).

Since increased translation is a major observation during cardiac hypertrophy, we asked whether the level of *CARDINAL* was altered during this process. We first analyzed transcriptomics data from more than 300 human heart samples and found that the level of *CARDINAL* was increased in failing hearts regardless of the heart failure etiology (Figure 3A). We validated these results by RT-qPCR using human diseased heart samples (Figure 3B). Similarly, we found that *Cardinal* expression was increased in a mouse model of pressure overload–induced cardiac hypertrophy (Figure 3C). *Cardinal* expression was also dramatically increased in hearts isolated from calcineurin A–transgenic (*CnA*-Tg) mice (Figure 3D). The cardiac hypertrophy induced by *CnA* overexpression is also associated with elevation of the hypertrophic markers *Bnp* and *Myh7* (29). As further support of this finding, we observed increased *Cardinal* expression in isolated adult mouse cardiomyocytes treated with PE to induce hypertrophy (Figure 3E).

To better understand how transcription of the *CARDINAL* gene is induced during cardiac hypertrophy, we examined the promoter regions of the *CARDINAL* gene. Pressure overload– induced cardiac hypertrophy was associated with increased H3K9 acetylation at the promoter regions of *Cardinal* in mouse hearts, indicating active transcription of *Cardinal* during cardiac hypertrophy (Figure 3F). As a positive control, we observed that the *Anp* genomic locus was also activated (Figure 3F). The sequences of these promoters were analyzed by the Find Individual Motif Occurrences tool to identify potential transcription factor binding sites at these regions (30). Analyses of the promoter sequences from both mouse and human genomes revealed multiple overlapping and conserved transcription factor binding sites, which included those for myocyte enhancer factor 2 (MEF2) and nuclear factor of activated T cells (NFAT) (Figure 3G), two important transcription factors that mediate transcriptomic changes in the heart under normal conditions and during cardiac hypertrophy (5). The binding of MEF2 to the promoter sequence was validated by MEF2A CHIP-Seq (Figure 3H). The results were consistent with the regulatory role of MEF2 on *CARDINAL* expression as previously reported (22). *CnA*-Tg mice (transgenic line in which the Myh6 promoter drives expression of a constitutively active calcineurin A [Ppp3ca] cDNA in cardiomyocytes) have sustained activated NFAT activity in the heart (29); coupled with the dramatic increase of *Cardinal* expression in *CnA*-Tg hearts (Figure 3D), this further supports a role for NFAT-activated *CARDINAL* transcription.

We next asked whether the increase in *CARDINAL* expression correlated with an increased association with the ribosome during heart failure. We used cardiomyocyte-specific (Ribo-Seq) data to identify transcript fragments protected by ribosomes (31). Our analysis revealed that ribosome-protected *Cardinal* began to increase 2 days after TAC surgery and peaked at 2 weeks (Figure 3I). In contrast, the overall *Cardinal* level (as revealed by RNA-Seq) began to increase as early as 3 hours after TAC and peaked at 2 days (Figure 3I). Collectively, these data demonstrated that transcript levels of *CARDINAL* and its association with ribosomes are dynamically regulated during cardiac hypertrophy.

### Loss of CARDINAL aggravates pressure overload–induced cardiac hypertrophy.

**Figure 4:**
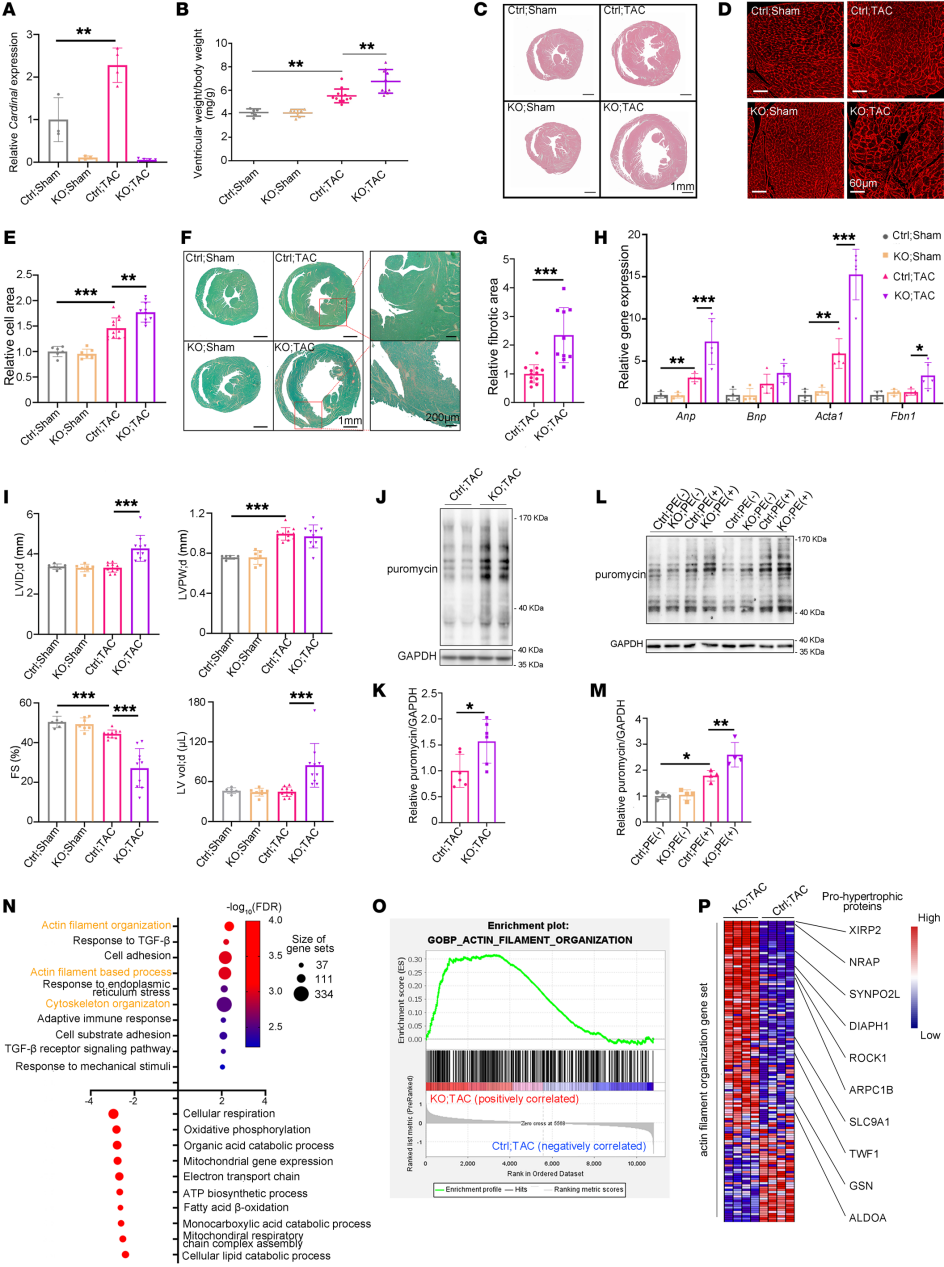
Pressure overload increases cardiac hypertrophy and enhances protein translation in *Cardinal*-KO mice. (**A**) Relative *Cardinal* expression levels detected by RT-qPCR (*n* ≥3 for each group) and (**B**) ventricular weight/body weight ratio (*n* ≥6 for each group). (**C**) H&E staining, (**D**) wheat germ agglutinin (WGA) staining (scale bars: 60 μm), (**E**) relative cardiomyocyte area quantification (*n* ≥6 for each group), (**F**) Picrosirius red/Fast Green staining (scale bars: 1 mm and 200 μm), (**G**) relative fibrosis area quantification (*n* ≥10 for each group) performed on cross sections, (**H**) relative expression levels of hypertrophy and fibrosis markers detected by RT-qPCR (*n* ≥4 for each group), and (**I**) echocardiographic parameters (*n* ≥6 for each group) of hearts from control and *Cardinal*-KO mice 4 weeks after sham or TAC surgery. (**J**) Western blot analysis and (**K**) quantification of puromycin-incorporated protein in hearts from control and *Cardinal*-KO mice 2 weeks after TAC surgery (*n* = 6 for each group). Mice were peritoneally injected with 25 mg/kg puromycin 45 minutes before sacrifice. (**L**) Western blot and (**M**) quantification of puromycin-incorporated protein in adult mouse cardiomyocytes from control or *Cardinal*-KO mice treated in culture medium with or without PE (50 μM) for 24 hours. Cells were treated with 1 μM puromycin for 30 minutes before harvesting (*n* = 4 for each group). (**N**) Summary of the GSEA results. Proteomic changes in hearts from KO TAC versus Ctrl TAC by GSEA using the gene sets from the Gene Ontology Biological Process. (**O**) Enrichment plot of the gene set “actin filament organization” generated by GSEA with translatomic alterations in hearts from KO TAC versus Ctrl TAC mice. (**P**) Heatmap showing proteomic changes in the “actin filament organization” gene set in hearts from KO TAC versus Ctrl TAC mice. Documented prohypertrophic factors among upregulated proteins are highlighted. *P < 0.05, **P < 0.01, and ***P < 0.001, by 2-tailed Student’s *t* test (**G** and **K**) or 2-way ANOVA with Tukey’s post hoc test (**A**, **B**, **E**, **H**, **I**, and **M**).

To define the function of *CARDINAL* on translation and cardiac hypertrophy in vivo, we generated *Cardinal*-KO mice (Supplemental Figures 4–6). *Cardinal* KO completely abolished *Cardinal* expression; however, the expression of myocardin (*Myocd*), located at a nearby locus, was not affected in the hearts of *Cardinal*-KO mice (Supplemental Figure 7A). We observed no overt phenotype in young adult *Cardinal*-KO mice under normal physiological conditions (Supplemental Figure 7, B and C, and Supplemental Table 1). Next, we performed TAC surgery in control and *Cardinal*-KO mice. Similar to previous results, we found that expression of *Cardinal* increased in control TAC (Ctrl TAC) compared with control sham (Ctrl sham) hearts (Figure 4A). This procedure induced more cardiac hypertrophy in *Cardinal*-KO mice compared with that observed in TAC-treated control animals (Figure 4, B–I). Compared with Ctrl TAC, *Cardinal*-KO TAC also had further increased the ventricular weight/body weight ratio (Figure 4B), heart size (Figure 4C), and cardiomyocyte cross-sectional area (Figure 4, D and E). We also observed an increase in cardiac fibrosis in *Cardinal*-KO TAC hearts (Figure 4, F and G). Furthermore, the expression levels of the hypertrophic markers *Anp*, *Bnp*, and *Acta1*, and the fibrosis marker *Fbn1* were all further increased in *Cardinal*-KO TAC hearts (Figure 4H). Echocardiographic measurement showed that control mice developed cardiac hypertrophy under TAC conditions; however, we observed further decompensated remodeling and worsened cardiac function in *Cardinal*-KO TAC hearts (Figure 4I and Supplemental Table 2). To further demonstrate the regulatory function of *CARDINAL* in cardiac hypertrophy in vivo, titrated ectopic expression of *Cardinal* in KO hearts was achieved by adeno-associated virus (AAV) to a level comparable to that in control hearts (Supplemental Figure 8, A and B). This ectopic *Cardinal* expression was able to rescue the severe cardiac hypertrophy phenotype in *Cardinal*-KO TAC hearts (Supplemental Figure 8, C–K).

To determine the molecular pathways in the heart affected by *CARDINAL* in response to stress, we performed RNA-Seq with heart samples from both control and *Cardinal*-KO mice that underwent the sham or TAC procedure. Pressure overload induced dramatic transcriptomic changes in the heart, while *Cardinal* KO further amplified these changes (Supplemental Figure 9, A and B, and Supplemental Table 3). Gene set enrichment analysis (GSEA) of *Cardinal*-KO TAC versus Ctrl TAC found that gene pathways associated with fibrosis and inflammation were upregulated, whereas pathways associated with fatty acid and amino acid metabolism and energy production were downregulated (Supplemental Figure 9C). These data support the hypothesis that *CARDINAL* participates in the regulation of cardiac hypertrophy in response to stress.

We wanted to confirm that *CARDINAL* affects protein translation in hypertrophic hearts in response to stress; we had already observed an increased rate of protein translation in *Cardinal*-KO hearts compared with controls 2 weeks after TAC surgery (Figure 4, J and K). Therefore, we isolated adult cardiomyocytes from control and *Cardinal*-KO hearts and then treated them with PE to induce cardiomyocyte hypertrophy. PE exposure boosted protein translation in these cells (Figure 4, L and M). Upon PE stimulation, cardiomyocytes from *Cardinal*-KO mice had an even higher protein translation rate compared with those from controls (Figure 4, L and M). Together, these data demonstrate that loss of *CARDINAL* promoted protein translation and cardiomyocyte hypertrophy in response to stresses.

Next, we wanted to explore how the translation enhancement in KO TAC affected the proteomics in hearts. We performed quantitative mass spectrometry using heart tissues from KO TAC and Ctrl TAC mice. GSEA analysis showed that protein levels related to actin and cytoskeleton organization, fibrosis, endoplasmic stress, and inflammation were upregulated, with the term “actin filament organization” on the top. Proteins related to energy production and metabolism were downregulated (Figure 4N). In contrast, “actin filament organization” was not among the top terms in the list of upregulated genes from the original GSEA analysis of transcriptomic changes in KO TAC hearts (Supplemental Figure 9C). The differences in these 2 GSEA analyses suggest that the upregulation of “actin filament organization” proteins was probably caused by the increase in translation. In parallel, we performed Ribo-Seq using hearts from KO TAC versus Ctrl TAC mice. Consistently, the GSEA analysis of Ribo-Seq data revealed an upregulation of “actin filament organization” genes (Figure 4O), suggesting that *Cardinal* KO promoted the translation of these genes in cardiomyocytes. Consistent with a previous report, we found upregulation of “actin filament organization” proteins to be closely linked to the promotion of cytoskeleton remodeling in cardiomyocytes, which is one of the core mechanisms for inducing cardiac hypertrophy (32). As expected, our analysis further showed that multiple upregulated proteins in the “actin filament organization” gene set have been documented as prohypertrophic factors (Figure 4P) (33–44).

### CARDINAL overexpression attenuates cardiac hypertrophy.

**Figure 5:**
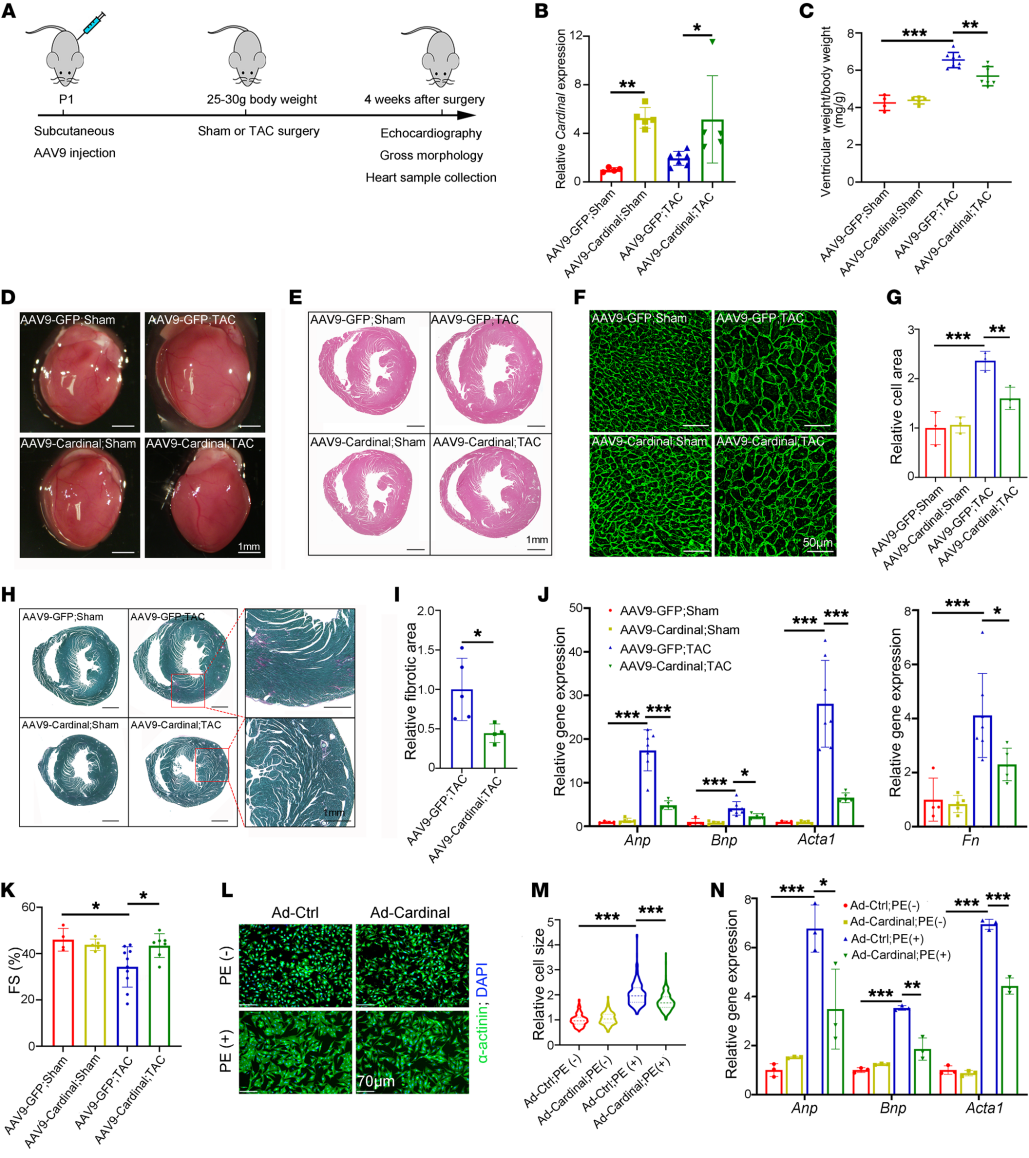
*CARDINAL* overexpression attenuates cardiomyocyte hypertrophy. (**A**) Timeline for in vivo Ca*rdinal* gain-of-function analysis. (**B**) Relative expression of *Cardinal* (*n* ≥4 for each group) detected by RT-qPCR. (**C**) Ventricular weight/body weight ratio (*n* ≥4 for each group), (**D**) gross morphology (scale bars: 1 mm), (**E**) H&E staining (scale bars: 1 mm), (**F**) WGA staining (scale bars: 50 μm), (**G**) cardiomyocyte size quantification (*n* = 3 for each group), (**H**) Picrosirius red/Fast Green staining (scale bars: 1 mm), and (**I**) fibrosis area quantification (*n* ≥4 for each group) using cross sections, (**J**) relative expression of cardiac hypertrophy and fibrosis markers (*n* ≥4 for each group), and (**K**) percentage of fractional shortening (FS) of hearts from mice injected with AAV9-Ctrl or AAV9-*Cardinal* 4 weeks after sham or TAC surgery. (**L**) Immunofluorescence images (scale bars: 70 μm) and (**M**) cell area quantification of NRVCs infected with control virus or Ad-*Cardinal* treated using culture medium with or without PE (50 μM) for 48 hours. Violin plots were generated to show the median and 25th and 75th percentiles. At least 300 cells were measured for quantification in each group. (**N**) RT-qPCR results showing relative gene expression levels of hypertrophy markers in NRVCs infected with control virus or Ad-*Cardinal* treated in culture medium with or without PE (50 μM) for 24 hours (*n* = 3 for each group). *P < 0.05, **P < 0.01, and ***P < 0.001, by 2-tailed Student’s *t* test (**I**) or 2-way ANOVA with Tukey’s post hoc test (**B**, **C**, **G**, **J**, **K**, **M**, and **N**).

Since loss of *CARDINAL* in the heart led to an increase in cardiac hypertrophy under stress conditions, we next sought to determine whether overexpression of *CARDINAL* could suppress cardiac hypertrophy. We cloned the full-length mouse *Cardinal* sequence into a vector containing AAV, serotype 9 (AAV9) with a cardiomyocyte-specific cTNT promoter, to generate AAV9-cTNT-*Cardinal* virus. Mice injected with AAV9-cTNT-*Cardinal* virus (either AAV9-cTNT-*GFP* or AAV9-cTNT-*Cardinal*-antisense virus used as a control) were subjected to TAC or sham surgery (Figure 5A and Supplemental Figure 10). We performed RT-qPCR to confirm the sustained overexpression of *Cardinal* throughout adulthood (Figure 5B). Cardiac-specific *Cardinal* overexpression did not result in an overt phenotype under normal physiological conditions. However, while the control mice developed pathological cardiac hypertrophy 4 weeks after TAC surgery, cardiomyocyte-specific overexpression of *Cardinal* suppressed these changes (Figure 5, C–K, and Supplemental Figure 10). Compared with the AAV9-*GFP* TAC group, *Cardinal*-overexpressing mice had a decrease in the ventricular weight/body weight ratio (Figure 5C), a smaller heart size (Figure 5, D and E), a reduced cardiomyocyte cross-sectional area (Figure 5, F and G), a decreased fibrotic area (Figure 5, H and I), lower expression levels of the hypertrophic markers *Anp*, *Bnp*, *Acta1*, and the fibrosis marker fibronectin (*Fn*) (Figure 5J), and improved cardiac function (Figure 5K and Supplemental Table 4). These data demonstrate the potential of *CARDINAL* overexpression in treating pressure overload–induced cardiac hypertrophy.

To further evaluate the effect of *CARDINAL* on cardiomyocyte hypertrophy, we used an in vitro cardiomyocyte hypertrophy model. PE stimulation led to increased cardiomyocyte size in NRVCs, which was suppressed upon Ad-*Cardinal* treatment (Figure 5, L and M). PE-induced expression of the hypertrophic markers *Anp*, *Bnp*, and *Acta1* was also repressed by *Cardinal* (Figure 5N). Together, the in vivo and in vitro data indicate the potential of *CARDINAL* overexpression for the attenuation of cardiomyocyte hypertrophy.

### CARDINAL interacts with the translational regulator DRG1.

**Figure 6:**
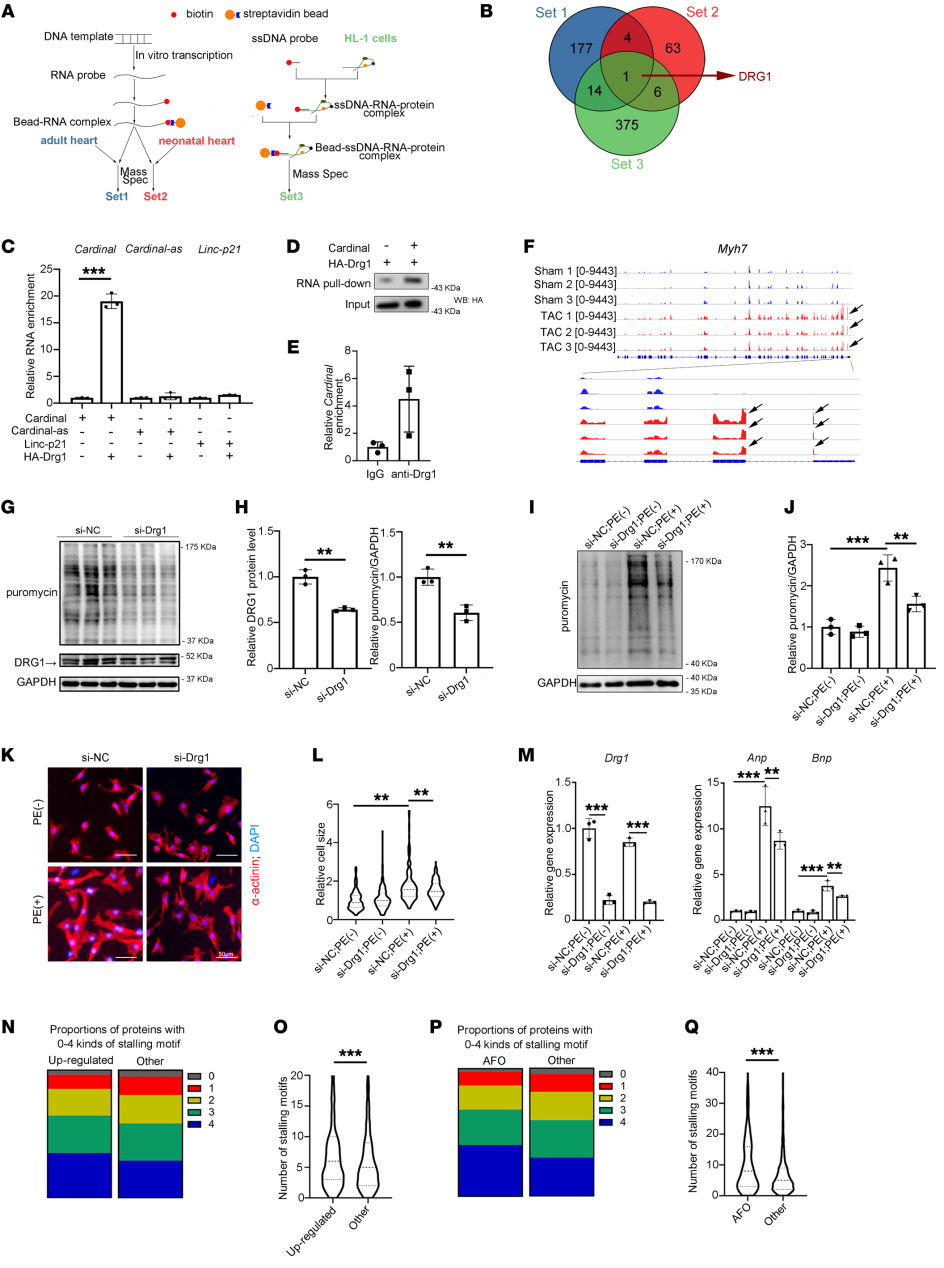
RNA interactome reveals that *CARDINAL* interacts with the translational regulator DRG1. (**A**) Designs for 3 sets of RNA pull-downs. (**B**) Venn diagram showing the *Cardinal*-interacting proteins. (**C**) Relative enrichment of *Cardinal, Cardinal-as*, and *Linc-p21* from HA-DRG1 and control IP (*n* = 3 for each group). Mass spec, mass spectrometry. (**D**) Western blot (WB) of HA-DRG1 in RNA pull-downs. HA, hemagglutinin. (**E**) Relative enrichment of *Cardinal* from IP in HL-1 cells. (**F**) Ribo-Seq coverages of hearts after sham surgery or 2 weeks after TAC surgery (PRJNA484227) over the *Myh7* genomic locus (*n* = 3 for each group). Black arrows show a potential ribosome stalling site. (**G**) Western blot and (**H**) quantification of DRG1 and puromycin-incorporated protein in HL-1 cells 48 hours after RNA interference. Cells were treated with 1 μM puromycin for 30 minutes before harvesting (*n* = 3 for each group). (**I**) Western blot and (**J**) quantification of puromycin-incorporated protein in NRVCs 24 hours after stimulation. Cells were treated by 1 μM puromycin for 30 minutes before harvesting (*n* = 3 for each group). (**K**) Immunofluorescence staining and (**L**) cell size quantification of NRVCs 48 hours after stimulation (*n* ≥300 for each group). Scale bars: 50 μm. (**M**) RT-qPCR results of relative gene expression in NRVCs 24 hours after stimulation (*n* = 3 for each group). (**I**–**M**) NRVCs were treated with si-NC or si-Drg1 and stimulated by culture medium with or without PE (50 μM). (**N**) Proportion of proteins with 0–4 categories of stalling motif among upregulated proteins versus the remaining proteins. (**O**) Violin plots showing the number of stalling motifs among upregulated versus the remaining proteins. (**P**) Proportion of proteins with 0–4 kinds of stalling motif among proteins in “actin filament organization” (AFO) gene set versus the remaining proteins. (**Q**) Violin plots showing numbers of stalling motifs among proteins in AFO gene set versus the remaining proteins. **P < 0.01 and ***P < 0.001, by 2-tailed Student’s *t* test (**C** and **H**), Mann-Whitney *U* test (**O** and **Q**), or 2-way ANOVA with Tukey’s post hoc test (**J**, **L**, and **M**). NC, negative control.

We hypothesized that *CARDINAL* functions by interacting with proteins associated with the ribosome and influencing protein translation. In order to understand the molecular mechanism of *CARDINAL* function and to identity its interacting proteins, we performed RNA pull-down experiments, followed by mass spectrometry. We performed these experiments in 3 independent conditions/settings to increase the specificity (Figure 6A). We used an in vitro–transcribed *Cardinal* probe labeled with biotin to pull down proteins from adult mouse hearts (set 1). Likewise, we used an in vitro–transcribed *Cardinal* probe labeled with biotin to pull down proteins from neonatal mouse hearts (set 2). And finally, we used a biotin-labeled DNA probe complementary to *Cardinal* to pull down proteins from a lysate of HL-1 cells (set 3). These 3 approaches yielded a single shared protein, DRG1 (Figure 6B and Supplemental Tables 5–8).

DRG1 has been implicated in a variety of biological functions, including an association with the polysome to regulate protein translation (45, 46). We verified the interaction of *Cardinal* and DRG1 using RNA immunoprecipitation (RIP) followed by RT-qPCR (Figure 6C). Additionally, we performed RNA pull-down followed by Western blotting to confirm the interaction between *Cardinal* and DRG1 (Figure 6D). Endogenous DRG1 RIP in HL-1 cells also resulted in *Cardinal* enrichment (Figure 6E). As a control, the *Cardinal* antisense (*Cardinal-as*) and an unrelated lncRNA, Linc-p21, did not interact with DRG1 (Figure 6C and Supplemental Figure 11, A and B). Interestingly, a recent study found that DRG1 suppresses ribosomal stalling on mRNA, therefore promoting efficient translation (46). To better understand the function and mechanism of DRG1 in cardiomyocytes, we performed DRG1 protein pull-down in neonatal cardiomyocytes, followed by mass spectrometry. Proteins enriched in precipitate pulled down by DRG1 compared with the negative control were considered DRG1-interacting proteins; a known DRG1 partner, DFRP1, was pulled down by DRG1 but not the negative control, demonstrating the efficacy of the assay (Supplemental Table 9).

We found that a large subset of DRG1-interacting proteins were ribosome-associated components (Supplemental Figure 11, C and D and Supplemental Table 9). Although this result provides further support that DRG1 may mediate ribosome activity and protein translation in the heart, ribosomal proteins are a common contaminant in IP tandem mass spectrometry (IP-MS/MS) studies. The control construct (Ad-GFP) was included for comparison to ensure the specificity of the interaction of these proteins with DRG1. In addition, we found that during cardiac hypertrophy induced by TAC surgery, there was a dramatic increase in the ribosome footprints near the start codon of *Myh7*, a transcript expressed in the ventricular wall that is dramatically induced during cardiac hypertrophy (Figure 6F). These results suggest that regulation of ribosome stalling is a possible mechanism for the translational changes observed during cardiac hypertrophy.

To directly test the function of DRG1 during translation, *Drg1* was knocked down in HL-1 cardiomyocytes and observed a decrease in protein translation (Figure 6, G and H). We also determined that *Drg1* knockdown suppressed the PE-induced increase in the translation rate in NRVCs (Figure 6, I and J), resulting in reduced cardiomyocyte size and decreased expression of the hypertrophic markers *Anp* and *Bnp* (Figure 6, K–M). On the basis of these results, we propose a role for DRG1 in cardiac hypertrophy: specifically, our data support a model in which the lncRNA *CARDINAL* and the ribosomal protein DRG1 interact to regulate protein translation during cardiac hypertrophy.

These data suggested that *CARDINAL* might regulate ribosome stalling in cardiac hypertrophy. Previously, multiple conserved amino acid motifs have been reported to be tightly associated with the occurrence of ribosome stalling (47). When we compared the upregulated proteins with the other proteins identified by the mass spectrometry analyses, stalling motifs were enriched in upregulated proteins both in terms of motif categories and motif numbers (Figure 6, N and O). The results suggested that loss of *CARDINAL* led to suppression of ribosome stalling.

Next, we asked why the translation of proteins related to “actin filament organization” were specifically enhanced. A similar analysis of stalling motifs showed that they were enriched in “actin filament organization” proteins both in terms of motif categories and motif numbers (Figure 6, P and Q). These results further suggested that translation of “actin filament organization” proteins were more likely affected by ribosome stalling and, therefore, that more translation of “actin filament organization” genes occurred in *Cardinal*-KO stressed hearts as a result of the suppression of ribosome stalling via DRG1.

### CARDINAL destabilizes DRG1 by preventing its interaction with DFRP1.

**Figure 7:**
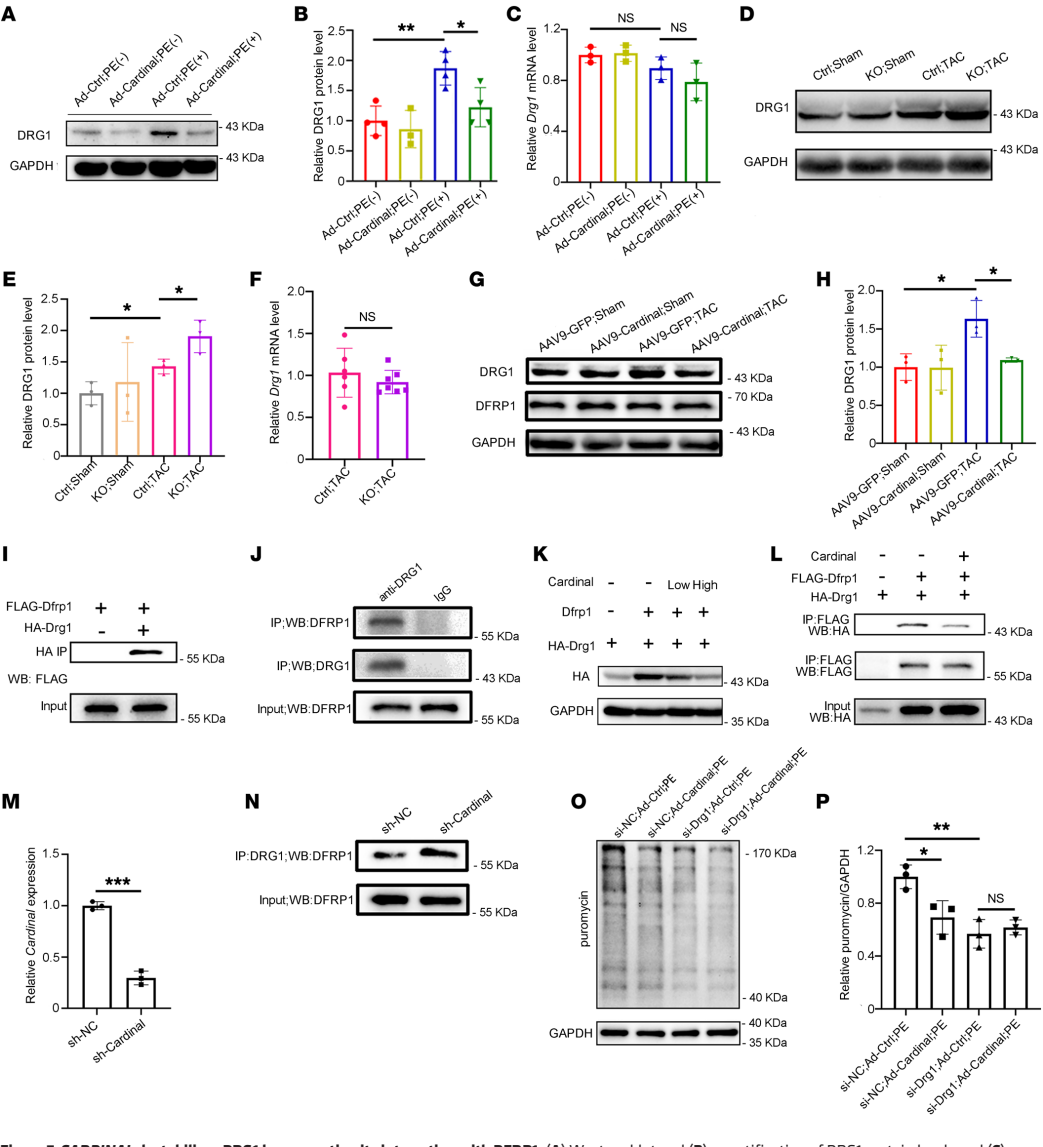
*CARDINAL* destabilizes DRG1 by preventing its interaction with DFRP1. (**A**) Western blot and (**B**) quantification of DRG1 protein levels and (**C**) quantification of *Drg1* mRNA levels detected by RT-qPCR in NRVCs infected with control virus or Ad-*Cardinal* and treated with or without PE for 48 hours (50 μM) (*n* ≥3 for each group). (**D**) Western blot and (**E**) quantification of DRG1 protein levels (*n* = 3 for each group) and (**F**) quantification of *Drg1* mRNA levels (*n* ≥6 for each group) detected by RT-qPCR in hearts from control or *Cardinal*-KO mice 4 weeks after sham or TAC surgery. (**G**) Western blot and (**H**) quantification of DRG1 protein levels in hearts from mice injected with AAV9-*GFP* or AAV9-*Cardinal* 4 weeks after sham or TAC surgery (*n* = 3 for each group). (**I**) Western blot of immunoprecipitated product and input in 293T cells showing the interaction between DRG1 and DFRP1. (**J**) Western blot of anti-DRG1 and IgG immunoprecipitated product and input in HL-1 cells. (**K**) Western blot of HA-DRG1 in 293T cells transfected with HA-Drg1 plasmid with or without cotransfection of *Dfrp1*and *Cardinal* plasmid. (**L**) Western blot of immunoprecipitated product and input of 293T cells showing the effect of *Cardinal* on DRG1-DFRP1 interaction. The amount of transfected plasmid was carefully titrated to ensure comparable inputs in the presence or absence of *Cardinal*. (**M**) Relative *Cardinal* expression levels detected by RT-qPCR in sh-NC and sh-*Cardinal* HL-1 cells (*n* = 3 for each group). (**N**) Western blot of anti-DRG1 immunoprecipitated product in stably knocked-down *Cardinal* (sh-*Cardinal*) and its control (sh-NC) HL-1 cells. (**O**) Western blot and (**P**) quantification of puromycin-incorporated protein in NRVCs with the indicated treatment and PE stimulation for 9 hours (*n* = 3 for each group). *P < 0.05, **P < 0.01, and ***P < 0.001, by 2-tailed Student’s *t* test (**F** and **M**) or 2-way ANOVA with Tukey’s post hoc test (**B**, **C**, **E**, **H**, and **P**).

To better understand the molecular mechanisms underlying the observed *CARDINAL*-DRG1 interaction and their function in cardiac hypertrophy, we further examined the expression and function of DRG1 protein in hypertrophic cardiomyocytes. In NRVCs, PE treatment increased the amount of DRG1 protein, which was attenuated by *Cardinal* overexpression (Figure 7, A and B). However, *Cardinal* overexpression did not affect the mRNA level of *Drg1* transcripts (Figure 7C). DRG1 protein levels were also increased in TAC-stressed hearts and further increased in *Cardinal*-KO hearts after the TAC procedure (Figure 7, D and E). As with the PE treatment, there was no observed change in *Drg1* mRNA levels in *Cardinal*-KO hearts when compared with the control (Figure 7F). In contrast, *Cardinal* overexpression in cardiomyocytes suppressed the increase in DRG1 protein expression in the TAC-stressed heart (Figure 7, G and H). These data revealed an inverse correlation between the expression pattern of the *Cardinal* transcript and DRG1 protein levels in cardiomyocytes in response to stress.

DFRP1 is an interacting partner of DRG1, which stabilizes DRG1 protein via their direct interaction (48). We first tested whether changes in DRG1 protein levels resulted from changes in DFRP1 protein levels. However, we found that *Cardinal* KO or overexpression did not result in obvious changes in DFRP1 levels after either sham or TAC surgery (Figure 7G and Supplemental Figure 12, A–C). Therefore, we hypothesized that *CARDINAL* downregulates DRG1 protein levels by interfering with its interaction with DFRP1. We first validated the interaction between DRG1 and DFRP1 by both exogenous and endogenous co-IP (Figure 7, I and J). Overexpression of *Dfrp1* increased the amount of DRG1 protein (Figure 7K), consistent with a previous report showing that DFRP1 stabilizes DRG1 protein (48). As expected, *Cardinal* overexpression inhibited the effect of DFRP1 on DRG1 protein levels in a dose-dependent manner (Figure 7K), but it did not affect DRG1 protein levels in the absence of DFRP1 (Supplemental Figure 12D). Next, we tested the effect of *Cardinal* on the interaction between DRG1 and DFRP1 by co-IP and demonstrated that co-IP in the presence of *Cardinal* attenuated the interaction between DRG1 and DFRP1 proteins (Figure 7L). As a control, overexpression of *Cardinal-as* did not affect DRG1-DFRP1 interaction (Supplemental Figure 12E). We further tested this finding using endogenous co-IP. We induced stable knockdown of *Cardinal* in HL-1 cells (Figure 7M). With lower *Cardinal* expression, the interaction between DRG1 and DFRP1 became stronger (Figure 7N). In order to test whether DRG1 is required for *CARDINAL* to influence protein translation and cardiac hypertrophy, we assessed its activity upon *Drg1* knockdown. Both *Cardinal* overexpression and *Drg1* knockdown decreased protein translation in NRVCs under treatment with PE (Figure 7, O and P). However, *Cardinal* overexpression in conjunction with *Drg1* knockdown did not further inhibit translation (Figure 7, O and P). This result supports a model for the inhibition of protein translation by *CARDINAL* through the downregulation of DRG1 protein levels.

## Discussion

**Figure 8:**
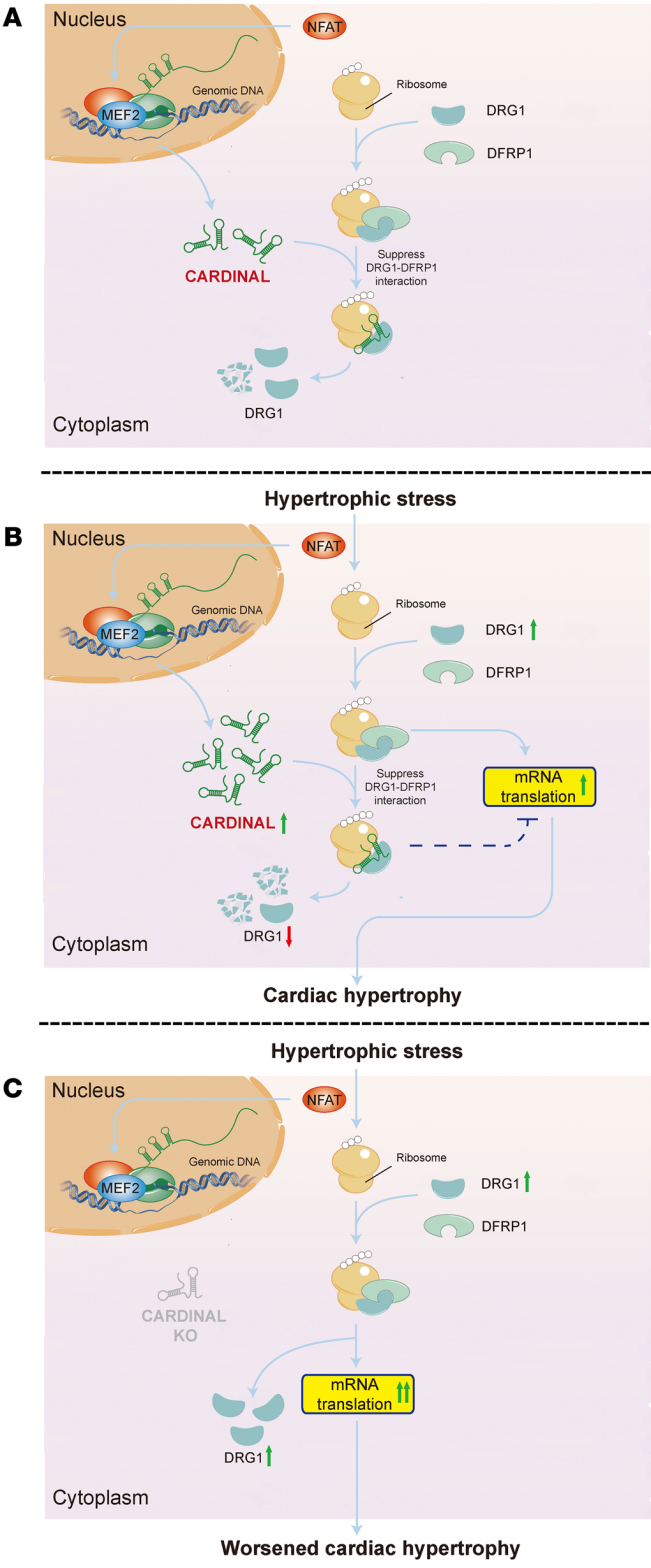
Proposed model for the regulation of mRNA translation and cardiac hypertrophy by *CARDINAL*. (**A**) *Cardinal* is a cardiac-specific lncRNA that can suppress mRNA translation. Under normal conditions, the expression of *Cardinal* and the ribosome-binding protein DRG1 (which promotes mRNA translation) are in balance. We propose that *CARDINAL* inhibits mRNA translation by interference with DRG1 function. *CARDINAL* binds DRG1 and interferes with the formation of the DRG1-DFRP1 stabilization complex; inhibition of DRG1-DFRP1 complex formation by *CARDINAL* results in reduced levels of DRG1, which helps maintain a normal level of translation. (**B**) Under stress conditions, both the lncRNA *CARDINAL* and DRG1 are upregulated. However, while *Cardinal* attempts to inhibit cardiomyocyte translation, it is no longer able to balance the increased translation induced by the increase in DRG1; the result is a net increase in mRNA translation and cardiac hypertrophy. (**C**) In the absence of *CARDINAL*, the constraint on DRG1 levels is lost. The result is an even greater elevation of protein synthesis and worsening of cardiac hypertrophy.

Here, we report the function of the cardiac-specific lncRNA *CARDINAL* in cardiac hypertrophy and protein translation. We found that *CARDINAL* modulated the process of translation and cardiac hypertrophy in response to stress by restraining the level and function of the translation regulator DRG1. We further demonstrated that the levels of DRG1 protein and its interaction with DFRP1 were increased under pathophysiological stress conditions; as a result, translational elongation was enhanced, increasing overall protein translation and inducing cardiac hypertrophy. Genetic deletion of *Cardinal* facilitated a more stable formation of the DRG1-DFRP1 complex, enhanced protein translation, and thereby increased cardiac hypertrophy. Our data reveal that the lncRNA *CARDINAL* regulated hypertrophic remodeling primarily under stress conditions (Figure 8, A–C). These findings further support the view that many lncRNAs are not essential for normal development or physiological function; instead, they are critical regulators for stress responses.

Translation enhancement is one of the distinct features of cardiac hypertrophy (4), and modulating this biological process is effective in suppressing cardiac hypertrophy (9–11). However, as translation is a basic cellular function in all cell types, organism-wide inhibition of translation leads to serious side effects. The fact that the heart has one of the lowest protein synthesis rates among different tissues compounds the problem (49). The harmful influence of inhibiting translation globally would outweigh any beneficial effect observed in a single tissue or cell type. Since many lncRNAs have higher tissue specificity than protein coding genes, a cardiac-specific lncRNA with the ability to regulate ribosomal activity could facilitate a targeted therapy to treat cardiac hypertrophy.

Since lncRNAs do not have protein-coding potential, they were not originally expected to be associated with the ribosome or to participate in protein translation. However, multiple translatomics techniques, including Ribo-Seq (19, 31), translating ribosome affinity purification–sequencing (TRAP-Seq) (21), and RNA-Seq of polysome fractions from polysome profiling (25, 50), indicated that this was not the case and association was common; however, the biological implications of this association were not immediately understood (19). In this study, we identified a cardiac-specific, ribosome-associated lncRNA, *CARDINAL*, that influences protein translation. We provide evidence that this ribosome-associated lncRNA can affect ribosomal function and protein translation and, in turn, alter the severity of cardiac hypertrophy. Our study describes a promising therapeutic target for translation-based therapy of heart failure and also emphasizes the potential translation-regulatory role of ribosome-associated lncRNAs.

*CARDINAL* was previously identified by Anderson et al. and described to have a role in transcriptional regulation (22). They demonstrated that *CARDINAL* functioned in the nucleus by interacting with SRF. The investigators also demonstrated that the *Cardinal* KO exacerbated systolic dysfunction after myocardial infarction. While our data also confirmed the nuclear localization of *Cardinal*, we found that it was more highly abundant in the cytoplasm of cardiomyocytes. We then demonstrated an interaction between *CARDINAL* and the ribosome in the cytoplasmic compartment. Therefore, it appears that both nuclear and cytoplasmic *CARDINAL* are functional, although each likely acts using a different mechanism. The present study reveals an important function for *CARDINAL* in altering ribosomal function and, consequently, cardiac hypertrophy.

The first report describing DRG1 was published over 30 years ago (51). The most established feature of DRG1 is its association with the ribosome (48, 52). However, its molecular role in protein synthesis was not known until a recent study was published describing its function in yeast (46); the authors found that the attachment of DRG1 to ribosomes promoted efficient translation by suppressing ribosome stalling (46). When a translating ribosome meets a stall signal on a mRNA, it transforms into an “unproductive” conformation (53). The authors proposed that the binding of DRG1 stabilized the ribosome in a “productive” conformation that was competent to proceed further in the elongation cycle (46). Our study linked DRG1 to cardiac hypertrophy and demonstrated that the upregulation of DRG1 protein was, at least partially, responsible for the enhancement of translation observed during cardiac hypertrophy. It also functioned as a downstream target of *CARDINAL* in the regulation of ribosomal function. These data demonstrate that the ribosome is a highly dynamic organelle that is regulated by a complex network, especially during the development of cardiac hypertrophy. However, the details regarding the mechanism of *CARDINAL* function in ribosome stalling still need further elucidation. It will also be interesting to explore whether the suppression of ribosome stalling in *Cardinal*-KO hearts leads to compromised protein quality control.

In summary, our study identified a cardiac-specific, ribosome-associated lncRNA (*CARDINAL*). *CARDINAL* suppressed the increase in translation observed during cardiac hypertrophy and the associated pathology, and it suppressed the upregulation of DRG1 protein levels during cardiac hypertrophy by preventing its interaction with its stabilizing partner DFRP1. This study highlights an important role for this lncRNA in the protein translation of cardiomyocytes and provides a feasible therapeutic target to treat cardiac hypertrophy by specifically modulating ribosomal function in the heart.

## Methods

### Sex as a biological variable.

For experiments involving humans, our study examined tissue from both men and women, and similar findings are reported for both sexes. For experiments involving mice, our study examined only male animals, since they exhibited less variability in phenotype.

### Human samples.

Left ventricular (LV) tissues were collected from patients with dilated cardiomyopathy (DCM) during heart transplantation performed in the First Affiliated Hospital of Sun Yat-sen University. When diseased hearts were removed from patients, a piece of LV tissue was dissected and snap-frozen in liquid nitrogen. LV tissue from donors who died for noncardiac reasons were used as a control.

### Animal models.

Cardiac hypertrophy was induced by transverse aortic constriction (TAC) surgery as described previously (54). Mice with a body weight of 25–30 g were anesthetized with isoflurane (3%–4% isoflurane for induction, 1%–2% isoflurane for maintenance). The chest was shaved and disinfected with alcohol. The chest was opened by left second intercostal thoracotomy. A 26 gauge needle was placed onto the ascending aorta. The needle and the ascending aorta were tightly ligated together using a 7-0 nylon suture at the transverse aorta, and the 26 gauge needle was removed immediately after ligation. In the sham operation, all procedures were the same except that no ligation was performed. The dissected intercostal space and chest skin were closed using a 6-0 silk suture. The surgeon was blinded to the mouse genotypes. Cardiac hypertrophy was assessed 4 weeks after surgery. *CnA*-Tg mice [Tg(Myh6-Ppp3ca)37Eno/J], were obtained from The Jackson Laboratory (strain no. 009075). In this strain, the Myh6 promoter drives expression of a constitutively active calcineurin A (Ppp3ca) cDNA in cardiomyocytes, serving as another model for cardiac hypertrophy.

### Statistics.

The mean and SD are presented for each measurement unless otherwise stated. Normality of data was evaluated by the Shapiro-Wilk test where warranted. For comparison between 2 groups, a 2-tailed Student’s *t* test was performed if the variable followed normal distribution, whereas a Mann-Whitney *U* test was performed if it did not follow a normal distribution. For comparisons among multiple groups, either 1-way or 2-way (if there were 2 factor levels) ANOVA was performed for variables with normal distribution, otherwise a Kruskal-Wallis H test was performed. For pairwise comparisons, post hoc tests were performed with Tukey’s correction. *P* values of less than 0.05 were considered statistically significant.

### Study approval.

All animal experiments were approved by Independent Ethics Committee for Clinical Research and Animal Trials of the First Affiliated Hospital of Sun Yat-sen University (protocol [2019]018) and the IACUCs of Boston Children’s Hospital (protocol 18-08-3759R) and the University of South Florida (protocol IS00009392). All procedures conformed to the 1964 Declaration of Helsinki and its later amendments or comparable ethics standards and were approved by the Ethics Committee of the First Affiliated Hospital of Sun Yat-sen University, Guangzhou, China.

The detailed experimental methods are available in Supplemental Methods. Sequences of primers used in this study for RT-qPCR are summarized in Supplemental Table 10.

### Data availability.

Supporting data values associated with the graphs in the main manuscript and the supplemental material are provided in the Supporting Data Values file. Values for each figure are presented in separate tabs. Next-generation sequencing data generated in this study have been deposited in the Genome Sequence Archive (GSA) in National Genomics Data Center (NGDC) of the Chinese Academy of Sciences (https://ngdc.cncb.ac.cn/gsa; GSA accession: CRA014575) for Ribo-Seq and in the NCBI’s Gene Expression Omnibus (GEO) database (GEO accession: GSE210985) for RNA-Seq.

## Author contributions

ZPH, DZW, and JH conceived the project, designed, and analyzed the experiments, and wrote the manuscript. XH, TY, and YWL performed molecular biology experiments and collected most of the data. HZ, TL, and YY contributed to human sample acquisition and Western blot analysis. GD and QM performed transverse aortic constriction surgery and collected mouse heart samples. CL contributed to echocardiographic data acquisition and analysis. MZ and ZL contributed to histological and immunofluorescence data acquisition and analysis. GW contributed to bioinformatics analyses of deep-sequencing data. HC, WP, YD, and JO supervised the *Cardinal*-KO mice generation and surgery. HC, WTP, YD, JO, and JDM reviewed and edited the manuscript. The determination of the order of the names of the co–first authors was made on the basis of each individual’s contribution to the figures and writing of the manuscript.

## Supplementary Material

Supplemental data

Unedited blot and gel images

Supporting data values

## Version 1 05/14/2024

In-Press Preview

## Version 2 07/01/2024

Electronic publication
