# Supplementary material for: The long noncoding RNA CARDINAL attenuates cardiac hypertrophy by modulating protein translation
Source: J Clin Invest. 2024 May 14;134(13):e169112. doi: 10.1172/JCI169112 (PMC11213465; doi:10.1172/JCI169112)
Supplement: Supplemental data [file jci-134-169112-s236.pdf]

Supplementary figure legend

Supplementary Figure 1 The multi-organ, developmental expression profile of mouse orthologues of 5 included and 3 excluded human lncRNAs. Each dot represented a developmental stage (e10.5, e11.5, e12.5, e13.5, e14.5, e16.5, e17.5, e18.5, P0, P3, P14, P28, P63 from left to right). Plots were generated in https://apps.kaessmannlab.org/lncRNA_app.

Supplementary Figure 2 Expression profile of Cardinal in different mouse organs, and relative level of Cardinal in hearts from different developmental stage detected by RT-qPCR.

Supplementary Figure 3 Positive control of single-molecule RNA FISH using Neat1 probes in (**A**) HL-1 cells and (**B**) cardiomyocytes from adult mice.

Supplementary Figure 4 Design of Cardinal-KO mice. **A**, Strategy of Cardinal knock-out. **B**, Design of the PCR primers for genotyping. **C**, A representative genotyping result.

Supplementary Figure 5 Validation of CRISPR/Cas9 editing using Sanger sequencing. **A**, Genomic sequence of WT mice at the target region. **B**, Results of Sanger sequencing of KO mice at target genomic region.

Supplementary Figure 6 Evaluation of potential off-target editing using CRISPR/Cas9 strategy. Potential off-target sites were calculated using COSMID algorithm (Cradick, 2014) with gRNA sequences as input. Four off-target sites were predicted. Genomic sequences near these sites were amplified from a KO mouse and sequenced.

Supplementary Figure 7 Characterization of Cardinal-KO mice. **A**, Results of qPCR detecting Cardinal and its nearby gene Myocd in hearts from Cardinal-KO and Ctrl mice. **B**, Ventricular weight/body weight ratio of Cardinal-KO mice vs. Ctrl at 2-month-old. **C**, H&E staining of cross-sections of hearts from Cardinal-KO and Ctrl mice at 2-month-old. Bars=500nm.

Supplementary Figure 8 Rescue experiment in stressed Cardinal KO mice. **A**, Timeline for rescue

experiment. **B**, Relative Cardinal expression detected by RT-qPCR. **C**, Ventricular weight/body weight, **D,** H&E staining, **E,** Wheat Germ Agglutinin (WGA) staining, **F,** relative cardiomyocyte area quantification, **G,** Relative expression level of hypertrophy and fibrosis markers detected by RT-qPCR, **H**, Sirius Red staining, and **I,** relative fibrosis area quantification were performed with hearts 4 weeks after sham or TAC surgery as indicated. **J and K,** echocardiographic parameters were measured with mice 4 weeks after TAC surgery as indicated. (n=5 in each groups)

Supplementary Figure 9 Transcriptomic change of Cardinal-KO;TAC vs. Ctrl;TAC. Heatmap (**A**) and Principal Component Analysis (PCA) plot (**B**) of transcriptome of hearts from control of Cardinal-KO mice 4 weeks after sham or TAC surgery. Three hundred and six differentially expressed genes in Ctrl;TAC vs Ctrl;Sham ($|\log_2 FC|>1$ and $FDR<0.05$) were plotted in panel A. **C**, Summary of Gene Set Enrichment Analysis (GSEA) of Gene Ontology-Biological Pathway.

Supplementary Figure 10 Cardinal overexpression attenuated cardiac hypertrophy induced by pressure overload. Timeline of the study was similar to that of Figure 5A. AAV9-Cardinal-as was used as the control virus instead of AAV9-GFP. **A**, Ventricular weight/body weight, **B,** H&E staining, **C,** Wheat Germ Agglutinin (WGA) staining, **D,** relative cardiomyocyte area quantification, **E**, Sirius Red staining, and **F,** relative fibrosis area quantification were performed with hearts 4 weeks after sham or TAC surgery as indicated. **G,** Relative expression level of hypertrophy and fibrosis markers detected by RT-qPCR. **H,** Cardiac function (Fraction shortening, FS%) were measured with mice 4 weeks after TAC surgery as indicated. (n≥3 in each group)

Supplementary Figure 11 Cardinal interacts with ribosome regulator Drg1. **A**, Western blotting of HA-Drg1 in Cardinal-as or their or control pull-downs. **B**, Western blotting of HA-Drg1 in Linc-p21 or control pull-downs. **C**, Western blotting showing the enrichment of HA-DRG1 protein in HA IP. NRVCs were infected by Ad-Ctrl or Ad-HA-Drg1 and stimulated by PE (50nM) for 24

hours before harvest. **D**, Results of mass spectrometry of the enriched proteins in HA IP. Proportions of ribosome related protein in Drg1 interactome. Proteins were considered potential candidates of Drg1 interacting partner if number of peptides matched in Ad-HA-Drg1 group > 2 times in Ad-Ctrl group. DRG1 itself, keratin-like proteins, and immunoglobins were excluded. There were totally 80 candidates, among which 11 were reported ribosome interacting proteins (Simsek et al., 2017), 24 were ribosome components, and 3 were tRNA/rRNA modification enzymes.

Supplementary Figure 12 Experimental results showing specificity of Cardinal's effect on DRG1-DFRP1 interaction. **A**, Western blotting and **B,** quantification of DFRP1 protein level in hearts from control or Cardinal-KO mice 4 weeks after sham or TAC surgery. (N=3 for each group) **C**, Quantification of DFRP1 protein level in hearts from mice injected with AAV9-GFP or AAV9-Cardinal 4 weeks after sham or TAC surgery (Representative figure in Figure 7G. **D**, Western blotting of HA-DRG1 in 293T cells transfected by HA-Drg1 plasmid and Cardinal plasmid. **E**, Western blotting of immunoprecipitated product and input of 293T cells showing effect of Cardinal-as on DRG1-DFRP1 interaction.

Supplementary Figure 1

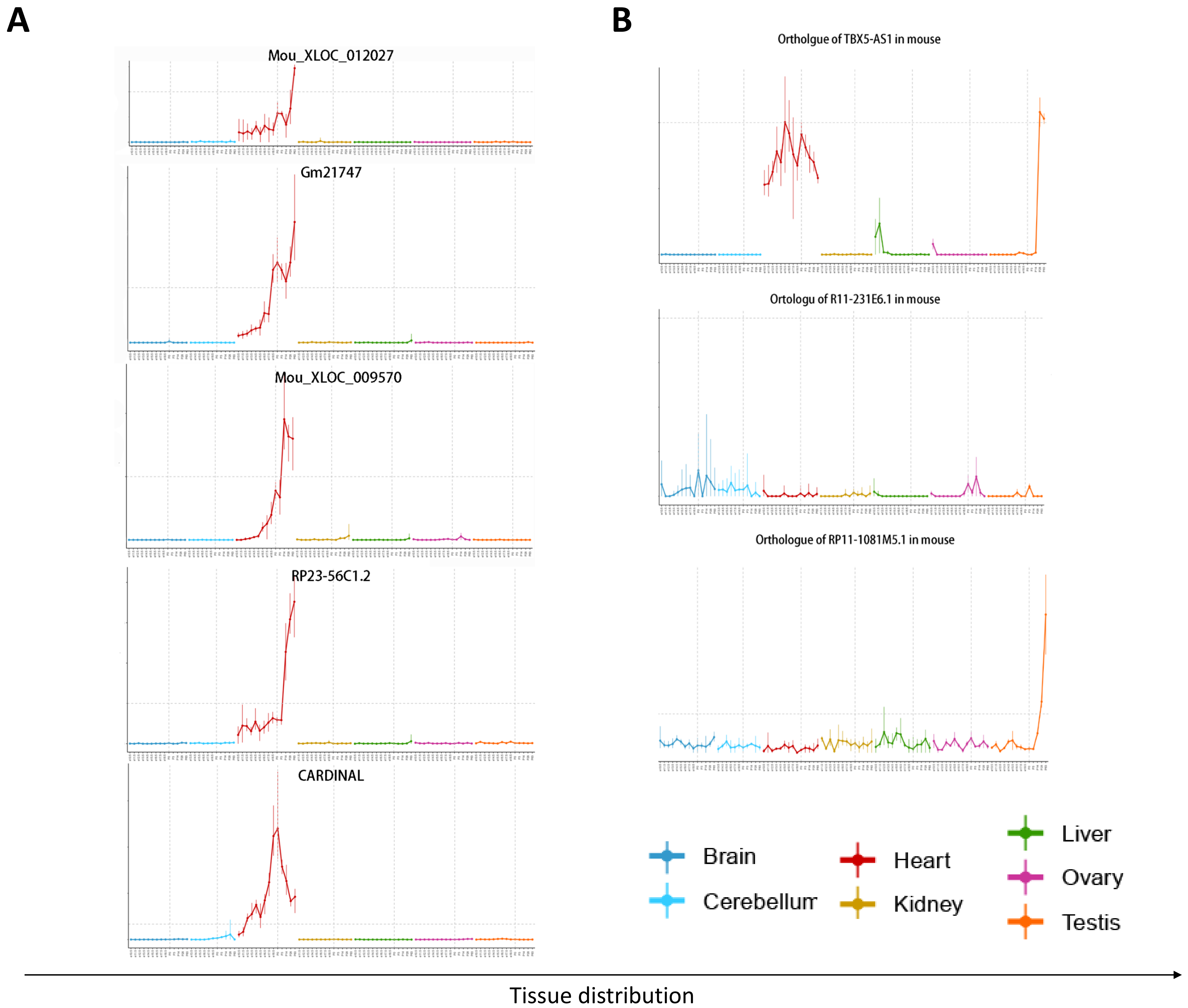

Supplementary Figure 2

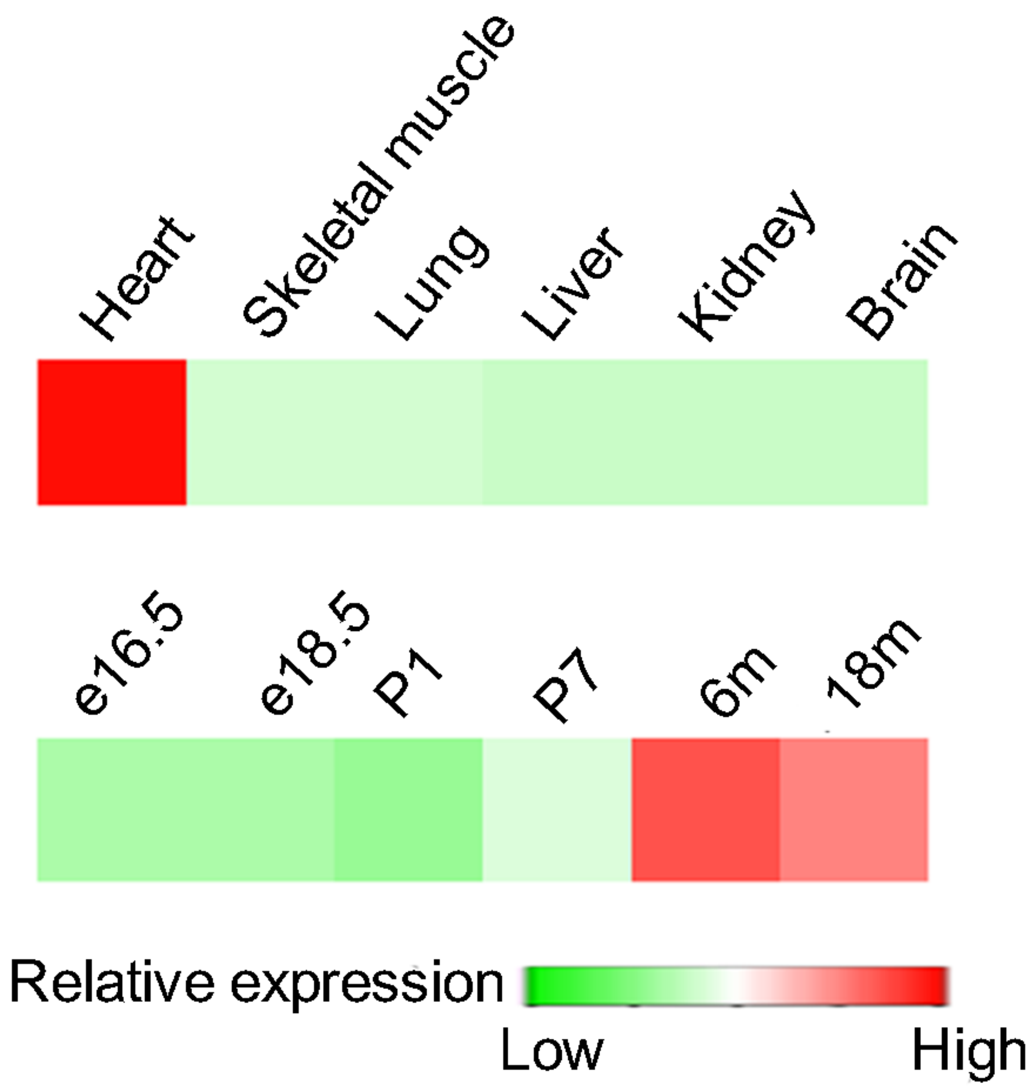

Supplementary Figure 3

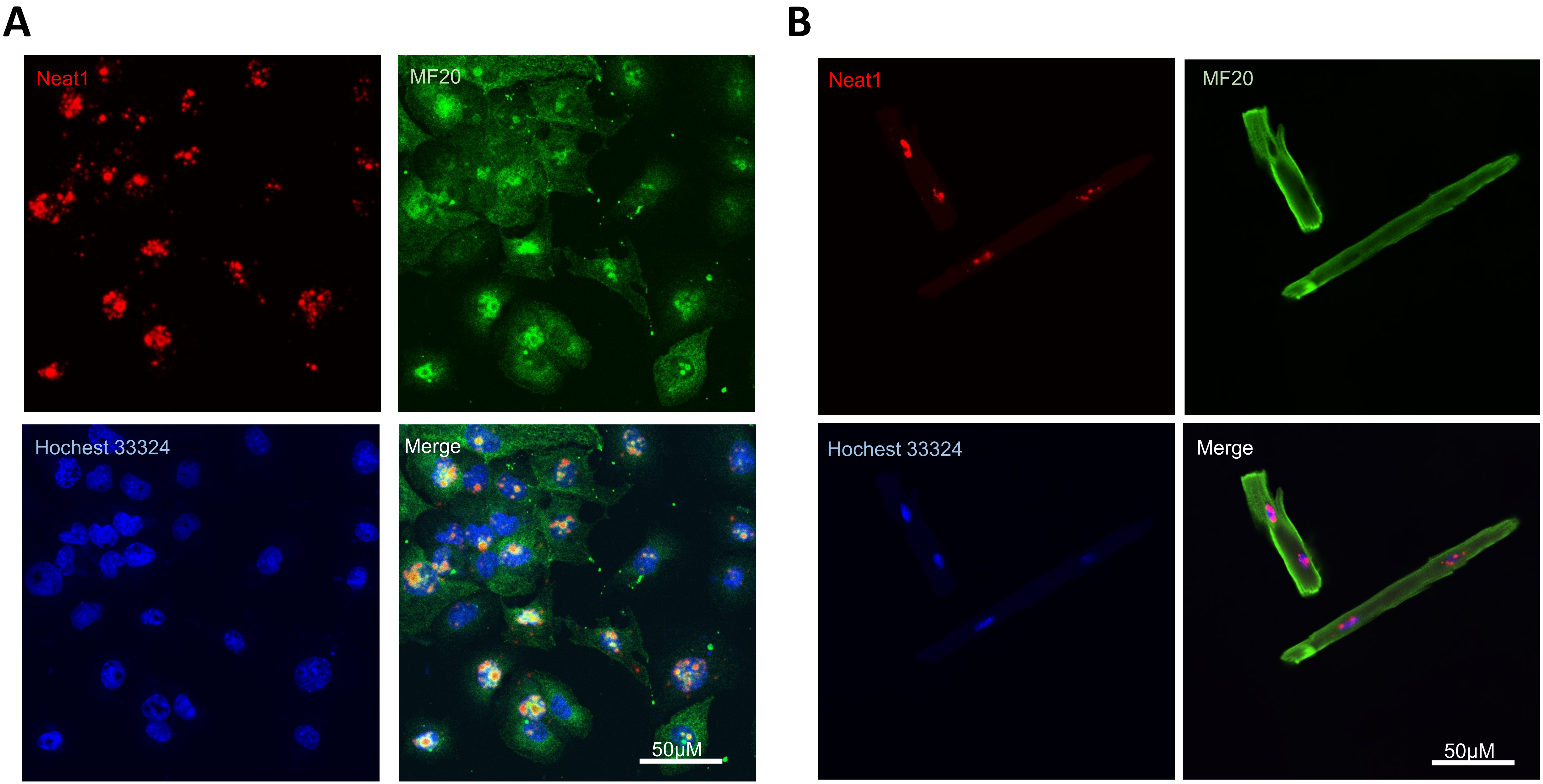

Supplementary Figure 4

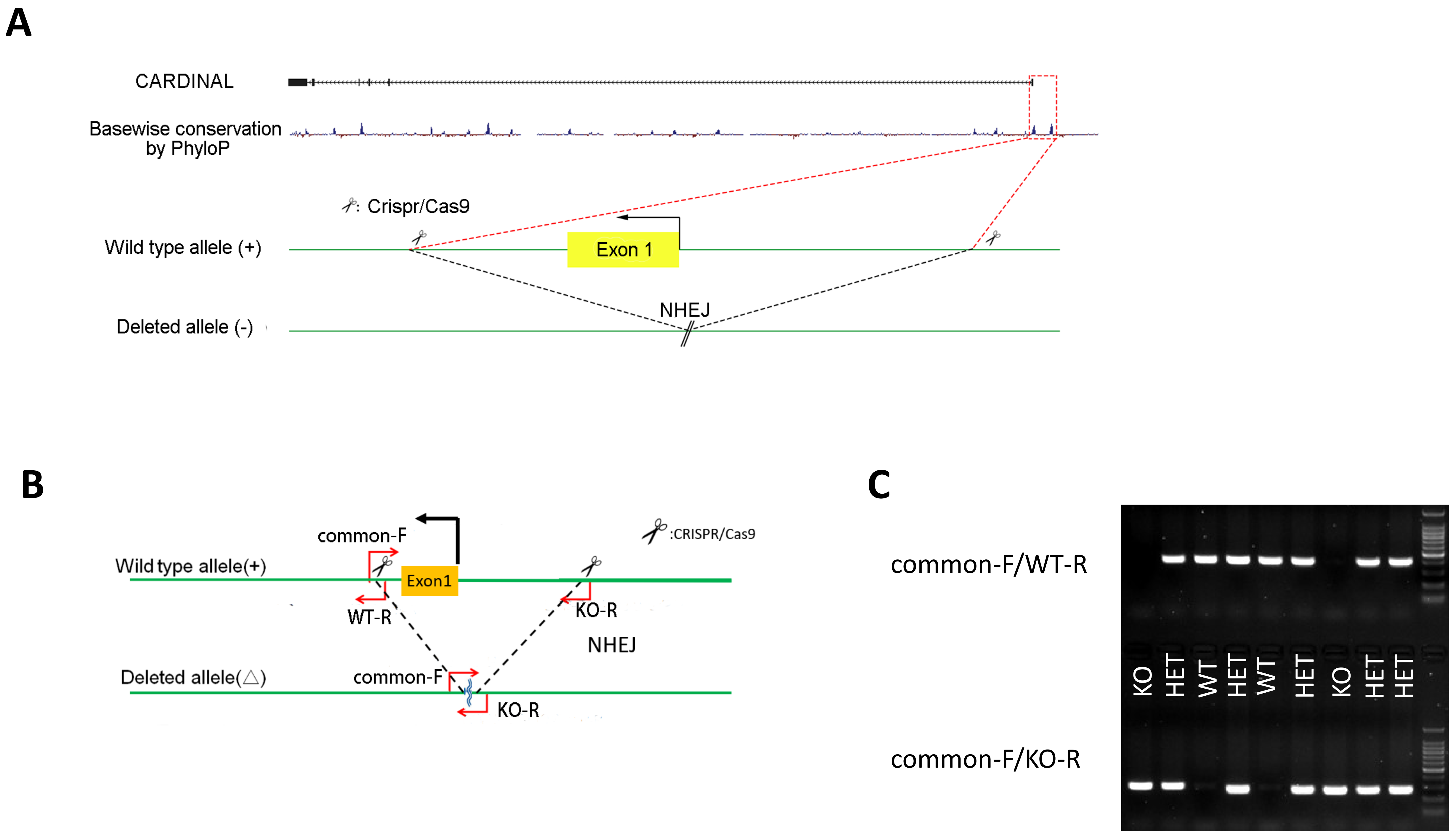

Supplementary Figure 5

A

5’ gRNA GTATACGCTACCCTCCTGTTAGG

3’ gRNA GAGGCCTCATTGACTAATGGAGG

WT

AAATGTCTACGACTATGGTCTGCCTGCCTCTCACTCTCATGACAGGTGTCATAGGCACAGACACTGGGATATTGCCGCAGTTCATCTGAGAACAAAGCAGGCGGTAAGTGC
CTAacaggagggtagcgtatacactgtgaacacgctgaccagaggacgagtcacggcatcggcatgggaatctgctacttaacatctaagtactgttcctttctgttattt
tccatttagtatttccgactgaaatttaccctgggtgtctgaagctgtggtaactggagctgctggaataggggtgagactatcacggtagccatgtgtggtctatcttc

---5K genomic DNA sequence---

ctggcctggagcaaaaggcttccagcttctcatctaatcttttacatctttggtttatgaccacacaacctgggatttgggactcaatagctaaaaatatgcctcctttgg
tagctggattttgttggttttcttttctttcgtgtttaacagaggcctcattgactaaTGGAGGAAAAACCACAAGAAATACCAGAGAAAACATATGGATATATGTCTCCT
AAGAGAAGGCTCACTTCTCTGCACAGAAAAACTCTGTGGAGATGACTAGAAAGTAGCCATT

B

5387 nt deletion

Founder #1

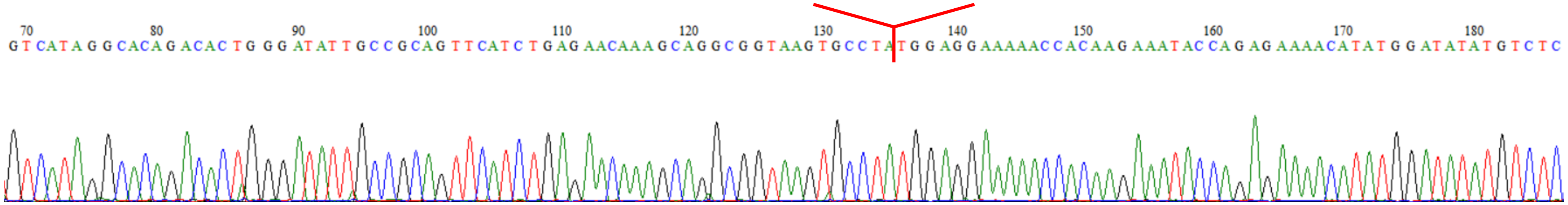

5411 nt deletion

Founder #2

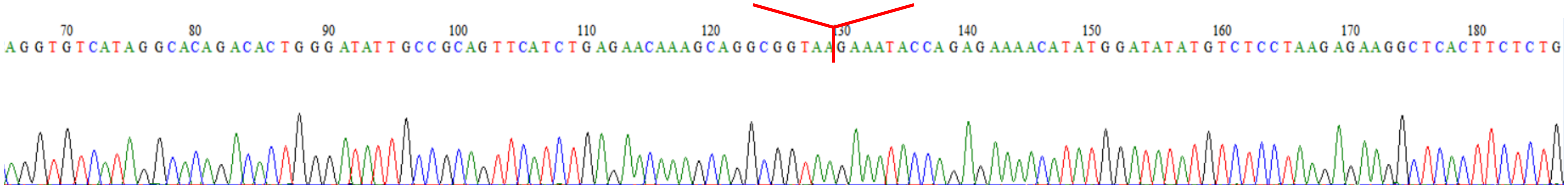

Supplementary Figure 6

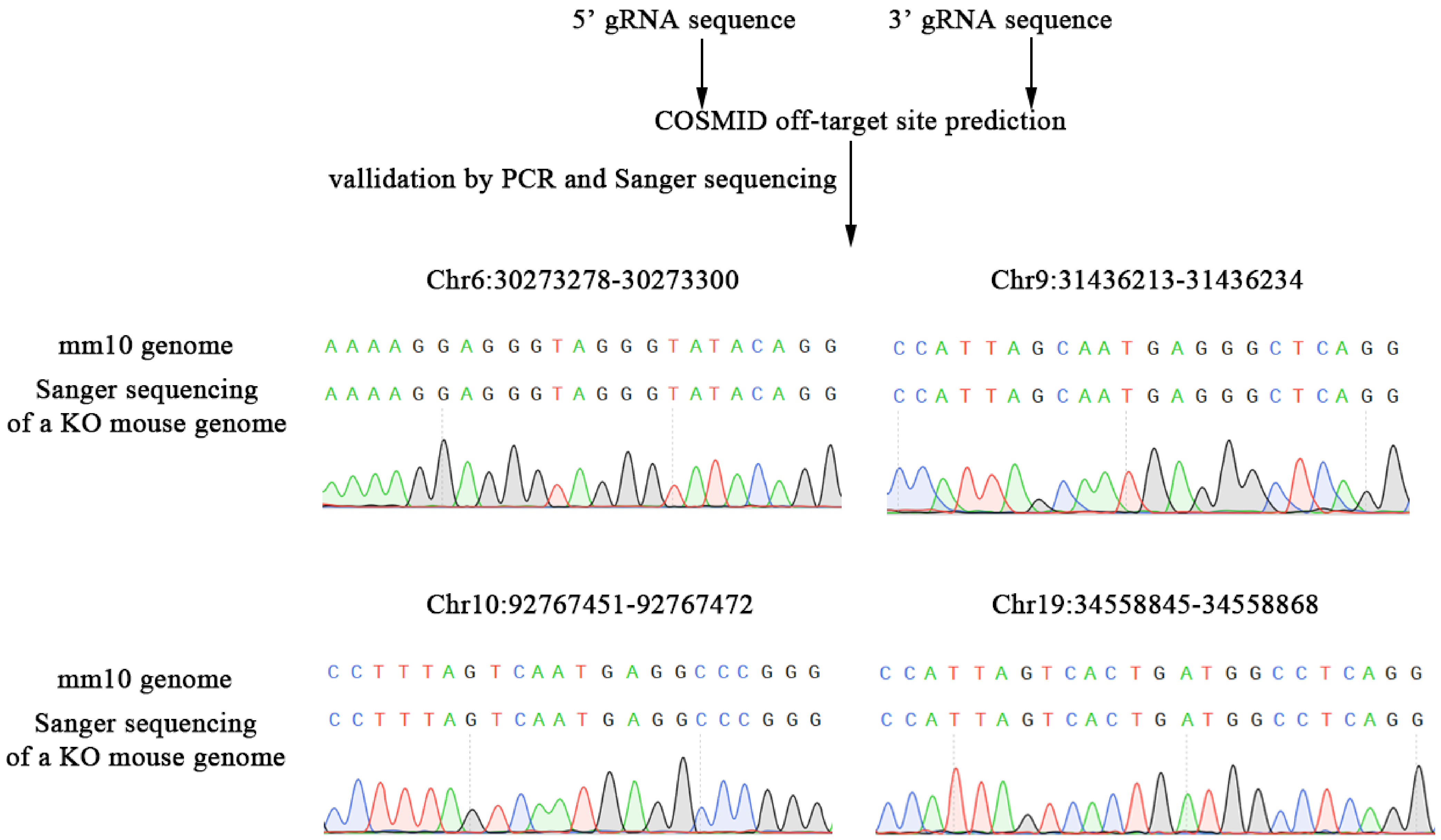

Supplementary Figure 7

A

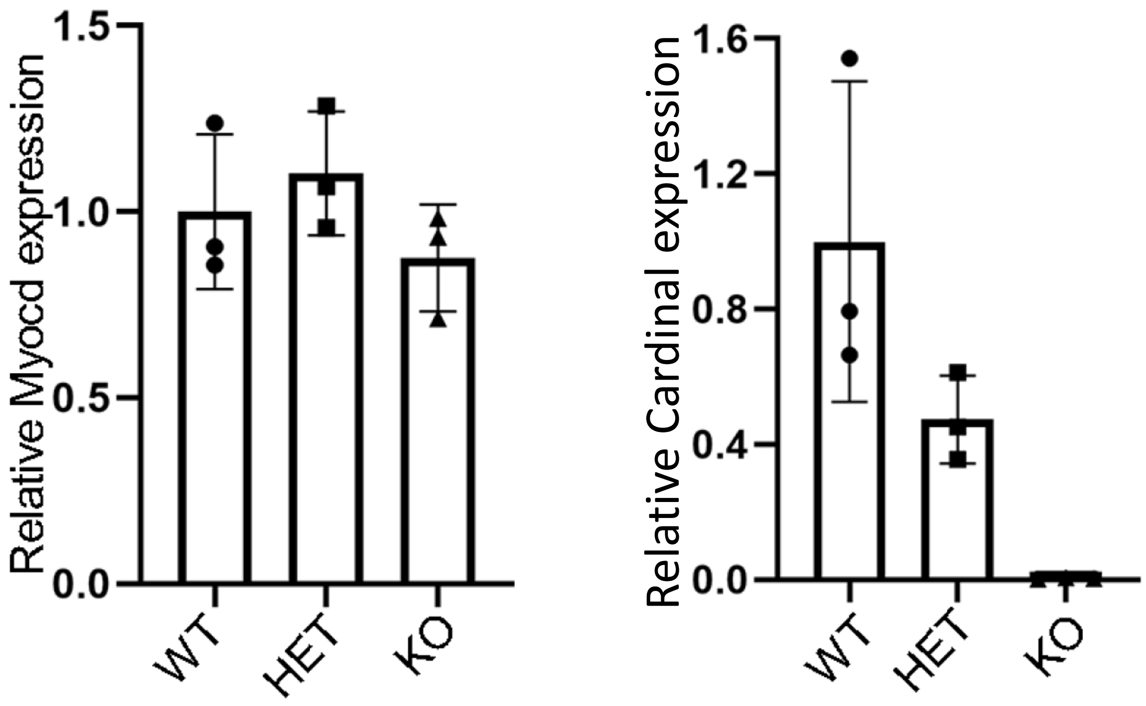

B

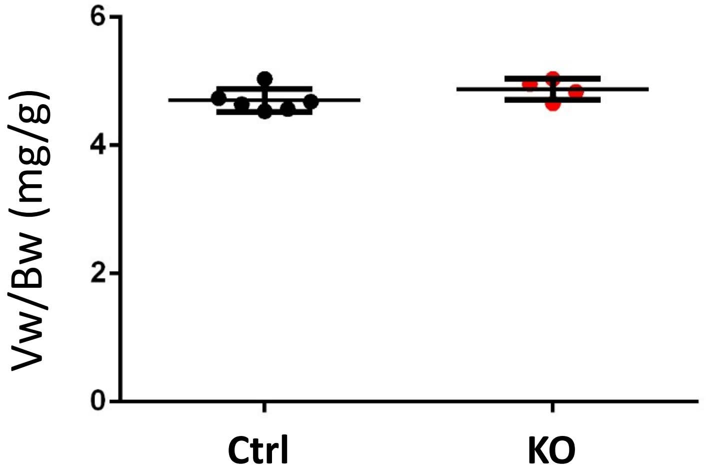

C

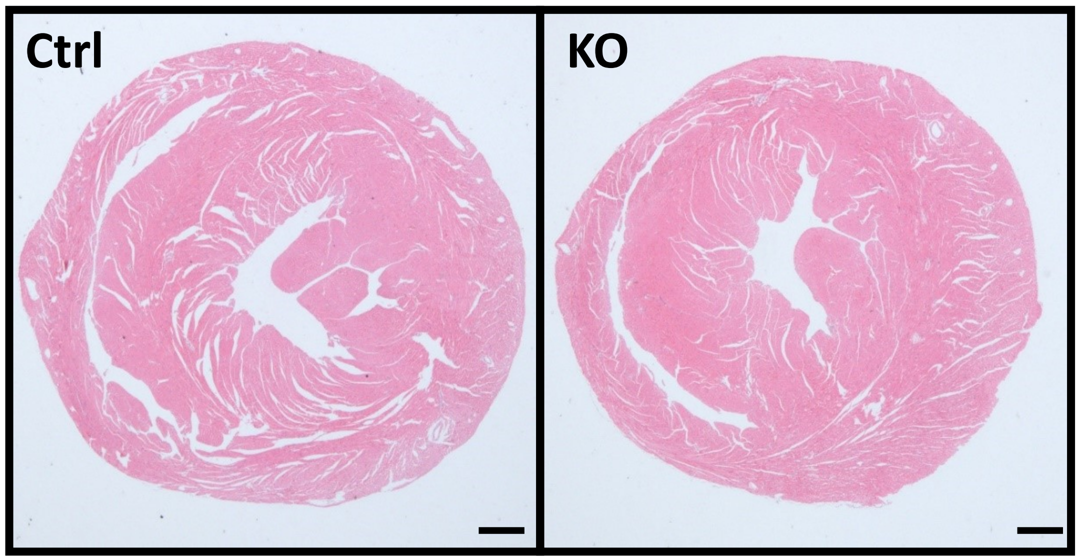

Supplementary Figure 8

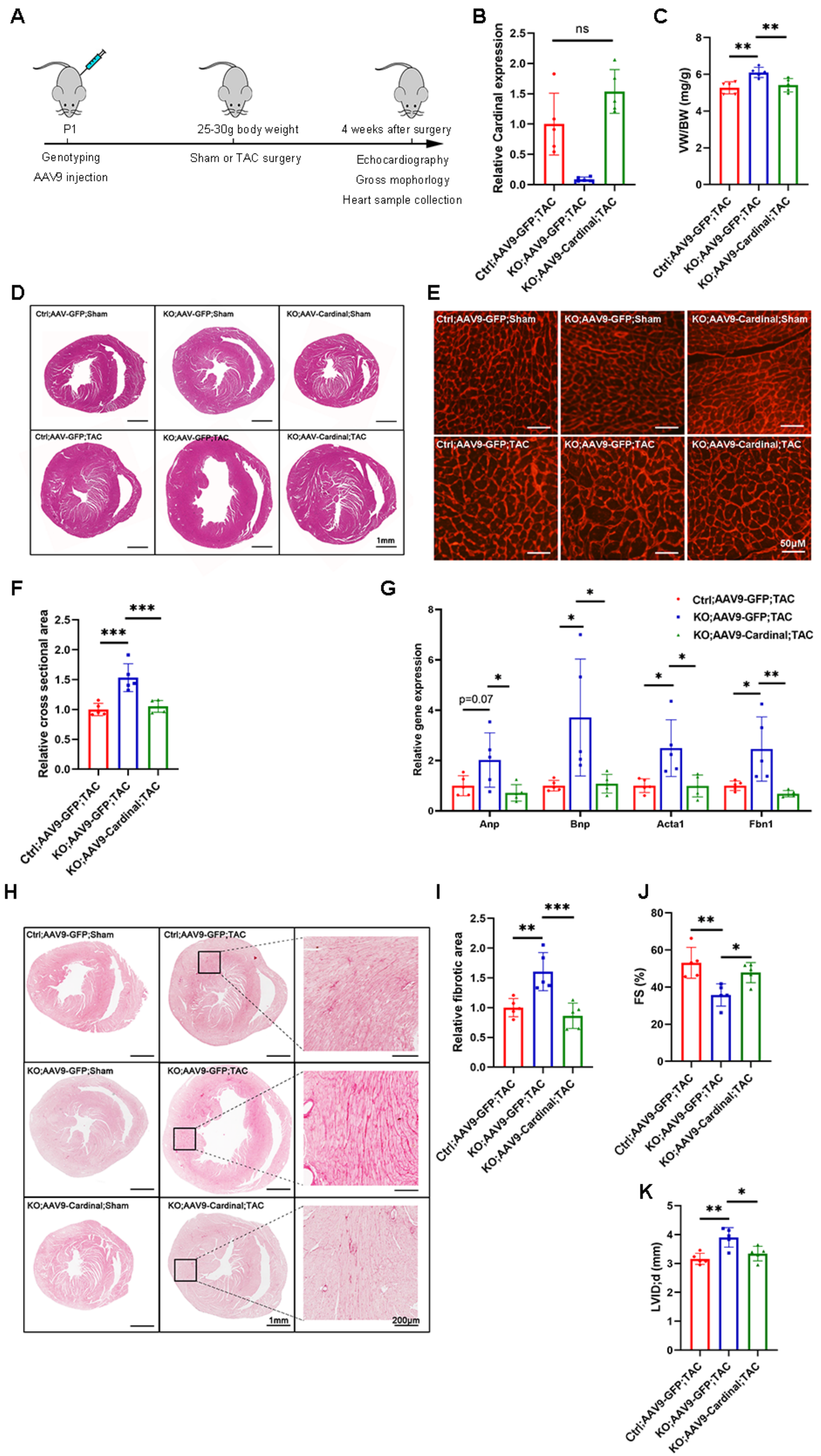

Supplementary Figure 9

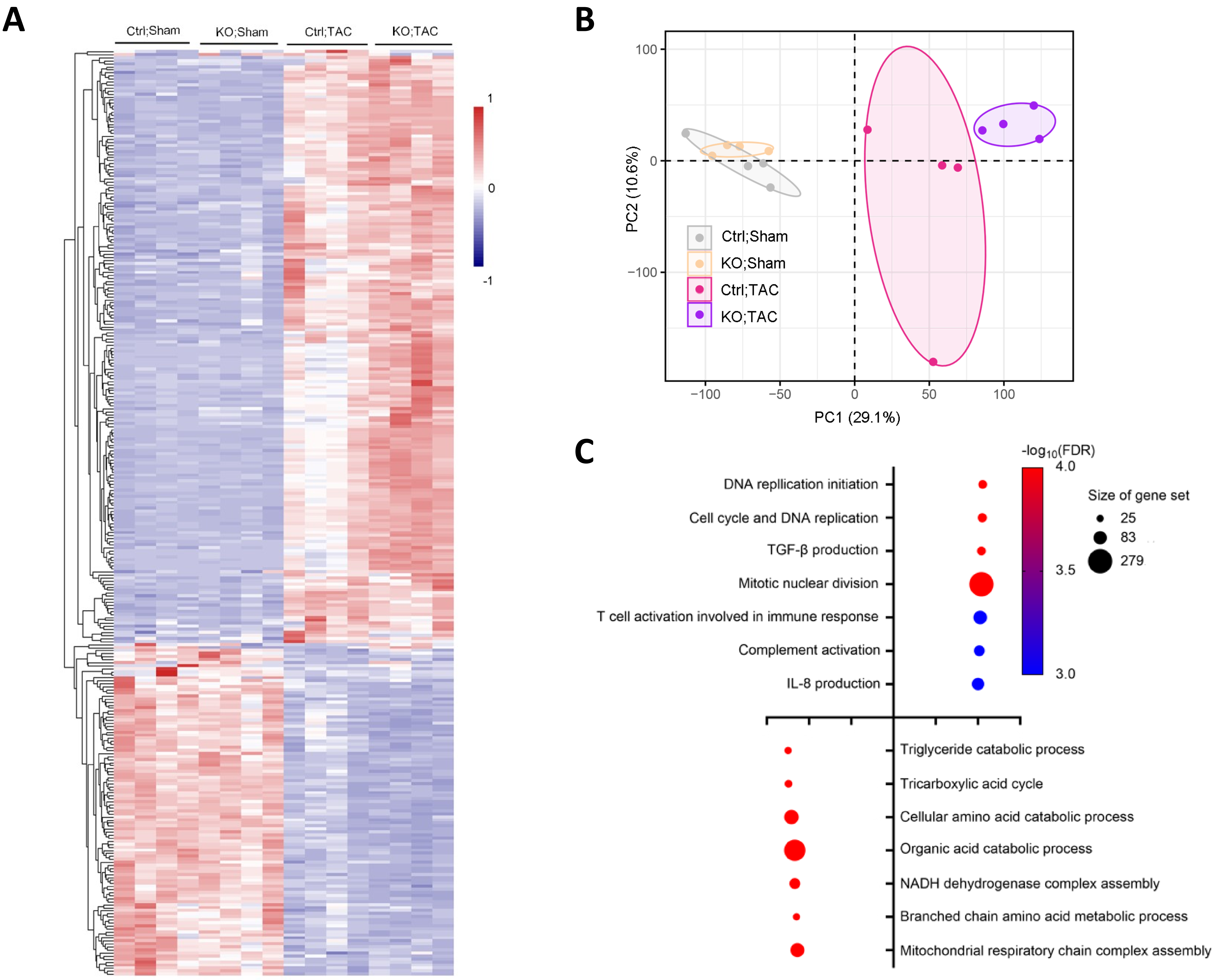

Supplementary Figure 10

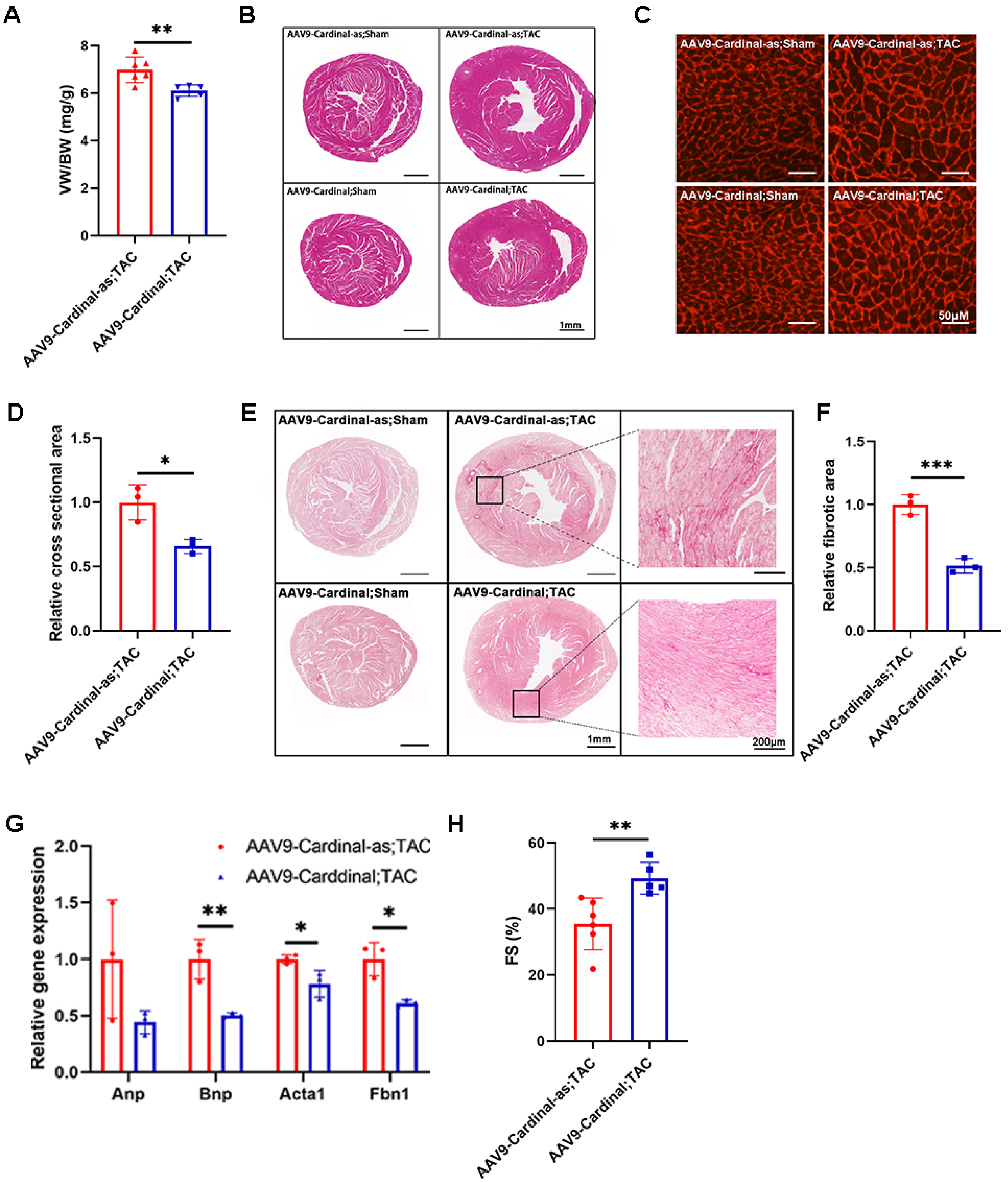

Supplementary Figure 11

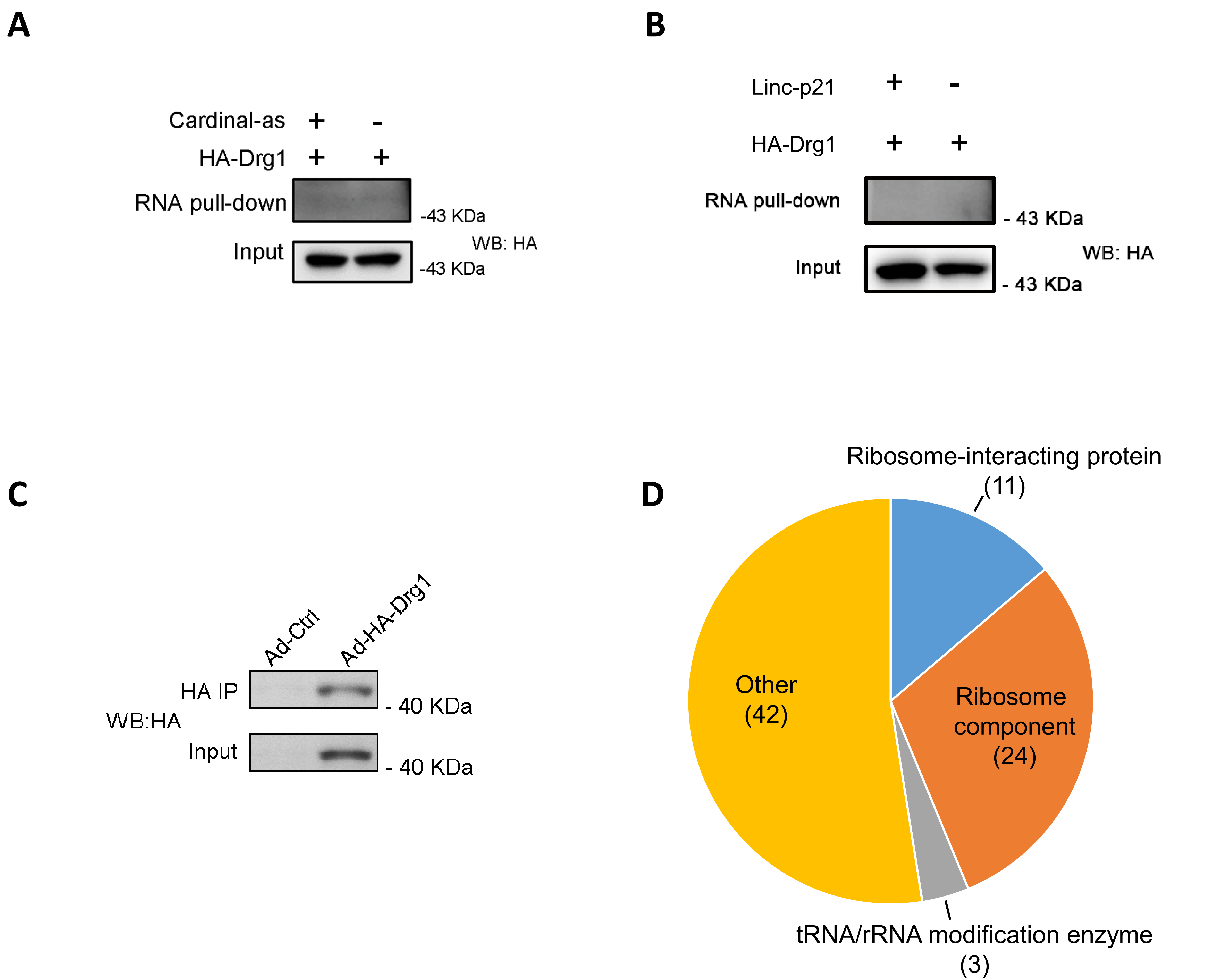

Supplementary Figure 12

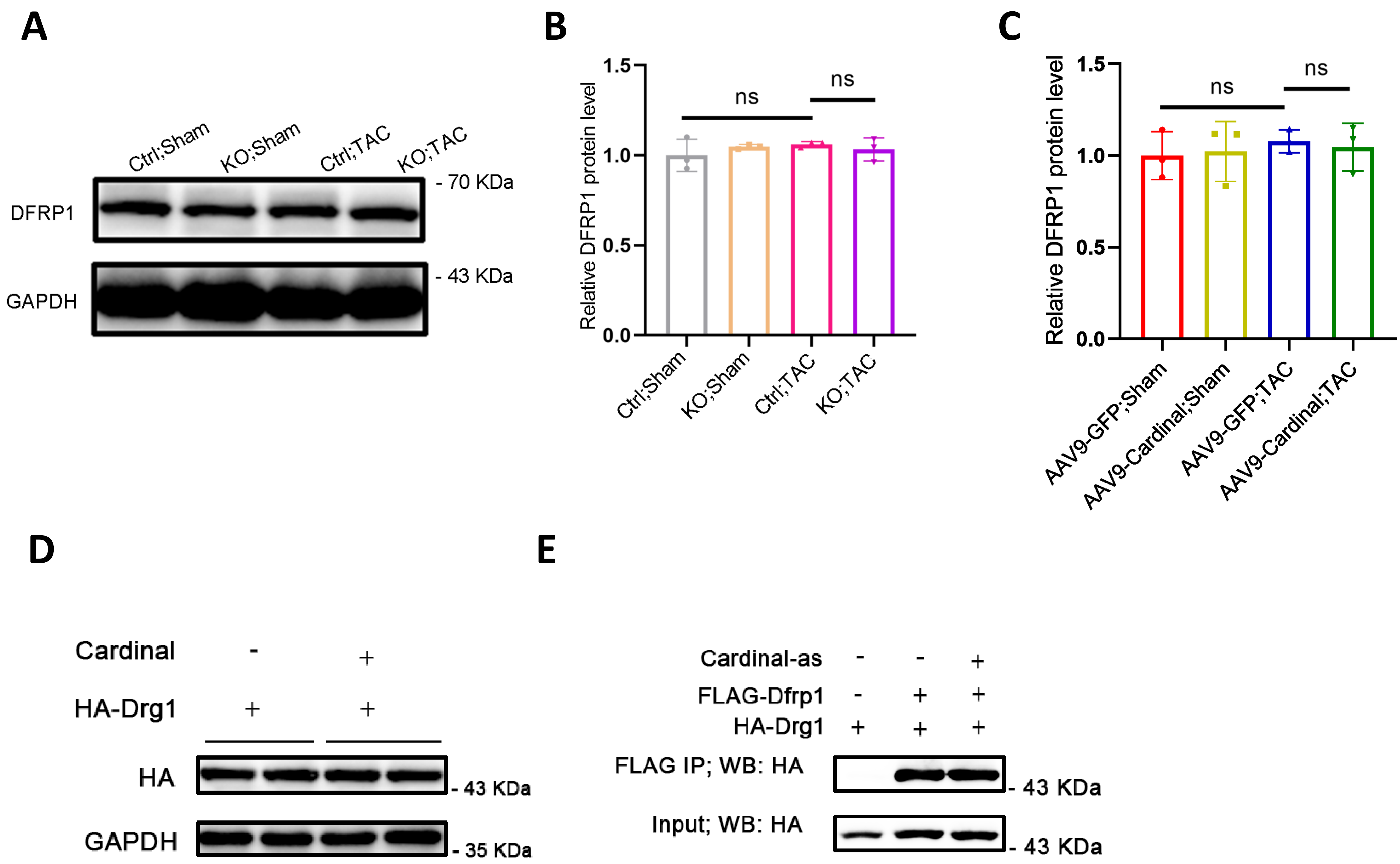

Supplementary Table 1 Echocardiography examination of 2-month-old CARDINAL-KO mice and their control littermates.

	WT (N=10)	KO (N=7)
**IVS;d (mm)**	0.744±0.043	0.768±0.036
**IVS;s (mm)**	1.377±0.067	1.458±0.115
**LVID;d (mm)**	3.387±0.166	3.325±0.162
**LVID;s (mm)**	1.776±0.059	1.756±0.105
**LVPW;d (mm)**	0.778±0.048	0.788±0.028
**LVPW;s (mm)**	1.386±0.106	1.436±0.085
**EF (%)**	79.91±1.77	79.66±1.79
**FS (%)**	47.49±1.94	47.17±1.81
**LV Mass (mg)**	83.75±7.05	83.88±8.31
**LV Mass (Corrected, mg)**	67.00±5.64	67.10±6.65
**LV Vol;d (uL)**	47.16±5.70	45.09±5.32
**LV Vol;s (uL)**	9.41±0.80	9.18±1.40
**Heart Rate (BPM)**	644±30	663±23

Supplementary Table 2 Echocardiography examination of CARDINAL-KO mice and their control littermates with transverse aortic constriction (TAC) or sham operation at different time points after surgery.

	Ctrl;Sham (N=6)		CARDINAL-KO;Sham (N=7)		Ctrl;TAC (N=12)		CARDINAL-KO;TAC (N=10)	
	2 wks	4 wks	2 wks	4 wks	2 wks	4 wks	2 wks	4 wks
**IVS;d (mm)**	0.733±0.019	0.749±0.043	0.742±0.031	0.742±0.066	0.958±0.074**	1.051±0.087**	1.003±0.062	1.014±0.112
**IVS;s (mm)**	1.425±0.113	1.399±0.111	1.416±0.070	1.368±0.116	1.682±0.083**	1.724±0.091**	1.633±0.112	1.518±0.276$^{\#}$
**LVID;d (mm)**	3.509±0.160	3.346±0.104	3.448±0.187	3.296±0.190	3.297±0.130**	3.314±0.209	3.423±0.241	4.278±0.646$^{\#\#}$
**LVID;s (mm)**	1.773±0.129	1.743±0.141	1.786±0.170	1.672±0.170	1.743±0.154	1.817±0.169	2.039±0.333$^{\#}$	3.162±0.888$^{\#\#}$
**LVPW;d (mm)**	0.760±0.028	0.760±0.019	0.752±0.031	0.758±0.068	0.941±0.050**	0.998±0.061**	0.980±0.048	0.969±0.115
**LVPW;s (mm)**	1.497±0.132	1.441±0.082	1.403±0.080	1.410±0.152	1.622±0.087*	1.613±0.139*	1.572±0.122	1.307±0.210$^{\#\#}$
**EF (%)**	81.60±2.90	80.31±3.23	80.59±1.52	81.67±3.08	79.63±3.18	77.57±3.27	72.04±8.12$^{\#\#}$	52.09±15.82$^{\#\#}$
**FS (%)**	49.45±3.13	47.95±3.24	48.23±1.52	49.33±3.21	47.20±3.02	45.21±3.29	40.67±6.28$^{\#\#}$	27.17±9.78$^{\#\#}$
**LV Mass (mg)**	86.42±6.96	81.12±5.72	84.02±7.11	79.24±14.02	110.23±10.04**	124.59±16.01**	124.84±18.39$^{\#}$	175.80±30.18$^{\#\#}$
**LV Mass (Corrected, mg)**	69.14±5.57	64.90±4.58	67.21±5.68	63.39±11.21	88.19±8.03**	99.67±12.80**	99.87±14.72$^{\#}$	140.64±24.14$^{\#\#}$
**LV Vol;d (uL)**	51.31±5.62	45.70±3.36	49.27±6.35	44.20±5.94	44.13±4.35**	44.85±6.77	48.56±8.11	84.60±32.88$^{\#\#}$
**LV Vol;s (uL)**	9.43±1.77	9.04±1.86	9.59±1.61	8.17±2.09	9.07±2.20	10.10±2.30	13.97±6.18$^{\#}$	44.40±32.50$^{\#\#}$
**Heart Rate (BPM)**	650±35	674±23	642±48	685±31	641±29	662±21	663±35	665±49

*: $P_{\text{Ctrl;Sham (same timepoint) vs. Ctrl;TAC (same timepoint)}}$ <0.05; **: $P_{\text{Ctrl;Sham (same timepoint) vs. Ctrl;TAC (same timepoint)}}$ <0.01; $^{\#}$: $P_{\text{Ctrl;AB (same timepoint) vs. CARDINAL-KO; TAC (same timepoint)}}$ <0.05; $^{\#\#}$: $P_{\text{Ctrl;TAC (same timepoint) vs. CARDINAL-KO;TAC (same timepoint)}}$ <0.01.

Supplementary Table 3 Differentially expressed genes in transcriptomic analysis of CARDINAL-KO and Ctrl hearts after TAC or sham surgery. Genes were displayed if FDR<0.05 and |logFC|>1 in Ctrl;TAC vs. Ctrl;Sham.

		Ctrl;TAC vs. Ctrl;Sham			CARDINAL-KO;TAC vs. Ctrl;TAC		
Gene_id	Gene_name	logFC	PValue	FDR	logFC	PValue	FDR
ENSMUSG00000040152	Thbs1	2.11115	1.59E-26	7.88E-24	0.91191	3.14E-15	8.01E-11
ENSMUSG00000027750	Postn	2.564828	6.31E-42	1.23E-38	1.211928	9.89E-15	1.26E-10
ENSMUSG00000032332	Col12a1	1.96964	1.84E-21	6.48E-19	1.310578	3.53E-14	3E-10
ENSMUSG00000021702	Thbs4	2.716662	3.26E-33	4.35E-30	1.432063	4.9E-13	3.12E-09
ENSMUSG00000042254	Cilp	3.421672	1.21E-33	1.70E-30	1.119939	5.35E-12	2.73E-08
ENSMUSG00000027656	Ccn5	1.8541	2.66E-34	3.96E-31	0.86684	2.72E-11	7.74E-08
ENSMUSG00000039899	Fgl2	1.052121	9.39E-21	3.17E-18	0.745809	2.73E-11	7.74E-08
ENSMUSG00000007682	Dio2	2.315708	1.33E-17	3.26E-15	1.641377	2.74E-10	5.65E-07
ENSMUSG00000030772	Dkk3	1.695149	1.69E-17	4.08E-15	1.038982	2.88E-10	5.65E-07
ENSMUSG00000022371	Col14a1	1.288708	1.01E-24	4.67E-22	0.640468	6.1E-10	1.04E-06
ENSMUSG00000056174	Col8a2	2.709332	6.29E-16	1.22E-13	1.344177	8.01E-10	1.15E-06
ENSMUSG00000031849	Comp	2.3579	9.18E-14	1.29E-11	1.63646	8.12E-10	1.15E-06
ENSMUSG00000049641	Vgll2	1.861287	9.82E-06	0.000289	1.870084	8.7E-10	1.17E-06
ENSMUSG00000033730	Egr3	1.9721	7.66E-13	9.29E-11	0.971033	4.26E-09	4.94E-06
ENSMUSG00000002020	Ltbp2	3.206479	1.32E-30	1.29E-27	1.274063	6.45E-09	6.86E-06
ENSMUSG00000037868	Egr2	2.263934	7.97E-06	0.000243	2.030756	7.35E-09	7.21E-06
ENSMUSG00000050359	Sprr1a	3.18894	5.15E-11	4.74E-09	1.827026	1.03E-08	9.39E-06
ENSMUSG00000074971	Fibin	1.886227	4.57E-43	1.05E-39	0.683762	1.5E-08	0.000012
ENSMUSG00000024529	Lox	1.560367	6.03E-15	1.02E-12	1.026032	1.75E-08	1.28E-05
ENSMUSG00000038600	Atp6v0a4	3.693018	1.01E-15	1.90E-13	1.372886	1.83E-08	0.000013
ENSMUSG00000007888	Crlf1	3.383122	1.39E-28	1.07E-25	1.161408	2.34E-08	1.57E-05
ENSMUSG00000031375	Bgn	1.387727	6.01E-29	4.91E-26	0.598181	3.16E-08	1.99E-05
ENSMUSG00000021091	Serpina3n	1.097036	2.75E-16	5.66E-14	0.707836	3.2E-08	1.99E-05
ENSMUSG00000027996	Sfrp2	1.517443	2.25E-11	2.15E-09	1.145914	5.09E-08	3.02E-05
ENSMUSG00000031595	Pdgfrl	1.324681	4.05E-09	2.77E-07	0.931096	6.35E-08	3.45E-05
ENSMUSG00000026042	Col5a2	1.609872	4.39E-44	1.39E-40	0.519123	7.03E-08	3.52E-05
ENSMUSG00000032334	Loxl1	1.23776	1.30E-22	5.01E-20	0.470112	8.11E-08	3.98E-05
ENSMUSG00000041559	Fmod	3.275419	1.44E-12	1.63E-10	1.588038	2.68E-07	0.000107
ENSMUSG00000056973	Ces1d	-1.98639	4.24E-24	1.82E-21	-1.12967	4.63E-07	0.000163
ENSMUSG00000000753	Serpinf1	1.401071	1.51E-23	6.27E-21	0.636537	4.53E-07	0.000163
ENSMUSG00000032925	Itgbl1	1.613731	1.74E-25	8.17E-23	0.630799	4.84E-07	0.000164
ENSMUSG00000027188	Pamr1	1.6448	3.55E-14	5.20E-12	0.798192	6.54E-07	0.000206
ENSMUSG00000047298	Kcnv2	-1.95431	1.02E-26	5.39E-24	-1.05887	7.7E-07	0.00024
ENSMUSG00000028369	Svep1	1.45962	6.02E-21	2.06E-18	0.622749	1.11E-06	0.00031
ENSMUSG00000027820	Mme	-1.00133	9.47E-13	1.13E-10	-0.69904	1.12E-06	0.000312
ENSMUSG00000054196	Cthrc1	2.010576	8.85E-05	0.00189	1.802516	1.21E-06	0.000333
ENSMUSG00000041616	Nppa	2.979091	8.41E-26	4.02E-23	1.183165	1.59E-06	0.000406
ENSMUSG00000035279	Ssc5d	1.028985	2.12E-14	3.28E-12	0.522078	1.8E-06	0.000438
ENSMUSG00000036256	Igfbp7	1.016739	3.04E-21	1.06E-18	0.502136	2.08E-06	0.000487

ENSMUSG00000022816	Fstl1	1.605913	5.40E-46	2.74E-42	0.445535	2.11E-06	0.000489
ENSMUSG00000074846	Gm10774	-5.69878	2.39E-06	8.63E-05	5.653538	2.54E-06	0.000568
ENSMUSG00000046402	Rbp1	1.291769	2.33E-13	3.03E-11	0.712154	3.33E-06	0.000686
ENSMUSG00000019997	Ccn2	1.84145	4.14E-28	2.83E-25	0.659094	3.49E-06	0.000712
ENSMUSG00000068196	Col8a1	2.269674	5.25E-53	6.65E-49	0.573534	4.03E-06	0.000804
ENSMUSG00000034205	Loxl2	1.646717	3.77E-43	9.56E-40	0.403418	5.09E-06	0.000947
ENSMUSG00000024593	Megf10	1.033548	0.002432	0.029159	1.058371	5.15E-06	0.000952
ENSMUSG00000028873	Cdca8	1.057989	8.32E-05	0.001788	1.043907	5.84E-06	0.001064
ENSMUSG00000042436	Mfap4	2.461993	1.51E-31	1.74E-28	0.846647	6.33E-06	0.001144
ENSMUSG00000004939	Nmrk2	1.144331	1.24E-05	0.000354	1.066599	6.55E-06	0.001146
ENSMUSG00000072949	Acot1	-1.29917	1.99E-12	2.19E-10	-0.92499	7.29E-06	0.001256
ENSMUSG00000029304	Spp1	1.264611	0.000328	0.005731	1.05436	7.74E-06	0.001307
ENSMUSG00000040705	A930016O22Rik	-1.06497	0.000563	0.009085	0.991904	9.3E-06	0.001511
ENSMUSG00000030208	Emp1	1.402745	7.17E-28	4.54E-25	0.48116	1.02E-05	0.0016
ENSMUSG00000026043	Col3a1	1.682212	9.98E-31	1.01E-27	0.393671	1.06E-05	0.001645
ENSMUSG00000093938	Evi2b	-2.41204	0.004833	0.04987	3.015443	1.37E-05	0.002013
ENSMUSG00000024481	Lvrn	-1.62042	5.28E-12	5.53E-10	-1.19442	1.42E-05	0.002071
ENSMUSG00000029661	Col1a2	1.249914	3.92E-24	1.71E-21	0.410112	1.47E-05	0.002099
ENSMUSG00000021567	Nkd2	1.278969	1.62E-08	9.76E-07	0.7909	1.69E-05	0.00226
ENSMUSG00000090877	Hspa1b	-3.2587	0.001479	0.019689	1.953167	1.69E-05	0.00226
ENSMUSG00000007122	Casq1	1.062883	8.46E-07	3.46E-05	-0.82044	1.76E-05	0.002334
ENSMUSG00000045573	Penk	-1.67696	9.41E-16	1.79E-13	-1.23347	2.13E-05	0.002662
ENSMUSG00000036545	Adamts2	1.284253	3.51E-29	3.17E-26	0.4371	2.34E-05	0.00288
ENSMUSG00000042734	Ttc9	1.080468	4.99E-06	0.000162	0.86029	2.35E-05	0.00288
ENSMUSG00000026051	Ecrg4	2.553582	0.000123	0.002495	1.708113	2.37E-05	0.00289
ENSMUSG00000030116	Mfap5	2.057578	1.19E-50	7.76E-47	0.537945	2.65E-05	0.003162
ENSMUSG00000049871	Nlrc3	2.286662	5.14E-28	3.43E-25	0.617292	2.84E-05	0.00332
ENSMUSG00000097652	Mhrt	-1.34713	7.92E-37	1.34E-33	-0.41125	3.11E-05	0.003558
ENSMUSG00000030562	Nox4	1.759078	5.14E-10	4.05E-08	0.990743	0.000031	0.003558
ENSMUSG00000001506	Col1a1	1.668108	4.38E-38	7.93E-35	0.354947	3.52E-05	0.003933
ENSMUSG00000029377	Ereg	2.591372	4.80E-06	0.000157	1.295594	4.29E-05	0.004593
ENSMUSG00000117110	Gm49808	3.06546	7.82E-07	3.23E-05	1.488291	4.51E-05	0.004774
ENSMUSG00000027204	Fbn1	1.598607	4.32E-30	4.05E-27	0.404917	4.54E-05	0.00478
ENSMUSG00000086806	Gm13054	3.887484	2.82E-14	4.23E-12	1.689812	4.66E-05	0.00489
ENSMUSG00000018593	Sparc	1.051163	1.54E-26	7.80E-24	0.359622	4.85E-05	0.005008
ENSMUSG00000021950	Anxa8	2.123326	2.99E-07	1.38E-05	1.238083	0.000057	0.005724
ENSMUSG00000029223	Uchl1	1.012712	2.02E-15	3.66E-13	0.518846	6.06E-05	0.005971
ENSMUSG00000038670	Mybpc2	1.727207	1.23E-50	7.76E-47	-0.43156	6.21E-05	0.006019
ENSMUSG00000031574	Star	1.077407	1.78E-06	6.68E-05	0.850667	6.57E-05	0.006252
ENSMUSG00000041482	Piezo2	1.129398	2.79E-06	9.80E-05	0.760747	6.57E-05	0.006252
ENSMUSG00000039137	Whrn	-1.0538	7.85E-15	1.28E-12	-0.5826	6.62E-05	0.006274
ENSMUSG00000077928	Mir208b	-1.15147	3.49E-14	5.14E-12	-0.71538	7.29E-05	0.006764
ENSMUSG00000031934	Panx1	1.277536	5.13E-10	4.05E-08	0.608798	7.38E-05	0.006799
ENSMUSG00000027004	Frzb	1.720291	7.92E-18	1.97E-15	0.637916	7.57E-05	0.006944
ENSMUSG00000027316	Gfra4	-1.06932	4.45E-08	2.44E-06	-0.76116	8.24E-05	0.007452

ENSMUSG00000054675	Tmem119	1.448632	2.32E-14	3.55E-12	0.543438	0.000083	0.007483
ENSMUSG00000093752	Gm20716	-1.85816	3.66E-05	0.000888	1.823575	9.49E-05	0.008119
ENSMUSG00000059049	Frem1	2.162563	1.28E-11	1.27E-09	0.966144	9.54E-05	0.008132
ENSMUSG00000030433	Sbk2	-1.27735	1.03E-06	4.12E-05	-1.14349	9.57E-05	0.008132
ENSMUSG00000096957	E230013L22Rik	1.001521	2.85E-14	4.25E-12	0.462162	0.000172	0.012738
ENSMUSG00000079465	Col4a3	1.494218	1.11E-18	2.95E-16	0.543696	0.000189	0.013733
ENSMUSG00000039457	Ppl	-1.29149	1.97E-13	2.60E-11	-0.80444	0.000189	0.013733
ENSMUSG00000063727	Tnfrsf11b	2.564415	7.97E-06	0.000243	1.299424	0.000229	0.015557
ENSMUSG00000079049	Serpinb1c	3.593966	3.62E-15	6.39E-13	1.025266	0.00023	0.015573
ENSMUSG00000090214	Gm15657	5.69173	8.99E-05	0.001915	-4.81921	0.000246	0.016391
ENSMUSG00000037370	Enpp1	1.15858	9.71E-11	8.48E-09	0.551724	0.000274	0.017668
ENSMUSG00000030717	Nupr1	1.192231	1.51E-06	5.84E-05	0.804129	0.000274	0.017668
ENSMUSG00000022025	Cnmd	-1.2199	8.72E-06	0.000263	-1.47068	0.000272	0.017668
ENSMUSG00000050010	Shisa3	1.313844	3.95E-08	2.20E-06	0.686769	0.000288	0.01826
ENSMUSG00000001493	Meox1	1.438381	1.38E-13	1.90E-11	0.581676	0.000302	0.018696
ENSMUSG00000031659	Adcy7	1.264239	1.61E-21	5.84E-19	0.413943	0.00051	0.026715
ENSMUSG00000028339	Col15a1	1.275165	2.62E-22	9.91E-20	0.345292	0.000545	0.028024
ENSMUSG00000025780	Itih5	1.020769	8.18E-10	6.24E-08	0.494227	0.000545	0.028024
ENSMUSG00000047485	Klhl34	1.380375	1.51E-11	1.47E-09	-0.64044	0.000556	0.028402
ENSMUSG00000031994	Adamts8	2.303623	4.28E-14	6.20E-12	0.754828	0.00059	0.029201
ENSMUSG00000032174	Icam5	3.373054	6.74E-05	0.001496	-2.51834	0.000599	0.029588
ENSMUSG00000096751	Gm28373	-5.19063	0.000172	0.00329	5.248026	0.000615	0.030267
ENSMUSG00000028613	Lrp8	2.693893	5.51E-12	5.70E-10	0.98541	0.000626	0.030577
ENSMUSG00000078815	Cacng6	-2.08417	1.22E-09	9.09E-08	-1.73637	0.000661	0.031455
ENSMUSG00000051279	Gdf6	1.665829	9.85E-13	1.16E-10	0.88372	0.0007	0.03236
ENSMUSG00000110496	Gm45909	-1.96193	1.93E-09	1.39E-07	-1.39373	0.000847	0.036851
ENSMUSG00000085277	Gm13943	1.248905	1.99E-12	2.19E-10	0.431713	0.000884	0.037961
ENSMUSG00000053964	Lgals4	-1.55844	2.06E-13	2.70E-11	-0.63714	0.000913	0.038773
ENSMUSG00000024011	Pi16	1.420195	2.44E-31	2.68E-28	0.34444	0.001028	0.042402
ENSMUSG00000028626	Col9a2	2.499571	1.79E-10	1.49E-08	1.010956	0.00105	0.043071
ENSMUSG00000037406	Htra4	1.119809	3.53E-08	1.99E-06	0.554308	0.001112	0.044648
ENSMUSG00000066755	Tnfsf18	3.745639	3.62E-05	0.000881	1.349291	0.001319	0.050368
ENSMUSG00000025271	Pfkfb1	-1.5974	5.91E-14	8.46E-12	-0.71609	0.001421	0.052881
ENSMUSG00000085272	Sbk3	-1.10301	0.002255	0.027546	-1.44653	0.001506	0.054928
ENSMUSG00000026343	Gpr39	1.970784	3.71E-07	1.67E-05	0.770794	0.001553	0.056166
ENSMUSG00000054626	Xlr	-1.35799	3.72E-05	0.000899	1.074707	0.001629	0.058333
ENSMUSG00000001131	Timp1	2.087676	5.81E-28	3.77E-25	0.604845	0.001678	0.059564
ENSMUSG00000063594	Gng8	1.476987	9.28E-06	0.000277	0.712572	0.001698	0.059905
ENSMUSG00000028307	Aldob	-1.7996	2.78E-05	0.000707	-1.50249	0.001754	0.060828
ENSMUSG00000032487	Ptgs2	1.937417	1.68E-12	1.88E-10	0.763628	0.001762	0.060956
ENSMUSG00000041670	Rims1	2.84936	0.001876	0.02375	-2.46037	0.001804	0.06201
ENSMUSG00000040690	Col16a1	1.347246	1.73E-24	7.83E-22	0.34653	0.001974	0.065045
ENSMUSG00000037411	Serpine1	1.224277	4.32E-09	2.92E-07	0.661581	0.001991	0.065354
ENSMUSG00000043719	Col6a6	-1.68526	1.88E-20	6.04E-18	-0.80974	0.002111	0.067896
ENSMUSG00000102160	Gm36944	4.930809	0.000502	0.008256	-3.46212	0.002137	0.068203

ENSMUSG00000090799	Klhl33	-1.28845	1.21E-22	4.70E-20	-0.44532	0.002246	0.06993
ENSMUSG00000043857	Mgat5b	2.661168	5.32E-14	7.66E-12	0.712123	0.002402	0.072841
ENSMUSG00000022425	Enpp2	-1.16066	1.03E-15	1.93E-13	-0.54586	0.002429	0.073313
ENSMUSG00000023505	Cdca3	1.239709	8.92E-05	0.001903	0.88302	0.002607	0.076945
ENSMUSG00000029093	Sorcs2	-1.04325	9.71E-07	3.91E-05	-0.70996	0.002611	0.076958
ENSMUSG00000074529	Zfp972	-1.28915	0.000582	0.009362	0.959452	0.002667	0.077748
ENSMUSG00000022754	Tmem45a	1.075246	2.03E-07	9.85E-06	0.455126	0.002818	0.08068
ENSMUSG00000028989	Angptl7	1.718394	8.14E-09	5.21E-07	0.521085	0.002947	0.083407
ENSMUSG00000041193	Pla2g5	-1.07478	1.93E-20	6.11E-18	-0.34832	0.003335	0.091538
ENSMUSG00000062248	Cks2	1.152377	4.44E-05	0.001049	0.880951	0.003358	0.09168
ENSMUSG00000024803	Ankrd1	2.132107	7.17E-44	2.02E-40	0.372285	0.003524	0.094998
ENSMUSG00000114800	Gm31600	-1.77071	9.56E-11	8.38E-09	-0.86578	0.003612	0.096055
ENSMUSG00000025386	Pde6g	-1.23764	0.001337	0.018249	-1.73336	0.003655	0.096588
ENSMUSG00000044933	Sstr3	1.035779	9.03E-06	0.000271	-0.49429	0.004038	0.102055
ENSMUSG00000067653	Ankrd23	1.67522	2.76E-28	2.06E-25	0.299169	0.004069	0.102462
ENSMUSG00000035486	Plk5	-1.05357	0.00063	0.010031	-1.08736	0.00439	0.108266
ENSMUSG00000026527	Rgs7	-1.32475	4.27E-08	2.35E-06	-0.839	0.004618	0.111675
ENSMUSG00000027331	Knstrn	1.117432	5.22E-06	0.000169	0.689789	0.004844	0.114704
ENSMUSG00000035860	Cdhr3	-1.35409	0.002271	0.027671	-1.3729	0.004853	0.114809
ENSMUSG00000029019	Nppb	2.494873	8.00E-22	2.94E-19	0.372712	0.004914	0.115485
ENSMUSG00000029862	Clcn1	-1.30203	1.50E-09	1.10E-07	-0.5568	0.004913	0.115485
ENSMUSG00000025727	A930017K11Rik	-1.70165	6.89E-05	0.001522	-2.12087	0.005192	0.120245
ENSMUSG00000018822	Sfrp5	-1.16794	0.001168	0.016478	-1.55001	0.005371	0.122697
ENSMUSG00000038508	Gdf15	2.051877	6.35E-10	4.93E-08	0.737181	0.005523	0.12447
ENSMUSG00000037254	Itih2	1.8234	1.06E-12	1.23E-10	0.564229	0.005805	0.128511
ENSMUSG00000117655	6720468P15Rik	5.082405	0.000295	0.005243	-2.85829	0.005851	0.129191
ENSMUSG00000021614	Vcan	1.819392	2.91E-42	6.14E-39	0.354753	0.006043	0.132399
ENSMUSG00000037544	Dlgap5	1.193982	2.00E-05	0.000537	0.922044	0.006148	0.133658
ENSMUSG00000000049	Apoh	2.511516	9.69E-05	0.002037	-1.53356	0.006765	0.141515
ENSMUSG00000063821	Dupd1	2.105199	0.001293	0.017833	-1.27296	0.007023	0.145037
ENSMUSG00000068587	Mgam	1.87721	3.38E-06	0.000116	0.736615	0.007138	0.14644
ENSMUSG00000029769	Ccdc136	1.472174	3.86E-15	6.69E-13	0.372329	0.007203	0.147311
ENSMUSG00000030483	Cyp2b10	-1.38793	0.001416	0.019017	-1.12063	0.007675	0.15291
ENSMUSG00000056220	Pla2g4a	1.203357	7.65E-09	4.95E-07	0.433745	0.007904	0.15564
ENSMUSG00000094526	Gm21451	1.662748	4.68E-27	2.78E-24	0.293721	0.008124	0.158392
ENSMUSG00000032218	Ccnb2	1.116313	3.49E-06	0.000119	0.594095	0.008116	0.158392
ENSMUSG00000022657	Cd96	5.744641	3.16E-06	0.000109	-2.24071	0.008278	0.160321
ENSMUSG00000070354	Evi2	2.086641	3.01E-12	3.25E-10	-1.13901	0.008386	0.161595
ENSMUSG00000026837	Col5a1	1.122108	4.27E-31	4.51E-28	0.203787	0.009439	0.173927
ENSMUSG00000107653	Gm31520	-1.42939	6.63E-08	3.49E-06	0.678783	0.00974	0.176523
ENSMUSG00000024411	Aqp4	-1.31818	0.0002	0.003737	-1.06905	0.010037	0.180509
ENSMUSG00000026955	Sapcd2	1.15426	0.004845	0.049982	0.772845	0.01038	0.183558
ENSMUSG00000022548	Apod	1.612113	1.64E-20	5.34E-18	0.373209	0.011067	0.190502
ENSMUSG00000034825	Nrip3	1.643977	9.16E-05	0.001945	0.77474	0.011267	0.192058
ENSMUSG00000028876	Epha10	5.075132	0.000862	0.012879	-2.89791	0.01212	0.200522

ENSMUSG00000026904	Slc4a10	3.433963	0.003142	0.035836	-2.78539	0.012666	0.204532
ENSMUSG00000089812	Gm15867	2.830229	2.74E-05	0.000699	0.868943	0.013331	0.211415
ENSMUSG00000035274	Tpbg	1.90478	0.000754	0.011605	0.779446	0.01353	0.212834
ENSMUSG00000093910	Zfp853	1.689338	0.002769	0.032284	0.940908	0.013687	0.214399
ENSMUSG00000014453	Blk	1.052502	3.76E-06	0.000127	0.490132	0.014055	0.217483
ENSMUSG00000094910	D430019H16Rik	1.271447	6.60E-06	0.000207	0.56763	0.014398	0.220382
ENSMUSG00000074824	Rslcan18	-1.08745	0.00042	0.007109	0.651592	0.014778	0.223584
ENSMUSG00000022449	Adamts20	1.457595	0.000191	0.003598	0.721056	0.015257	0.228857
ENSMUSG00000022440	C1qtnf6	1.318684	6.96E-27	3.83E-24	0.245332	0.015518	0.230431
ENSMUSG00000032269	Htr3a	3.003561	0.000228	0.004194	-1.37248	0.015662	0.231561
ENSMUSG00000000567	Sox9	1.247665	2.29E-07	1.09E-05	0.57284	0.015809	0.233306
ENSMUSG00000097418	Mir155hg	-1.91174	0.000131	0.002625	1.265819	0.016032	0.23496
ENSMUSG00000064294	Aox3	-1.04195	0.00145	0.019394	-1.37421	0.016643	0.239632
ENSMUSG00000106783	Chaer1	-1.25801	1.69E-05	0.000462	-0.82429	0.016793	0.24018
ENSMUSG00000041798	Gck	1.451428	7.62E-15	1.26E-12	-0.26299	0.017008	0.242167
ENSMUSG00000030867	Plk1	1.134664	0.000798	0.012113	0.667801	0.017098	0.242775
ENSMUSG00000031262	Cenpi	1.513105	7.72E-05	0.001674	0.711933	0.017175	0.243597
ENSMUSG00000069855	Slc47a2	-2.9767	0.001513	0.020041	2.76764	0.017591	0.245809
ENSMUSG00000109510	Gm42417	2.389432	1.15E-26	5.93E-24	0.313704	0.017654	0.246418
ENSMUSG00000036330	Slc18a1	1.249338	0.00209	0.02589	0.818385	0.019693	0.260526
ENSMUSG00000110673	Gm45833	5.02112	0.000302	0.005334	-2.31904	0.020634	0.266407
ENSMUSG00000020467	Efemp1	-1.46154	3.74E-16	7.52E-14	-0.49215	0.021215	0.270519
ENSMUSG00000024989	Cep55	1.34354	1.27E-05	0.00036	0.629708	0.021759	0.272962
ENSMUSG00000074973	Gm11382	3.492369	7.52E-08	3.91E-06	0.838992	0.022591	0.280202
ENSMUSG00000056494	Cngb3	-1.30659	7.10E-07	2.97E-05	-0.58892	0.022902	0.282004
ENSMUSG00000024990	Rbp4	2.261812	0.000296	0.005245	-1.32419	0.023242	0.283317
ENSMUSG00000100141	Gm6445	-2.00053	2.58E-06	9.24E-05	-1.5942	0.023702	0.286048
ENSMUSG00000108815	Gm49388	7.441569	6.26E-08	3.30E-06	-2.78615	0.024032	0.288245
ENSMUSG00000043122	A530016L24Rik	-1.01966	5.94E-16	1.16E-13	-0.3031	0.024801	0.292878
ENSMUSG00000110676	Gm45897	1.823329	0.002108	0.025994	0.854321	0.027066	0.310063
ENSMUSG00000115767	Gm10366	1.115748	0.00298	0.034293	-0.82325	0.027237	0.310894
ENSMUSG00000049420	Tmem200a	2.861841	9.41E-09	5.96E-07	0.592404	0.027388	0.311378
ENSMUSG00000029843	Slc13a4	1.606917	0.004222	0.04489	0.691525	0.028004	0.315465
ENSMUSG00000029361	Nos1	1.460176	4.26E-05	0.001012	-0.67455	0.028263	0.316259
ENSMUSG00000078137	Ankrd63	-1.61892	1.48E-06	5.72E-05	-0.73567	0.029284	0.320918
ENSMUSG00000109061	Gm49320	-3.55095	3.87E-07	1.73E-05	2.187272	0.029977	0.324989
ENSMUSG00000030862	Cpxm2	1.200564	7.19E-13	8.79E-11	0.270606	0.03138	0.331109
ENSMUSG00000038233	Gask1a	1.60184	5.82E-09	3.88E-07	0.442976	0.03143	0.331338
ENSMUSG00000042804	Gpr153	1.044766	3.41E-16	6.92E-14	0.258102	0.032092	0.334985
ENSMUSG00000110626	Gm45805	-1.97658	4.16E-07	1.85E-05	-1.10515	0.032482	0.336857
ENSMUSG00000106641	Gm43534	-1.17695	2.70E-05	0.00069	-0.74395	0.034538	0.348964
ENSMUSG00000043003	Rasef	-1.3368	1.50E-05	0.000415	-0.94533	0.034773	0.349961
ENSMUSG00000025658	Cnksr2	1.047486	0.003549	0.039369	-0.65654	0.036049	0.356623
ENSMUSG00000075324	Fign	-1.42455	2.18E-09	1.53E-07	0.559423	0.036538	0.358079
ENSMUSG00000036040	Adamtsl2	2.830163	1.79E-45	7.57E-42	0.336967	0.036603	0.358207

ENSMUSG00000029675	Eln	1.134533	5.37E-17	1.18E-14	0.296548	0.037349	0.36222
ENSMUSG00000010830	Kdelr3	1.084764	4.48E-08	2.44E-06	0.343102	0.038173	0.364995
ENSMUSG00000042388	Dlgap3	1.854422	0.00081	0.012258	-0.96961	0.038383	0.365316
ENSMUSG00000112376	Gm40649	-1.00684	0.004526	0.047307	0.699142	0.038636	0.365996
ENSMUSG00000040428	Plekha4	1.074953	5.56E-08	2.96E-06	0.342093	0.039522	0.370095
ENSMUSG00000039323	Igfbp2	3.942069	2.99E-05	0.000752	-1.27776	0.039546	0.370095
ENSMUSG00000030376	Slc8a2	1.010196	1.64E-05	0.000447	-0.45129	0.040246	0.37292
ENSMUSG00000022860	Chodl	3.752761	9.29E-06	0.000277	1.056664	0.040358	0.373041
ENSMUSG00000035365	Parpbp	1.28528	0.00029	0.005156	0.679683	0.044843	0.395414
ENSMUSG00000044006	Cilp2	1.554165	0.000416	0.007054	0.844055	0.045034	0.396203
ENSMUSG00000109341	Gm30873	2.463945	5.04E-08	2.72E-06	0.63127	0.045407	0.397778
ENSMUSG00000034675	Dbn1	1.227232	3.28E-20	1.00E-17	0.203782	0.046502	0.401166
ENSMUSG00000025127	Gcgr	1.407324	0.000503	0.008256	0.65091	0.046982	0.402587
ENSMUSG00000102692	Dchs2	1.378042	0.000114	0.002331	0.528069	0.047022	0.402787
ENSMUSG00000105466	Gm42998	4.72128	0.000901	0.013302	-2.03692	0.047072	0.402828
ENSMUSG00000114005	Gm24474	-1.38481	4.39E-07	1.93E-05	-0.5926	0.048006	0.40719
ENSMUSG00000009376	Met	1.222618	9.35E-06	0.000278	0.4589	0.048635	0.410062
ENSMUSG00000030236	Slco1b2	3.673679	0.001392	0.018813	-1.89113	0.048841	0.411052
ENSMUSG00000056031	9330154J02Rik	4.816888	0.000688	0.010798	-2.10184	0.049618	0.413632
ENSMUSG00000004473	Clec11a	1.479014	1.18E-10	1.02E-08	0.38458	0.050766	0.420049
ENSMUSG00000064080	Fbln2	1.015563	2.46E-27	1.52E-24	0.161951	0.051454	0.422673
ENSMUSG00000037996	Slc24a2	1.373758	2.41E-08	1.40E-06	0.36881	0.051669	0.423282
ENSMUSG00000043415	Otud1	1.214385	5.84E-06	0.000186	0.50315	0.052513	0.426207
ENSMUSG00000052336	Cx3cr1	1.127336	0.003787	0.041269	0.554426	0.053149	0.429339
ENSMUSG00000098318	Lockd	1.49607	0.000112	0.002307	0.608299	0.053578	0.431413
ENSMUSG00000041324	Inhba	1.146514	1.64E-07	8.04E-06	0.409762	0.054088	0.433061
ENSMUSG00000025089	Gfra1	-1.1036	1.96E-11	1.89E-09	-0.38601	0.055562	0.438664
ENSMUSG00000055775	Myh8	4.821673	7.29E-20	2.07E-17	0.527793	0.055925	0.439492
ENSMUSG00000008658	Rbfox1	-1.05819	4.00E-17	9.05E-15	-0.25387	0.056146	0.440171
ENSMUSG00000037653	Kctd8	1.2245	0.000199	0.00372	-0.55701	0.056699	0.442029
ENSMUSG00000030607	Acan	3.406208	2.22E-07	1.06E-05	0.760405	0.057384	0.445165
ENSMUSG00000111765	Gm10635	-2.26369	2.98E-13	3.78E-11	-0.61167	0.058622	0.449197
ENSMUSG00000033633	Clec18a	-1.8013	5.97E-09	3.95E-07	-0.74289	0.059409	0.452155
ENSMUSG00000022893	Adamts1	1.046175	4.21E-17	9.35E-15	0.215171	0.060061	0.453681
ENSMUSG00000025723	Nmb	-1.18068	2.29E-07	1.09E-05	-0.58984	0.060061	0.453681
ENSMUSG00000027894	Slc6a17	-1.2421	2.93E-10	2.35E-08	-0.40964	0.060763	0.456078
ENSMUSG00000020123	Avpr1a	-1.27667	0.000109	0.002249	-0.67883	0.060879	0.45661
ENSMUSG00000046733	Gprc5a	1.071318	0.000277	0.004961	0.473775	0.061318	0.457511
ENSMUSG00000027524	Edn3	2.226056	1.68E-23	6.86E-21	-0.28625	0.0618	0.45866
ENSMUSG00000024511	Rab27b	1.062838	4.45E-08	2.44E-06	0.325338	0.062482	0.460504
ENSMUSG00000056271	Lman1l	3.322571	2.85E-32	3.44E-29	0.29652	0.063406	0.462769
ENSMUSG00000085591	Gm13479	-1.23407	5.28E-08	2.83E-06	-0.46747	0.064468	0.466215
ENSMUSG00000044689	Gm13749	3.348351	0.00014	0.002775	1.054284	0.065634	0.469228
ENSMUSG00000116879	Gm49794	-2.18683	3.47E-05	0.00085	-1.53266	0.065675	0.46939
ENSMUSG00000027022	Xirp2	1.846741	2.52E-35	3.99E-32	0.279002	0.070957	0.484502

ENSMUSG00000015829	Tnr	1.279383	0.002957	0.034078	-0.6208	0.07201	0.487215
ENSMUSG00000059408	Mrgprh	-1.12634	9.86E-06	0.00029	-0.49265	0.072042	0.487308
ENSMUSG00000040276	Pacsin1	1.520454	1.55E-09	1.12E-07	0.393279	0.074323	0.496108
ENSMUSG00000064065	Ipcef1	1.571607	0.000643	0.010189	-0.69838	0.074525	0.496724
ENSMUSG00000062593	Gm49339	1.47576	0.000729	0.011342	-0.81368	0.075474	0.499136
ENSMUSG00000075015	Gm10801	-3.3479	0.001554	0.020418	1.783693	0.076241	0.501216
ENSMUSG00000051111	Sv2c	-1.62706	4.21E-06	0.000141	-0.89774	0.076353	0.501248
ENSMUSG00000037447	Arid5a	1.251177	1.23E-11	1.23E-09	0.281877	0.077161	0.503788
ENSMUSG00000020256	Aldh1l2	-1.27064	1.16E-08	7.16E-07	-0.41163	0.077164	0.503788
ENSMUSG00000086283	2810433D01Rik	2.66065	0.000829	0.012491	0.879034	0.080653	0.514172
ENSMUSG00000025330	Padi4	1.483521	0.00016	0.003092	0.500325	0.083392	0.522479
ENSMUSG00000005089	Slc1a2	1.643469	0.001683	0.021784	-0.87597	0.085283	0.527864
ENSMUSG00000026834	Acvr1c	3.013284	0.000674	0.010614	-1.1033	0.085419	0.528319
ENSMUSG00000032507	Fbxl2	1.020928	0.00079	0.012047	0.467068	0.085841	0.529774
ENSMUSG00000034533	Scn10a	-1.28083	1.14E-06	4.53E-05	-0.52838	0.089098	0.539482
ENSMUSG00000022512	Cldn1	2.320265	0.003782	0.041232	-1.2719	0.090188	0.542551
ENSMUSG00000020374	Rasgef1c	-1.39408	0.001097	0.015697	-1.17288	0.090345	0.542731
ENSMUSG00000048065	Cyb5r2	-1.11859	7.84E-13	9.46E-11	0.269966	0.090437	0.543028
ENSMUSG00000104263	9430062P05Rik	-1.29293	2.03E-06	7.52E-05	-0.38489	0.09115	0.545126
ENSMUSG00000107688	Gm44091	-3.34152	0.003734	0.040813	2.413673	0.091172	0.545131
ENSMUSG00000030641	Ddias	1.374569	0.000164	0.003165	0.504808	0.091671	0.546809
ENSMUSG00000021678	F2rl1	1.402383	5.00E-08	2.70E-06	0.361035	0.092112	0.548048
ENSMUSG00000039476	Prrx2	2.479381	3.37E-08	1.91E-06	0.538513	0.092193	0.548148
ENSMUSG00000020241	Col6a2	1.153973	6.29E-29	4.98E-26	0.146576	0.093732	0.552789
ENSMUSG00000022951	Rcan1	1.164821	2.83E-07	1.31E-05	0.364412	0.094053	0.553409
ENSMUSG00000097697	4833412C05Rik	3.338964	6.97E-06	0.000217	0.751064	0.094357	0.553696
ENSMUSG00000047420	Fam180a	2.259545	5.50E-06	0.000177	0.595451	0.094735	0.554479
ENSMUSG00000020627	Klhl29	1.399843	2.10E-09	1.48E-07	0.297013	0.100392	0.569668
ENSMUSG00000111318	Gm48483	-1.09103	0.000743	0.011483	-0.52758	0.100472	0.569712
ENSMUSG00000115783	Bc1	1.267076	7.41E-12	7.57E-10	0.282468	0.100962	0.570363
ENSMUSG00000032942	Ucp3	-1.15415	0.000152	0.002962	-0.416	0.102462	0.574617
ENSMUSG00000022483	Col2a1	3.594686	0.002104	0.025983	-1.47236	0.102375	0.574617
ENSMUSG00000040724	Kcna2	-1.01001	0.000297	0.00526	-0.52417	0.105834	0.581641
ENSMUSG00000061100	Retnla	-1.72791	7.84E-09	5.04E-07	-0.81077	0.108764	0.587529
ENSMUSG00000058019	Ces5a	2.214382	1.54E-06	5.94E-05	0.604192	0.110796	0.591863
ENSMUSG00000116903	Gm19522	-1.17902	2.48E-09	1.73E-07	-0.35696	0.113514	0.596726
ENSMUSG00000058743	Kcnj14	1.890322	3.93E-13	4.90E-11	0.339049	0.113561	0.596754
ENSMUSG00000041889	Shisa4	1.203885	2.19E-17	5.13E-15	0.186956	0.114105	0.597925
ENSMUSG00000044770	Scml4	2.066178	6.32E-22	2.35E-19	0.272997	0.116918	0.604161
ENSMUSG00000037990	Sh3rf3	1.294926	8.90E-08	4.56E-06	0.285561	0.123376	0.618358
ENSMUSG00000014602	Kif1a	1.157628	0.000174	0.003314	0.395572	0.129787	0.631393
ENSMUSG00000074398	Gm15441	-1.22907	0.000151	0.002952	0.556963	0.131108	0.634964
ENSMUSG00000031748	Gnao1	1.357892	4.25E-44	1.39E-40	-0.13583	0.134161	0.641281
ENSMUSG00000020000	Moxd1	-1.41725	1.46E-05	0.000407	0.554592	0.134592	0.641728
ENSMUSG00000085643	Gm12519	-1.13637	2.04E-05	0.000547	-0.36274	0.135158	0.642242

ENSMUSG00000025172	Ankrd2	2.900809	4.86E-15	8.31E-13	-0.47405	0.136486	0.645499
ENSMUSG00000068699	Flnc	1.023373	3.01E-15	5.37E-13	-0.18474	0.136997	0.646478
ENSMUSG00000117048	Gm41584	3.720316	0.000224	0.00414	-0.98579	0.138288	0.649257
ENSMUSG00000021228	Acot3	-1.4452	0.00041	0.006976	-1.03699	0.138491	0.649735
ENSMUSG00000035818	Plekhs1	-3.74373	0.003674	0.040306	1.862758	0.141756	0.656625
ENSMUSG00000014704	Hoxa2	1.401762	0.004145	0.044257	-0.66295	0.142239	0.657583
ENSMUSG00000017724	Etv4	1.64105	1.71E-08	1.02E-06	0.33307	0.144682	0.6593
ENSMUSG00000072849	Serpina1e	1.893545	0.001578	0.02068	-0.80016	0.143906	0.6593
ENSMUSG00000049719	Prss46	6.541456	4.75E-07	2.06E-05	-0.96082	0.151939	0.670347
ENSMUSG00000052188	Gm14964	-1.82957	6.39E-12	6.57E-10	0.383278	0.154938	0.674375
ENSMUSG00000061878	Sphk1	1.268596	4.43E-09	2.98E-07	0.271178	0.155749	0.675392
ENSMUSG00000041372	B4galnt3	3.614981	2.87E-05	0.000727	0.674836	0.156072	0.675927
ENSMUSG00000026259	Ngef	2.49916	2.39E-10	1.95E-08	0.355705	0.156504	0.676303
ENSMUSG00000025475	Adgra1	3.027638	3.62E-05	0.000881	0.588295	0.158255	0.677975
ENSMUSG00000074635	3110070M22Rik	1.491046	0.000122	0.002473	-0.53935	0.161391	0.683163
ENSMUSG00000101634	1700066B17Rik	-1.49553	0.000463	0.007699	0.635493	0.165271	0.689669
ENSMUSG00000022887	Masp1	1.168202	5.24E-27	2.95E-24	-0.14054	0.166871	0.692055
ENSMUSG00000026580	Selp	1.909288	2.30E-05	0.000598	0.47575	0.173008	0.699963
ENSMUSG00000090125	Pou3f1	1.881867	2.14E-05	0.000569	-0.54659	0.173467	0.700277
ENSMUSG00000047798	Cd300lf	2.600532	4.33E-05	0.001025	-0.60876	0.175257	0.703041
ENSMUSG00000023092	Fhl1	1.268055	3.28E-17	7.49E-15	0.239212	0.17558	0.703413
ENSMUSG00000116180	Gm49492	-1.06971	1.42E-07	7.07E-06	-0.27834	0.176069	0.70342
ENSMUSG00000117919	Gm41717	-1.05219	8.55E-07	3.49E-05	-0.33293	0.175877	0.70342
ENSMUSG00000013523	Bcas1	1.609114	0.002929	0.033808	-0.63915	0.176662	0.704687
ENSMUSG00000087107	AI662270	1.0253	0.000889	0.013174	0.314714	0.179851	0.70843
ENSMUSG00000022442	Ttll1	-1.14087	9.15E-15	1.49E-12	-0.18067	0.180322	0.709356
ENSMUSG00000048126	Col6a3	1.069424	3.23E-28	2.34E-25	-0.13418	0.18151	0.711411
ENSMUSG00000028909	Ptpru	-1.02783	2.12E-07	1.02E-05	-0.28979	0.18546	0.71516
ENSMUSG00000042115	Klhdc8a	1.015901	3.82E-16	7.62E-14	0.142606	0.189377	0.719069
ENSMUSG00000029838	Ptn	1.036048	4.30E-06	0.000144	0.299238	0.194823	0.726988
ENSMUSG00000037185	Krt80	1.113275	7.43E-06	0.000229	0.226182	0.195227	0.727637
ENSMUSG00000053192	Mllt11	1.141863	3.58E-20	1.08E-17	0.150179	0.197062	0.73036
ENSMUSG00000020216	Jsrp1	1.327462	3.49E-06	0.000119	-0.36274	0.199225	0.732632
ENSMUSG00000030553	Pgpep1l	1.300843	0.003163	0.036025	0.421755	0.20121	0.735272
ENSMUSG00000054409	Tmem74	4.50723	0.003483	0.038761	-1.42937	0.202678	0.736779
ENSMUSG00000043448	Gjc2	1.018057	0.000191	0.003598	0.304699	0.205051	0.741073
ENSMUSG00000117030	Gm49959	2.753861	0.003978	0.042866	-0.99166	0.206128	0.742652
ENSMUSG00000023279	Bmp15	5.311126	4.05E-05	0.000967	-1.01563	0.207083	0.74353
ENSMUSG00000039115	Itga9	1.06324	6.73E-23	2.66E-20	0.116886	0.207335	0.743743
ENSMUSG00000005360	Slc1a3	1.683854	2.96E-13	3.77E-11	0.242592	0.207831	0.74416
ENSMUSG00000027966	Col11a1	2.399454	0.001128	0.016067	0.577891	0.209404	0.746757
ENSMUSG00000109419	Gm45163	-1.314	6.53E-07	2.75E-05	0.330684	0.210659	0.748295
ENSMUSG00000021732	Fgf10	-1.91064	2.13E-08	1.25E-06	-0.63895	0.212842	0.752837
ENSMUSG00000104528	Gm43314	-1.68259	0.00265	0.031123	-0.96558	0.213674	0.754715
ENSMUSG00000045087	S1pr5	-1.07087	0.000582	0.009357	-0.38935	0.219348	0.763793

ENSMUSG00000040536	Necab1	1.124292	0.000465	0.007734	0.373988	0.220165	0.76491
ENSMUSG00000061816	Myl1	1.006781	1.69E-07	8.30E-06	0.226783	0.225829	0.771564
ENSMUSG00000085594	Gm11551	2.989624	0.00226	0.02756	0.755436	0.227002	0.773383
ENSMUSG00000021196	Pfkp	1.216075	6.51E-15	1.09E-12	0.181388	0.229354	0.776588
ENSMUSG00000085218	BB218582	-1.01916	1.49E-08	9.07E-07	-0.21364	0.23344	0.780806
ENSMUSG00000116604	Gm49745	-1.00659	0.003168	0.036063	-0.47365	0.236897	0.784251
ENSMUSG00000020411	Nipal4	4.70864	0.00077	0.011818	-1.20874	0.238662	0.78683
ENSMUSG00000118572	Gm53023	1.092532	0.00084	0.012603	-0.33844	0.238933	0.78702
ENSMUSG00000027560	Dok5	1.228886	0.000139	0.002755	0.343518	0.244543	0.79269
ENSMUSG00000116851	Gm35576	-1.31478	2.15E-05	0.00057	-0.43216	0.247101	0.794151
ENSMUSG00000004415	Col26a1	2.11304	1.06E-06	4.22E-05	-0.41136	0.249986	0.796959
ENSMUSG00000034813	Grip1	1.331541	0.000149	0.002916	0.591715	0.249937	0.796959
ENSMUSG00000079355	Ackr4	1.18706	1.53E-08	9.29E-07	0.139814	0.252238	0.798492
ENSMUSG00000012705	Retn	5.240862	0.000126	0.002546	-1.05837	0.253716	0.799556
ENSMUSG00000085055	Gm15958	-2.20096	0.000168	0.003231	-1.15682	0.256362	0.801248
ENSMUSG00000107768	Gm36582	3.46157	4.06E-05	0.000968	0.581698	0.258948	0.803539
ENSMUSG00000109119	Gm44752	3.109956	0.000911	0.013399	-0.81575	0.259492	0.803852
ENSMUSG00000053093	Myh7	1.168741	1.67E-11	1.62E-09	0.217574	0.260098	0.804557
ENSMUSG00000061451	Tmem151a	1.100371	0.000834	0.012547	-0.34267	0.260136	0.804576
ENSMUSG00000048620	Olfr1336	-3.34012	8.61E-06	0.00026	0.959423	0.262639	0.807058
ENSMUSG00000039376	Synpo2l	1.399609	2.15E-17	5.09E-15	0.198946	0.264019	0.807671
ENSMUSG00000048521	Cxcr6	1.426761	0.000403	0.006857	0.407745	0.265313	0.809205
ENSMUSG00000026558	Uck2	1.402337	1.36E-13	1.88E-11	0.204335	0.267036	0.809834
ENSMUSG00000006462	A530013C23Rik	-1.2948	1.06E-06	4.23E-05	0.273733	0.268352	0.812048
ENSMUSG00000110547	Gm29773	3.235353	2.66E-11	2.52E-09	-0.35992	0.268901	0.812594
ENSMUSG00000046245	Pilra	1.131237	0.000278	0.004968	0.333474	0.271109	0.815223
ENSMUSG00000003477	Inmt	-1.65751	3.61E-28	2.54E-25	-0.29876	0.273505	0.81728
ENSMUSG00000113909	Gm36377	4.038042	6.97E-05	0.001535	0.595628	0.276362	0.819289
ENSMUSG00000073608	Gal3st2c	-1.20879	1.62E-10	1.36E-08	-0.21968	0.276614	0.819556
ENSMUSG00000074802	Gas2l3	1.216297	4.85E-05	0.001133	0.30144	0.27822	0.820426
ENSMUSG00000020785	Camkk1	1.203629	3.90E-08	2.18E-06	0.188924	0.282391	0.825001
ENSMUSG00000020325	Fstl3	1.749216	2.32E-32	2.94E-29	0.156861	0.289187	0.831003
ENSMUSG00000097157	Gm26512	3.713145	0.002285	0.027805	0.68981	0.2923	0.833769
ENSMUSG00000068101	Cenpm	1.340583	0.000481	0.007964	0.318375	0.296493	0.838607
ENSMUSG00000072902	Gm10435	-1.04751	1.69E-10	1.42E-08	-0.14422	0.299918	0.842778
ENSMUSG00000050195	Scd4	1.000512	0.001179	0.016607	-0.31052	0.306773	0.849303
ENSMUSG00000117492	Gm19052	-3.51844	0.000513	0.008385	1.602785	0.307529	0.849607
ENSMUSG00000022376	Adcy8	-1.22329	8.92E-05	0.001903	-0.31472	0.307787	0.849997
ENSMUSG00000074736	Syndig1	3.433549	1.17E-11	1.17E-09	-0.38834	0.310218	0.851729
ENSMUSG00000030762	Aqp8	2.454537	2.75E-20	8.49E-18	0.237307	0.315469	0.856281
ENSMUSG00000078640	Gm11627	1.12351	0.001891	0.023849	0.311991	0.315458	0.856281
ENSMUSG00000045102	Poln	-1.26717	1.03E-05	0.000301	-0.26189	0.324089	0.863394
ENSMUSG00000022580	Rhpn1	3.779591	3.32E-05	0.000814	-0.61607	0.325144	0.863908
ENSMUSG00000036242	Armh4	-1.27964	3.84E-29	3.36E-26	-0.11406	0.329415	0.866827
ENSMUSG00000086384	Banf2os	-1.38717	2.68E-06	9.52E-05	-0.33495	0.340159	0.876327

ENSMUSG00000073433	Arhgdig	1.541099	1.34E-06	5.20E-05	0.225514	0.343124	0.879221
ENSMUSG00000032691	Nlrp3	1.363436	0.002077	0.025784	0.290922	0.3469	0.882455
ENSMUSG00000114212	Gm47985	2.269851	4.60E-12	4.83E-10	0.271371	0.353971	0.888372
ENSMUSG00000021359	Tfap2a	-2.9886	0.000429	0.007248	1.255198	0.355311	0.889777
ENSMUSG00000118002	Gm4107	-2.49662	0.003101	0.035469	1.06423	0.357497	0.890767
ENSMUSG00000044172	Ptx4	3.117419	1.31E-05	0.00037	0.459903	0.36361	0.894466
ENSMUSG00000116029	Gm41414	-1.42272	0.001201	0.016852	-0.63119	0.364403	0.894604
ENSMUSG00000060509	Xcr1	1.226025	0.003699	0.040513	-0.26574	0.368114	0.898052
ENSMUSG00000110529	Gm45694	-1.13219	0.001789	0.022844	0.350023	0.372121	0.900413
ENSMUSG00000022797	Tfrc	1.809708	4.33E-26	2.11E-23	0.318776	0.376534	0.902495
ENSMUSG00000021208	Ifi27l2b	2.573182	0.000206	0.003854	0.409643	0.384004	0.907232
ENSMUSG00000112831	Gm35533	1.098137	9.34E-05	0.001979	-0.20734	0.384489	0.907604
ENSMUSG00000054890	Olfr1535	4.7108	0.000795	0.012099	-0.93081	0.38516	0.907939
ENSMUSG00000030124	Lag3	1.196689	0.001275	0.017644	0.236689	0.392032	0.911274
ENSMUSG00000073415	Gm10501	-1.78489	0.000104	0.002164	0.37965	0.395457	0.912877
ENSMUSG00000092522	Gm20389	-2.80657	0.002605	0.030719	1.2553	0.40935	0.919386
ENSMUSG00000027887	Sypl2	1.825199	2.98E-17	6.86E-15	-0.18031	0.409639	0.919676
ENSMUSG00000031503	Col4a2	1.044333	5.55E-24	2.34E-21	0.073031	0.411486	0.920604
ENSMUSG00000039714	Cplx3	2.249827	0.003565	0.039493	0.395689	0.411501	0.920604
ENSMUSG00000039639	Kcne1	1.023748	1.60E-05	0.000439	0.182141	0.415549	0.923801
ENSMUSG00000037129	Tmprss13	-1.03377	0.001289	0.017801	-0.31226	0.432665	0.933772
ENSMUSG00000112593	Gm48882	-1.19696	1.28E-11	1.27E-09	-0.11746	0.438405	0.936504
ENSMUSG00000044534	Ackr2	1.246076	4.03E-08	2.23E-06	0.14242	0.450857	0.941668
ENSMUSG00000031502	Col4a1	1.026171	2.48E-20	7.76E-18	0.069431	0.451651	0.942095
ENSMUSG00000043439	Epop	-1.70906	4.62E-08	2.51E-06	-0.28481	0.452543	0.94285
ENSMUSG00000097593	Gm26676	-1.16923	0.001386	0.018766	-0.31427	0.462412	0.947604
ENSMUSG00000027868	Tbx15	3.116229	7.25E-20	2.07E-17	0.196515	0.467586	0.94988
ENSMUSG00000050296	Abca12	-1.35631	2.43E-14	3.69E-12	-0.15932	0.467549	0.94988
ENSMUSG00000028415	Spink4	3.196039	0.000274	0.004915	-0.44277	0.468371	0.950269
ENSMUSG00000031170	Slc38a5	-1.86853	0.001148	0.016266	0.569067	0.471136	0.95125
ENSMUSG00000052613	Pcdh15	-1.25398	0.004665	0.048417	0.377189	0.473007	0.952068
ENSMUSG00000020032	Nuak1	1.230297	2.80E-19	7.80E-17	-0.0738	0.47771	0.953856
ENSMUSG00000036123	Slc9a3	1.778752	3.86E-13	4.87E-11	0.216989	0.477814	0.953856
ENSMUSG00000079428	Tceal7	1.165993	0.000863	0.012883	0.22258	0.481589	0.954536
ENSMUSG00000061723	Tnnt3	1.539443	0.000184	0.003492	-0.18892	0.485975	0.956669
ENSMUSG00000037949	Ano10	-1.04655	9.34E-27	5.04E-24	-0.0634	0.49665	0.960858
ENSMUSG00000114792	Gm6416	-1.04767	6.31E-06	0.000199	0.172995	0.497972	0.962029
ENSMUSG00000115388	Eppk1	1.706985	0.000108	0.002235	0.229878	0.500904	0.963395
ENSMUSG00000053963	Stum	-1.65135	2.57E-11	2.45E-09	-0.16644	0.513348	0.968118
ENSMUSG00000054871	Tmem158	1.066174	2.52E-07	1.19E-05	0.12058	0.516363	0.969358
ENSMUSG00000101972	H3c11	-1.40236	0.004105	0.04392	-0.44233	0.520567	0.971274
ENSMUSG00000032500	Dclk3	1.747099	0.000818	0.01235	0.252798	0.524025	0.972042
ENSMUSG00000110630	K230015D01Rik	1.013045	0.003686	0.040429	0.183443	0.525339	0.972942
ENSMUSG00000078451	Ppil6	-1.27351	0.000687	0.010793	0.237333	0.528495	0.974276
ENSMUSG00000091890	A830073O21Rik	-1.34079	3.20E-05	0.000791	-0.22628	0.53591	0.976831

ENSMUSG00000024366	Gfra3	-1.32809	0.001117	0.015929	-0.28507	0.536382	0.976831
ENSMUSG00000051435	Fhad1	2.99864	3.47E-07	1.58E-05	-0.15724	0.54311	0.979371
ENSMUSG00000043621	Ubxn10	-1.40187	2.39E-08	1.39E-06	-0.21704	0.55433	0.982552
ENSMUSG00000000386	Mx1	1.241718	7.57E-05	0.001649	0.155108	0.552681	0.982552
ENSMUSG00000030000	Add2	2.063642	0.001629	0.021225	0.318512	0.551597	0.982552
ENSMUSG00000081209	Gm14890	-1.31279	0.000455	0.0076	0.273685	0.559972	0.985274
ENSMUSG00000035459	Stab2	-1.07181	0.003612	0.039869	0.261143	0.559958	0.985274
ENSMUSG00000116641	Gm41505	2.029451	7.13E-11	6.40E-09	0.132361	0.566664	0.988631
ENSMUSG00000058952	Cfi	2.611798	0.00172	0.022162	-0.37366	0.570836	0.990939
ENSMUSG00000087129	Gm16316	-1.60812	6.35E-06	0.0002	0.209584	0.572482	0.992378
ENSMUSG00000079455	Gm16026	5.798595	5.76E-06	0.000184	0.377019	0.574413	0.993054
ENSMUSG00000102098	2310016D03Rik	-2.7289	5.68E-10	4.46E-08	-0.53085	0.576744	0.993791
ENSMUSG00000036136	Fam110c	3.032457	0.001184	0.016639	-0.5633	0.577357	0.99422
ENSMUSG00000041423	Paqr6	1.199028	0.001411	0.019004	0.200451	0.579251	0.99511
ENSMUSG00000112950	Gm32369	-1.82165	2.35E-10	1.92E-08	-0.19488	0.579674	0.995347
ENSMUSG00000025537	Phkg1	-1.4568	4.76E-27	2.78E-24	-0.08435	0.583117	0.995672
ENSMUSG00000000204	Slfn4	1.445406	0.001096	0.01569	0.183642	0.583579	0.995974
ENSMUSG00000114582	3110040M04Rik	1.953326	3.48E-06	0.000119	0.181398	0.586483	0.996609
ENSMUSG00000070574	2310016G11Rik	-1.16906	7.83E-05	0.00169	-0.18007	0.586801	0.996609
ENSMUSG00000031972	Acta1	3.912054	3.41E-71	8.63E-67	-0.04616	0.755642	1
ENSMUSG00000112327	Gm36827	-1.3598	4.17E-29	3.52E-26	0.062928	0.633443	1
ENSMUSG00000026605	Cenpf	-1.74401	4.82E-27	2.78E-24	0.002725	0.988148	1
ENSMUSG00000020676	Ccl11	-2.92497	5.54E-23	2.23E-20	-0.15273	0.704922	1
ENSMUSG00000028841	Cnksr1	1.479698	1.73E-21	6.16E-19	-0.03972	0.800463	1
ENSMUSG00000023905	Tnfrsf12a	1.914931	9.89E-21	3.30E-18	0.028511	0.874205	1
ENSMUSG00000000183	Fgf6	2.060846	3.61E-20	1.08E-17	-0.02708	0.900956	1
ENSMUSG00000087651	1500009L16Rik	1.397618	1.49E-18	3.93E-16	-0.04846	0.70315	1
ENSMUSG00000026986	Hnmt	-1.27291	2.25E-18	5.88E-16	-0.01982	0.906275	1
ENSMUSG00000104348	Gm37691	-1.35438	5.33E-18	1.36E-15	-0.0415	0.818522	1
ENSMUSG00000025900	Rp1	-2.31614	1.61E-16	3.41E-14	0.120839	0.772143	1
ENSMUSG00000040247	Tbc1d10c	-1.87928	1.63E-16	3.42E-14	-0.11411	0.760619	1
ENSMUSG00000042895	Abra	1.435642	1.62E-15	2.99E-13	0.051316	0.711621	1
ENSMUSG00000024486	Hbegf	1.345311	1.64E-15	3.01E-13	0.015614	0.923037	1
ENSMUSG00000028364	Tnc	2.063037	2.25E-15	4.03E-13	0.080486	0.739408	1
ENSMUSG00000117916	9630028I04Rik	-1.5035	7.85E-15	1.28E-12	-0.07325	0.757611	1
ENSMUSG00000046997	Spsb4	1.634208	4.10E-14	5.97E-12	-0.08722	0.68378	1
ENSMUSG00000061533	Cep128	-1.176	1.01E-12	1.18E-10	0.004154	0.978045	1
ENSMUSG00000025140	Pycr1	1.766877	4.49E-12	4.74E-10	-0.06913	0.72094	1
ENSMUSG00000053930	Shisa6	-2.03658	1.18E-11	1.18E-09	-0.04219	0.966571	1
ENSMUSG00000116725	Gm29686	-1.22783	4.31E-11	4.00E-09	-0.01242	0.965495	1
ENSMUSG00000074219	Gm10644	-1.55989	1.89E-10	1.57E-08	0.018626	0.980817	1
ENSMUSG00000032105	Pdzd3	1.054694	2.64E-10	2.13E-08	0.024355	0.844098	1
ENSMUSG00000051606	2010001K21Rik	-2.41777	1.45E-09	1.06E-07	0.069985	0.910673	1
ENSMUSG00000041594	Tmtc4	-1.044	1.85E-09	1.33E-07	0.006289	0.969062	1
ENSMUSG00000032064	Dixdc1	-1.22173	3.55E-09	2.44E-07	0.037357	0.83246	1

ENSMUSG00000037143	Cfap61	-1.69323	3.14E-08	1.80E-06	-0.00797	1	1
ENSMUSG00000108207	1810059H22Rik	-1.15858	3.25E-08	1.85E-06	-0.04226	0.868508	1
ENSMUSG00000042851	Zc3h6	-1.08901	2.71E-07	1.26E-05	0.041378	0.832971	1
ENSMUSG00000056738	A730036I17Rik	2.567087	3.05E-07	1.40E-05	-0.03402	0.946826	1
ENSMUSG00000084819	Gm11967	1.128651	4.28E-07	1.89E-05	-0.01072	0.960098	1
ENSMUSG00000112800	Gm40604	-1.73055	4.45E-07	1.95E-05	-0.1019	0.85261	1
ENSMUSG00000110534	Gm45708	3.611288	5.79E-07	2.47E-05	-0.1913	0.613619	1
ENSMUSG00000114018	Gm36495	6.577119	7.96E-07	3.28E-05	0.328034	0.642933	1
ENSMUSG00000054545	Ugt1a6a	1.948911	1.01E-06	4.05E-05	0.105456	0.826337	1
ENSMUSG00000045620	Odf3l1	5.873072	1.29E-06	5.05E-05	-0.04441	1	1
ENSMUSG00000020142	Slc1a4	1.044133	2.29E-06	8.36E-05	0.057548	0.780784	1
ENSMUSG00000116121	Gm49486	-1.81501	2.48E-06	8.91E-05	0.167775	0.683331	1
ENSMUSG00000040364	Sec1	1.915106	4.38E-06	0.000146	0.169223	0.603196	1
ENSMUSG00000063681	Crb1	-1.58906	5.58E-06	0.000179	-0.11852	0.839108	1
ENSMUSG00000035498	Cdcp1	1.895634	9.93E-06	0.000292	0.011834	1	1
ENSMUSG00000024600	Slc27a6	2.129156	1.04E-05	0.000303	0.155904	0.702826	1
ENSMUSG00000021799	Opn4	-1.00964	1.07E-05	0.00031	-0.11211	0.637641	1
ENSMUSG00000020102	Slc16a7	-1.20364	1.22E-05	0.000348	0.102866	0.639301	1
ENSMUSG00000031284	Pak3	2.325464	1.22E-05	0.000349	-0.1257	0.756937	1
ENSMUSG00000083307	AA414768	1.331791	1.23E-05	0.000351	0.128373	0.619259	1
ENSMUSG00000033491	Prss35	5.444697	2.20E-05	0.000579	0.157136	0.832692	1
ENSMUSG00000046415	B430212C06Rik	-1.27243	2.27E-05	0.000594	-0.04114	0.929678	1
ENSMUSG00000028463	Car9	1.543835	3.09E-05	0.000769	0.117565	0.692117	1
ENSMUSG00000021536	Adcy2	1.898786	3.10E-05	0.00077	-0.01071	1	1
ENSMUSG00000117399	Gm19689	-1.80632	3.12E-05	0.000775	-0.27452	0.658319	1
ENSMUSG00000061959	Ces1e	-1.4266	3.97E-05	0.000949	-0.26906	0.5969	1
ENSMUSG00000073975	Olfr550	2.436202	5.55E-05	0.001276	0.123943	0.795625	1
ENSMUSG00000096960	A230028O05Rik	5.600125	5.92E-05	0.001339	-0.10846	0.927076	1
ENSMUSG00000037145	Lypd8l	-1.04225	7.21E-05	0.001579	0.016096	0.956471	1
ENSMUSG00000018930	Ccl4	5.248417	7.59E-05	0.001651	0.246988	0.886743	1
ENSMUSG00000101445	Gm28932	2.242391	8.38E-05	0.001798	-0.02293	1	1
ENSMUSG00000073759	4933407E24Rik	3.40266	0.0001	0.002104	0.077525	0.955236	1
ENSMUSG00000114159	Gm48866	-1.76553	0.000109	0.002253	0.180515	0.785537	1
ENSMUSG00000087604	Cdrt4os1	5.087993	0.000134	0.002671	-0.52445	0.64566	1
ENSMUSG00000043333	Rhbdl2	2.894756	0.000144	0.002846	-0.25976	0.744874	1
ENSMUSG00000033676	Gabrb3	2.207141	0.000167	0.003222	-0.12647	0.808805	1
ENSMUSG00000085682	Gm14267	-1.15813	0.000191	0.003598	-0.17123	0.622874	1
ENSMUSG00000054944	5330416C01Rik	5.077971	0.000224	0.004143	-0.2041	0.918566	1
ENSMUSG00000017204	Gsdma	1.848362	0.000256	0.00464	0.062126	0.885063	1
ENSMUSG00000028965	Tnfrsf9	3.051625	0.000293	0.005214	0.226651	0.749063	1
ENSMUSG00000022619	Mapk8ip2	1.677928	0.000299	0.005287	0.025193	0.965972	1
ENSMUSG00000058831	Opn1sw	3.042833	0.000358	0.006183	-0.17685	0.85143	1
ENSMUSG00000111963	Gm32283	-2.46103	0.000432	0.007293	0.167816	1	1
ENSMUSG00000001349	Cnn1	1.179939	0.00044	0.007403	0.096855	0.718625	1
ENSMUSG00000106515	Gm30382	-1.28065	0.000455	0.0076	-0.03091	1	1

ENSMUSG00000042379	Esm1	1.040191	0.000484	0.008017	-0.06522	0.751632	1
ENSMUSG00000092371	Gm20511	3.161916	0.000564	0.009099	-0.18715	0.798166	1
ENSMUSG00000118024	Gm50419	-1.3753	0.000578	0.009309	0.040166	1	1
ENSMUSG00000089827	1700023H06Rik	4.707766	0.000761	0.011698	-0.2617	0.871393	1
ENSMUSG00000000308	Ckmt1	1.546469	0.000778	0.01191	-0.00295	1	1
ENSMUSG00000110618	Gm39822	1.172114	0.00079	0.012047	-0.10775	0.732573	1
ENSMUSG00000030048	Gkn3	-1.52706	0.000802	0.01217	0.175027	0.801174	1
ENSMUSG00000106547	B230303O12Rik	-1.27047	0.000816	0.012328	-0.04481	0.928544	1
ENSMUSG00000118202	C030017B01Rik	2.114464	0.000833	0.012534	-0.10038	0.896094	1
ENSMUSG00000105699	Gm43703	3.194041	0.000867	0.012938	-0.10893	0.930666	1
ENSMUSG00000078722	Gm12394	-2.02319	0.000895	0.013235	0.228223	1	1
ENSMUSG00000105211	Mir3544	1.896164	0.000897	0.013257	0.03326	1	1
ENSMUSG00000118387	Gm50415	-1.75818	0.001167	0.016478	-0.30108	0.780209	1
ENSMUSG00000106140	Gm42208	-1.02254	0.001473	0.019639	-0.08339	0.847126	1
ENSMUSG00000101028	Gm28723	1.075337	0.001483	0.019727	-0.16525	0.600637	1
ENSMUSG00000042817	Flt3	2.153287	0.001605	0.020947	-0.13915	0.735546	1
ENSMUSG00000034687	Fras1	1.366214	0.001619	0.021109	0.138336	0.652771	1
ENSMUSG00000015619	Gata3	-1.66443	0.001766	0.022631	0.267039	0.665583	1
ENSMUSG00000096237		-1.83097	0.001768	0.022646	-0.43775	0.752634	1
ENSMUSG00000117926	C030004G16Rik	2.92995	0.001778	0.022733	0.106175	0.928006	1
ENSMUSG00000034959	Rubcnl	1.062726	0.00183	0.023284	0.041684	0.896328	1
ENSMUSG00000011589	Fsd1	2.654762	0.00185	0.023485	-0.28934	0.676418	1
ENSMUSG00000104342	Gm36401	2.121505	0.001944	0.024377	-0.00208	1	1
ENSMUSG00000110298	Gm8189	-1.05079	0.001953	0.024467	-0.03087	0.962085	1
ENSMUSG00000024107	Lhcgr	-2.29556	0.001959	0.024537	0.652718	0.694861	1
ENSMUSG00000114286	Gm47850	-1.24156	0.001984	0.024797	-0.21917	0.622346	1
ENSMUSG00000027612	Mmp24	1.907476	0.002468	0.029457	-0.09883	0.869559	1
ENSMUSG00000075014	Gm10800	-2.0762	0.002492	0.029662	0.035424	0.937387	1
ENSMUSG00000001348	Acp5	1.191892	0.00269	0.031522	-0.00923	1	1
ENSMUSG00000097146	Gm4211	-1.4422	0.002731	0.03191	0.021144	1	1
ENSMUSG00000109882	Gm18706	-1.58263	0.003063	0.035156	-0.31515	0.62969	1
ENSMUSG00000035352	Ccl12	1.082722	0.003381	0.037951	0.02596	0.957886	1
ENSMUSG00000040867	Begain	1.077267	0.003571	0.039545	-0.1476	0.640242	1
ENSMUSG00000054204	Alkal2	-1.90534	0.003582	0.039625	0.260786	0.754581	1
ENSMUSG00000027107	Chrna1	1.959666	0.003614	0.039874	0.095289	0.881451	1
ENSMUSG00000043727	F830045P16Rik	2.12016	0.003745	0.040915	0.274658	0.597556	1
ENSMUSG00000033576	Apol6	1.168618	0.003911	0.042321	-0.02273	0.97565	1
ENSMUSG00000022367	Has2	1.321007	0.004222	0.04489	-0.15334	0.710308	1
ENSMUSG00000091648	C2cd4d	-1.73476	0.004795	0.049547	-0.24657	0.808602	1
ENSMUSG00000097286	Gm26684	-5.92799	4.16E-07	1.85E-05	#N/A	#N/A	#N/A
ENSMUSG00000029282	Amtn	-6.04453	4.66E-07	2.03E-05	#N/A	#N/A	#N/A
ENSMUSG00000087192	Lexis1	-5.70633	2.62E-06	9.33E-05	#N/A	#N/A	#N/A
ENSMUSG00000085996	A830012C17Rik	-4.51463	2.87E-05	0.000727	#N/A	#N/A	#N/A
ENSMUSG00000020323	Prss57	-5.36072	5.61E-05	0.001285	#N/A	#N/A	#N/A
ENSMUSG00000042451	Mybph	-5.37082	6.90E-05	0.001524	#N/A	#N/A	#N/A

ENSMUSG00000117670	Gm50270	-5.00486	0.000265	0.004774	#N/A	#N/A	#N/A
ENSMUSG00000104336	Gm34240	-3.43105	0.000285	0.005088	#N/A	#N/A	#N/A
ENSMUSG00000028386	Slc46a2	-3.5462	0.000377	0.006468	#N/A	#N/A	#N/A
ENSMUSG00000108696	Gm5738	-3.97272	0.001153	0.016315	#N/A	#N/A	#N/A
ENSMUSG00000111840	Gm48832	-2.42368	0.001295	0.017846	#N/A	#N/A	#N/A
ENSMUSG00000054909	Wbscr25	-2.82916	0.001642	0.021365	#N/A	#N/A	#N/A
ENSMUSG00000111151	Gm5120	-4.5697	0.0024	0.028843	#N/A	#N/A	#N/A
ENSMUSG00000083038	Gm13233	-4.59769	0.002549	0.030248	#N/A	#N/A	#N/A
ENSMUSG00000052951	C130021I20Rik	-3.07666	0.002768	0.032281	#N/A	#N/A	#N/A
ENSMUSG00000100389	Rbm6-ps2	-2.95813	0.002806	0.032593	#N/A	#N/A	#N/A
ENSMUSG00000103657	Gm37204	-3.47567	0.003277	0.036996	#N/A	#N/A	#N/A
ENSMUSG00000087476	Rap1gapos	-4.43227	0.003482	0.038761	#N/A	#N/A	#N/A
ENSMUSG00000042268	Slc26a9	-2.81426	0.003995	0.042961	#N/A	#N/A	#N/A
ENSMUSG00000097762	4732463B04Rik	-3.09426	0.00467	0.048447	#N/A	#N/A	#N/A

Supplementary Table 4 Echocardiography examination of mice injected by AAV9-cTnT-Cardinal and their control littermates with transverse aortic constriction (TAC) or sham operation at different time points after surgery.

	AAV9-GFP;Sham		AAV9-Cardinal;Sham		AAV9-GFP;TAC		AAV9-Cardinal;TAC	
	2 wks (N=6)	4 wks (N=4)	2 wks (N=5)	4 wks (N=5)	2 wks(N=10)	4 wks(N=10)	2 wks(N=10)	4 wks (N=7)
**IVS;d (mm)**	1.166±0.152	1.085±0.125	1.034±0.06	1.049±0.073	1.371±0.144*	1.629±0.143**	1.398±0.121	1.439±0.141#
**IVS;s (mm)**	1.752±0.121	1.736±0.195	1.686±0.131	1.638±0.156	1.902±0.222	2.12±0.184*	1.982±0.154	2.058±0.115
**LVID;d (mm)**	3.641±0.507	3.515±0.247	3.735±0.168	3.779±0.288	3.817±0.715	3.672±0.778	3.36±0.349	3.32±0.174
**LVID;s (mm)**	2.035±0.617	1.898±0.219	2.175±0.153	2.128±0.232	2.591±0.917	2.469±0.885	1.878±0.355#	1.878±0.193
**LVPW;d (mm)**	0.938±0.125	1.003±0.095	0.965±0.221	1.038±0.092	1.166±0.201*	1.224±0.204	1.085±0.075	1.184±0.095
**LVPW;s (mm)**	1.458±0.198	1.538±0.12	1.433±0.284	1.53±0.111	1.591±0.348	1.664±0.326	1.645±0.178	1.712±0.136
**EF (%)**	76.147±10.454	78.06±4.92	73.39±3.81	75.7±2.86	61.72±16.26	63.25±13.21	76.14±7.46#	75.48±5.87#
**FS (%)**	45.158±10.029	45.98±4.92	41.75±3.19	43.81±2.48	33.75±11.37	34.26±8.74*	44.32±6.82#	43.43±5.12#
**LV Mass (mg)**	149.37±30.84	140.37±23	144.16±21.43	156.6±24.81	214.4±63.05*	239.9±45.18**	170.11±17.86	182.59±16.96#
**LV Mass (Corrected, mg)**	119.5±24.67	112.3±18.4	115.33±17.15	125.28±19.85	171.52±50.44*	191.92±36.14**	136.09±14.29	146.08±13.57#
**LV Vol;d (uL)**	57.32±18.63	51.69±8.84	59.58±6.45	61.59±10.74	65.66±30.36	60.68±34.73	46.79±12.12	44.94±5.72
**LV Vol;s (uL)**	15.15±10.56	11.35±3.45	15.86±2.81	15.14±3.82	29.13±25.61	26.07±26.34	11.49±5.77	11.02±2.87
**Heart Rate (BPM)**	695±44	703±50	701±107	671±55	714±28	710±33	723±37	722±43

*: $P_{\text{AAV9-GFP;Sham (same timepoint) vs. AAV9-GFP;TAC (same timepoint)}}$ <0.05; **: $P_{\text{AAV9-GFP;Sham (same timepoint) vs. AAV9-GFP;TAC (same timepoint)}}$ <0.01; #: $P_{\text{AAV9-GFP;TAC (same timepoint) vs. AAV9-Cardinal;TAC (same timepoint)}}$ <0.05; ##: $P_{\text{AAV9-GFP;TAC (same timepoint) vs. AAV9-Cardinal;TAC (same timepoint)}}$ <0.01

Supplementary Table 5 Results of mass spectrometry of Set 1 RNA pull-down experiment

Note: Data in each column represented numbers of total or unique peptides that matched to each protein

Proteins	Unique Cardinal	Total Cardinal	Unique Cardinal-as	Total Cardinal-as	Unique Lincp21	Total Lincp21	Unique Nppa-as	Total Nppa-as
sp|A2ASS6|TITIN_MOUSE	192	244	208	266	185	239	215	286
sp|Q91Z83|MYH7_MOUSE	163	1033	161	1133	143	1136	156	1387
sp|Q61879|MYH10_MOUSE	56	94	65	110	63	114	59	120
tr|E9Q616|E9Q616_MOUSE	53	70	35	51	41	57	36	53
sp|Q8VDD5|MYH9_MOUSE	49	73	53	86	46	86	55	100
sp|O70468|MYPC3_MOUSE	39	85	40	85	33	77	38	95
sp|Q02566|MYH6_MOUSE	36	118	35	133	35	123	31	155
sp|Q9JHU4|DYHC1_MOUSE	36	48	30	37	33	43	25	34
sp|O55143|AT2A2_MOUSE	35	77	30	60	30	57	28	64
sp|P16546|SPTN1_MOUSE	34	41	26	33	32	42	21	25
sp|P70670|NACAM_MOUSE	32	50	32	51	33	50	30	42
sp|P56480|ATPB_MOUSE	31	104	30	68	33	90	28	75
sp|E9Q4Z2|ACACB_MOUSE	29	45	28	37	28	37	24	32
sp|Q68FD5|CLH1_MOUSE	29	42	19	26	24	38	21	32
sp|Q03265|ATPA_MOUSE	26	93	26	77	23	82	25	79
sp|Q99KI0|ACON_MOUSE	26	65	25	46	28	60	26	45
sp|P11499|HS90B_MOUSE	25	62	22	46	24	46	25	57
sp|P63038|CH60_MOUSE	25	42	20	32	20	41	20	36
sp|Q62261|SPTB2_MOUSE	25	28	19	23	17	22	19	23
sp|Q6PB66|LPPRC_MOUSE	24	33	28	35	15	18	21	27
sp|Q99MR8|MCCA_MOUSE	24	42	22	46	21	47	22	54
sp|P52480|KPYM_MOUSE	24	55	21	50	23	48	21	52
sp|P14824|ANXA6_MOUSE	23	37	16	24	19	34	18	34
sp|Q9JI91|ACTN2_MOUSE	22	46	28	51	26	53	25	60
sp|Q8BMS1|ECHA_MOUSE	22	40	22	37	19	35	18	33
sp|Q9WUB3|PYGM_MOUSE	22	39	20	31	13	24	19	28
sp|P58252|EF2_MOUSE	22	44	20	30	23	44	18	31
sp|P50544|ACADV_MOUSE	22	34	15	19	12	24	18	30
sp|P09542|MYL3_MOUSE	21	117	21	121	20	117	19	122
sp|Q60597|ODO1_MOUSE	21	33	15	28	17	29	17	39
sp|Q8VHX6|FLNC_MOUSE	20	21	19	22	16	25	18	23
sp|P17710|HXK1_MOUSE	20	32	18	22	18	25	20	27
sp|Q8CI94|PYGB_MOUSE	20	38	16	26	15	24	16	28
sp|Q01853|TERA_MOUSE	20	35	16	25	15	26	16	25
sp|Q05920|PYC_MOUSE	19	34	25	37	26	46	18	34
sp|P54071|IDHP_MOUSE	19	49	19	32	16	40	17	39

sp|P09411|PGK1_MOUSE	19	42	17	40	18	45	18	42
sp|Q8VDN2|AT1A1_MOUSE	19	34	15	28	17	34	17	34
sp|P60710|ACTB_MOUSE	18	158	20	154	18	144	19	191
sp|P07310|KCRM_MOUSE	18	76	16	47	17	63	15	55
sp|P08113|ENPL_MOUSE	18	29	10	15	9	16	10	14
sp|P20029|GRP78_MOUSE	17	33	18	28	16	28	13	27
sp|P06151|LDHA_MOUSE	17	27	17	25	13	24	14	26
sp|P16858|G3P_MOUSE	17	63	16	48	9	42	11	50
sp|Q91VD9|NDUS1_MOUSE	17	23	16	24	15	26	18	28
tr|B7ZN52|B7ZN52_MOUSE	17	21	16	22	20	24	18	22
sp|P27773|PDIA3_MOUSE	17	31	14	20	11	24	9	19
sp|P06745|G6PI_MOUSE	17	30	14	21	14	25	12	16
sp|P09103|PDIA1_MOUSE	17	27	14	24	14	21	16	25
sp|P05202|AATM_MOUSE	16	30	16	34	16	29	13	27
sp|P09405|NUCL_MOUSE	16	28	15	23	9	12	11	17
sp|Q8CAQ8|MIC60_MOUSE	16	22	15	18	15	22	13	17
sp|Q99JY0|ECHB_MOUSE	16	21	13	21	15	23	13	19
sp|Q8VIJ6|SFPQ_MOUSE	16	51	12	20	12	24	10	19
sp|P24527|LKHA4_MOUSE	16	25	8	12	12	21	10	17
sp|P26039|TLN1_MOUSE	16	23	4	5	9	13	8	10
sp|P05201|AATC_MOUSE	15	27	17	23	18	33	16	27
sp|P31001|DESM_MOUSE	15	23	16	26	8	19	12	25
sp|P17182|ENOA_MOUSE	15	42	15	36	15	33	12	36
sp|P38647|GRP75_MOUSE	15	24	15	25	19	42	12	25
sp|P51667|MLRV_MOUSE	15	99	15	119	14	100	14	128
sp|Q8K2B3|SDHA_MOUSE	15	34	14	19	12	20	12	21
sp|Q7TQ48|SRCA_MOUSE	15	35	13	17	12	19	13	22
sp|P10126|EF1A1_MOUSE	15	57	13	54	15	61	16	70
sp|P68369|TBA1A_MOUSE	15	36	12	29	11	32	12	32
sp|Q64727|VINC_MOUSE	15	19	8	12	12	16	10	12
sp|Q8BH59|CMC1_MOUSE	15	20	5	6	6	10	9	13
sp|P58771|TPM1_MOUSE	14	37	16	46	15	45	18	58
sp|P63017|HSP7C_MOUSE	14	40	15	38	14	41	14	49
sp|P97807|FUMH_MOUSE	14	22	12	18	9	14	10	14
sp|Q9D0F9|PGM1_MOUSE	14	26	11	16	9	14	8	14
sp|Q3ULD5|MCCB_MOUSE	14	26	11	18	12	22	13	29
sp|P20152|VIME_MOUSE	14	20	11	16	12	15	10	20
sp|Q3UTJ2|SRBS2_MOUSE	14	20	10	16	11	14	13	18
sp|O70250|PGAM2_MOUSE	14	41	9	23	10	25	7	25
sp|Q07113|MPRI_MOUSE	14	17	7	7	6	7	10	10
sp|Q99K48|NONO_MOUSE	14	38	4	15	9	19	5	12
sp|P57780|ACTN4_MOUSE	13	31	18	36	13	26	11	21
sp|P14733|LMNB1_MOUSE	13	21	15	24	9	16	12	16
sp|Q8BWT1|THIM_MOUSE	13	25	14	23	16	26	13	20
sp|Q04447|KCRB_MOUSE	13	27	12	19	11	22	8	16

sp|P62908|RS3_MOUSE	13	17	12	15	11	18	11	18
sp|Q8BFR5|EFTU_MOUSE	13	20	12	17	9	14	11	16
sp|P05064|ALDOA_MOUSE	13	29	11	20	12	26	10	20
sp|P61979|HNRPK_MOUSE	13	22	11	22	9	21	10	22
sp|P80314|TCPB_MOUSE	13	17	9	15	8	12	8	14
sp|Q02053|UBA1_MOUSE	13	23	9	11	12	17	13	17
sp|Q9D8E6|RL4_MOUSE	13	19	9	14	9	12	10	16
sp|Q9CZS1|AL1B1_MOUSE	13	20	8	10	7	13	9	14
sp|P16125|LDHB_MOUSE	12	23	14	28	10	21	11	25
sp|Q60864|STIP1_MOUSE	12	13	13	16	7	8	8	12
sp|P48962|ADT1_MOUSE	12	30	12	32	11	33	12	35
sp|P62737|ACTA_MOUSE	12	58	12	57	9	51	9	65
sp|Q60932|VDAC1_MOUSE	12	25	11	17	6	16	9	18
sp|Q9D0K2|SCOT1_MOUSE	12	20	11	17	9	16	10	14
sp|Q9D0E1|HNRPM_MOUSE	12	17	11	14	9	14	11	23
sp|Q91ZA3|PCCA_MOUSE	12	16	11	12	13	21	17	35
sp|P09541|MYL4_MOUSE	12	24	11	28	11	23	11	19
sp|P47857|PFKAM_MOUSE	12	16	8	12	10	13	8	13
sp|Q9CZU6|CISY_MOUSE	11	22	12	26	8	15	8	19
sp|P62702|RS4X_MOUSE	11	17	12	14	8	11	10	15
sp|Q9DB77|QCR2_MOUSE	11	18	12	18	12	19	10	20
sp|Q91WD5|NDUS2_MOUSE	11	15	11	18	12	18	9	14
sp|P42932|TCPQ_MOUSE	11	17	11	14	11	13	11	15
sp|O08749|DLDH_MOUSE	11	20	11	18	10	17	11	18
sp|Q5SWU9|ACACA_MOUSE	11	15	11	15	11	13	16	20
sp|Q7TPW1|NEXN_MOUSE	11	13	11	14	8	11	12	13
sp|P50752|TNNT2_MOUSE	11	61	11	58	13	59	10	66
sp|P49813|TMOD1_MOUSE	11	18	10	17	9	14	9	11
sp|P08249|MDHM_MOUSE	11	36	9	26	12	30	9	23
sp|P45952|ACADM_MOUSE	11	23	9	13	11	17	11	17
sp|Q9DCX2|ATP5H_MOUSE	11	16	9	14	8	13	9	14
sp|Q9JKS4|LDB3_MOUSE	11	15	8	12	8	12	8	14
sp|Q61598|GDIB_MOUSE	11	15	7	11	7	11	7	10
sp|P80317|TCPZ_MOUSE	11	15	7	10	6	10	5	6
sp|Q99PL5|RRBP1_MOUSE	11	12	7	8	8	9	5	6
sp|P48036|ANXA5_MOUSE	11	15	7	11	9	14	9	13
sp|Q99LF4|RTCB_MOUSE	11	16	6	7	4	5	4	6
sp|P70414|NAC1_MOUSE	11	15	6	8	7	9	8	14
sp|P26231|CTNA1_MOUSE	11	14	6	7	6	9	6	8
sp|P70168|IMB1_MOUSE	11	20	5	10	4	5	6	13
sp|Q8K1M6|DNM1L_MOUSE	11	15	3	6	4	7	3	6
sp|Q9WTI7|MYO1C_MOUSE	10	14	14	17	10	12	15	17
sp|P51174|ACADL_MOUSE	10	20	12	20	10	21	10	26
sp|P14152|MDHC_MOUSE	10	24	11	24	10	24	9	21
sp|P19324|SERPH_MOUSE	10	12	11	12	8	11	7	12

sp|Q99LC5|ETFA_MOUSE	10	20	10	17	8	14	8	11
sp|P13020|GELS_MOUSE	10	18	10	17	11	17	13	22
sp|Q8QZT1|THIL_MOUSE	10	19	10	18	8	19	8	18
sp|O88569|ROA2_MOUSE	10	26	9	20	7	15	10	21
sp|Q9ERD7|TBB3_MOUSE	10	29	9	20	9	23	9	27
sp|Q61316|HSP74_MOUSE	10	18	9	13	9	16	7	10
sp|Q9DCW4|ETFB_MOUSE	10	20	9	16	9	17	10	19
sp|O35129|PHB2_MOUSE	10	21	9	13	7	12	5	12
sp|P47911|RL6_MOUSE	10	16	9	16	8	12	9	17
sp|P26041|MOES_MOUSE	10	15	9	13	10	16	9	12
sp|P41216|ACSL1_MOUSE	10	18	8	12	8	11	7	9
sp|Q99MN9|PCCB_MOUSE	10	17	8	12	13	20	15	27
sp|Q9WUA3|PFKAP_MOUSE	10	14	7	12	8	9	8	10
sp|Q9DC69|NDUA9_MOUSE	10	14	7	9	8	12	6	9
sp|P80318|TCPG_MOUSE	10	16	7	15	9	13	7	10
sp|Q9DBG3|AP2B1_MOUSE	10	15	6	8	8	10	3	5
sp|Q61425|HCDH_MOUSE	10	13	6	10	4	6	4	5
sp|Q8CGY6|UN45B_MOUSE	10	15	6	8	5	7	7	12
sp|Q99LC3|NDUAA_MOUSE	10	21	5	11	7	11	6	14
sp|P60843|IF4A1_MOUSE	10	14	4	8	5	5	4	7
sp|Q9Z1X4|ILF3_MOUSE	9	13	15	19	4	4	7	9
sp|P62259|1433E_MOUSE	9	18	14	22	11	18	9	18
sp|P67778|PHB_MOUSE	9	13	11	13	7	10	8	11
sp|Q9CQQ7|AT5F1_MOUSE	9	14	11	18	10	17	10	19
sp|P21550|ENOB_MOUSE	9	17	11	14	11	17	9	16
sp|P25444|RS2_MOUSE	9	16	9	14	9	13	9	14
sp|P04247|MYG_MOUSE	9	30	9	25	8	29	8	28
sp|O08638|MYH11_MOUSE	9	15	9	14	8	11	8	14
sp|Q9D6R2|IDH3A_MOUSE	9	21	8	14	6	13	7	11
sp|P12787|COX5A_MOUSE	9	19	8	14	8	15	4	8
sp|Q9D051|ODPB_MOUSE	9	16	8	14	9	19	9	15
sp|Q61656|DDX5_MOUSE	9	13	8	10	8	12	7	12
sp|P07724|ALBU_MOUSE	9	18	7	15	9	15	8	18
sp|Q91YT0|NDUV1_MOUSE	9	12	7	11	9	12	7	8
sp|Q9EQP2|EHD4_MOUSE	9	14	7	10	8	14	8	14
sp|P35486|ODPA_MOUSE	9	16	6	11	8	15	8	15
sp|P11983|TCPA_MOUSE	9	13	6	7	7	12	6	10
sp|P26040|EZRI_MOUSE	9	15	6	11	6	14	8	12
sp|Q8BMK4|CKAP4_MOUSE	9	12	6	8	6	8	8	10
sp|Q9JMH9|MY18A_MOUSE	9	11	4	6	3	4	3	3
sp|Q9WVA4|TAGL2_MOUSE	9	19	4	5	7	11	6	10
sp|Q9D8N0|EF1G_MOUSE	9	11	4	5	7	10	8	12
sp|P58774|TPM2_MOUSE	8	24	14	38	12	46	10	48
sp|Q8BWB1|SYP2L_MOUSE	8	14	12	18	8	13	9	14
sp|Q9CXY6|ILF2_MOUSE	8	12	11	20	4	6	7	10

sp|Q8VEM8|MPCP_MOUSE	8	18	10	14	8	11	7	14
sp|P02088|HBB1_MOUSE	8	30	9	23	9	37	7	31
sp|Q9CZ13|QCR1_MOUSE	8	20	9	17	8	18	9	14
sp|Q924X2|CPT1B_MOUSE	8	11	9	14	9	13	7	8
sp|P27546|MAP4_MOUSE	8	11	9	11	7	12	7	11
sp|Q9DB20|ATPO_MOUSE	8	18	8	17	6	13	6	14
sp|Q9CWF2|TBB2B_MOUSE	8	17	8	12	5	16	7	21
sp|Q9Z0X1|AIFM1_MOUSE	8	11	8	12	11	15	10	13
sp|P21981|TGM2_MOUSE	8	8	8	11	9	11	8	11
tr|Q91VA7|Q91VA7_MOUSE	8	12	7	11	9	15	9	11
sp|Q07417|ACADS_MOUSE	8	13	7	10	9	16	7	12
sp|P26443|DHE3_MOUSE	8	9	7	8	5	5	6	9
sp|P63158|HMGB1_MOUSE	8	14	7	11	6	14	5	11
sp|P14131|RS16_MOUSE	8	14	6	6	6	10	6	8
sp|P80316|TCPE_MOUSE	8	13	6	10	7	10	7	13
sp|Q6ZQ73|CAND2_MOUSE	8	9	6	7	4	6	5	7
sp|P16381|DDX3L_MOUSE	8	11	6	9	3	6	4	6
sp|P47757|CAPZB_MOUSE	8	11	6	9	5	7	6	9
sp|Q00623|APOA1_MOUSE	8	12	5	5	5	6	4	5
sp|Q91ZJ5|UGPA_MOUSE	8	12	5	7	6	10	4	5
sp|Q9CR62|M2OM_MOUSE	8	9	5	6	5	5	4	6
sp|P14206|RSSA_MOUSE	8	13	5	7	5	9	5	8
sp|P35700|PRDX1_MOUSE	8	14	5	6	8	13	7	14
sp|P42208|SEPT2_MOUSE	8	11	5	8	5	10	4	6
sp|P56959|FUS_MOUSE	8	11	5	8	5	8	4	8
sp|Q8JZN5|ACAD9_MOUSE	8	11	4	5	4	5	4	4
sp|Q8CIB5|FERM2_MOUSE	8	13	2	4	4	5	3	7
sp|Q8BH64|EHD2_MOUSE	8	8	2	2	4	6	3	4
sp|P62806|H4_MOUSE	7	36	10	40	7	33	7	45
sp|P17751|TPIS_MOUSE	7	19	10	23	9	30	9	24
sp|P14148|RL7_MOUSE	7	13	9	9	8	11	8	13
sp|P80315|TCPD_MOUSE	7	11	9	14	7	12	7	10
sp|Q921G7|ETFD_MOUSE	7	9	9	12	6	12	5	9
sp|O88342|WDR1_MOUSE	7	9	8	9	6	8	5	7
sp|Q8VEK3|HNRPU_MOUSE	7	7	8	10	9	12	7	10
sp|Q9WUZ5|TNNI1_MOUSE	7	17	8	20	7	14	7	22
sp|P19123|TNNC1_MOUSE	7	16	8	19	6	11	6	20
sp|Q62188|DPYL3_MOUSE	7	12	7	12	4	5	8	9
sp|P14602|HSPB1_MOUSE	7	10	7	9	6	9	6	9
sp|P48678|LMNA_MOUSE	7	9	7	8	7	8	3	4
sp|Q9Z2I9|SUCB1_MOUSE	7	8	7	8	8	17	8	12
sp|Q78PY7|SND1_MOUSE	7	8	7	9	5	7	6	7
sp|O55234|PSB5_MOUSE	7	9	7	9	4	6	4	6
sp|O54724|PTRF_MOUSE	7	13	7	12	6	9	7	13
sp|P62270|RS18_MOUSE	7	16	7	14	8	13	8	13

sp|Q9D855|QCR7_MOUSE	7	18	6	9	7	9	5	10
sp|P17742|PPIA_MOUSE	7	19	6	14	5	13	6	16
sp|P24369|PPIB_MOUSE	7	9	6	7	7	9	7	14
sp|P27659|RL3_MOUSE	7	13	6	14	3	5	6	13
sp|Q9CQA3|SDHB_MOUSE	7	14	6	11	5	11	5	20
sp|Q76MZ3|2AAA_MOUSE	7	10	6	8	7	8	5	6
sp|P11404|FABPH_MOUSE	7	18	6	15	5	13	5	16
sp|Q9JHI5|IVD_MOUSE	7	9	6	7	5	7	5	8
sp|Q11011|PSA_MOUSE	7	9	5	8	6	8	5	6
sp|P62962|PROF1_MOUSE	7	16	5	11	5	13	5	11
sp|Q9CYT6|CAP2_MOUSE	7	10	5	6	3	4	6	9
sp|P47754|CAZA2_MOUSE	7	14	5	8	9	17	8	12
sp|Q61595|KTN1_MOUSE	7	10	5	5	4	4	5	6
sp|P46638|RB11B_MOUSE	7	9	5	6	7	8	6	9
sp|Q9Z2I8|SUCB2_MOUSE	7	8	5	8	6	7	8	9
sp|P84084|ARF5_MOUSE	7	13	5	6	7	15	8	12
sp|Q8BMF4|ODP2_MOUSE	7	15	4	4	6	10	6	7
sp|Q9R0Y5|KAD1_MOUSE	7	14	4	8	4	6	6	10
sp|Q9DBG5|PLIN3_MOUSE	7	9	4	6	4	4	5	8
sp|P12382|PFKAL_MOUSE	7	7	4	4	6	10	6	14
sp|O35737|HNRH1_MOUSE	7	10	4	5	5	8	6	10
sp|Q6ZWX6|IF2A_MOUSE	7	9	4	9	4	8	5	7
sp|O08553|DPYL2_MOUSE	7	13	4	11	4	9	7	9
sp|Q9CZD3|GARS_MOUSE	7	10	3	3	3	4	3	4
sp|Q6ZQ38|CAND1_MOUSE	7	9	3	4	6	8	5	5
sp|P47738|ALDH2_MOUSE	7	8	3	4	3	4	2	2
sp|P99029|PRDX5_MOUSE	7	12	3	6	6	9	6	9
sp|Q61584|FXR1_MOUSE	7	7	3	4	3	3	2	3
sp|P58281|OPA1_MOUSE	7	11	2	2	4	6	2	2
sp|P50396|GDIA_MOUSE	7	10	2	2	3	3	3	3
sp|P97371|PSME1_MOUSE	7	10	2	4	6	6	2	3
sp|O70133|DHX9_MOUSE	6	9	12	17	5	5	10	12
sp|P29341|PABP1_MOUSE	6	9	11	15	4	5	4	4
sp|O09161|CASQ2_MOUSE	6	11	8	9	5	9	5	8
sp|Q99P72|RTN4_MOUSE	6	7	8	10	7	11	9	12
sp|P50580|PA2G4_MOUSE	6	8	8	10	7	10	7	11
sp|Q569Z5|DDX46_MOUSE	6	6	7	8	0	0	0	0
sp|Q6ZWN5|RS9_MOUSE	6	10	7	8	7	9	5	10
sp|Q9R062|GLYG_MOUSE	6	10	7	9	5	9	5	6
sp|Q9DBJ1|PGAM1_MOUSE	6	12	7	16	4	8	5	7
sp|Q91VR2|ATPG_MOUSE	6	11	7	11	5	8	5	8
sp|P14211|CALR_MOUSE	6	11	7	11	5	8	5	8
sp|P99027|RLA2_MOUSE	6	12	7	14	5	11	6	13
sp|P14869|RLA0_MOUSE	6	15	7	10	6	13	6	14
sp|P12970|RL7A_MOUSE	6	11	7	10	4	7	5	10

sp|Q9CZX8|RS19_MOUSE	6	11	7	11	5	6	6	8
sp|P49312|ROA1_MOUSE	6	11	6	10	5	7	4	7
sp|Q8BG05|ROA3_MOUSE	6	12	6	13	4	8	5	10
sp|P06728|APOA4_MOUSE	6	8	6	8	4	4	4	5
sp|P80313|TCPH_MOUSE	6	9	6	7	6	8	4	7
sp|P07356|ANXA2_MOUSE	6	17	6	9	8	14	7	12
sp|Q61171|PRDX2_MOUSE	6	14	6	11	6	10	6	11
sp|P62631|EF1A2_MOUSE	6	24	6	18	6	21	5	23
sp|P07901|HS90A_MOUSE	6	8	6	8	6	9	8	12
sp|P01942|HBA_MOUSE	6	41	6	39	6	38	7	44
tr|Q3UIK0|Q3UIK0_MOUSE	6	13	6	13	6	13	5	10
sp|Q9DCT2|NDUS3_MOUSE	6	9	5	8	1	1	2	5
sp|Q9QYR9|ACOT2_MOUSE	6	9	5	7	5	8	2	3
sp|P51410|RL9_MOUSE	6	14	5	5	4	9	6	13
sp|P60335|PCBP1_MOUSE	6	7	5	5	5	8	3	4
sp|P62082|RS7_MOUSE	6	12	5	9	5	7	4	9
sp|Q9D2G2|ODO2_MOUSE	6	9	5	8	7	9	5	6
sp|Q9CR68|UCRI_MOUSE	6	9	5	6	6	11	6	9
sp|Q922R8|PDIA6_MOUSE	6	9	5	7	5	6	4	6
sp|P35564|CALX_MOUSE	6	12	5	6	6	8	6	9
sp|P62751|RL23A_MOUSE	6	9	5	6	6	9	6	12
sp|Q9CZM2|RL15_MOUSE	6	19	5	22	4	8	4	11
sp|Q60931|VDAC3_MOUSE	6	12	4	6	6	10	5	12
sp|Q99MD9|NASP_MOUSE	6	8	4	5	3	4	3	6
tr|Q3TFD0|Q3TFD0_MOUSE	6	7	4	6	5	7	6	6
sp|P47963|RL13_MOUSE	6	13	4	8	5	13	5	11
sp|P30681|HMGB2_MOUSE	6	7	4	4	6	8	4	5
sp|P19253|RL13A_MOUSE	6	12	4	8	4	6	0	0
sp|P35980|RL18_MOUSE	6	10	4	9	5	8	6	10
sp|Q9R0P5|DEST_MOUSE	6	10	4	5	7	12	7	10
sp|Q9CZ44|NSF1C_MOUSE	6	6	3	4	6	8	3	3
sp|P63325|RS10_MOUSE	6	13	3	5	3	3	4	5
sp|Q9QYG0|NDRG2_MOUSE	6	7	3	4	3	5	2	3
sp|P57776|EF1D_MOUSE	6	8	3	4	4	5	3	5
sp|P09055|ITB1_MOUSE	6	9	3	5	5	10	4	5
sp|P10649|GSTM1_MOUSE	6	8	3	4	3	3	2	3
sp|Q9JIF0|ANM1_MOUSE	6	7	3	3	3	4	5	5
sp|P97351|RS3A_MOUSE	6	6	3	4	4	5	4	6
sp|Q62351|TFR1_MOUSE	6	6	2	2	0	0	0	0
sp|Q8CGK3|LONM_MOUSE	6	8	2	3	5	7	2	3
sp|P70698|PYRG1_MOUSE	6	8	2	2	5	5	3	4
sp|Q91VR5|DDX1_MOUSE	6	9	2	2	4	5	4	4
sp|P15116|CADH2_MOUSE	6	10	2	4	2	3	2	2
sp|P15532|NDKA_MOUSE	6	9	0	0	4	6	3	4
sp|Q8K310|MATR3_MOUSE	5	5	12	23	4	7	5	8

sp|Q60930|VDAC2_MOUSE	5	8	7	8	6	10	5	11
sp|Q9QXX4|CMC2_MOUSE	5	8	7	10	6	10	6	10
sp|P04117|FABP4_MOUSE	5	10	7	14	5	9	5	9
sp|P19783|COX41_MOUSE	5	10	7	10	7	15	5	12
sp|O35887|CALU_MOUSE	5	6	6	7	0	0	0	0
sp|O35286|DHX15_MOUSE	5	8	6	8	2	3	5	6
sp|P63101|1433Z_MOUSE	5	12	6	11	5	15	5	15
sp|O08756|HCD2_MOUSE	5	12	6	8	4	7	5	10
sp|Q62446|FKBP3_MOUSE	5	8	6	12	4	6	6	7
sp|Q9Z2X1|HNRPF_MOUSE	5	8	6	11	4	6	6	7
sp|P43277|H13_MOUSE	5	15	6	15	4	12	5	14
sp|Q3THE2|ML12B_MOUSE	5	7	6	10	5	13	6	13
sp|Q9QYB1|CLIC4_MOUSE	5	7	5	6	3	4	3	4
sp|Q9QYS9|QKI_MOUSE	5	6	5	6	2	2	3	5
sp|Q8BTM8|FLNA_MOUSE	5	7	5	6	4	6	2	3
sp|Q8VH51|RBM39_MOUSE	5	5	5	5	1	1	0	0
sp|Q9WUM5|SUCA_MOUSE	5	9	5	6	4	6	3	4
sp|P61358|RL27_MOUSE	5	6	5	5	3	4	3	3
sp|Q9Z1E4|GYS1_MOUSE	5	6	5	7	5	7	5	8
sp|O70373|XIRP1_MOUSE	5	7	5	7	5	5	4	5
sp|Q99PT1|GDIR1_MOUSE	5	9	5	8	4	7	5	6
sp|P68040|RACK1_MOUSE	5	7	5	6	5	7	4	7
sp|P62242|RS8_MOUSE	5	7	5	5	5	7	4	8
sp|Q60605|MYL6_MOUSE	5	11	5	13	5	12	5	13
sp|P62264|RS14_MOUSE	5	6	5	10	4	7	5	10
sp|Q9Z204|HNRPC_MOUSE	5	6	4	8	0	0	2	2
sp|P45591|COF2_MOUSE	5	11	4	5	7	12	4	9
sp|Q01768|NDKB_MOUSE	5	11	4	7	4	6	3	6
sp|P29758|OAT_MOUSE	5	8	4	6	2	2	4	4
sp|P50247|SAHH_MOUSE	5	5	4	4	2	2	2	2
sp|Q8CGP1|H2B1K_MOUSE	5	12	4	12	5	10	4	8
sp|Q64433|CH10_MOUSE	5	8	4	6	4	6	3	6
sp|Q9WV35|ABEC2_MOUSE	5	10	4	7	3	6	3	5
sp|Q9D6J6|NDUV2_MOUSE	5	9	4	5	4	6	4	8
sp|P62301|RS13_MOUSE	5	7	4	4	5	6	5	8
sp|P18572|BASI_MOUSE	5	7	4	6	4	6	4	5
sp|P70404|IDHG1_MOUSE	5	8	4	6	4	8	4	5
sp|P45376|ALDR_MOUSE	5	5	4	5	4	5	0	0
sp|Q2TPA8|HSDL2_MOUSE	5	8	4	4	3	5	6	11
sp|P47753|CAZA1_MOUSE	5	8	4	6	5	5	4	8
sp|Q99L47|F10A1_MOUSE	5	6	4	5	2	4	3	3
sp|Q9Z1Q5|CLIC1_MOUSE	5	6	4	5	3	4	4	5
sp|Q62082|MYL10_MOUSE	5	22	4	26	4	19	4	23
sp|P23116|EIF3A_MOUSE	5	5	4	5	3	4	2	3
sp|O88685|PRS6A_MOUSE	5	7	3	3	3	6	2	2

sp|Q9EQH3|VPS35_MOUSE	5	7	3	4	3	3	4	4
sp|Q9CQ62|DECR_MOUSE	5	8	3	3	0	0	1	2
sp|P08003|PDIA4_MOUSE	5	5	3	3	1	2	4	4
sp|O88533|DDC_MOUSE	5	5	3	4	4	6	3	3
sp|Q91YQ5|RPN1_MOUSE	5	6	3	4	2	2	4	5
sp|Q91Z53|GRHPR_MOUSE	5	5	3	3	3	3	2	2
sp|Q61937|NPM_MOUSE	5	9	3	4	2	3	3	8
sp|P97450|ATP5J_MOUSE	5	9	3	4	4	7	4	8
sp|P61089|UBE2N_MOUSE	5	5	3	5	2	4	2	3
sp|Q9CY58|PAIRB_MOUSE	5	8	3	3	7	8	3	6
sp|P10630|IF4A2_MOUSE	5	7	3	4	5	7	6	11
sp|Q9JHR7|IDE_MOUSE	5	5	3	3	6	7	2	2
sp|Q8BH95|ECHM_MOUSE	5	6	3	3	4	7	3	4
sp|Q9CQM9|GLRX3_MOUSE	5	6	3	4	2	3	3	3
sp|P62245|RS15A_MOUSE	5	6	2	2	3	7	3	7
sp|P02469|LAMB1_MOUSE	5	8	2	2	1	1	2	3
sp|Q64737|PUR2_MOUSE	5	11	2	4	3	4	4	5
sp|Q3UIZ8|MYLK3_MOUSE	5	7	2	2	1	1	1	2
sp|P17563|SBP1_MOUSE	5	7	2	2	4	4	2	2
sp|Q9CPP6|NDUA5_MOUSE	5	7	2	3	5	9	6	9
sp|Q8K0E8|FIBB_MOUSE	5	9	2	3	3	5	4	7
sp|Q9CQN1|TRAP1_MOUSE	5	6	2	3	2	2	2	2
sp|Q9WVJ2|PSD13_MOUSE	5	6	1	1	3	3	0	0
sp|P28650|PURA1_MOUSE	5	7	1	1	2	2	0	0
sp|Q99JY9|ARP3_MOUSE	5	6	1	2	3	5	0	0
sp|E9Q401|RYR2_MOUSE	5	7	0	0	0	0	4	4
sp|P59279|RAB2B_MOUSE	5	6	0	0	2	2	0	0
sp|Q8C2Q3|RBM14_MOUSE	5	6	0	0	0	0	0	0
sp|Q0II04|NEBL_MOUSE	4	5	8	8	6	7	8	13
sp|Q3U0V1|FUBP2_MOUSE	4	6	7	7	3	3	3	3
sp|Q3UEB3|PUF60_MOUSE	4	6	7	10	5	8	2	5
sp|Q501J6|DDX17_MOUSE	4	7	6	7	3	4	3	4
tr|Q99LF8|Q99LF8_MOUSE	4	5	6	8	3	3	0	0
sp|P53026|RL10A_MOUSE	4	7	6	7	6	10	5	8
sp|P62918|RL8_MOUSE	4	5	6	7	5	8	4	8
sp|P56399|UBP5_MOUSE	4	4	5	7	4	4	1	1
sp|Q8R1B4|EIF3C_MOUSE	4	5	5	6	0	0	2	2
sp|P14094|AT1B1_MOUSE	4	5	5	8	4	5	3	4
sp|Q8R081|HNRPL_MOUSE	4	7	5	11	3	6	3	5
sp|P23927|CRYAB_MOUSE	4	15	5	13	5	10	5	11
sp|O08709|PRDX6_MOUSE	4	6	5	6	5	6	5	6
sp|P97855|G3BP1_MOUSE	4	8	5	6	3	5	3	5
sp|P62281|RS11_MOUSE	4	6	5	5	3	5	4	6
sp|P63005|LIS1_MOUSE	4	4	5	5	3	4	2	3
sp|P26369|U2AF2_MOUSE	4	6	4	6	1	2	1	1

sp|O35381|AN32A_MOUSE	4	5	4	5	0	0	2	2
sp|Q9CQE8|CN166_MOUSE	4	5	4	4	3	3	0	0
sp|P63242|IF5A1_MOUSE	4	8	4	5	4	7	3	5
sp|Q9D880|TIM50_MOUSE	4	5	4	5	2	2	2	2
sp|Q8CGC7|SYEP_MOUSE	4	6	4	5	3	4	4	6
sp|Q9DCN2|NB5R3_MOUSE	4	7	4	6	4	6	3	4
sp|Q8VDJ3|VIGLN_MOUSE	4	4	4	5	4	6	2	2
sp|Q6PDM2|SRSF1_MOUSE	4	6	4	5	3	5	4	5
sp|P62897|CYC_MOUSE	4	8	4	5	4	11	4	10
sp|Q921F2|TADBP_MOUSE	4	5	4	5	4	7	2	3
sp|Q63918|SDPR_MOUSE	4	10	4	14	5	15	3	5
sp|Q9DC70|NDUS7_MOUSE	4	6	4	7	4	5	4	7
sp|Q9CXZ1|NDUS4_MOUSE	4	7	4	5	3	5	3	4
sp|P04370|MBP_MOUSE	4	4	4	5	3	4	3	7
sp|Q99020|ROAA_MOUSE	4	5	4	7	3	7	2	6
sp|O55126|NIPS2_MOUSE	4	8	4	9	4	9	4	10
sp|Q78IK4|MIC27_MOUSE	4	6	4	5	3	5	4	6
sp|Q9Z2U0|PSA7_MOUSE	4	4	4	4	4	7	3	4
sp|Q9DBG6|RPN2_MOUSE	4	6	4	5	3	3	5	6
sp|Q62234|MYOM1_MOUSE	4	6	4	5	5	5	6	9
sp|Q922F4|TBB6_MOUSE	4	4	3	4	2	2	1	2
sp|Q922B2|SYDC_MOUSE	4	4	3	5	2	2	3	5
sp|P19536|COX5B_MOUSE	4	13	3	5	3	7	3	9
sp|Q93092|TALDO_MOUSE	4	7	3	3	3	4	3	6
sp|Q9Z0N2|IF2H_MOUSE	4	7	3	4	3	4	2	7
sp|Q8BG32|PSD11_MOUSE	4	6	3	3	3	3	2	2
sp|Q01730|RSU1_MOUSE	4	6	3	4	2	3	3	5
sp|Q7TNC4|LC7L2_MOUSE	4	4	3	7	2	3	2	4
sp|P00405|COX2_MOUSE	4	11	3	8	5	10	4	7
sp|Q8C1B7|SEP11_MOUSE	4	7	3	3	5	5	3	3
sp|P09671|SODM_MOUSE	4	5	3	3	3	3	3	4
sp|P62849|RS24_MOUSE	4	7	3	6	3	4	4	11
sp|Q62425|NDUA4_MOUSE	4	6	3	4	2	6	4	7
sp|Q9WUZ7|SH3BG_MOUSE	4	5	3	7	2	3	3	5
sp|Q60865|CAPR1_MOUSE	4	4	3	5	2	2	3	3
sp|Q78ZA7|NP1L4_MOUSE	4	5	3	4	3	3	4	5
sp|P99024|TBB5_MOUSE	4	11	3	12	4	6	4	11
sp|P0DP27|CALM2_MOUSE	4	8	3	7	2	5	2	5
sp|Q7TPR4|ACTN1_MOUSE	4	9	3	6	3	8	3	9
sp|P68510|1433F_MOUSE	4	6	3	5	5	8	4	7
sp|P62852|RS25_MOUSE	4	6	3	6	3	5	3	6
sp|Q9DCZ1|GMPR1_MOUSE	4	6	2	2	2	4	1	2
sp|Q8R1Q8|DC1L1_MOUSE	4	5	2	3	4	5	3	3
sp|P05132|KAPCA_MOUSE	4	7	2	3	2	2	1	1
sp|Q9CQH3|NDUB5_MOUSE	4	6	2	4	2	4	3	6

sp|O55222|ILK_MOUSE	4	6	2	2	3	3	2	3
sp|P54227|STMN1_MOUSE	4	6	2	3	3	4	1	2
sp|Q99LP6|GRPE1_MOUSE	4	4	2	3	3	4	1	1
sp|P59999|ARPC4_MOUSE	4	5	2	2	2	3	2	2
sp|O35685|NUDC_MOUSE	4	6	2	3	3	3	1	1
sp|Q9QUR6|PPCE_MOUSE	4	5	2	3	3	5	1	1
sp|P47934|CACP_MOUSE	4	6	2	2	5	6	0	0
sp|Q9ES83|POPD1_MOUSE	4	6	2	4	2	3	1	2
sp|O08807|PRDX4_MOUSE	4	6	2	4	3	4	2	3
sp|P43276|H15_MOUSE	4	6	2	5	3	4	3	4
sp|P14685|PSMD3_MOUSE	4	4	2	3	4	4	0	0
sp|P68254|1433T_MOUSE	4	4	2	3	3	5	3	4
sp|E9PVA8|GCN1_MOUSE	4	4	1	1	0	0	1	1
sp|P46471|PRS7_MOUSE	4	6	1	1	2	4	1	1
sp|P46935|NEDD4_MOUSE	4	4	1	1	2	2	3	3
sp|P13595|NCAM1_MOUSE	4	8	1	1	1	1	1	2
sp|P62196|PRS8_MOUSE	4	6	1	1	2	2	2	2
sp|P62192|PRS4_MOUSE	4	5	1	1	1	1	0	0
sp|Q8JZQ2|AFG32_MOUSE	4	5	1	1	1	1	0	0
sp|Q8K0D5|EFGM_MOUSE	4	5	1	1	1	2	1	1
sp|O88844|IDHC_MOUSE	4	6	1	1	3	8	1	3
sp|P70349|HINT1_MOUSE	4	5	1	1	2	3	2	3
sp|Q61081|CDC37_MOUSE	4	4	1	1	0	0	0	0
sp|Q9EQ20|MMSA_MOUSE	4	4	1	2	0	0	0	0
sp|Q9DCL9|PUR6_MOUSE	4	5	1	1	0	0	0	0
sp|Q05793|PGBM_MOUSE	4	4	1	2	2	2	3	3
sp|Q9CQ60|6PGL_MOUSE	4	4	1	1	2	3	2	3
tr|Q9CQ73|Q9CQ73_MOUSE	4	5	1	2	0	0	3	4
sp|P99026|PSB4_MOUSE	4	7	1	2	3	4	3	4
sp|Q99PV0|PRP8_MOUSE	4	4	0	0	1	1	2	2
sp|Q9JJI8|RL38_MOUSE	4	6	0	0	2	3	0	0
sp|Q61768|KINH_MOUSE	4	4	0	0	0	0	0	0
sp|O88587|COMT_MOUSE	4	4	0	0	0	0	0	0
sp|P51150|RAB7A_MOUSE	4	5	0	0	4	5	2	2
sp|P40124|CAP1_MOUSE	4	5	0	0	0	0	0	0
sp|Q7TMK9|HNRPQ_MOUSE	3	4	12	15	5	7	5	5
sp|P17225|PTBP1_MOUSE	3	5	8	16	2	6	4	7
sp|Q8CJG0|AGO2_MOUSE	3	3	6	8	1	1	0	0
sp|O09167|RL21_MOUSE	3	6	5	9	2	5	3	5
sp|Q6IRU2|TPM4_MOUSE	3	4	5	5	4	4	4	4
sp|Q9D0M3|CY1_MOUSE	3	8	5	7	4	7	5	12
sp|P68037|UB2L3_MOUSE	3	4	4	5	4	8	2	3
sp|Q9Z130|HNRDL_MOUSE	3	5	4	6	1	1	0	0
sp|P40142|TKT_MOUSE	3	6	4	6	4	7	3	4
sp|P70372|ELAV1_MOUSE	3	4	4	5	2	3	1	1

sp|Q6P8J7|KCRS_MOUSE	3	15	4	10	4	12	3	9
sp|Q8BKC5|IPO5_MOUSE	3	4	4	5	2	3	3	4
sp|Q61545|EWS_MOUSE	3	4	4	4	1	1	2	3
sp|Q9CPR4|RL17_MOUSE	3	6	4	5	2	5	2	4
sp|P42125|ECI1_MOUSE	3	6	4	5	4	6	4	5
sp|P18760|COF1_MOUSE	3	6	4	5	4	5	3	5
sp|Q9DB15|RM12_MOUSE	3	3	4	4	4	5	4	4
sp|Q9CPQ1|COX6C_MOUSE	3	6	4	6	4	5	0	0
sp|P70296|PEBP1_MOUSE	3	7	4	5	3	5	3	7
sp|Q9DBC7|KAP0_MOUSE	3	3	4	4	3	6	3	4
sp|Q9Z1W9|STK39_MOUSE	3	4	3	4	2	3	2	3
sp|Q3URD3|SLMAP_MOUSE	3	4	3	4	2	2	4	5
sp|Q9DCD0|6PGD_MOUSE	3	5	3	4	3	4	4	5
sp|Q8VDM4|PSMD2_MOUSE	3	5	3	3	2	2	4	5
sp|Q60749|KHDR1_MOUSE	3	6	3	5	2	4	3	4
sp|Q9EQU5|SET_MOUSE	3	4	3	4	3	4	2	2
sp|Q9CPN8|IF2B3_MOUSE	3	3	3	5	0	0	0	0
sp|C0HKE3|H2A1D_MOUSE	3	10	3	10	3	8	2	7
sp|Q8BJW6|EIF2A_MOUSE	3	3	3	3	1	1	3	3
sp|P17156|HSP72_MOUSE	3	4	3	6	4	7	4	7
sp|P62754|RS6_MOUSE	3	5	3	5	4	7	4	8
sp|P61255|RL26_MOUSE	3	5	3	5	3	4	4	4
tr|Q99N15|Q99N15_MOUSE	3	4	3	5	3	3	2	4
sp|O35658|C1QBP_MOUSE	3	4	3	4	4	4	2	7
sp|Q9CXW4|RL11_MOUSE	3	5	3	3	2	2	3	4
sp|P61014|PPLA_MOUSE	3	4	3	5	2	4	1	1
sp|O70251|EF1B_MOUSE	3	8	3	6	3	5	3	8
sp|P51881|ADT2_MOUSE	3	8	3	5	2	5	2	5
sp|P60867|RS20_MOUSE	3	4	3	3	2	3	2	2
sp|Q925I1|ATAD3_MOUSE	3	4	3	3	2	2	2	2
sp|Q8BSL7|ARF2_MOUSE	3	8	3	8	3	8	3	9
sp|P08752|GNAI2_MOUSE	3	6	3	6	3	8	2	3
sp|P63028|TCTP_MOUSE	3	4	3	5	1	3	1	2
sp|Q9R1P1|PSB3_MOUSE	3	5	3	4	3	5	0	0
sp|P62137|PP1A_MOUSE	3	4	3	3	4	5	5	6
sp|P63276|RS17_MOUSE	3	4	3	5	0	0	2	3
sp|P35979|RL12_MOUSE	3	6	3	6	3	9	3	7
sp|Q9QUM9|PSA6_MOUSE	3	3	3	5	2	3	2	3
sp|Q9D6U8|F162A_MOUSE	3	6	3	5	3	4	3	4
sp|P41105|RL28_MOUSE	3	4	3	3	3	3	4	7
sp|Q60692|PSB6_MOUSE	3	5	3	5	4	8	3	5
sp|P62900|RL31_MOUSE	3	4	3	6	3	8	4	8
sp|P68433|H31_MOUSE	3	6	3	6	0	0	3	6
sp|P70195|PSB7_MOUSE	3	4	3	4	0	0	2	4
sp|P26638|SYSC_MOUSE	3	5	2	2	2	3	1	2

sp|P54822|PUR8_MOUSE	3	4	2	2	2	3	1	1
sp|O08583|THOC4_MOUSE	3	3	2	5	1	2	2	2
sp|O08528|HXK2_MOUSE	3	4	2	2	1	1	0	0
sp|Q9Z2U1|PSA5_MOUSE	3	4	2	3	1	1	0	0
sp|Q99L13|3HIDH_MOUSE	3	4	2	3	1	1	0	0
sp|Q8QZS1|HIBCH_MOUSE	3	6	2	3	2	3	4	5
sp|P54728|RD23B_MOUSE	3	3	2	2	0	0	1	1
sp|P97822|AN32E_MOUSE	3	5	2	3	2	3	1	1
sp|Q61390|TCPW_MOUSE	3	3	2	2	3	4	3	5
sp|Q9D6J5|NDUB8_MOUSE	3	5	2	2	0	0	2	3
sp|O89086|RBM3_MOUSE	3	7	2	5	2	3	2	3
sp|Q8BP67|RL24_MOUSE	3	4	2	2	2	2	3	5
sp|Q91W97|HKDC1_MOUSE	3	3	2	2	1	1	1	1
sp|P62827|RAN_MOUSE	3	7	2	3	2	5	3	6
tr|Q8C7C3|Q8C7C3_MOUSE	3	4	2	2	2	2	0	0
sp|O09111|NDUBB_MOUSE	3	4	2	2	2	2	2	2
sp|Q91WQ3|SYYC_MOUSE	3	3	2	2	1	1	0	0
sp|Q6NZJ6|IF4G1_MOUSE	3	4	2	2	0	0	0	0
sp|Q9Z1Z2|STRAP_MOUSE	3	3	2	2	0	0	0	0
sp|P46425|GSTP2_MOUSE	3	4	2	3	3	3	2	2
sp|Q5SW19|CLU_MOUSE	3	4	2	3	0	0	0	0
sp|Q9Z1Q9|SYVC_MOUSE	3	3	2	2	0	0	0	0
sp|Q8R4N0|CLYBL_MOUSE	3	3	2	3	3	4	3	3
sp|O55023|IMPA1_MOUSE	3	3	2	2	2	3	0	0
sp|Q02248|CTNB1_MOUSE	3	4	2	3	0	0	0	0
sp|P34022|RANG_MOUSE	3	5	2	3	2	3	2	4
sp|Q9CQV8|1433B_MOUSE	3	7	2	3	4	8	3	9
sp|Q9ERS2|NDUAD_MOUSE	3	5	2	2	2	3	3	5
sp|P62855|RS26_MOUSE	3	7	2	4	2	4	2	3
sp|P32067|LA_MOUSE	3	4	2	2	0	0	0	0
sp|P62267|RS23_MOUSE	3	4	2	3	0	0	0	0
sp|Q9CPY7|AMPL_MOUSE	3	5	2	3	3	3	3	4
sp|Q9CWJ9|PUR9_MOUSE	3	3	2	3	2	3	0	0
sp|P62869|ELOB_MOUSE	3	3	2	3	2	2	3	3
sp|Q9CQ75|NDUA2_MOUSE	3	4	2	3	4	5	3	6
sp|P47962|RL5_MOUSE	3	4	2	2	3	4	4	6
sp|P62889|RL30_MOUSE	3	4	2	3	2	3	3	6
sp|Q3TEA8|HP1B3_MOUSE	3	3	2	2	0	0	2	2
sp|Q9CQZ6|NDUB3_MOUSE	3	4	2	3	2	3	2	3
tr|Q8VCQ8|Q8VCQ8_MOUSE	3	3	2	4	2	2	2	2
sp|Q9D819|IPYR_MOUSE	3	3	1	1	0	0	1	1
sp|Q91V41|RAB14_MOUSE	3	4	1	1	3	4	3	4
sp|O08739|AMPD3_MOUSE	3	5	1	2	1	1	0	0
sp|P02772|FETA_MOUSE	3	6	1	2	2	4	3	5
sp|P02468|LAMC1_MOUSE	3	4	1	1	2	2	2	2

sp|Q9CZ30|OLA1_MOUSE	3	4	1	2	2	3	2	2
sp|O55131|SEPT7_MOUSE	3	6	1	2	2	2	2	2
sp|Q9D6K8|FUND2_MOUSE	3	3	1	1	2	3	1	3
sp|Q9WVA3|BUB3_MOUSE	3	4	1	1	2	2	1	1
sp|P61087|UBE2K_MOUSE	3	3	1	1	0	0	0	0
sp|Q9CVB6|ARPC2_MOUSE	3	3	1	1	0	0	4	4
sp|P62821|RAB1A_MOUSE	3	4	1	1	0	0	0	0
tr|Q3UMT8|Q3UMT8_MOUSE	3	4	1	1	0	0	0	0
sp|Q9CPV4|GLOD4_MOUSE	3	4	1	1	0	0	0	0
sp|P11798|KCC2A_MOUSE	3	3	1	1	1	2	1	4
sp|Q66JS6|EI3JB_MOUSE	3	3	1	1	0	0	0	0
sp|Q8R5C5|ACTY_MOUSE	3	5	1	2	2	3	2	2
sp|P53986|MOT1_MOUSE	3	3	1	2	1	1	1	1
sp|Q791V5|MTCH2_MOUSE	3	3	0	0	1	1	1	1
sp|Q62433|NDRG1_MOUSE	3	3	0	0	2	2	0	0
sp|Q8R326|PSPC1_MOUSE	3	6	0	0	0	0	1	3
sp|P60229|EIF3E_MOUSE	3	4	0	0	1	1	0	0
sp|Q99PR8|HSPB2_MOUSE	3	6	0	0	3	6	2	3
sp|Q924M7|MPI_MOUSE	3	3	0	0	0	0	1	1
sp|Q7TNG5|EMAL2_MOUSE	3	4	0	0	2	2	2	2
sp|P19096|FAS_MOUSE	3	5	0	0	2	3	2	2
sp|Q99JB2|STML2_MOUSE	3	5	0	0	2	2	1	1
sp|Q9CR16|PPID_MOUSE	3	3	0	0	1	1	1	2
sp|Q9CQ54|NDUC2_MOUSE	3	6	0	0	2	3	0	0
sp|Q9CPU0|LGUL_MOUSE	3	3	0	0	2	2	0	0
sp|Q02819|NUCB1_MOUSE	3	3	0	0	2	2	1	1
sp|P61222|ABCE1_MOUSE	3	4	0	0	0	0	0	0
sp|Q3UPL0|SC31A_MOUSE	3	6	0	0	0	0	0	0
sp|P61027|RAB10_MOUSE	3	6	0	0	0	0	0	0
sp|Q9JII6|AK1A1_MOUSE	3	3	0	0	0	0	1	1
sp|Q61548|AP180_MOUSE	3	4	0	0	0	0	0	0
sp|Q8BGK2|ARHL1_MOUSE	3	5	0	0	1	2	1	1
sp|Q5EBG6|HSPB6_MOUSE	3	3	0	0	0	0	0	0
sp|Q08857|CD36_MOUSE	3	3	0	0	0	0	0	0
sp|Q9QXS1|PLEC_MOUSE	3	3	0	0	0	0	0	0
sp|P97825|JUPI1_MOUSE	3	5	0	0	2	2	0	0
tr|G5E8J6|G5E8J6_MOUSE	3	6	0	0	2	3	2	4
sp|Q9ER00|STX12_MOUSE	3	3	0	0	0	0	0	0
sp|Q6P5F9|XPO1_MOUSE	3	3	0	0	2	2	0	0
sp|Q91VM9|IPYR2_MOUSE	3	3	0	0	2	2	3	3
sp|Q01405|SC23A_MOUSE	3	3	0	0	2	2	1	1
sp|P10107|ANXA1_MOUSE	3	3	0	0	0	0	2	2
sp|Q9DBP5|KCY_MOUSE	3	3	0	0	0	0	0	0
sp|Q00915|RET1_MOUSE	3	3	0	0	1	2	0	0
sp|P62835|RAP1A_MOUSE	3	4	0	0	3	4	4	4

sp|Q8R2Y2|MUC18_MOUSE	3	3	0	0	3	4	0	0
sp|O88477|IF2B1_MOUSE	2	2	7	10	3	3	0	0
sp|Q91Z31|PTBP2_MOUSE	2	3	7	11	4	5	2	3
sp|Q91WJ8|FUBP1_MOUSE	2	2	6	8	0	0	2	2
sp|O35459|ECH1_MOUSE	2	3	6	8	3	3	2	3
sp|P62960|YBOX1_MOUSE	2	5	5	5	3	4	4	5
sp|Q9CQR4|ACO13_MOUSE	2	5	4	5	3	7	3	3
sp|Q61990|PCBP2_MOUSE	2	4	4	6	3	6	0	0
sp|Q8CIE6|COPA_MOUSE	2	3	4	5	2	3	2	3
sp|Q9D172|ES1_MOUSE	2	3	4	4	3	7	4	5
sp|Q3U1J4|DDB1_MOUSE	2	4	3	4	0	0	1	1
sp|P61161|ARP2_MOUSE	2	4	3	4	3	3	1	3
sp|Q9DCJ5|NDUA8_MOUSE	2	6	3	3	2	3	2	4
tr|A8DUK4|A8DUK4_MOUSE	2	10	3	8	2	7	2	6
sp|Q8K1Z0|COQ9_MOUSE	2	2	3	3	2	2	2	2
sp|Q6NVF9|CPSF6_MOUSE	2	2	3	5	1	1	2	2
sp|Q99KN9|EPN4_MOUSE	2	4	3	3	1	1	1	2
sp|Q9ESZ8|GTF2I_MOUSE	2	4	3	3	0	0	2	3
sp|P68368|TBA4A_MOUSE	2	2	3	3	0	0	1	1
sp|Q71LX4|TLN2_MOUSE	2	4	3	3	2	2	0	0
sp|Q06185|ATP5I_MOUSE	2	4	3	3	3	3	2	4
sp|Q9CR57|RL14_MOUSE	2	5	3	4	2	5	2	5
sp|Q9DCS9|NDUBA_MOUSE	2	3	3	4	3	5	2	4
sp|P00920|CAH2_MOUSE	2	3	3	4	3	5	2	4
sp|O09131|GSTO1_MOUSE	2	3	3	3	2	3	2	3
sp|Q9D6F9|TBB4A_MOUSE	2	5	3	9	1	2	2	6
sp|Q8BHN3|GANAB_MOUSE	2	3	3	3	0	0	0	0
tr|B7ZCI2|B7ZCI2_MOUSE	2	3	3	3	5	8	6	10
sp|P30999|CTND1_MOUSE	2	2	3	4	1	2	2	4
sp|P48787|TNNI3_MOUSE	2	4	3	6	4	7	3	5
sp|Q9WV02|RBMX_MOUSE	2	2	3	5	3	4	2	3
sp|Q61029|LAP2B_MOUSE	2	3	3	4	2	2	2	5
sp|Q9R1P3|PSB2_MOUSE	2	2	3	4	2	3	2	3
sp|Q9R0P9|UCHL1_MOUSE	2	2	3	4	0	0	2	5
sp|P97379|G3BP2_MOUSE	2	2	3	4	1	2	2	2
sp|O09061|PSB1_MOUSE	2	3	3	4	0	0	0	0
sp|P18242|CATD_MOUSE	2	3	2	2	1	2	1	2
sp|O88544|CSN4_MOUSE	2	3	2	2	1	1	1	1
sp|Q9WV27|AT1A4_MOUSE	2	5	2	2	1	2	2	3
sp|P97372|PSME2_MOUSE	2	3	2	2	3	3	1	1
sp|Q923D2|BLVRB_MOUSE	2	2	2	3	1	1	2	2
tr|Q91VB8|Q91VB8_MOUSE	2	3	2	4	2	2	2	3
sp|P47955|RLA1_MOUSE	2	3	2	4	2	2	2	3
sp|P62874|GBB1_MOUSE	2	3	2	3	0	0	2	4
sp|Q922B1|MACD1_MOUSE	2	2	2	2	0	0	0	0

sp|Q99MN1|SYK_MOUSE	2	3	2	3	0	0	1	1
sp|O70435|PSA3_MOUSE	2	3	2	2	1	2	1	1
sp|O35855|BCAT2_MOUSE	2	3	2	2	1	1	1	1
sp|Q9DC77|SMPX_MOUSE	2	3	2	2	1	1	0	0
sp|P36536|SAR1A_MOUSE	2	4	2	3	1	3	2	3
sp|P52503|NDUS6_MOUSE	2	4	2	2	2	3	1	2
sp|O54879|HMGB3_MOUSE	2	2	2	3	2	3	1	1
sp|Q8R3H7|HS2ST_MOUSE	2	4	2	2	1	1	0	0
sp|P62911|RL32_MOUSE	2	2	2	3	2	2	1	1
sp|Q5FWK3|RHG01_MOUSE	2	5	2	2	3	3	0	0
sp|Q61820|RANT_MOUSE	2	5	2	2	2	2	3	4
sp|P51660|DHB4_MOUSE	2	2	2	2	1	1	1	1
sp|P43274|H14_MOUSE	2	7	2	3	2	6	1	3
sp|Q6ZWU9|RS27_MOUSE	2	4	2	2	2	2	2	3
sp|P43275|H11_MOUSE	2	5	2	3	2	3	2	3
sp|A2AAJ9|OBSCN_MOUSE	2	2	2	2	2	2	2	2
sp|Q9DCZ4|MIC26_MOUSE	2	4	2	3	2	3	2	3
sp|Q8BGY2|IF5A2_MOUSE	2	2	2	2	2	2	1	2
sp|Q9R0Q3|TMED2_MOUSE	2	3	2	3	2	4	1	1
sp|P56391|CX6B1_MOUSE	2	6	2	7	1	4	2	5
sp|Q07646|MEST_MOUSE	2	2	2	2	4	6	3	5
sp|P14115|RL27A_MOUSE	2	4	2	3	2	3	2	4
sp|Q05816|FABP5_MOUSE	2	4	2	3	2	4	2	4
sp|P03930|ATP8_MOUSE	2	3	2	2	3	5	2	4
sp|P13541|MYH3_MOUSE	2	4	2	6	2	4	2	6
sp|Q60668|HNRPD_MOUSE	2	5	2	4	2	5	2	3
sp|Q9CQR2|RS21_MOUSE	2	4	2	4	2	3	2	4
sp|P62843|RS15_MOUSE	2	5	2	4	4	7	3	7
sp|P15331|PERI_MOUSE	2	2	2	2	1	2	2	2
sp|Q9D3D9|ATPD_MOUSE	2	10	2	8	1	8	1	9
sp|P00493|HPRT_MOUSE	2	3	2	2	0	0	0	0
sp|P56392|CX7A1_MOUSE	2	3	2	3	2	3	2	5
sp|Q62093|SRSF2_MOUSE	2	3	2	4	3	5	2	3
sp|Q5FW52|MLIP_MOUSE	2	3	2	2	3	3	0	0
sp|P84104|SRSF3_MOUSE	2	3	2	3	2	2	2	4
sp|P62858|RS28_MOUSE	2	3	2	3	2	5	2	6
sp|Q9R0P3|ESTD_MOUSE	2	3	2	3	2	4	2	2
sp|P11031|TCP4_MOUSE	2	2	2	2	2	2	2	4
sp|Q6A0A9|F120A_MOUSE	2	2	2	2	0	0	0	0
sp|P97315|CSRP1_MOUSE	2	2	2	3	2	3	2	3
sp|P61982|1433G_MOUSE	2	2	2	2	2	3	0	0
sp|Q9JIF7|COPB_MOUSE	2	2	2	2	0	0	2	3
sp|Q9CRB9|MIC19_MOUSE	2	2	2	4	3	6	3	5
sp|P62830|RL23_MOUSE	2	4	2	4	3	4	3	6
tr|Q9CZ19|Q9CZ19_MOUSE	2	4	2	4	3	4	3	5

sp|P17879|HS71B_MOUSE	2	3	2	5	2	5	2	6
sp|P48771|CX7A2_MOUSE	2	5	2	5	2	6	2	3
sp|Q9CR61|NDUB7_MOUSE	2	2	2	3	2	2	2	3
sp|O88487|DC1I2_MOUSE	2	3	2	4	2	3	0	0
sp|Q8BWF0|SSDH_MOUSE	2	4	1	1	1	3	2	3
sp|Q921M3|SF3B3_MOUSE	2	3	1	1	1	1	1	1
sp|Q64152|BTF3_MOUSE	2	3	1	3	1	2	2	4
sp|Q9CQC7|NDUB4_MOUSE	2	4	1	1	2	2	1	1
sp|Q9ET01|PYGL_MOUSE	2	2	1	1	0	0	1	2
sp|Q7TNP2|2AAB_MOUSE	2	3	1	1	1	2	1	3
sp|Q9D1G1|RAB1B_MOUSE	2	2	1	1	1	1	0	0
sp|Q05D44|IF2P_MOUSE	2	2	1	1	0	0	1	1
sp|P97447|FHL1_MOUSE	2	4	1	1	0	0	2	3
sp|P58389|PTPA_MOUSE	2	3	1	1	1	1	2	2
sp|Q3UHX2|HAP28_MOUSE	2	3	1	1	0	0	1	1
sp|Q9DBR7|MYPT1_MOUSE	2	3	1	1	2	3	2	4
sp|Q5XJY5|COPD_MOUSE	2	3	1	1	1	1	0	0
sp|Q925E7|2ABD_MOUSE	2	3	1	3	1	1	1	1
sp|P47915|RL29_MOUSE	2	2	1	1	0	0	0	0
sp|Q9CQC9|SAR1B_MOUSE	2	4	1	2	2	3	1	3
sp|Q9D1D4|TMEDA_MOUSE	2	4	1	2	1	1	1	1
sp|Q9DAM7|TM263_MOUSE	2	3	1	1	1	1	1	2
sp|O70456|1433S_MOUSE	2	3	1	1	4	6	2	5
sp|Q8BK64|AHSA1_MOUSE	2	3	1	2	1	1	1	1
sp|Q6PDI5|ECM29_MOUSE	2	2	1	2	0	0	2	2
sp|O54984|ASNA_MOUSE	2	2	1	1	0	0	1	1
sp|P53395|ODB2_MOUSE	2	3	1	1	0	0	1	1
sp|O08795|GLU2B_MOUSE	2	3	1	1	2	3	2	3
sp|P61750|ARF4_MOUSE	2	2	1	2	1	1	1	1
sp|Q99LX0|PARK7_MOUSE	2	3	1	1	1	2	3	3
sp|P67984|RL22_MOUSE	2	2	1	1	1	2	1	1
sp|Q60872|IF1A_MOUSE	2	2	1	1	1	1	1	2
sp|P51859|HDGF_MOUSE	2	2	1	1	1	2	2	4
sp|Q9QYJ0|DNJA2_MOUSE	2	2	1	2	1	1	2	4
sp|Q9Z1D1|EIF3G_MOUSE	2	2	1	1	1	2	1	1
sp|P10605|CATB_MOUSE	2	3	1	3	2	5	0	0
sp|Q9CWZ3|RBM8A_MOUSE	2	2	1	2	0	0	1	1
sp|O88441|MTX2_MOUSE	2	4	1	3	2	5	1	2
sp|Q9DAK9|PHP14_MOUSE	2	2	1	1	1	1	2	2
sp|P26645|MARCS_MOUSE	2	3	1	1	4	7	2	2
sp|Q91V12|BACH_MOUSE	2	2	1	1	3	3	0	0
sp|Q9Z1N5|DX39B_MOUSE	2	2	1	2	1	1	1	1
sp|Q9CQ19|MYL9_MOUSE	2	3	1	2	1	1	1	2
sp|Q9D8W5|PSD12_MOUSE	2	2	1	1	1	2	0	0
sp|Q9WUK2|IF4H_MOUSE	2	2	1	2	2	3	2	3

sp|Q9Z2W0|DNPEP_MOUSE	2	2	1	1	1	1	0	0
sp|P99028|QCR6_MOUSE	2	3	1	1	1	1	0	0
sp|Q99KP6|PRP19_MOUSE	2	3	1	1	0	0	1	1
sp|Q9WV92|E41L3_MOUSE	2	2	1	1	0	0	1	1
sp|Q9EQI8|RM46_MOUSE	2	3	1	2	2	3	0	0
sp|O54734|OST48_MOUSE	2	2	1	1	2	3	1	1
sp|Q9CX34|SGT1_MOUSE	2	3	1	1	0	0	0	0
sp|P52293|IMA1_MOUSE	2	2	1	1	0	0	0	0
sp|Q8BX10|PGAM5_MOUSE	2	2	1	1	0	0	0	0
sp|Q9CX86|ROA0_MOUSE	2	4	1	2	1	2	1	2
sp|Q99KJ8|DCTN2_MOUSE	2	2	1	2	0	0	3	4
sp|P62141|PP1B_MOUSE	2	2	1	1	2	2	2	2
sp|Q8CDN6|TXNL1_MOUSE	2	2	1	2	2	2	2	2
sp|P02104|HBE_MOUSE	2	2	1	2	2	3	1	2
sp|Q9JJW6|ALRF2_MOUSE	2	2	1	1	1	1	0	0
sp|Q05186|RCN1_MOUSE	2	2	1	2	0	0	1	2
sp|P61294|RAB6B_MOUSE	2	2	1	1	0	0	0	0
sp|P34914|HYES_MOUSE	2	3	0	0	1	1	0	0
sp|Q5SX39|MYH4_MOUSE	2	5	0	0	1	2	2	7
sp|Q9CQ65|MTAP_MOUSE	2	3	0	0	1	1	0	0
sp|P07742|RIR1_MOUSE	2	4	0	0	2	3	1	1
sp|Q8R001|MARE2_MOUSE	2	3	0	0	1	1	0	0
sp|Q9DAW9|CNN3_MOUSE	2	4	0	0	1	1	1	1
sp|P38060|HMGCL_MOUSE	2	4	0	0	1	1	3	4
sp|P62334|PRS10_MOUSE	2	3	0	0	2	3	0	0
sp|P62996|TRA2B_MOUSE	2	2	0	0	0	0	0	0
sp|Q3THK7|GUAA_MOUSE	2	2	0	0	0	0	0	0
sp|Q8K019|BCLF1_MOUSE	2	3	0	0	1	1	0	0
sp|Q9Z2I0|LETM1_MOUSE	2	3	0	0	0	0	2	3
sp|P30416|FKBP4_MOUSE	2	2	0	0	3	4	2	3
sp|E9PV24|FIBA_MOUSE	2	3	0	0	2	2	1	1
sp|Q6P1B1|XPP1_MOUSE	2	3	0	0	0	0	1	1
sp|P23198|CBX3_MOUSE	2	4	0	0	2	4	1	1
sp|P56135|ATPK_MOUSE	2	5	0	0	2	3	2	3
sp|Q91UZ5|IMPA2_MOUSE	2	2	0	0	0	0	1	1
sp|P49722|PSA2_MOUSE	2	2	0	0	2	3	1	1
sp|Q07076|ANXA7_MOUSE	2	2	0	0	0	0	0	0
sp|Q64514|TPP2_MOUSE	2	2	0	0	1	1	0	0
sp|Q9DCH4|EIF3F_MOUSE	2	3	0	0	0	0	1	1
sp|Q9DBL1|ACDSB_MOUSE	2	3	0	0	1	2	0	0
sp|Q9CXU9|EIF1B_MOUSE	2	3	0	0	0	0	1	2
sp|Q9D1B9|RM28_MOUSE	2	3	0	0	0	0	1	1
sp|Q9DB05|SNAA_MOUSE	2	2	0	0	0	0	1	1
sp|Q61553|FSCN1_MOUSE	2	3	0	0	2	2	0	0
sp|P35550|FBRL_MOUSE	2	2	0	0	0	0	0	0

sp|Q3TXS7|PSMD1_MOUSE	2	3	0	0	0	0	1	3
sp|Q9QXY6|EHD3_MOUSE	2	2	0	0	1	2	1	1
sp|Q9WTP6|KAD2_MOUSE	2	3	0	0	0	0	2	3
sp|P11352|GPX1_MOUSE	2	2	0	0	2	2	1	1
sp|Q9CQZ5|NDUA6_MOUSE	2	3	0	0	0	0	2	3
sp|P28271|ACOC_MOUSE	2	2	0	0	0	0	1	1
sp|Q62009|POSTN_MOUSE	2	2	0	0	0	0	2	2
sp|Q8BU30|SYIC_MOUSE	2	2	0	0	1	1	0	0
sp|Q9WVK4|EHD1_MOUSE	2	2	0	0	0	0	0	0
sp|O35857|TIM44_MOUSE	2	2	0	0	1	1	0	0
sp|P63001|RAC1_MOUSE	2	2	0	0	2	2	1	1
sp|Q9QZD9|EIF3I_MOUSE	2	2	0	0	1	2	0	0
sp|Q922D8|C1TC_MOUSE	2	2	0	0	2	2	0	0
sp|Q8CI51|PDLI5_MOUSE	2	3	0	0	3	3	4	4
sp|Q9JLV1|BAG3_MOUSE	2	3	0	0	3	3	0	0
sp|Q62159|RHOC_MOUSE	2	3	0	0	0	0	0	0
sp|Q6P1F6|2ABA_MOUSE	2	3	0	0	0	0	3	4
sp|Q8VDW0|DX39A_MOUSE	2	2	0	0	2	2	0	0
sp|Q64442|DHSO_MOUSE	2	3	0	0	0	0	0	0
sp|Q9JKB1|UCHL3_MOUSE	2	3	0	0	0	0	0	0
sp|Q91WK1|SPRY4_MOUSE	2	3	0	0	0	0	0	0
sp|Q9CQ69|QCR8_MOUSE	2	3	0	0	0	0	0	0
sp|Q9DBH5|LMAN2_MOUSE	2	2	0	0	0	0	0	0
sp|O70194|EIF3D_MOUSE	2	2	0	0	0	0	3	4
tr|Q91X76|Q91X76_MOUSE	2	2	0	0	0	0	0	0
sp|Q5SUF2|LC7L3_MOUSE	2	2	0	0	0	0	0	0
sp|P28656|NP1L1_MOUSE	2	2	0	0	2	3	0	0
sp|Q8QZY1|EIF3L_MOUSE	2	2	0	0	0	0	0	0
sp|Q7TPV4|MBB1A_MOUSE	2	2	0	0	0	0	0	0
sp|O35593|PSDE_MOUSE	2	2	0	0	0	0	0	0
sp|Q921H8|THIKA_MOUSE	2	2	0	0	0	0	0	0
sp|Q9CZW5|TOM70_MOUSE	2	2	0	0	0	0	1	1
sp|Q8VCM7|FIBG_MOUSE	2	2	0	0	0	0	0	0
sp|Q8BHC4|DCAKD_MOUSE	2	2	0	0	0	0	0	0
tr|A0A0R4J083|A0A0R4J083_MOUSE	2	4	0	0	0	0	2	6
sp|Q9D6Y9|GLGB_MOUSE	2	2	0	0	2	2	2	3
sp|Q8BGQ7|SYAC_MOUSE	2	2	0	0	0	0	1	2
sp|Q8BVQ5|PPME1_MOUSE	2	2	0	0	0	0	0	0
sp|Q62318|TIF1B_MOUSE	2	2	0	0	0	0	1	2
sp|Q91VK1|BZW2_MOUSE	2	2	0	0	0	0	0	0
sp|Q8BP47|SYNC_MOUSE	2	2	0	0	0	0	0	0
sp|P50516|VATA_MOUSE	2	2	0	0	0	0	0	0
sp|O35326|SRSF5_MOUSE	2	2	0	0	0	0	0	0
sp|Q9CZ04|CSN7A_MOUSE	2	2	0	0	0	0	0	0
sp|P17427|AP2A2_MOUSE	2	2	0	0	0	0	1	1

sp|Q9QZ88|VPS29_MOUSE	2	2	0	0	0	0	0	0
sp|Q9CQC6|BZW1_MOUSE	2	2	0	0	0	0	0	0
sp|Q8BP40|PPA6_MOUSE	2	2	0	0	0	0	0	0
tr|G3X8R0|G3X8R0_MOUSE	2	2	0	0	0	0	1	1
sp|Q9WTP7|KAD3_MOUSE	2	2	0	0	0	0	0	0
sp|Q9CT10|RANB3_MOUSE	2	2	0	0	0	0	0	0
sp|Q8R1I1|QCR9_MOUSE	2	2	0	0	0	0	0	0
sp|Q9CY50|SSRA_MOUSE	2	2	0	0	0	0	0	0
sp|Q9CXV1|DHSD_MOUSE	2	2	0	0	0	0	0	0
sp|Q03963|E2AK2_MOUSE	1	1	4	4	1	1	3	3
sp|Q9CQF3|CPSF5_MOUSE	1	1	3	4	0	0	1	1
sp|Q9R069|BCAM_MOUSE	1	1	3	3	2	2	3	6
sp|P35385|HSPB7_MOUSE	1	2	3	3	3	4	0	0
sp|O08788|DCTN1_MOUSE	1	1	3	3	0	0	2	2
sp|P26043|RADI_MOUSE	1	4	3	5	3	5	2	6
sp|O35841|API5_MOUSE	1	1	3	3	0	0	2	2
sp|P54823|DDX6_MOUSE	1	1	2	2	1	1	0	0
sp|Q61753|SERA_MOUSE	1	2	2	2	1	1	1	1
sp|Q9DCC8|TOM20_MOUSE	1	2	2	3	2	2	1	2
sp|Q61335|BAP31_MOUSE	1	2	2	2	2	3	0	0
sp|P10639|THIO_MOUSE	1	2	2	2	1	2	2	3
sp|Q8BJ03|COX15_MOUSE	1	1	2	3	1	1	2	3
sp|Q8JZQ9|EIF3B_MOUSE	1	2	2	3	1	2	1	1
sp|P31230|AIMP1_MOUSE	1	2	2	2	2	2	1	2
sp|Q61733|RT31_MOUSE	1	1	2	2	1	1	1	1
sp|Q99KK7|DPP3_MOUSE	1	1	2	2	2	2	1	2
sp|P60122|RUVB1_MOUSE	1	1	2	2	0	0	0	0
sp|Q9Z1P6|NDUA7_MOUSE	1	1	2	2	2	4	2	3
sp|P62814|VATB2_MOUSE	1	1	2	2	2	2	0	0
sp|P03921|NU5M_MOUSE	1	1	2	3	3	4	1	2
sp|P84099|RL19_MOUSE	1	4	2	3	2	3	2	4
sp|O35643|AP1B1_MOUSE	1	1	2	4	1	3	1	2
sp|P56565|S10A1_MOUSE	1	3	2	3	1	2	1	2
sp|Q64213|SF01_MOUSE	1	1	2	3	1	2	1	2
sp|O55142|RL35A_MOUSE	1	1	2	2	1	1	1	2
sp|O70325|GPX41_MOUSE	1	1	2	2	0	0	2	2
sp|Q9D0I9|SYRC_MOUSE	1	2	2	2	0	0	0	0
sp|Q61074|PPM1G_MOUSE	1	1	2	2	0	0	0	0
tr|Q9QUN8|Q9QUN8_MOUSE	1	37	1	20	1	30	1	28
sp|Q9D8B4|NDUAB_MOUSE	1	2	1	1	1	3	1	2
sp|Q6ZWV7|RL35_MOUSE	1	4	1	3	1	1	2	5
sp|P62983|RS27A_MOUSE	1	2	1	1	2	3	2	3
sp|Q3UX10|TBAL3_MOUSE	1	3	1	2	1	1	1	2
sp|P60766|CDC42_MOUSE	1	5	1	2	1	3	1	3
sp|P26350|PTMA_MOUSE	1	8	1	5	2	5	1	2

sp|P0C0S6|H2AZ_MOUSE	1	3	1	2	0	0	1	2
sp|Q9CZB0|C560_MOUSE	1	2	1	2	1	4	1	2
sp|P68373|TBA1C_MOUSE	1	6	1	4	1	2	1	3
tr|A2AKU9|A2AKU9_MOUSE	1	3	1	2	1	1	0	0
sp|P54731|FAF1_MOUSE	1	3	1	1	0	0	1	1
sp|P61021|RAB5B_MOUSE	1	1	1	1	0	0	1	2
sp|P16045|LEG1_MOUSE	1	2	1	1	1	1	0	0
sp|Q921Z5|TFIP8_MOUSE	1	2	1	2	0	0	1	2
sp|O88696|CLPP_MOUSE	1	1	1	3	1	2	1	1
sp|P26883|FKB1A_MOUSE	1	2	1	1	1	3	1	1
sp|Q3THS6|METK2_MOUSE	1	2	1	1	0	0	1	1
tr|D4N6R6|D4N6R6_MUSMC	1	24	1	19	1	22	1	23
sp|P53657|KPYR_MOUSE	1	2	1	2	1	2	1	2
sp|Q99MS7|EH1L1_MOUSE	1	2	1	1	0	0	1	1
sp|P22907|HEM3_MOUSE	1	2	1	1	1	1	1	2
tr|Q3UU58|Q3UU58_MOUSE	1	2	1	1	0	0	0	0
sp|B2RSH2|GNAI1_MOUSE	1	2	1	1	1	1	2	3
sp|O54931|AKAP2_MOUSE	1	1	1	1	1	1	1	1
sp|Q641P0|ARP3B_MOUSE	1	1	1	1	1	1	1	1
sp|Q9D1R9|RL34_MOUSE	1	2	1	2	1	2	1	1
sp|Q9D1L0|CHCH2_MOUSE	1	3	1	2	1	2	1	2
sp|Q9ERP3|TRI54_MOUSE	1	2	1	2	1	1	0	0
sp|A2AUC9|KLH41_MOUSE	1	1	1	1	1	1	0	0
sp|Q91WS0|CISD1_MOUSE	1	3	1	2	2	4	1	2
sp|P05063|ALDOC_MOUSE	1	1	1	1	0	0	1	2
sp|Q8R404|MIC13_MOUSE	1	3	1	2	1	3	1	4
sp|P59325|IF5_MOUSE	1	2	1	2	1	2	1	2
sp|P68372|TBB4B_MOUSE	1	1	1	1	1	1	1	1
tr|Q1WNP4|Q1WNP4_MOUSE	1	2	1	3	1	2	1	2
sp|P54775|PRS6B_MOUSE	1	3	1	2	2	4	2	5
tr|D3YW14|D3YW14_MOUSE	1	3	1	2	1	2	1	2
sp|P56375|ACYP2_MOUSE	1	2	1	1	1	2	0	0
sp|P62862|RS30_MOUSE	1	3	1	3	1	3	1	2
sp|P00342|LDHC_MOUSE	1	2	1	2	1	2	1	2
tr|A0A0S3CWK3|A0A0S3CWK3_MOUSE	1	1	1	2	1	1	1	2
tr|A0A140T8K6|A0A140T8K6_MOUSE	1	1	1	1	1	1	2	2
sp|O55135|IF6_MOUSE	1	1	1	1	0	0	1	1
sp|Q9DC07|LNEBL_MOUSE	1	2	1	1	2	2	1	1
sp|P61164|ACTZ_MOUSE	1	2	1	2	0	0	0	0
sp|Q9CQ92|FIS1_MOUSE	1	2	1	2	2	3	1	3
sp|P28667|MRP_MOUSE	1	1	1	1	1	1	1	2
sp|Q91V64|ISOC1_MOUSE	1	2	1	1	3	4	1	3
sp|P00158|CYB_MOUSE	1	1	1	1	1	1	1	1
sp|P0DN34|NDUB1_MOUSE	1	1	1	1	0	0	1	2
sp|Q9R1P0|PSA4_MOUSE	1	1	1	1	2	2	2	2

sp|Q9JII5|DAZP1_MOUSE	1	1	1	2	1	1	1	2
sp|Q9ES46|PARVB_MOUSE	1	1	1	1	1	1	1	1
sp|P17426|AP2A1_MOUSE	1	1	1	1	0	0	1	2
sp|P62627|DLRB1_MOUSE	1	1	1	1	2	3	1	2
sp|Q4KML4|ABRAL_MOUSE	1	1	1	2	1	1	1	1
sp|Q62048|PEA15_MOUSE	1	2	1	1	1	1	1	1
sp|P20108|PRDX3_MOUSE	1	1	1	1	3	3	3	3
sp|P62075|TIM13_MOUSE	1	2	1	2	1	2	2	3
sp|Q99L45|IF2B_MOUSE	1	1	1	2	2	3	3	3
sp|P46735|MYO1B_MOUSE	1	1	1	1	1	2	0	0
sp|P26516|PSMD7_MOUSE	1	1	1	2	1	1	0	0
sp|Q99K85|SERC_MOUSE	1	1	1	1	1	1	1	1
sp|Q99JW4|LIMS1_MOUSE	1	2	1	2	2	2	1	2
sp|Q91WK0|LRRF2_MOUSE	1	1	1	2	1	2	1	2
sp|O70591|PFD2_MOUSE	1	1	1	1	1	1	1	1
sp|Q8BZF8|PGM5_MOUSE	1	1	1	1	2	3	1	2
sp|O88346|TNNT1_MOUSE	1	1	1	1	1	1	2	2
sp|Q9CXT8|MPPB_MOUSE	1	1	1	2	1	1	2	2
sp|P61804|DAD1_MOUSE	1	1	1	1	1	1	0	0
sp|P60824|CIRBP_MOUSE	1	1	1	1	1	1	0	0
sp|A2AMM0|MURC_MOUSE	1	1	1	1	0	0	0	0
sp|Q8BSY0|ASPH_MOUSE	1	1	1	1	1	1	0	0
sp|P97823|LYPA1_MOUSE	1	1	1	1	0	0	0	0
sp|Q62465|VAT1_MOUSE	1	1	1	1	0	0	0	0
sp|Q60870|REEP5_MOUSE	1	3	1	1	0	0	0	0
sp|P70677|CASP3_MOUSE	1	2	1	1	0	0	0	0
sp|Q3UN02|LCLT1_MOUSE	1	1	1	1	0	0	0	0
sp|Q920E5|FPPS_MOUSE	1	2	1	1	0	0	0	0
sp|O35639|ANXA3_MOUSE	1	1	1	1	0	0	0	0
sp|Q80ZS3|RT26_MOUSE	1	1	1	1	0	0	0	0
sp|P83940|ELOC_MOUSE	1	1	1	1	0	0	0	0
tr|Q3TML0|Q3TML0_MOUSE	1	1	1	1	0	0	0	0
sp|Q9ERK4|XPO2_MOUSE	1	1	1	1	0	0	0	0
sp|Q9D6Y7|MSRA_MOUSE	1	1	1	1	0	0	0	0
tr|Q3UWL8|Q3UWL8_MOUSE	1	1	1	1	0	0	0	0
sp|Q9QYA2|TOM40_MOUSE	1	1	1	1	0	0	0	0
sp|A2AGT5|CKAP5_MOUSE	1	1	1	2	0	0	0	0
sp|Q9Z1R2|BAG6_MOUSE	1	2	1	1	0	0	0	0
sp|Q64522|H2A2B_MOUSE	1	3	1	4	1	3	1	3
tr|Q3UIJ3|Q3UIJ3_MOUSE	1	3	1	2	1	3	1	3
tr|Q9CPN9|Q9CPN9_MOUSE	1	1	1	2	1	3	1	3
sp|P63168|DYL1_MOUSE	1	1	1	2	1	2	1	3
sp|P62774|MTPN_MOUSE	1	1	1	2	1	2	1	2
sp|Q9D173|TOM7_MOUSE	1	1	1	1	1	2	1	2
sp|Q6ZWV3|RL10_MOUSE	1	1	1	2	1	2	1	1

sp|Q8CI43|MYL6B_MOUSE	1	2	1	1	1	3	1	1
sp|Q569Z6|TR150_MOUSE	1	1	1	2	1	1	1	2
sp|O08579|EMD_MOUSE	1	1	1	1	1	2	1	2
sp|Q3TBW2|RM10_MOUSE	1	1	1	2	0	0	1	1
sp|Q8VE22|RT23_MOUSE	1	1	1	3	0	0	1	2
sp|Q9EPT5|SO2A1_MOUSE	1	2	1	4	0	0	0	0
sp|Q9D0J8|PTMS_MOUSE	1	1	1	1	1	1	1	1
tr|Q678L1|Q678L1_MOUSE	1	1	1	2	1	2	0	0
sp|P57722|PCBP3_MOUSE	1	1	1	2	1	2	0	0
sp|P97427|DPYL1_MOUSE	1	1	1	2	1	2	0	0
sp|Q9JMA1|UBP14_MOUSE	1	1	1	1	1	1	1	1
sp|Q61206|PA1B2_MOUSE	1	1	1	1	1	1	0	0
sp|P62320|SMD3_MOUSE	1	1	1	1	0	0	0	0
sp|Q8K2Y7|RM47_MOUSE	1	1	1	1	0	0	1	1
sp|Q9CX56|PSMD8_MOUSE	1	1	1	1	0	0	0	0
sp|Q8BMA6|SRP68_MOUSE	1	1	1	1	0	0	0	0
sp|P53810|PIPNA_MOUSE	1	1	1	1	0	0	0	0
sp|Q9QXA5|LSM4_MOUSE	1	1	1	1	0	0	0	0
sp|Q9R000|ITBP2_MOUSE	1	2	0	0	0	0	1	1
sp|P61759|PFD3_MOUSE	1	2	0	0	1	1	1	1
sp|P08030|APT_MOUSE	1	2	0	0	1	1	1	1
sp|Q03173|ENAH_MOUSE	1	3	0	0	0	0	0	0
sp|P51807|DYLT1_MOUSE	1	3	0	0	1	2	1	1
sp|Q9CY16|RT28_MOUSE	1	1	0	0	1	1	1	1
sp|Q64105|SPRE_MOUSE	1	2	0	0	2	3	0	0
sp|Q9R1P4|PSA1_MOUSE	1	3	0	0	3	4	2	3
sp|Q7TNV0|DEK_MOUSE	1	2	0	0	0	0	1	1
sp|Q3UIL6|PKHA7_MOUSE	1	2	0	0	1	2	1	2
sp|Q64010|CRK_MOUSE	1	1	0	0	0	0	1	2
sp|Q62241|RU1C_MOUSE	1	2	0	0	0	0	1	1
sp|O70423|AOC3_MOUSE	1	2	0	0	1	1	1	1
sp|P29595|NEDD8_MOUSE	1	1	0	0	1	1	1	1
sp|P10493|NID1_MOUSE	1	1	0	0	0	0	0	0
sp|Q99LT0|DPY30_MOUSE	1	1	0	0	1	1	0	0
sp|Q99LY9|NDUS5_MOUSE	1	2	0	0	0	0	1	2
tr|B0LAA9|B0LAA9_MOUSE	1	3	0	0	0	0	1	3
sp|O88543|CSN3_MOUSE	1	2	0	0	0	0	1	1
sp|O70311|NMT2_MOUSE	1	2	0	0	0	0	1	1
tr|Q8R5L1|Q8R5L1_MOUSE	1	1	0	0	0	0	1	1
sp|Q9D967|MGDP1_MOUSE	1	2	0	0	1	1	1	1
sp|Q8K5B2|MCFD2_MOUSE	1	1	0	0	1	1	1	1
sp|P31254|UBA1Y_MOUSE	1	1	0	0	0	0	1	1
sp|P57759|ERP29_MOUSE	1	1	0	0	0	0	1	1
sp|P35278|RAB5C_MOUSE	1	1	0	0	1	2	1	1
sp|Q8K021|SCAM1_MOUSE	1	1	0	0	1	1	0	0

sp|Q920Q6|MSI2H_MOUSE	1	1	0	0	0	0	1	1
sp|O35226|PSMD4_MOUSE	1	1	0	0	1	1	1	1
sp|Q64521|GPDM_MOUSE	1	1	0	0	1	1	0	0
sp|Q8BL97|SRSF7_MOUSE	1	2	0	0	1	1	1	2
sp|Q9ER88|RT29_MOUSE	1	3	0	0	0	0	1	2
sp|Q9EPU0|RENT1_MOUSE	1	1	0	0	0	0	1	1
sp|Q80WJ7|LYRIC_MOUSE	1	1	0	0	1	1	0	0
sp|Q9WUR2|ECI2_MOUSE	1	2	0	0	2	3	2	2
sp|Q9CR00|PSMD9_MOUSE	1	1	0	0	0	0	1	1
sp|O08529|CAN2_MOUSE	1	1	0	0	1	1	1	1
tr|G3X8Y1|G3X8Y1_MOUSE	1	1	0	0	1	2	1	2
sp|Q99JI4|PSMD6_MOUSE	1	1	0	0	2	3	2	2
sp|Q8BKZ9|ODPX_MOUSE	1	1	0	0	2	2	0	0
sp|O08759|UBE3A_MOUSE	1	1	0	0	3	3	0	0
sp|Q9DBG9|TX1B3_MOUSE	1	1	0	0	2	2	1	1
sp|Q8K4Z5|SF3A1_MOUSE	1	1	0	0	0	0	2	3
sp|Q9D1A2|CNDP2_MOUSE	1	1	0	0	2	2	0	0
sp|Q9D338|RM19_MOUSE	1	2	0	0	1	1	1	1
sp|P63323|RS12_MOUSE	1	1	0	0	0	0	0	0
sp|Q9ES82|POPD2_MOUSE	1	1	0	0	0	0	2	2
sp|Q3UZA1|CPZIP_MOUSE	1	2	0	0	0	0	1	1
sp|Q6DFW4|NOP58_MOUSE	1	1	0	0	0	0	1	1
sp|Q80X19|COEA1_MOUSE	1	1	0	0	1	1	0	0
sp|P48774|GSTM5_MOUSE	1	1	0	0	1	1	0	0
sp|P19157|GSTP1_MOUSE	1	1	0	0	0	0	0	0
sp|Q9JHS4|CLPX_MOUSE	1	1	0	0	0	0	1	1
sp|P16332|MUTA_MOUSE	1	1	0	0	1	1	0	0
sp|Q791T5|MTCH1_MOUSE	1	1	0	0	0	0	0	0
tr|Q6ZWQ9|Q6ZWQ9_MOUSE	1	2	0	0	1	2	1	3
sp|Q9CPU4|MGST3_MOUSE	1	1	0	0	1	2	1	2
sp|Q80ZK0|RT10_MOUSE	1	1	0	0	0	0	1	2
tr|A2AT70|A2AT70_MOUSE	1	1	0	0	1	2	1	1
sp|Q9CQF0|RM11_MOUSE	1	1	0	0	1	1	1	1
sp|P47758|SRPRB_MOUSE	1	2	0	0	1	2	0	0
tr|B9EKP8|B9EKP8_MOUSE	1	1	0	0	0	0	0	0
sp|P03888|NU1M_MOUSE	1	1	0	0	1	1	1	1
sp|P70697|DCUP_MOUSE	1	3	0	0	0	0	0	0
sp|P24472|GSTA4_MOUSE	1	1	0	0	0	0	0	0
sp|Q9WV60|GSK3B_MOUSE	1	1	0	0	0	0	1	2
sp|Q61102|ABCB7_MOUSE	1	1	0	0	0	0	1	2
sp|P28738|KIF5C_MOUSE	1	1	0	0	1	1	1	1
sp|Q9Z172|SUMO3_MOUSE	1	1	0	0	0	0	1	2
tr|Q9D9G7|Q9D9G7_MOUSE	1	1	0	0	1	1	0	0
sp|Q9D1J3|SARNP_MOUSE	1	1	0	0	1	1	0	0
sp|Q08093|CNN2_MOUSE	1	1	0	0	0	0	1	2

sp|Q6P4T2|U520_MOUSE	1	2	0	0	0	0	0	0
sp|P63024|VAMP3_MOUSE	1	1	0	0	1	1	0	0
sp|Q99PU8|DHX30_MOUSE	1	1	0	0	0	0	0	0
sp|Q9JM14|NT5C_MOUSE	1	1	0	0	0	0	0	0
sp|Q91YY4|ATPF2_MOUSE	1	2	0	0	0	0	0	0
sp|P63030|MPC1_MOUSE	1	2	0	0	0	0	0	0
sp|P62073|TIM10_MOUSE	1	1	0	0	1	1	0	0
sp|Q8BH58|TIPRL_MOUSE	1	1	0	0	1	1	0	0
sp|P61922|GABT_MOUSE	1	1	0	0	1	1	0	0
sp|O88492|PLIN4_MOUSE	1	2	0	0	0	0	0	0
sp|Q3UIU2|NDUB6_MOUSE	1	2	0	0	0	0	0	0
sp|P39054|DYN2_MOUSE	1	1	0	0	1	1	0	0
sp|Q99KF1|TMED9_MOUSE	1	2	0	0	0	0	0	0
sp|Q8VBW6|ULA1_MOUSE	1	2	0	0	0	0	0	0
sp|P55302|AMRP_MOUSE	1	2	0	0	0	0	0	0
tr|Q6XLQ8|Q6XLQ8_MOUSE	1	2	0	0	0	0	0	0
sp|Q99KB8|GLO2_MOUSE	1	2	0	0	0	0	0	0
sp|Q9D8S9|BOLA1_MOUSE	1	2	0	0	0	0	0	0
sp|Q9EST5|AN32B_MOUSE	1	2	0	0	0	0	0	0
sp|Q3U7R1|ESYT1_MOUSE	1	1	0	0	0	0	1	1
sp|Q9JLT4|TRXR2_MOUSE	1	2	0	0	0	0	0	0
sp|Q80UM3|NAA15_MOUSE	1	2	0	0	0	0	0	0
sp|Q9WUU7|CATZ_MOUSE	1	2	0	0	0	0	0	0
sp|Q9CR86|CHSP1_MOUSE	1	2	0	0	0	0	0	0
sp|P97927|LAMA4_MOUSE	1	2	0	0	0	0	0	0
sp|Q32MW3|ACO10_MOUSE	1	1	0	0	1	1	0	0
sp|Q9CR41|HYPK_MOUSE	1	1	0	0	1	1	0	0
sp|Q6ZWM4|LSM8_MOUSE	1	2	0	0	0	0	0	0
sp|P62838|UB2D2_MOUSE	1	1	0	0	0	0	0	0
sp|Q9Z127|LAT1_MOUSE	1	1	0	0	0	0	1	1
tr|A2AV75|A2AV75_MOUSE	1	2	0	0	0	0	0	0
sp|Q9D6P8|CALL3_MOUSE	1	2	0	0	0	0	0	0
sp|Q8CHT0|AL4A1_MOUSE	1	1	0	0	0	0	0	0
sp|O08917|FLOT1_MOUSE	1	1	0	0	0	0	0	0
sp|O88746|TOM1_MOUSE	1	1	0	0	0	0	0	0
sp|P50431|GLYC_MOUSE	1	1	0	0	0	0	0	0
sp|P22892|AP1G1_MOUSE	1	1	0	0	0	0	0	0
sp|Q8R4S0|PP14C_MOUSE	1	1	0	0	0	0	0	0
sp|O35609|SCAM3_MOUSE	1	1	0	0	0	0	0	0
sp|P37040|NCPR_MOUSE	1	1	0	0	0	0	0	0
sp|Q9ET78|JPH2_MOUSE	1	1	0	0	0	0	0	0
sp|Q99JX3|GORS2_MOUSE	1	1	0	0	0	0	0	0
sp|Q60715|P4HA1_MOUSE	1	1	0	0	0	0	0	0
sp|Q9Z2M7|PMM2_MOUSE	1	1	0	0	0	0	0	0
sp|E9PZQ0|RYR1_MOUSE	1	1	0	0	0	0	0	0

sp|Q501J7|PHAR4_MOUSE	1	1	0	0	0	0	0	0
sp|Q99J99|THTM_MOUSE	1	1	0	0	0	0	0	0
sp|Q9D7X3|DUS3_MOUSE	1	1	0	0	0	0	0	0
sp|Q6GQT1|A2MG_MOUSE	1	1	0	0	0	0	0	0
sp|Q6PDL0|DC1L2_MOUSE	1	1	0	0	0	0	0	0
sp|Q9DC61|MPPA_MOUSE	1	1	0	0	0	0	0	0
sp|Q9JLZ3|AUHM_MOUSE	1	1	0	0	0	0	0	0
sp|Q8BH04|PCKGM_MOUSE	1	1	0	0	0	0	0	0
sp|Q91YP2|NEUL_MOUSE	1	1	0	0	0	0	0	0
tr|Q9ER67|Q9ER67_MOUSE	1	1	0	0	0	0	0	0
sp|Q9QZ23|NFU1_MOUSE	1	1	0	0	0	0	0	0
sp|P32233|DRG1_MOUSE	1	1	0	0	0	0	0	0
sp|Q9JMG1|EDF1_MOUSE	1	1	0	0	0	0	0	0
sp|Q8CFV9|RIFK_MOUSE	1	1	0	0	0	0	0	0
sp|Q91YR1|TWF1_MOUSE	1	1	0	0	0	0	0	0
sp|Q61035|SYHC_MOUSE	1	1	0	0	0	0	0	0
sp|Q8CBY8|DCTN4_MOUSE	1	1	0	0	0	0	0	0
sp|P62315|SMD1_MOUSE	1	1	0	0	0	0	0	0
sp|Q9CQB2|MCRI2_MOUSE	1	1	0	0	0	0	0	0
sp|Q9QUR7|PIN1_MOUSE	1	1	0	0	0	0	0	0
sp|P62500|T22D1_MOUSE	1	1	0	0	0	0	0	0
sp|Q9ESW4|AGK_MOUSE	1	1	0	0	0	0	0	0
sp|O35435|PYRD_MOUSE	1	1	0	0	0	0	0	0
sp|Q9R1T2|SAE1_MOUSE	1	1	0	0	0	0	0	0
tr|Q80U83|Q80U83_MOUSE	1	1	0	0	0	0	0	0
sp|Q9CYZ2|TPD54_MOUSE	1	1	0	0	0	0	0	0
sp|Q6P5E6|GGA2_MOUSE	1	1	0	0	0	0	0	0
tr|D3YWT1|D3YWT1_MOUSE	1	1	0	0	0	0	0	0
sp|Q8BL66|EEA1_MOUSE	1	1	0	0	0	0	0	0
sp|P62077|TIM8B_MOUSE	1	1	0	0	0	0	0	0
sp|P35235|PTN11_MOUSE	1	1	0	0	0	0	0	0
sp|P84096|RHOG_MOUSE	1	1	0	0	0	0	0	0
sp|Q9WTM5|RUVB2_MOUSE	1	1	0	0	0	0	0	0
sp|Q5HZI9|S2551_MOUSE	1	1	0	0	0	0	0	0
sp|Q62186|SSRD_MOUSE	1	1	0	0	0	0	0	0
sp|Q9DAR7|DCPS_MOUSE	1	1	0	0	0	0	0	0
sp|P27048|RSMB_MOUSE	1	1	0	0	0	0	0	0
sp|Q9CPW4|ARPC5_MOUSE	1	1	0	0	0	0	0	0
sp|Q99LB6|MAT2B_MOUSE	1	1	0	0	0	0	0	0
sp|Q91VA6|PDIP2_MOUSE	1	1	0	0	0	0	0	0
tr|A2RSX9|A2RSX9_MOUSE	1	1	0	0	0	0	0	0
sp|P97311|MCM6_MOUSE	1	1	0	0	0	0	0	0
sp|P08122|CO4A2_MOUSE	1	1	0	0	0	0	0	0
tr|A0A087WSU3|A0A087WSU3_MOUSE	1	1	0	0	0	0	0	0
sp|A2AJI0|MA7D1_MOUSE	1	1	0	0	0	0	0	0

sp|Q99K51|PLST_MOUSE	1	1	0	0	0	0	0	0
sp|G5E8K5|ANK3_MOUSE	1	1	0	0	0	0	0	0
sp|Q78IK2|USMG5_MOUSE	1	1	0	0	0	0	0	0
sp|Q9CQD1|RAB5A_MOUSE	1	1	0	0	0	0	0	0
sp|Q6PIE5|AT1A2_MOUSE	1	1	0	0	0	0	0	0
sp|Q8BVI4|DHPR_MOUSE	1	1	0	0	0	0	0	0
sp|Q8CAL5|GPC5_MOUSE	1	1	0	0	0	0	0	0
tr|E9PUM4|E9PUM4_MOUSE	1	1	0	0	0	0	0	0
sp|Q8BGD9|IF4B_MOUSE	1	1	0	0	0	0	0	0
sp|Q8JZN7|MIRO2_MOUSE	1	1	0	0	0	0	0	0
sp|P61082|UBC12_MOUSE	1	1	0	0	0	0	0	0
sp|P28658|ATX10_MOUSE	1	1	0	0	0	0	0	0
tr|B7ZNL3|B7ZNL3_MOUSE	1	1	0	0	0	0	0	0
sp|Q8BZ98|DYN3_MOUSE	1	1	0	0	0	0	0	0
sp|P11440|CDK1_MOUSE	1	1	0	0	0	0	0	0
sp|P97765|WBP2_MOUSE	1	1	0	0	0	0	0	0
sp|Q9R1Q7|PLP2_MOUSE	1	1	0	0	0	0	0	0
sp|Q9D0M1|KPRA_MOUSE	1	1	0	0	0	0	0	0
sp|B2RQC6|PYR1_MOUSE	1	1	0	0	0	0	0	0
sp|Q91W50|CSDE1_MOUSE	1	1	0	0	0	0	0	0
sp|Q6P069|SORCN_MOUSE	1	1	0	0	0	0	0	0
sp|P57784|RU2A_MOUSE	1	1	0	0	0	0	0	0
sp|Q9D0T1|NH2L1_MOUSE	1	1	0	0	0	0	0	0
sp|Q91VM5|RMXL1_MOUSE	1	1	0	0	0	0	0	0
sp|O08547|SC22B_MOUSE	1	1	0	0	0	0	0	0
sp|O88667|RAD_MOUSE	1	1	0	0	0	0	0	0
sp|P63213|GBG2_MOUSE	1	1	0	0	0	0	0	0
sp|P40336|VP26A_MOUSE	1	1	0	0	0	0	0	0
sp|Q99KK9|SYHM_MOUSE	1	1	0	0	0	0	0	0
sp|Q8CDM8|F16B1_MOUSE	1	1	0	0	0	0	0	0
tr|Q80XN6|Q80XN6_MOUSE	1	1	0	0	0	0	0	0
tr|Q7M0F4|Q7M0F4_MOUSE	1	1	0	0	0	0	0	0
sp|Q922D4|PP6R3_MOUSE	1	1	0	0	0	0	0	0
sp|Q9EQS3|MYCBP_MOUSE	1	1	0	0	0	0	0	0
sp|Q8CI08|SLAI2_MOUSE	1	1	0	0	0	0	0	0
sp|Q99N87|RT05_MOUSE	1	1	0	0	0	0	0	0
sp|Q9CWX2|CIA30_MOUSE	1	1	0	0	0	0	0	0
sp|Q8BHZ0|FA49A_MOUSE	1	1	0	0	0	0	0	0
sp|Q80UU9|PGRC2_MOUSE	1	1	0	0	0	0	0	0
sp|Q9QXN0|SHRM3_MOUSE	1	1	0	0	0	0	0	0
tr|Q8K1M3|Q8K1M3_MOUSE	1	1	0	0	0	0	0	0
sp|Q9Z2Z6|MCAT_MOUSE	1	1	0	0	0	0	0	0
sp|P56379|68MP_MOUSE	1	1	0	0	0	0	0	0
sp|Q9Z2N8|ACL6A_MOUSE	1	1	0	0	0	0	0	0
sp|P11688|ITA5_MOUSE	1	1	0	0	0	0	0	0

sp|O08582|GTPB1_MOUSE	1	1	0	0	0	0	0	0
sp|Q8CIN4|PAK2_MOUSE	1	1	0	0	0	0	0	0
sp|Q91YT7|YTHD2_MOUSE	1	1	0	0	0	0	0	0
sp|Q64310|SURF4_MOUSE	1	1	0	0	0	0	0	0
sp|Q811Q9|PCY1B_MOUSE	1	1	0	0	0	0	0	0
sp|P97429|ANXA4_MOUSE	1	1	0	0	0	0	0	0
sp|Q9CWK8|SNX2_MOUSE	1	1	0	0	0	0	0	0
sp|Q8K4G5|ABLM1_MOUSE	0	0	7	7	4	5	5	6
sp|Q5SF07|IF2B2_MOUSE	0	0	5	6	2	2	2	3
tr|Q61933|Q61933_MOUSE	0	0	2	2	0	0	4	9
sp|Q8R016|BLMH_MOUSE	0	0	2	2	2	2	0	0
tr|F8VPN4|F8VPN4_MOUSE	0	0	2	2	1	2	2	2
sp|Q9JHJ0|TMOD3_MOUSE	0	0	2	3	1	2	1	2
sp|Q921I1|TRFE_MOUSE	0	0	2	3	3	4	0	0
sp|O89079|COPE_MOUSE	0	0	2	2	3	3	2	3
sp|Q9CY27|TECR_MOUSE	0	0	2	2	2	2	2	2
tr|Q3UJB0|Q3UJB0_MOUSE	0	0	2	3	1	1	1	1
sp|Q8BTI8|SRRM2_MOUSE	0	0	2	3	0	0	1	1
sp|Q9WTX2|PRKRA_MOUSE	0	0	2	3	0	0	1	2
tr|Q6PJ18|Q6PJ18_MOUSE	0	0	2	2	1	1	1	2
sp|Q8BK30|NDUV3_MOUSE	0	0	2	3	0	0	1	2
sp|Q9JKB3|YBOX3_MOUSE	0	0	2	2	0	0	1	2
sp|Q3U0S6|RAIN_MOUSE	0	0	2	2	0	0	2	2
sp|Q6PFD9|NUP98_MOUSE	0	0	2	2	0	0	0	0
tr|Q5RJV5|Q5RJV5_MOUSE	0	0	2	3	0	0	0	0
sp|Q99LD8|DDAH2_MOUSE	0	0	2	2	0	0	0	0
sp|Q80TV8|CLAP1_MOUSE	0	0	2	2	0	0	0	0
sp|Q8K3J1|NDUS8_MOUSE	0	0	2	2	0	0	0	0
sp|Q9WV55|VAPA_MOUSE	0	0	1	1	3	5	3	4
sp|Q7TQI3|OTUB1_MOUSE	0	0	1	2	1	3	0	0
sp|Q9QZE5|COPG1_MOUSE	0	0	1	2	0	0	3	3
sp|Q9R0N0|GALK1_MOUSE	0	0	1	1	1	1	1	1
sp|Q6P542|ABCF1_MOUSE	0	0	1	2	0	0	1	1
sp|Q99NB9|SF3B1_MOUSE	0	0	1	1	0	0	2	2
sp|P84091|AP2M1_MOUSE	0	0	1	1	0	0	0	0
sp|Q7TMF3|NDUAC_MOUSE	0	0	1	2	2	2	1	1
sp|P53994|RAB2A_MOUSE	0	0	1	1	2	2	0	0
sp|Q9JJW5|MYOZ2_MOUSE	0	0	1	1	0	0	1	2
sp|Q9CZY3|UB2V1_MOUSE	0	0	1	1	0	0	0	0
sp|P28659|CELF1_MOUSE	0	0	1	1	1	1	1	1
sp|Q91V92|ACLY_MOUSE	0	0	1	1	1	1	0	0
sp|Q52KI8|SRRM1_MOUSE	0	0	1	1	1	1	0	0
sp|Q8K4L3|SVIL_MOUSE	0	0	1	1	0	0	0	0
sp|Q9JJ28|FLII_MOUSE	0	0	1	1	1	1	0	0
sp|Q9CS72|FLIP1_MOUSE	0	0	1	1	1	1	0	0

sp|Q91YM4|TBRG4_MOUSE	0	0	1	1	0	0	1	1
sp|O35295|PURB_MOUSE	0	0	1	2	1	2	0	0
sp|Q7TMM9|TBB2A_MOUSE	0	0	1	1	1	1	1	1
tr|A1E281|A1E281_MOUSE	0	0	1	3	0	0	1	2
sp|Q9D023|MPC2_MOUSE	0	0	1	1	1	1	1	3
sp|P29699|FETUA_MOUSE	0	0	1	2	1	1	1	1
sp|P56812|PDCD5_MOUSE	0	0	1	1	1	2	1	1
sp|Q9WTX5|SKP1_MOUSE	0	0	1	1	1	2	0	0
sp|Q148W8|DUS27_MOUSE	0	0	1	2	1	2	0	0
sp|O70433|FHL2_MOUSE	0	0	1	1	1	2	1	1
sp|Q3TWW8|SRSF6_MOUSE	0	0	1	1	1	1	1	1
sp|P50543|S10AB_MOUSE	0	0	1	1	0	0	1	1
sp|P63280|UBC9_MOUSE	0	0	1	2	1	1	0	0
tr|G5E8R2|G5E8R2_MOUSE	0	0	1	1	1	1	0	0
sp|P62071|RRAS2_MOUSE	0	0	1	1	1	2	0	0
sp|Q62418|DBNL_MOUSE	0	0	1	1	1	1	0	0
sp|Q8K411|PREP_MOUSE	0	0	1	1	1	2	0	0
sp|P50462|CSRP3_MOUSE	0	0	1	1	1	2	0	0
tr|E9PWE8|E9PWE8_MOUSE	0	0	1	1	0	0	1	1
sp|Q60598|SRC8_MOUSE	0	0	1	2	1	1	0	0
sp|Q91V61|SFXN3_MOUSE	0	0	1	1	1	1	0	0
sp|Q9CPQ3|TOM22_MOUSE	0	0	1	2	0	0	0	0
tr|Q5SUH6|Q5SUH6_MOUSE	0	0	1	1	0	0	1	1
sp|Q9WTQ5|AKA12_MOUSE	0	0	1	1	1	1	0	0
sp|Q6PD26|PIGS_MOUSE	0	0	1	1	0	0	1	1
sp|Q9CQU0|TXD12_MOUSE	0	0	1	2	0	0	0	0
sp|Q9CY73|RM44_MOUSE	0	0	1	2	0	0	0	0
sp|Q9CR51|VATG1_MOUSE	0	0	1	1	1	1	0	0
tr|B7ZMP8|B7ZMP8_MOUSE	0	0	1	1	0	0	1	1
sp|Q9CW03|SMC3_MOUSE	0	0	1	1	0	0	0	0
sp|Q9D892|ITPA_MOUSE	0	0	1	1	1	1	0	0
sp|Q7TS72|IP3KC_MOUSE	0	0	1	2	0	0	0	0
sp|Q99LB2|DHRS4_MOUSE	0	0	1	2	0	0	0	0
sp|Q6QD59|SEC20_MOUSE	0	0	1	1	1	1	0	0
sp|Q8BRK8|AAPK2_MOUSE	0	0	1	2	0	0	0	0
sp|O08810|U5S1_MOUSE	0	0	1	2	0	0	0	0
sp|P63254|CRIP1_MOUSE	0	0	1	2	0	0	0	0
sp|Q8BP92|RCN2_MOUSE	0	0	1	1	0	0	0	0
sp|Q9CXW3|CYBP_MOUSE	0	0	1	1	0	0	0	0
sp|Q9D883|U2AF1_MOUSE	0	0	1	1	0	0	0	0
sp|Q6ZQI3|MLEC_MOUSE	0	0	1	1	0	0	0	0
tr|E9Q740|E9Q740_MOUSE	0	0	1	1	0	0	0	0
sp|Q38HM4|TRI63_MOUSE	0	0	1	1	0	0	0	0
sp|Q99LM2|CK5P3_MOUSE	0	0	1	1	0	0	0	0
sp|Q9WVB0|RBPMS_MOUSE	0	0	1	1	0	0	0	0

sp|Q60936|COQ8A_MOUSE	0	0	1	1	0	0	0	0
tr|D3YU08|D3YU08_MOUSE	0	0	1	1	0	0	0	0
tr|F6ZGN3|F6ZGN3_MOUSE	0	0	1	1	0	0	0	0
sp|Q9CPX8|QCR10_MOUSE	0	0	1	1	0	0	0	0
sp|Q9EPL8|IPO7_MOUSE	0	0	1	1	0	0	0	0
sp|Q3UX61|NAA11_MOUSE	0	0	1	1	0	0	0	0
sp|Q9CXI0|COQ5_MOUSE	0	0	1	1	0	0	0	0
tr|Q8BML9|Q8BML9_MOUSE	0	0	1	1	0	0	0	0
sp|Q91VN4|MIC25_MOUSE	0	0	1	1	0	0	0	0
sp|Q9JIK9|RT34_MOUSE	0	0	1	1	0	0	0	0
sp|Q8BZ20|PAR12_MOUSE	0	0	1	1	0	0	0	0
tr|Q80VH1|Q80VH1_MOUSE	0	0	1	1	0	0	0	0
tr|Q3TU36|Q3TU36_MOUSE	0	0	1	1	0	0	0	0
sp|Q8R0F8|FAHD1_MOUSE	0	0	1	1	0	0	0	0
sp|Q9D7H3|RTCA_MOUSE	0	0	1	1	0	0	0	0
sp|P61202|CSN2_MOUSE	0	0	1	1	0	0	0	0
sp|P52825|CPT2_MOUSE	0	0	1	1	0	0	0	0
sp|P52912|TIA1_MOUSE	0	0	1	1	0	0	0	0
tr|G3UZ48|G3UZ48_MOUSE	0	0	1	1	0	0	0	0
sp|Q62448|IF4G2_MOUSE	0	0	1	1	0	0	0	0
sp|Q9D7S7|RL22L_MOUSE	0	0	1	1	0	0	0	0
sp|Q8BIJ6|SYIM_MOUSE	0	0	1	1	0	0	0	0
sp|Q9CR21|ACPM_MOUSE	0	0	1	1	0	0	0	0
tr|B2KF05|B2KF05_MOUSE	0	0	1	1	0	0	0	0
sp|B2RY56|RBM25_MOUSE	0	0	1	1	0	0	0	0
sp|P97352|S10AD_MOUSE	0	0	1	1	0	0	0	0
sp|Q8JZS9|RM48_MOUSE	0	0	1	1	0	0	0	0
sp|Q60649|CLPB_MOUSE	0	0	1	1	0	0	0	0
sp|P63087|PP1G_MOUSE	0	0	1	1	0	0	0	0
sp|Q6PHN9|RAB35_MOUSE	0	0	1	1	0	0	0	0
sp|Q9JKV1|ADRM1_MOUSE	0	0	1	1	0	0	0	0
sp|Q3V3R1|C1TM_MOUSE	0	0	1	1	0	0	0	0
sp|Q9D1C1|UBE2C_MOUSE	0	0	1	1	0	0	0	0
sp|P62892|RL39_MOUSE	0	0	1	1	0	0	0	0
sp|Q8K1N4|SPAS2_MOUSE	0	0	1	1	0	0	0	0
sp|Q6PEB6|PHOCN_MOUSE	0	0	1	1	0	0	0	0
tr|Q9CWF9|Q9CWF9_MOUSE	0	0	1	1	0	0	0	0
sp|Q9D8V0|HM13_MOUSE	0	0	1	1	0	0	0	0
sp|Q80U16|RIPR2_MOUSE	0	0	1	1	0	0	0	0
sp|Q8CG48|SMC2_MOUSE	0	0	1	1	0	0	0	0
sp|Q91WK2|EIF3H_MOUSE	0	0	1	1	0	0	0	0
sp|Q8BWY3|ERF1_MOUSE	0	0	0	0	3	3	3	3
sp|P24270|CATA_MOUSE	0	0	0	0	3	3	1	2
sp|Q9DCT8|CRIP2_MOUSE	0	0	0	0	3	4	0	0
sp|Q8C7E7|STBD1_MOUSE	0	0	0	0	3	3	1	1

sp|P10922|H10_MOUSE	0	0	0	0	2	4	0	0
tr|Q792Z1|Q792Z1_MOUSE	0	0	0	0	2	5	0	0
sp|Q99MR6|SRRT_MOUSE	0	0	0	0	2	3	0	0
sp|Q8BG95|MYPT2_MOUSE	0	0	0	0	2	2	1	1
sp|Q9WU78|PDC6I_MOUSE	0	0	0	0	2	2	0	0
sp|Q505F5|LRC47_MOUSE	0	0	0	0	1	1	1	1
sp|O88456|CPNS1_MOUSE	0	0	0	0	2	2	0	0
sp|Q9Z110|P5CS_MOUSE	0	0	0	0	2	2	0	0
sp|Q8R2Y8|PTH2_MOUSE	0	0	0	0	2	2	0	0
sp|P23506|PIMT_MOUSE	0	0	0	0	2	2	0	0
sp|P04919|B3AT_MOUSE	0	0	0	0	1	1	0	0
sp|Q8CCS6|PABP2_MOUSE	0	0	0	0	1	1	0	0
sp|Q9CQ91|NDUA3_MOUSE	0	0	0	0	2	2	0	0
sp|Q8C570|RAE1L_MOUSE	0	0	0	0	1	1	1	3
tr|Q8BPF4|Q8BPF4_MOUSE	0	0	0	0	1	2	1	3
sp|P63330|PP2AA_MOUSE	0	0	0	0	1	2	1	1
tr|E9PZ43|E9PZ43_MOUSE	0	0	0	0	1	2	1	1
tr|E9QQ93|E9QQ93_MOUSE	0	0	0	0	1	1	1	1
sp|Q61107|GBP4_MOUSE	0	0	0	0	1	3	0	0
sp|O35083|PLCA_MOUSE	0	0	0	0	1	1	1	1
sp|P03911|NU4M_MOUSE	0	0	0	0	1	1	1	1
sp|Q3UFY7|5NT3B_MOUSE	0	0	0	0	1	1	1	1
sp|Q8BMJ2|SYLC_MOUSE	0	0	0	0	1	1	0	0
sp|Q3UM45|PP1R7_MOUSE	0	0	0	0	1	1	1	1
sp|Q6P9R2|OXSR1_MOUSE	0	0	0	0	1	1	1	1
sp|Q9D0S9|HINT2_MOUSE	0	0	0	0	1	1	0	0
sp|Q9WVE8|PACN2_MOUSE	0	0	0	0	1	1	0	0
sp|Q9DB27|MCTS1_MOUSE	0	0	0	0	1	1	0	0
sp|Q61160|FADD_MOUSE	0	0	0	0	1	1	0	0
sp|Q8VEK0|CC50A_MOUSE	0	0	0	0	1	1	0	0
sp|P08228|SODC_MOUSE	0	0	0	0	1	1	0	0
tr|D3Z761|D3Z761_MOUSE	0	0	0	0	1	1	0	0
sp|Q9EPJ9|ARFG1_MOUSE	0	0	0	0	1	1	0	0
sp|Q7M6Y3|PICAL_MOUSE	0	0	0	0	1	1	0	0
sp|Q9JIW9|RALB_MOUSE	0	0	0	0	1	1	0	0
sp|Q6PDQ2|CHD4_MOUSE	0	0	0	0	1	1	0	0
sp|Q8VHA6|ASB18_MOUSE	0	0	0	0	0	0	1	1
sp|Q9QZM0|UBQL2_MOUSE	0	0	0	0	1	1	0	0
sp|Q9JI39|ABCBA_MOUSE	0	0	0	0	1	1	0	0
sp|Q80X50|UBP2L_MOUSE	0	0	0	0	0	0	2	3
tr|A6H5U5|A6H5U5_MOUSE	0	0	0	0	0	0	2	2
sp|Q9WV98|TIM9_MOUSE	0	0	0	0	0	0	1	2
sp|Q9WTR5|CAD13_MOUSE	0	0	0	0	1	1	0	0
sp|P28474|ADHX_MOUSE	0	0	0	0	1	1	0	0
sp|E9Q557|DESP_MOUSE	0	0	0	0	0	0	2	2

sp|P40630|TFAM_MOUSE	0	0	0	0	1	1	0	0
sp|Q9JHW2|NIT2_MOUSE	0	0	0	0	1	1	0	0
sp|Q9WTX6|CUL1_MOUSE	0	0	0	0	1	1	0	0
sp|O88307|SORL_MOUSE	0	0	0	0	0	0	1	1
sp|Q9WVA2|TIM8A_MOUSE	0	0	0	0	0	0	1	1
sp|Q9CU62|SMC1A_MOUSE	0	0	0	0	0	0	2	2
sp|Q9WV32|ARC1B_MOUSE	0	0	0	0	0	0	1	1
sp|P61620|S61A1_MOUSE	0	0	0	0	0	0	1	1
tr|Q9Z2L2|Q9Z2L2_MOUSE	0	0	0	0	1	1	0	0
tr|A0A0A6YY91|A0A0A6YY91_MOUSE	0	0	0	0	0	0	1	2
sp|P08226|APOE_MOUSE	0	0	0	0	0	0	1	1
sp|P04444|HBBZ_MOUSE	0	0	0	0	0	0	1	1
sp|Q9Z1T1|AP3B1_MOUSE	0	0	0	0	1	1	0	0
tr|Q80Y35|Q80Y35_MOUSE	0	0	0	0	1	1	0	0
sp|Q8R516|MIB2_MOUSE	0	0	0	0	0	0	1	1
tr|E9Q800|E9Q800_MOUSE	0	0	0	0	1	1	0	0
tr|Q921K2|Q921K2_MOUSE	0	0	0	0	0	0	1	1
sp|P11276|FINC_MOUSE	0	0	0	0	0	0	2	2
sp|P06467|HBAZ_MOUSE	0	0	0	0	0	0	1	1
sp|Q62095|DDX3Y_MOUSE	0	0	0	0	1	1	0	0
sp|P17918|PCNA_MOUSE	0	0	0	0	1	1	0	0
sp|Q60759|GCDH_MOUSE	0	0	0	0	1	1	0	0
sp|P97310|MCM2_MOUSE	0	0	0	0	0	0	1	1
sp|P70333|HNRH2_MOUSE	0	0	0	0	1	1	0	0
sp|O70209|PDLI3_MOUSE	0	0	0	0	0	0	1	1
sp|Q9Z2Q5|RM40_MOUSE	0	0	0	0	0	0	1	1
sp|Q61033|LAP2A_MOUSE	0	0	0	0	0	0	1	1
sp|Q61702|ITIH1_MOUSE	0	0	0	0	0	0	1	1
sp|Q61655|DD19A_MOUSE	0	0	0	0	1	1	0	0
sp|O08663|MAP2_MOUSE	0	0	0	0	0	0	1	1
sp|P10833|RRAS_MOUSE	0	0	0	0	0	0	1	1
sp|Q9JI67|B3GT5_MOUSE	0	0	0	0	0	0	1	1
sp|P30412|PPIC_MOUSE	0	0	0	0	0	0	1	1
sp|P35279|RAB6A_MOUSE	0	0	0	0	1	1	0	0
sp|Q9R0Q6|ARC1A_MOUSE	0	0	0	0	1	1	0	0
tr|Q3UHH3|Q3UHH3_MOUSE	0	0	0	0	0	0	1	1
sp|Q6P3A8|ODBB_MOUSE	0	0	0	0	0	0	1	1
sp|Q9EPC1|PARVA_MOUSE	0	0	0	0	0	0	1	1
sp|A2ADY9|DDI2_MOUSE	0	0	0	0	0	0	1	1
sp|Q9Z108|STAU1_MOUSE	0	0	0	0	0	0	1	1
sp|Q60876|4EBP1_MOUSE	0	0	0	0	0	0	1	1
sp|Q62523|ZYX_MOUSE	0	0	0	0	1	1	0	0
sp|P21619|LMNB2_MOUSE	0	0	0	0	1	1	0	0
sp|Q61187|TS101_MOUSE	0	0	0	0	1	1	0	0
tr|Q3U2G2|Q3U2G2_MOUSE	0	0	0	0	0	0	1	1

sp|Q8CGY8|OGT1_MOUSE	0	0	0	0	1	1	0	0
sp|Q3UHZ5|LMOD2_MOUSE	0	0	0	0	0	0	2	2
sp|Q9D7A8|ARMC1_MOUSE	0	0	0	0	1	1	0	0
tr|A2ADM8|A2ADM8_MOUSE	0	0	0	0	0	0	1	1
sp|P70288|HDAC2_MOUSE	0	0	0	0	0	0	1	1
sp|Q91YN9|BAG2_MOUSE	0	0	0	0	0	0	1	1
sp|Q8BKW4|ZCHC4_MOUSE	0	0	0	0	1	1	0	0
sp|Q9CWI3|BCCIP_MOUSE	0	0	0	0	0	0	1	1
sp|Q9CQL5|RM18_MOUSE	0	0	0	0	0	0	1	1
sp|Q9CZU3|SK2L2_MOUSE	0	0	0	0	1	1	0	0
sp|Q8K215|LYRM4_MOUSE	0	0	0	0	0	0	1	1
sp|Q3UW53|NIBAN_MOUSE	0	0	0	0	0	0	2	2
sp|Q922U1|PRPF3_MOUSE	0	0	0	0	1	1	0	0
sp|Q6P8X1|SNX6_MOUSE	0	0	0	0	1	1	0	0
tr|Q80ZM5|Q80ZM5_MOUSE	0	0	0	0	0	0	1	1
sp|Q3UZ39|LRRF1_MOUSE	0	0	0	0	0	0	1	1
sp|Q61387|COX7R_MOUSE	0	0	0	0	0	0	1	1
sp|Q9Z0U1|ZO2_MOUSE	0	0	0	0	1	1	0	0
sp|Q62219|TGFI1_MOUSE	0	0	0	0	0	0	1	1
sp|P84089|ERH_MOUSE	0	0	0	0	1	1	0	0
sp|Q9CXJ4|ABCB8_MOUSE	0	0	0	0	0	0	1	1
tr|Q61667|Q61667_MOUSE	0	0	0	0	1	1	0	0
sp|Q8VBT0|TMX1_MOUSE	0	0	0	0	0	0	1	1
sp|Q9CYR0|SSBP_MOUSE	0	0	0	0	0	0	1	1
sp|Q921F4|HNRLL_MOUSE	0	0	0	0	0	0	1	1
sp|P12367|KAP2_MOUSE	0	0	0	0	0	0	1	1
tr|Q8VFI8|Q8VFI8_MOUSE	0	0	0	0	1	1	0	0
sp|P50136|ODBA_MOUSE	0	0	0	0	0	0	1	1
sp|Q8BMP6|GCP60_MOUSE	0	0	0	0	0	0	1	1
sp|Q8C129|LCAP_MOUSE	0	0	0	0	0	0	1	1
sp|Q9R0P6|SC11A_MOUSE	0	0	0	0	0	0	1	1
sp|A2AN08|UBR4_MOUSE	0	0	0	0	0	0	1	1
sp|Q9CQ71|RFA3_MOUSE	0	0	0	0	0	0	1	1
sp|Q6ZPZ3|ZC3H4_MOUSE	0	0	0	0	0	0	1	1

Supplementary Table 6 Results of mass spectrometry of Set 2 RNA pull-down experiment

Note: Data in each column represented numbers of total or unique peptides that matched to each protein

Proteins	Unique CARDINAL	Total CARDINAL	Unique CARDINAL-AS	Total CARDINAL-AS	Unique Lincp21	Total Lincp21
sp|Q91Z83|MYH7_MOUSE	67	278	73	315	70	315
sp|A2ASS6|TITIN_MOUSE	21	27	33	46	25	28
sp|O55143|AT2A2_MOUSE	39	84	43	122	42	100
sp|Q99MR8|MCCA_MOUSE	34	64	36	77	37	88
sp|Q02566|MYH6_MOUSE	34	75	29	68	32	80
sp|Q99KI0|ACON_MOUSE	37	104	40	120	39	119
sp|P50544|ACADV_MOUSE	37	68	35	76	34	74
sp|E9Q4Z2|ACACB_MOUSE	25	38	18	29	28	40
sp|Q8BMS1|ECHA_MOUSE	33	73	38	97	36	85
sp|P56480|ATPB_MOUSE	38	140	37	179	38	156
sp|Q91ZA3|PCCA_MOUSE	29	54	28	56	33	86
sp|O70468|MYPC3_MOUSE	31	55	32	63	30	54
sp|Q61941|NNTM_MOUSE	26	36	35	74	33	57
sp|Q9JI91|ACTN2_MOUSE	35	86	35	67	35	74
sp|Q03265|ATPA_MOUSE	34	152	32	164	33	141
sp|Q60597|ODO1_MOUSE	23	37	30	64	26	56
sp|P70670|NACAM_MOUSE	29	50	24	37	19	26
sp|Q91VD9|NDUS1_MOUSE	27	46	27	63	29	59
sp|P63038|CH60_MOUSE	25	55	25	52	27	50
tr|E9Q616|E9Q616_MOUSE	19	22	17	23	20	26
sp|Q05920|PYC_MOUSE	24	45	21	41	26	51
sp|P41216|ACSL1_MOUSE	21	34	21	42	23	41
sp|Q99JY0|ECHB_MOUSE	26	57	25	61	24	56
sp|P05202|AATM_MOUSE	27	77	27	89	25	75
sp|P54071|IDHP_MOUSE	25	70	22	50	22	50
sp|Q3ULD5|MCCB_MOUSE	21	45	20	43	27	62
sp|Q9WUB3|PYGM_MOUSE	20	36	26	43	23	37
sp|Q7TQ48|SRCA_MOUSE	16	28	24	37	21	38
sp|P16546|SPTN1_MOUSE	13	19	19	24	15	18
sp|P38647|GRP75_MOUSE	20	35	14	27	19	37
tr|Q14BI5|Q14BI5_MOUSE	14	23	19	32	17	22
sp|Q6PB66|LPPRC_MOUSE	6	6	14	15	12	14
sp|Q99MN9|PCCB_MOUSE	17	32	21	38	24	52
sp|Q8BWT1|THIM_MOUSE	25	61	21	56	23	54
sp|P97807|FUMH_MOUSE	19	36	21	43	16	31
sp|P16858|G3P_MOUSE	20	56	19	63	25	61

sp|P21550|ENOB_MOUSE	24	59	20	41	19	35
sp|Q9CZU6|CISY_MOUSE	19	44	16	41	17	35
sp|Q8K2B3|SDHA_MOUSE	20	38	17	42	16	35
sp|P05201|AATC_MOUSE	21	49	19	44	19	45
sp|P52480|KPYM_MOUSE	21	46	17	35	17	30
sp|P20029|GRP78_MOUSE	15	24	13	23	14	28
sp|P07310|KCRM_MOUSE	21	89	22	75	20	86
sp|Q9D0K2|SCOT1_MOUSE	20	41	22	44	17	32
sp|P11499|HS90B_MOUSE	20	26	18	34	19	34
sp|P52825|CPT2_MOUSE	14	22	17	31	16	22
sp|Q8CI94|PYGB_MOUSE	12	19	18	29	11	19
sp|Q8VDN2|AT1A1_MOUSE	11	18	16	26	12	22
sp|P14824|ANXA6_MOUSE	13	18	13	19	16	23
sp|Q9DB77|QCR2_MOUSE	19	36	21	42	22	46
sp|P16125|LDHB_MOUSE	17	38	17	32	16	28
sp|P06745|G6PI_MOUSE	8	15	17	32	10	18
sp|P09411|PGK1_MOUSE	12	20	15	26	8	15
sp|Q9CQQ7|AT5F1_MOUSE	16	30	17	31	16	30
sp|Q9D0F9|PGM1_MOUSE	15	21	14	17	14	22
sp|P58252|EF2_MOUSE	10	16	11	14	13	18
sp|P04247|MYG_MOUSE	16	219	20	280	15	235
sp|P51667|MLRV_MOUSE	15	82	18	78	18	85
sp|P09542|MYL3_MOUSE	20	81	20	81	20	72
sp|P51174|ACADL_MOUSE	18	85	18	82	18	63
sp|P45952|ACADM_MOUSE	18	51	16	44	18	43
sp|Q921G7|ETFD_MOUSE	14	27	16	34	15	28
sp|P63017|HSP7C_MOUSE	15	22	15	37	13	23
sp|Q91WD5|NDUS2_MOUSE	15	24	18	37	15	26
sp|Q8CAQ8|MIC60_MOUSE	14	23	15	24	16	26
sp|O08749|DLDH_MOUSE	13	36	18	41	16	45
sp|Q9D051|ODPB_MOUSE	17	29	17	41	19	33
sp|Q8QZT1|THIL_MOUSE	12	27	13	33	13	32
sp|O35459|ECH1_MOUSE	16	30	12	24	16	31
sp|P35486|ODPA_MOUSE	12	18	14	26	12	23
sp|Q9JKS4|LDB3_MOUSE	10	21	13	22	11	20
sp|P31001|DESM_MOUSE	11	18	16	18	12	18
sp|P08249|MDHM_MOUSE	16	132	13	114	15	113
sp|P05064|ALDOA_MOUSE	15	42	14	37	14	44
sp|P48962|ADT1_MOUSE	13	23	18	46	18	38
tr|Q91VA7|Q91VA7_MOUSE	9	20	14	31	13	22
sp|P47934|CACP_MOUSE	10	14	13	27	12	23
sp|Q8BH59|CMC1_MOUSE	6	8	16	24	14	23
sp|P47857|PFKAM_MOUSE	10	12	12	15	10	16
sp|Q99LC5|ETFA_MOUSE	16	42	14	43	12	30
sp|Q9JHI5|IVD_MOUSE	13	36	15	38	16	44

sp|Q61425|HCDH_MOUSE	15	29	16	34	13	30
sp|Q9Z2I9|SUCB1_MOUSE	11	21	15	26	15	25
sp|Q99NB1|ACS2L_MOUSE	10	18	15	22	12	19
sp|Q8BMF4|ODP2_MOUSE	13	19	12	19	12	21
sp|Q9EQ20|MMSA_MOUSE	9	15	9	14	11	18
sp|Q6P8J7|KCRS_MOUSE	15	136	14	129	15	124
sp|Q9D6R2|IDH3A_MOUSE	12	38	13	34	12	38
sp|Q9CZ13|QCR1_MOUSE	14	26	13	37	15	33
sp|Q9Z0X1|AIFM1_MOUSE	13	22	11	20	15	24
sp|Q9QYR9|ACOT2_MOUSE	10	16	13	20	12	16
sp|P06151|LDHA_MOUSE	9	16	10	15	11	15
sp|Q64727|VINC_MOUSE	7	8	7	8	8	11
sp|Q8VEM8|MPCP_MOUSE	11	39	15	46	13	40
sp|P17182|ENOA_MOUSE	10	20	9	20	10	24
sp|Q9DC69|NDUA9_MOUSE	8	12	15	23	10	16
sp|Q8BFR5|EFTU_MOUSE	8	12	12	15	12	14
sp|P19783|COX41_MOUSE	10	25	12	25	10	26
sp|Q91YT0|NDUV1_MOUSE	13	26	13	25	12	23
sp|P42125|ECI1_MOUSE	10	17	13	20	10	17
sp|Q924X2|CPT1B_MOUSE	10	16	11	20	9	16
sp|Q07417|ACADS_MOUSE	12	15	14	22	11	15
sp|Q9QXX4|CMC2_MOUSE	8	13	13	22	10	14
sp|O88492|PLIN4_MOUSE	9	14	9	12	9	18
tr|B2RXT3|B2RXT3_MOUSE	8	12	9	13	6	10
sp|Q01853|TERA_MOUSE	4	8	7	12	4	5
sp|Q62234|MYOM1_MOUSE	4	4	10	14	3	4
sp|P01027|CO3_MOUSE	8	11	6	8	6	6
sp|O70250|PGAM2_MOUSE	14	41	11	34	12	43
sp|Q9D855|QCR7_MOUSE	12	36	9	23	10	35
sp|Q9DB20|ATPO_MOUSE	10	19	13	30	12	23
sp|P26443|DHE3_MOUSE	5	10	7	11	7	9
sp|P17710|HXK1_MOUSE	7	10	7	13	7	9
sp|P10649|GSTM1_MOUSE	10	21	11	61	13	54
sp|P60710|ACTB_MOUSE	11	33	11	47	9	44
sp|Q9DCX2|ATP5H_MOUSE	12	30	13	31	14	33
sp|Q9CR68|UCRI_MOUSE	11	31	11	25	9	23
sp|Q60932|VDAC1_MOUSE	10	25	12	26	10	23
sp|Q9CQ62|DECR_MOUSE	12	27	13	24	12	17
sp|O35129|PHB2_MOUSE	8	21	8	22	7	20
sp|P47738|ALDH2_MOUSE	9	15	11	24	9	14
sp|Q8BH95|ECHM_MOUSE	6	14	10	21	7	10
sp|P62259|1433E_MOUSE	11	20	8	16	8	19
sp|P34914|HYES_MOUSE	8	13	8	9	9	13
sp|Q9CR62|M2OM_MOUSE	3	6	7	12	8	10
sp|Q9R0H0|ACOX1_MOUSE	3	3	4	6	7	11

sp|Q62261|SPTB2_MOUSE	6	6	5	5	8	10
sp|Q9DCW4|ETFB_MOUSE	13	37	10	31	12	36
sp|P12787|COX5A_MOUSE	11	30	9	25	12	25
sp|P04117|FABP4_MOUSE	10	20	6	17	7	19
sp|Q99LC3|NDUAA_MOUSE	8	15	9	20	10	19
sp|P67778|PHB_MOUSE	11	21	11	14	10	18
sp|P57780|ACTN4_MOUSE	7	18	9	18	9	17
sp|Q9WUM5|SUCA_MOUSE	6	14	8	16	8	17
sp|Q9ERD7|TBB3_MOUSE	8	11	11	16	8	12
sp|Q8QZS1|HIBCH_MOUSE	10	12	9	13	11	17
sp|O54724|PTRF_MOUSE	7	15	7	10	7	16
sp|Q9ERS2|NDUAD_MOUSE	9	12	7	10	7	12
sp|Q00623|APOA1_MOUSE	5	6	9	12	8	12
sp|O08739|AMPD3_MOUSE	12	15	5	7	8	9
sp|A6X935|ITIH4_MOUSE	4	4	8	9	5	5
sp|P27546|MAP4_MOUSE	7	9	2	2	4	6
tr|F8VPN4|F8VPN4_MOUSE	4	4	5	5	4	4
sp|P14152|MDHC_MOUSE	10	48	11	30	10	28
sp|P11404|FABPH_MOUSE	9	26	9	34	9	30
sp|Q9D2G2|ODO2_MOUSE	9	17	10	26	9	15
sp|P07724|ALBU_MOUSE	6	9	12	24	7	16
sp|Q9D0M3|CY1_MOUSE	8	15	7	12	4	11
sp|Q61646|HPT_MOUSE	8	15	9	14	9	16
sp|Q8K0E8|FIBB_MOUSE	5	11	7	15	6	9
sp|Q2TPA8|HSDL2_MOUSE	6	11	8	13	6	14
sp|Q9QYG0|NDRG2_MOUSE	8	10	8	13	6	9
sp|P08113|ENPL_MOUSE	7	9	8	14	5	5
sp|P58281|OPA1_MOUSE	3	7	6	8	6	10
sp|O08528|HXK2_MOUSE	4	5	7	8	6	9
sp|P17563|SBP1_MOUSE	6	7	6	9	6	8
sp|P26041|MOES_MOUSE	7	7	5	7	5	6
sp|P09103|PDIA1_MOUSE	3	4	5	7	8	11
sp|P00405|COX2_MOUSE	7	15	11	28	7	24
sp|Q9R0Y5|KAD1_MOUSE	9	21	11	16	10	24
sp|P62897|CYC_MOUSE	7	20	8	21	7	20
sp|Q91VR2|ATPG_MOUSE	7	11	9	27	9	18
sp|P09671|SODM_MOUSE	8	18	7	17	9	24
sp|P17751|TPIS_MOUSE	8	16	7	14	7	12
tr|G5E8J6|G5E8J6_MOUSE	6	14	9	22	6	14
sp|P45376|ALDR_MOUSE	7	15	8	12	9	14
sp|Q9CQA3|SDHB_MOUSE	8	11	9	16	9	13
sp|Q8BKZ9|ODPX_MOUSE	6	12	9	13	7	10
sp|Q1XH17|TRI72_MOUSE	5	8	7	11	7	11
sp|P68369|TBA1A_MOUSE	4	5	7	10	7	14
sp|Q8JZN5|ACAD9_MOUSE	5	6	6	7	5	5

sp|E9Q401|RYR2_MOUSE	3	3	6	11	4	7
sp|Q8CGK3|LONM_MOUSE	5	6	5	5	4	4
sp|P27773|PDIA3_MOUSE	5	6	3	4	4	5
sp|Q61838|PZP_MOUSE	0	0	5	5	6	7
sp|P97450|ATP5J_MOUSE	8	27	8	21	8	20
sp|P62737|ACTA_MOUSE	8	16	9	19	9	18
sp|Q9D6J6|NDUV2_MOUSE	9	18	7	12	8	19
sp|P10126|EF1A1_MOUSE	8	19	10	21	6	10
sp|P50752|TNNT2_MOUSE	7	15	4	11	6	18
sp|Q9CPP6|NDUA5_MOUSE	6	10	5	13	5	10
sp|Q9CPQ1|COX6C_MOUSE	9	13	6	11	8	16
sp|Q8R4N0|CLYBL_MOUSE	8	14	7	10	6	13
sp|Q9DCT2|NDUS3_MOUSE	5	9	7	8	6	11
sp|P32020|NLTP_MOUSE	7	9	7	11	6	9
sp|Q921I1|TRFE_MOUSE	3	5	9	17	6	8
sp|P48678|LMNA_MOUSE	5	8	7	11	7	10
sp|Q9Z2I0|LETM1_MOUSE	4	9	6	7	4	5
sp|Q02053|UBA1_MOUSE	3	5	3	6	3	4
sp|Q60675|LAMA2_MOUSE	4	5	0	0	3	4
sp|P19536|COX5B_MOUSE	6	32	6	37	7	28
sp|P02088|HBB1_MOUSE	7	18	9	31	8	22
sp|P62631|EF1A2_MOUSE	7	32	8	25	7	33
sp|P99029|PRDX5_MOUSE	6	13	8	15	7	13
sp|Q9D172|ES1_MOUSE	6	7	8	14	7	14
sp|P70404|IDHG1_MOUSE	5	10	7	15	7	11
sp|Q9CXZ1|NDUS4_MOUSE	4	6	7	13	5	8
sp|O09161|CASQ2_MOUSE	6	10	7	10	5	10
sp|Q8CHT0|AL4A1_MOUSE	7	13	7	11	8	12
sp|O88569|ROA2_MOUSE	9	16	7	13	6	9
sp|Q9CR61|NDUB7_MOUSE	5	9	7	13	7	12
sp|Q8VIJ6|SFPQ_MOUSE	7	14	7	9	5	6
sp|Q9DCM2|GSTK1_MOUSE	5	10	6	9	7	11
sp|Q8VCM7|FIBG_MOUSE	5	7	5	9	5	6
sp|P22599|A1AT2_MOUSE	5	6	4	8	5	7
sp|O35855|BCAT2_MOUSE	4	5	7	10	5	7
sp|P51660|DHB4_MOUSE	6	9	5	7	5	8
sp|Q91VM9|IPYR2_MOUSE	4	5	5	8	3	3
sp|Q8K370|ACD10_MOUSE	5	7	6	9	3	4
sp|Q8R2G4|NAR3_MOUSE	4	6	4	6	2	2
sp|P16332|MUTA_MOUSE	3	3	6	7	4	5
sp|Q9DBL1|ACDSB_MOUSE	6	7	5	7	3	4
sp|Q91ZJ5|UGPA_MOUSE	2	3	4	6	4	6
sp|P24270|CATA_MOUSE	4	4	4	5	2	2
sp|P58771|TPM1_MOUSE	5	8	3	4	2	2
sp|Q9DBG5|PLIN3_MOUSE	3	5	4	5	3	3

tr|J3QQ16|J3QQ16_MOUSE	2	2	6	8	2	2
sp|Q3UTJ2|SRBS2_MOUSE	3	3	4	4	2	2
sp|Q8CC88|VWA8_MOUSE	3	3	3	4	4	5
sp|P21981|TGM2_MOUSE	3	3	2	2	4	4
tr|Q3UIK0|Q3UIK0_MOUSE	6	15	5	13	7	15
sp|Q61171|PRDX2_MOUSE	5	8	7	16	7	12
sp|P23927|CRYAB_MOUSE	4	9	7	14	5	11
sp|P56959|FUS_MOUSE	8	20	7	10	5	6
sp|Q78IK4|MIC27_MOUSE	5	8	4	6	7	12
sp|Q60930|VDAC2_MOUSE	6	12	6	11	6	10
sp|O08709|PRDX6_MOUSE	6	11	4	8	7	12
sp|Q91X72|HEMO_MOUSE	6	8	7	15	6	6
sp|Q64433|CH10_MOUSE	7	8	3	6	2	4
sp|Q99L13|3HIDH_MOUSE	3	5	4	6	4	6
sp|P48787|TNNI3_MOUSE	5	6	4	10	3	3
sp|P35700|PRDX1_MOUSE	5	7	6	7	6	9
sp|P50136|ODBA_MOUSE	5	8	6	7	3	3
sp|Q7TMF3|NDUAC_MOUSE	3	6	3	6	3	5
sp|Q9D6J5|NDUB8_MOUSE	0	0	5	7	5	7
sp|Q9WUR2|ECI2_MOUSE	3	5	5	8	2	4
sp|Q9CQZ5|NDUA6_MOUSE	3	5	3	5	2	3
sp|Q8BIJ6|SYIM_MOUSE	4	6	6	8	4	8
sp|O88342|WDR1_MOUSE	3	6	5	6	5	7
sp|P53395|ODB2_MOUSE	3	5	3	3	5	6
sp|P00920|CAH2_MOUSE	5	6	5	5	4	4
sp|Q8BGK2|ARHL1_MOUSE	2	2	6	8	4	6
sp|Q9JJW5|MYOZ2_MOUSE	2	2	3	4	1	1
sp|Q9WTP7|KAD3_MOUSE	1	2	3	5	3	3
sp|Q9Z2I8|SUCB2_MOUSE	4	4	4	5	6	6
sp|P35564|CALX_MOUSE	0	0	5	7	2	3
sp|P02468|LAMC1_MOUSE	2	2	5	6	3	4
sp|Q8VDD5|MYH9_MOUSE	2	2	3	3	3	4
sp|Q9JLZ3|AUHM_MOUSE	5	6	0	0	2	4
sp|Q9JHU4|DYHC1_MOUSE	2	3	4	4	0	0
sp|P01942|HBA_MOUSE	5	27	6	39	6	34
sp|O55126|NIPS2_MOUSE	3	5	6	15	4	8
sp|Q9DC70|NDUS7_MOUSE	5	9	6	12	6	7
sp|O08756|HCD2_MOUSE	6	8	6	12	5	6
sp|Q60931|VDAC3_MOUSE	4	6	4	8	5	9
sp|Q9CQH3|NDUB5_MOUSE	4	6	5	8	5	10
sp|P70296|PEBP1_MOUSE	5	10	4	9	6	9
sp|P20108|PRDX3_MOUSE	4	7	5	7	4	8
sp|Q9CRB9|MIC19_MOUSE	5	6	4	7	4	5
sp|E9PV24|FIBA_MOUSE	5	5	5	9	5	8
sp|Q9CQZ6|NDUB3_MOUSE	3	4	4	7	3	7

sp|Q08857|CD36_MOUSE	4	4	5	9	6	9
sp|Q9D8N0|EF1G_MOUSE	4	5	4	5	6	8
sp|Q99LB2|DHRS4_MOUSE	6	8	6	9	2	3
sp|P61979|HNRPK_MOUSE	3	6	5	9	3	5
sp|P20152|VIME_MOUSE	4	7	4	4	4	4
sp|Q60936|COQ8A_MOUSE	2	5	2	3	5	8
sp|Q8BMF3|MAON_MOUSE	3	5	3	6	2	3
sp|P15626|GSTM2_MOUSE	3	5	2	3	2	4
sp|Q99KQ4|NAMPT_MOUSE	2	3	5	5	3	3
sp|P03921|NU5M_MOUSE	1	1	2	3	3	3
sp|Q9CQN1|TRAP1_MOUSE	1	1	4	4	2	3
sp|Q8BGH2|SAM50_MOUSE	0	0	6	7	3	4
sp|P62806|H4_MOUSE	5	16	6	22	6	14
sp|Q8CI51|PDLI5_MOUSE	5	9	5	9	3	6
sp|Q9CQ75|NDUA2_MOUSE	5	9	3	6	3	8
sp|Q9DCS9|NDUBA_MOUSE	4	7	5	8	4	5
sp|P50171|DHB8_MOUSE	4	8	5	8	5	8
sp|P17742|PPIA_MOUSE	4	6	4	7	4	6
sp|Q61316|HSP74_MOUSE	4	9	3	5	3	5
sp|O09111|NDUBB_MOUSE	3	4	4	8	3	4
sp|Q5EBG6|HSPB6_MOUSE	3	5	3	4	3	5
sp|P18572|BASI_MOUSE	3	4	5	9	4	5
sp|Q00898|A1AT5_MOUSE	4	7	4	4	4	7
sp|Q62446|FKBP3_MOUSE	4	7	5	7	3	4
sp|Q9WTP6|KAD2_MOUSE	5	9	3	4	2	3
sp|P52503|NDUS6_MOUSE	3	4	4	7	2	5
sp|Q8K1M6|DNM1L_MOUSE	3	5	2	4	3	3
sp|P62962|PROF1_MOUSE	4	4	2	3	4	7
sp|O88441|MTX2_MOUSE	0	0	4	8	3	3
sp|Q9DBJ1|PGAM1_MOUSE	2	4	3	5	0	0
sp|P57776|EF1D_MOUSE	3	6	1	1	3	5
sp|P48036|ANXA5_MOUSE	0	0	5	8	2	2
sp|P45591|COF2_MOUSE	2	3	5	5	3	5
sp|O88844|IDHC_MOUSE	3	4	2	3	1	1
sp|Q9JHW2|NIT2_MOUSE	2	2	5	5	1	2
sp|Q9CQX8|RT36_MOUSE	0	0	4	4	0	0
sp|Q791V5|MTCH2_MOUSE	1	1	4	5	2	2
sp|Q63918|SDPR_MOUSE	2	2	2	4	4	4
sp|O09131|GSTO1_MOUSE	2	3	3	4	1	1
sp|Q8BW75|AOFB_MOUSE	1	1	4	4	3	3
sp|Q9CZ44|NSF1C_MOUSE	4	4	2	3	2	3
sp|P26040|EZRI_MOUSE	2	4	3	3	2	2
sp|Q8BG05|ROA3_MOUSE	5	10	3	4	0	0
sp|Q99K48|NONO_MOUSE	6	11	0	0	0	0
sp|Q9DCV4|RMD1_MOUSE	2	2	2	3	3	4

sp|P29758|OAT_MOUSE	1	2	0	0	3	4
sp|Q05793|PGBM_MOUSE	0	0	4	4	0	0
sp|Q60864|STIP1_MOUSE	0	0	0	0	3	4
sp|Q9CR21|ACPM_MOUSE	3	3	0	0	0	0
sp|P10630|IF4A2_MOUSE	1	1	2	3	1	1
sp|P62702|RS4X_MOUSE	1	1	0	0	1	1
sp|Q99LF4|RTCB_MOUSE	5	5	2	3	0	0
sp|Q9Z1P6|NDUA7_MOUSE	5	23	4	38	4	38
sp|Q62082|MYL10_MOUSE	5	16	5	14	5	15
sp|Q06185|ATP5I_MOUSE	3	8	5	12	4	10
sp|Q62425|NDUA4_MOUSE	3	11	5	14	3	9
sp|Q7TPR4|ACTN1_MOUSE	3	11	4	9	4	9
sp|P07356|ANXA2_MOUSE	4	10	4	6	5	8
sp|Q9JLV1|BAG3_MOUSE	4	8	5	8	2	3
sp|Q9CQC7|NDUB4_MOUSE	4	5	2	6	4	8
sp|Q91WS0|CISD1_MOUSE	3	5	3	8	3	6
sp|Q9WTR5|CAD13_MOUSE	3	7	2	6	5	6
tr|Q99N15|Q99N15_MOUSE	3	5	2	2	5	8
sp|Q8CGP1|H2B1K_MOUSE	4	4	3	4	2	3
sp|P43277|H13_MOUSE	5	5	5	5	2	2
sp|Q64105|SPRE_MOUSE	3	8	3	5	2	5
sp|P19123|TNNC1_MOUSE	3	8	1	2	3	6
sp|P24472|GSTA4_MOUSE	4	5	3	5	3	4
sp|P63158|HMGB1_MOUSE	4	6	4	7	4	7
tr|A0A0R4J0I1|A0A0R4J0I1_MOUSE	2	3	3	5	2	2
sp|Q99LX0|PARK7_MOUSE	2	3	3	6	3	3
sp|Q99LP6|GRPE1_MOUSE	4	5	4	5	3	3
sp|Q9CRB8|MTFP1_MOUSE	2	2	5	7	5	6
sp|Q9CQ54|NDUC2_MOUSE	3	4	3	5	2	4
sp|P59017|B2L13_MOUSE	1	1	5	9	3	3
sp|P14211|CALR_MOUSE	2	3	3	6	2	3
sp|O55234|PSB5_MOUSE	2	4	4	5	2	4
sp|Q9DB15|RM12_MOUSE	5	6	3	5	2	2
sp|Q04447|KCRB_MOUSE	3	7	0	0	2	5
sp|P63101|1433Z_MOUSE	0	0	3	6	1	1
sp|Q76MZ3|2AAA_MOUSE	4	6	3	3	0	0
sp|P14602|HSPB1_MOUSE	0	0	3	3	4	6
sp|P99027|RLA2_MOUSE	4	6	3	4	2	2
sp|P17156|HSP72_MOUSE	3	4	2	2	0	0
sp|Q9CPY7|AMPL_MOUSE	3	4	2	5	2	2
sp|Q8BLF1|NCEH1_MOUSE	4	6	1	2	1	1
sp|P13020|GELS_MOUSE	0	0	1	2	2	3
sp|P97443|SMYD1_MOUSE	0	0	3	3	0	0
sp|P12246|SAMP_MOUSE	3	4	2	2	3	4
sp|Q8VCT4|CES1D_MOUSE	3	4	2	3	0	0

sp|Q91WP6|SPA3N_MOUSE	1	1	2	2	2	2
sp|Q8K4G5|ABLM1_MOUSE	2	2	1	2	0	0
sp|Q8BH86|CN159_MOUSE	2	3	0	0	2	3
sp|Q9EQP2|EHD4_MOUSE	2	2	4	4	3	4
sp|Q91VR5|DDX1_MOUSE	4	5	2	2	3	4
sp|P46638|RB11B_MOUSE	2	2	2	2	2	2
sp|P50247|SAHH_MOUSE	1	2	3	4	1	1
sp|Q9D7J9|ECHD3_MOUSE	0	0	2	2	1	2
sp|P23242|CXA1_MOUSE	0	0	3	3	0	0
sp|Q99PT1|GDIR1_MOUSE	0	0	0	0	3	3
sp|Q3URD3|SLMAP_MOUSE	2	2	1	1	1	1
sp|P26039|TLN1_MOUSE	2	3	2	2	0	0
sp|P61014|PPLA_MOUSE	2	13	3	13	3	16
sp|P56135|ATPK_MOUSE	3	6	4	10	4	10
sp|Q01768|NDKB_MOUSE	3	4	3	9	3	9
sp|Q8K1Z0|COQ9_MOUSE	4	9	4	9	3	7
sp|Q9DCJ5|NDUA8_MOUSE	3	8	4	8	3	6
sp|Q9CQR4|ACO13_MOUSE	3	6	3	6	3	7
sp|P14094|AT1B1_MOUSE	2	4	4	7	3	7
sp|Q99KR7|PPIF_MOUSE	3	5	4	8	4	7
sp|Q9D6F9|TBB4A_MOUSE	2	4	4	8	3	4
sp|P46425|GSTP2_MOUSE	2	4	4	8	3	5
sp|P51881|ADT2_MOUSE	1	2	3	6	3	6
sp|Q8BWF0|SSDH_MOUSE	3	4	2	2	3	7
sp|Q149B8|PERM1_MOUSE	3	4	3	4	2	3
sp|P62960|YBOX1_MOUSE	2	4	3	3	2	3
sp|Q9CPQ8|ATP5L_MOUSE	2	4	3	6	2	2
sp|P15532|NDKA_MOUSE	3	5	4	7	4	4
sp|P61089|UBE2N_MOUSE	1	5	2	5	3	5
sp|P14206|RSSA_MOUSE	2	2	4	5	2	3
sp|Q9D023|MPC2_MOUSE	1	1	2	3	4	5
sp|O08553|DPYL2_MOUSE	2	2	3	3	2	2
sp|P68510|1433F_MOUSE	2	3	3	5	1	1
sp|Q11011|PSA_MOUSE	1	1	1	1	4	4
sp|Q9R1P4|PSA1_MOUSE	1	2	4	6	1	1
sp|P70349|HINT1_MOUSE	2	2	2	3	1	1
sp|Q99J39|DCMC_MOUSE	2	3	2	3	2	2
sp|O35857|TIM44_MOUSE	2	2	2	2	2	3
sp|P24369|PPIB_MOUSE	2	3	1	1	0	0
sp|P07759|SPA3K_MOUSE	4	5	0	0	2	3
sp|Q9EQH3|VPS35_MOUSE	1	2	2	2	0	0
sp|Q8BZF8|PGM5_MOUSE	1	1	2	3	2	3
sp|Q9CZM2|RL15_MOUSE	0	0	2	3	0	0
sp|Q9WVK4|EHD1_MOUSE	1	2	2	2	1	1
sp|P12970|RL7A_MOUSE	0	0	2	3	2	2

sp|P80314|TCPB_MOUSE	2	2	2	2	1	1
sp|Q9WVA4|TAGL2_MOUSE	2	3	0	0	1	2
sp|P47963|RL13_MOUSE	2	2	2	2	1	1
sp|Q9JLT4|TRXR2_MOUSE	1	1	1	1	2	3
sp|P68040|RACK1_MOUSE	2	4	2	2	0	0
sp|Q68FD5|CLH1_MOUSE	0	0	4	5	1	1
sp|Q3UIU2|NDUB6_MOUSE	0	0	4	5	0	0
sp|O88587|COMT_MOUSE	2	2	2	2	2	2
sp|P58774|TPM2_MOUSE	2	2	1	2	2	2
sp|P10493|NID1_MOUSE	0	0	2	2	0	0
sp|P08226|APOE_MOUSE	0	0	1	1	2	2
sp|Q9JK42|PDK2_MOUSE	0	0	3	3	0	0
sp|P35980|RL18_MOUSE	0	0	3	4	0	0
sp|P61982|1433G_MOUSE	1	1	2	2	1	1
sp|Q9CZX8|RS19_MOUSE	2	3	1	1	2	2
sp|Q924D0|RT4I1_MOUSE	0	0	1	1	2	3
sp|Q91VT4|CBR4_MOUSE	0	0	0	0	1	1
sp|P06728|APOA4_MOUSE	1	1	0	0	0	0
sp|P62082|RS7_MOUSE	3	4	0	0	0	0
sp|Q9D3D9|ATPD_MOUSE	2	20	2	13	3	10
sp|P56392|CX7A1_MOUSE	3	14	3	8	3	10
sp|P56391|CX6B1_MOUSE	2	7	3	9	2	10
sp|C0HKE3|H2A1D_MOUSE	3	3	3	9	2	5
tr|A8DUK4|A8DUK4_MOUSE	2	3	2	8	3	4
sp|P48771|CX7A2_MOUSE	2	5	3	6	2	6
sp|P68368|TBA4A_MOUSE	2	3	3	7	3	5
sp|P03930|ATP8_MOUSE	3	5	2	6	3	4
sp|P99028|QCR6_MOUSE	2	4	2	6	2	6
sp|Q9R0P3|ESTD_MOUSE	2	5	2	4	2	4
sp|Q922B1|MACD1_MOUSE	2	4	2	2	2	4
sp|O35658|C1QBP_MOUSE	2	3	3	5	3	7
sp|P17879|HS71B_MOUSE	1	3	2	5	2	3
sp|Q02013|AQP1_MOUSE	2	3	3	3	2	4
sp|P03911|NU4M_MOUSE	3	5	2	2	3	8
sp|A2AMM0|MURC_MOUSE	2	3	2	5	1	2
sp|P08228|SODC_MOUSE	1	3	3	3	2	4
sp|Q9WV27|AT1A4_MOUSE	2	3	2	2	3	4
sp|Q922R8|PDIA6_MOUSE	1	2	1	3	2	2
sp|Q9CPU4|MGST3_MOUSE	0	0	2	5	2	2
sp|Q93092|TALDO_MOUSE	2	4	1	1	2	5
sp|Q78ZA7|NP1L4_MOUSE	2	3	1	2	1	3
sp|P62075|TIM13_MOUSE	2	2	2	4	2	2
sp|Q9DCZ4|MIC26_MOUSE	1	2	2	3	0	0
sp|P11031|TCP4_MOUSE	2	3	2	3	2	2
sp|P62827|RAN_MOUSE	2	2	3	4	2	4

sp|O70622|RTN2_MOUSE	1	1	2	3	1	1
sp|Q60605|MYL6_MOUSE	3	4	0	0	0	0
sp|Q91Z53|GRHPR_MOUSE	3	4	2	2	1	1
sp|Q01730|RSU1_MOUSE	2	3	1	1	2	2
sp|Q9CQC9|SAR1B_MOUSE	1	1	2	4	0	0
sp|Q61598|GDIB_MOUSE	2	2	2	2	2	3
sp|Q9DC61|MPPA_MOUSE	0	0	1	1	1	1
tr|D3Z7X0|D3Z7X0_MOUSE	1	1	0	0	2	5
sp|P09055|ITB1_MOUSE	2	2	0	0	2	3
sp|Q9CPV4|GLOD4_MOUSE	2	3	2	3	2	2
sp|Q8K3J1|NDUS8_MOUSE	0	0	2	2	0	0
sp|Q8CGC7|SYEP_MOUSE	2	3	2	3	2	2
sp|P08752|GNAI2_MOUSE	0	0	2	3	1	1
sp|Q9DCN2|NB5R3_MOUSE	1	1	1	1	1	1
sp|P50580|PA2G4_MOUSE	2	2	2	2	1	2
sp|P09405|NUCL_MOUSE	3	6	0	0	1	2
sp|Q9QUM9|PSA6_MOUSE	3	4	1	1	1	1
sp|P62264|RS14_MOUSE	0	0	1	1	0	0
sp|P62242|RS8_MOUSE	0	0	1	1	1	1
sp|Q9CXV1|DHSD_MOUSE	0	0	2	2	3	4
sp|Q9R1P3|PSB2_MOUSE	2	2	2	2	2	2
sp|P30275|KCRU_MOUSE	1	2	1	1	2	2
sp|Q99LY9|NDUS5_MOUSE	2	3	1	1	2	3
tr|Q792Z1|Q792Z1_MOUSE	1	1	1	2	3	4
sp|Q9CXT8|MPPB_MOUSE	0	0	2	3	2	4
sp|Q9CQ60|6PGL_MOUSE	0	0	1	2	1	1
sp|P62908|RS3_MOUSE	0	0	1	1	2	2
sp|P23953|EST1C_MOUSE	2	2	1	1	2	3
sp|Q8R081|HNRPL_MOUSE	0	0	3	6	2	2
sp|P47911|RL6_MOUSE	0	0	2	3	1	1
sp|P28665|MUG1_MOUSE	1	2	2	2	1	2
sp|Q9Z2U0|PSA7_MOUSE	2	2	2	2	1	1
sp|P62918|RL8_MOUSE	0	0	1	1	2	2
sp|Q8R0F8|FAHD1_MOUSE	0	0	1	2	1	2
tr|Q60638|Q60638_MOUSE	2	4	0	0	0	0
sp|Q00896|A1AT3_MOUSE	2	3	0	0	0	0
sp|Q9Z2U1|PSA5_MOUSE	2	2	3	4	1	1
sp|P63242|IF5A1_MOUSE	0	0	1	1	0	0
sp|Q9ET78|JPH2_MOUSE	1	1	2	2	2	3
sp|Q9CXJ4|ABCB8_MOUSE	2	2	1	2	1	1
sp|Q8VEK3|HNRPU_MOUSE	2	2	2	2	0	0
sp|Q00897|A1AT4_MOUSE	1	2	0	0	0	0
sp|Q8VD26|TM143_MOUSE	0	0	3	4	0	0
sp|Q9D6K8|FUND2_MOUSE	0	0	1	1	0	0
sp|Q8BP40|PPA6_MOUSE	1	1	1	1	1	1

sp|P82347|SGCD_MOUSE	0	0	2	2	0	0
sp|Q3ULJ0|GPD1L_MOUSE	1	2	0	0	3	3
sp|P14733|LMNB1_MOUSE	0	0	2	3	0	0
sp|P55264|ADK_MOUSE	2	2	0	0	2	3
sp|Q8JZQ2|AFG32_MOUSE	1	1	0	0	2	2
sp|Q6PDM2|SRSF1_MOUSE	2	3	1	1	0	0
sp|P26231|CTNA1_MOUSE	1	1	0	0	0	0
sp|P16045|LEG1_MOUSE	1	1	0	0	0	0
sp|Q9CYT6|CAP2_MOUSE	1	1	1	1	1	1
tr|Q3UHH3|Q3UHH3_MOUSE	0	0	0	0	1	2
sp|O08532|CA2D1_MOUSE	1	1	1	1	1	1
sp|P80318|TCPG_MOUSE	2	3	0	0	1	1
sp|Q8BWM0|PGES2_MOUSE	0	0	0	0	2	2
sp|P05132|KAPCA_MOUSE	0	0	0	0	2	2
sp|Q99JR6|NMNA3_MOUSE	3	3	0	0	1	1
sp|Q9WVE8|PACN2_MOUSE	1	1	1	2	0	0
sp|P61161|ARP2_MOUSE	1	1	2	2	1	1
sp|P82349|SGCB_MOUSE	0	0	1	1	0	0
sp|Q99KJ8|DCTN2_MOUSE	1	1	0	0	0	0
sp|Q8R326|PSPC1_MOUSE	3	3	0	0	0	0
sp|Q8BSL7|ARF2_MOUSE	0	0	1	1	1	1
sp|O35943|FRDA_MOUSE	1	1	0	0	2	2
sp|Q91Z31|PTBP2_MOUSE	0	0	3	3	0	0
sp|O09061|PSB1_MOUSE	2	2	0	0	0	0
sp|P08003|PDIA4_MOUSE	0	0	0	0	2	2
sp|P11531|DMD_MOUSE	2	2	0	0	0	0
sp|Q91VC9|GHITM_MOUSE	0	0	3	3	0	0
sp|Q8BK30|NDUV3_MOUSE	2	10	2	6	2	7
sp|Q9CWF2|TBB2B_MOUSE	2	4	2	6	2	4
sp|P50462|CSRP3_MOUSE	2	3	2	5	1	3
sp|P85094|ISC2A_MOUSE	2	2	2	2	2	5
sp|P84104|SRSF3_MOUSE	2	5	2	4	1	3
sp|P56565|S10A1_MOUSE	2	4	2	3	2	3
sp|Q9DCT8|CRIP2_MOUSE	2	4	0	0	2	5
sp|P10639|THIO_MOUSE	2	5	2	3	2	3
tr|A0A0R4J083|A0A0R4J083_MOUSE	0	0	2	4	2	6
sp|P62983|RS27A_MOUSE	2	2	2	4	2	3
sp|P56382|ATP5E_MOUSE	2	5	2	4	2	2
sp|Q9D1L0|CHCH2_MOUSE	1	3	1	2	2	4
sp|O88696|CLPP_MOUSE	2	4	1	1	2	3
sp|Q9CQ69|QCR8_MOUSE	1	2	2	3	0	0
sp|P29699|FETUA_MOUSE	2	3	1	2	2	4
sp|Q9D1I5|MCEE_MOUSE	0	0	2	4	0	0
sp|Q9D0S9|HINT2_MOUSE	1	1	2	3	2	3
sp|Q9CQV8|1433B_MOUSE	1	1	2	3	2	2

sp|Q9CZB0|C560_MOUSE	1	1	2	4	2	3
tr|Q91VB8|Q91VB8_MOUSE	1	1	2	3	1	3
sp|Q64737|PUR2_MOUSE	0	0	1	3	1	2
sp|O35643|AP1B1_MOUSE	1	1	1	1	2	3
sp|Q9CQJ8|NDUB9_MOUSE	2	3	0	0	2	3
sp|P08122|CO4A2_MOUSE	2	4	2	3	1	1
sp|P17665|COX7C_MOUSE	2	4	2	3	0	0
sp|P62874|GBB1_MOUSE	0	0	1	3	1	2
sp|Q9EQI8|RM46_MOUSE	1	1	1	3	0	0
sp|P38060|HMGCL_MOUSE	0	0	2	3	1	1
sp|Q6P3A8|ODBB_MOUSE	1	2	2	3	1	1
sp|P60867|RS20_MOUSE	2	4	1	1	2	3
sp|P53986|MOT1_MOUSE	2	4	2	4	0	0
sp|Q8BMS4|COQ3_MOUSE	0	0	2	4	2	2
sp|Q9DCC8|TOM20_MOUSE	1	3	2	4	1	1
sp|Q8CG76|ARK72_MOUSE	2	3	1	3	1	2
sp|Q61820|RANT_MOUSE	1	1	1	3	1	2
sp|P43276|H15_MOUSE	2	2	2	3	1	2
sp|Q60692|PSB6_MOUSE	1	3	2	3	1	1
sp|P52196|THTR_MOUSE	0	0	1	2	1	2
sp|Q9D6U8|F162A_MOUSE	2	5	2	2	0	0
sp|Q8VE95|CH082_MOUSE	0	0	1	2	1	2
sp|P62270|RS18_MOUSE	1	1	1	2	1	1
sp|P97823|LYPA1_MOUSE	1	1	1	2	0	0
sp|O08807|PRDX4_MOUSE	0	0	2	3	0	0
sp|P62751|RL23A_MOUSE	1	2	2	3	0	0
sp|Q80X76|SPA3F_MOUSE	2	2	0	0	2	3
sp|P62137|PP1A_MOUSE	1	1	2	2	1	1
sp|Q62093|SRSF2_MOUSE	1	1	2	4	0	0
sp|O70435|PSA3_MOUSE	1	2	2	3	1	1
sp|Q6ZWX6|IF2A_MOUSE	2	3	1	1	1	1
sp|Q9CQM9|GLRX3_MOUSE	1	1	1	2	1	1
sp|P62858|RS28_MOUSE	2	3	2	2	0	0
sp|Q924M7|MPI_MOUSE	2	3	1	2	2	2
sp|Q9D6Y7|MSRA_MOUSE	1	1	1	2	2	2
sp|P61358|RL27_MOUSE	1	2	2	3	0	0
sp|Q8CI43|MYL6B_MOUSE	2	3	1	1	1	2
sp|P19253|RL13A_MOUSE	1	2	0	0	2	3
sp|Q80Y14|GLRX5_MOUSE	1	2	2	2	1	2
sp|P97371|PSME1_MOUSE	0	0	1	1	0	0
sp|Q8R1G2|CMBL_MOUSE	0	0	1	1	1	1
sp|Q9DCD0|6PGD_MOUSE	0	0	1	1	1	1
sp|Q60668|HNRPD_MOUSE	1	1	2	2	0	0
sp|Q9CRD0|OCAD1_MOUSE	0	0	1	1	0	0
sp|Q61545|EWS_MOUSE	1	1	1	1	1	2

sp|O88487|DC1I2_MOUSE	1	1	1	1	0	0
sp|Q922B2|SYDC_MOUSE	1	1	0	0	1	2
sp|P60843|IF4A1_MOUSE	0	0	0	0	1	1
tr|G3X8R0|G3X8R0_MOUSE	1	1	0	0	1	1
sp|Q8BL97|SRSF7_MOUSE	2	2	1	2	1	1
sp|P54822|PUR8_MOUSE	1	2	2	2	1	1
sp|P97372|PSME2_MOUSE	0	0	1	2	1	2
sp|P13707|GPDA_MOUSE	1	2	1	1	2	2
sp|P37804|TAGL_MOUSE	1	1	0	0	1	3
sp|Q62376|RU17_MOUSE	2	3	0	0	1	1
sp|Q921H8|THIKA_MOUSE	1	2	0	0	0	0
sp|Q5SW19|CLU_MOUSE	1	1	0	0	2	3
sp|P56399|UBP5_MOUSE	1	1	0	0	1	2
sp|O70251|EF1B_MOUSE	0	0	1	1	0	0
sp|Q60865|CAPR1_MOUSE	2	2	0	0	1	1
sp|Q9CPX8|QCR10_MOUSE	1	2	1	1	1	1
sp|Q9D6S7|RRFM_MOUSE	1	1	0	0	2	2
sp|Q9R062|GLYG_MOUSE	2	4	0	0	1	1
sp|Q9CXI0|COQ5_MOUSE	0	0	0	0	2	2
sp|Q9Z1E4|GYS1_MOUSE	0	0	2	2	0	0
sp|Q9DCB8|ISCA2_MOUSE	0	0	1	1	1	1
sp|P23506|PIMT_MOUSE	0	0	1	1	0	0
sp|Q9CQ91|NDUA3_MOUSE	0	0	1	1	2	2
sp|P49722|PSA2_MOUSE	0	0	1	1	2	2
sp|Q9Z1W8|AT12A_MOUSE	2	3	0	0	0	0
sp|P19157|GSTP1_MOUSE	0	0	1	1	1	1
sp|Q9Z2Y8|PLPHP_MOUSE	0	0	1	1	0	0
sp|Q9R069|BCAM_MOUSE	1	1	1	2	1	1
sp|A2AGL3|RYR3_MOUSE	0	0	1	1	2	2
sp|P36536|SAR1A_MOUSE	0	0	0	0	2	2
sp|O88685|PRS6A_MOUSE	0	0	1	1	0	0
sp|P62245|RS15A_MOUSE	2	2	0	0	0	0
sp|P47791|GSHR_MOUSE	1	1	0	0	1	1
sp|P63030|MPC1_MOUSE	1	2	1	1	0	0
sp|Q9QX60|DGUOK_MOUSE	0	0	2	2	0	0
sp|Q78IK2|USMG5_MOUSE	0	0	1	1	0	0
sp|P80315|TCPD_MOUSE	1	1	2	2	0	0
sp|Q5FW52|MLIP_MOUSE	0	0	1	1	0	0
sp|Q8C7H1|MMAA_MOUSE	0	0	2	2	1	1
sp|Q9R0P5|DEST_MOUSE	0	0	2	2	0	0
sp|Q91W97|HKDC1_MOUSE	0	0	2	3	0	0
sp|P56395|CYB5_MOUSE	2	3	0	0	0	0
sp|P68433|H31_MOUSE	2	2	0	0	0	0
sp|P62754|RS6_MOUSE	1	1	0	0	1	1
sp|P80317|TCPZ_MOUSE	1	1	0	0	0	0

sp|P28650|PURA1_MOUSE	0	0	1	1	1	1
sp|Q99PR8|HSPB2_MOUSE	2	3	0	0	0	0
sp|Q9D8E6|RL4_MOUSE	1	1	0	0	1	1
sp|Q7TPW1|NEXN_MOUSE	0	0	1	1	0	0
sp|P10107|ANXA1_MOUSE	1	1	0	0	0	0
sp|P63005|LIS1_MOUSE	0	0	2	2	0	0
sp|Q3THE2|ML12B_MOUSE	0	0	1	1	1	1
sp|Q7TMK9|HNRPQ_MOUSE	1	1	1	2	0	0
sp|P0DP27|CALM2_MOUSE	2	2	0	0	1	1
sp|Q99JI4|PSMD6_MOUSE	0	0	0	0	1	1
sp|Q02357|ANK1_MOUSE	1	2	1	1	0	0
sp|Q9Z2M7|PMM2_MOUSE	0	0	1	1	1	1
sp|P99026|PSB4_MOUSE	1	1	0	0	0	0
sp|Q9D0G0|RT30_MOUSE	0	0	2	2	0	0
sp|Q64669|NQO1_MOUSE	1	1	0	0	0	0
sp|P07901|HS90A_MOUSE	0	0	1	1	0	0
sp|P97351|RS3A_MOUSE	1	1	0	0	2	2
sp|Q99MN1|SYK_MOUSE	2	3	0	0	0	0
sp|Q9CQN6|TM14C_MOUSE	0	0	1	1	0	0
sp|P02469|LAMB1_MOUSE	0	0	2	2	0	0
sp|Q3TJZ6|FA98A_MOUSE	2	2	0	0	0	0
sp|Q99JY9|ARP3_MOUSE	1	1	0	0	0	0
sp|Q99L47|F10A1_MOUSE	1	1	0	0	1	1
sp|P26369|U2AF2_MOUSE	0	0	1	1	0	0
sp|P62835|RAP1A_MOUSE	0	0	0	0	2	2
sp|O70325|GPX41_MOUSE	1	1	0	0	0	0
sp|Q8BTY1|KAT1_MOUSE	2	2	0	0	0	0
sp|Q9R1P0|PSA4_MOUSE	0	0	0	0	2	2
sp|P42932|TCPQ_MOUSE	2	2	0	0	0	0
sp|P11983|TCPA_MOUSE	2	2	0	0	0	0
sp|P22315|HEMH_MOUSE	0	0	0	0	2	2
sp|Q00519|XDH_MOUSE	1	1	1	1	0	0
sp|Q8VBT1|TXLNB_MOUSE	0	0	0	0	2	2
tr|Q9QUN8|Q9QUN8_MOUSE	1	58	1	34	1	34
tr|D4N6R6|D4N6R6_MUSMC	1	27	1	32	1	27
sp|Q9CR84|AT5G1_MOUSE	0	0	1	6	1	1
tr|Q9CPN9|Q9CPN9_MOUSE	1	3	1	2	1	4
sp|P56379|68MP_MOUSE	1	1	1	3	1	1
sp|P26645|MARCS_MOUSE	1	2	1	3	1	2
sp|Q9D8B4|NDUAB_MOUSE	1	3	1	2	1	2
sp|P56375|ACYP2_MOUSE	1	1	1	3	1	3
sp|P68372|TBB4B_MOUSE	1	2	1	2	1	2
sp|P43274|H14_MOUSE	1	3	1	1	1	2
sp|Q99KB8|GLO2_MOUSE	1	2	1	2	1	2
sp|P53657|KPYR_MOUSE	1	2	1	2	1	2

sp|Q60590|A1AG1_MOUSE	1	3	1	3	0	0
tr|A2AKU9|A2AKU9_MOUSE	1	1	1	3	1	1
sp|O88456|CPNS1_MOUSE	1	2	1	2	1	1
sp|P00158|CYB_MOUSE	0	0	1	2	1	2
sp|Q9CQ92|FIS1_MOUSE	1	2	1	2	1	2
sp|P99024|TBB5_MOUSE	1	2	1	1	0	0
sp|Q9Z239|PLM_MOUSE	1	3	1	2	1	2
sp|Q9DBB8|DHDH_MOUSE	0	0	1	3	1	1
sp|Q99020|ROAA_MOUSE	1	1	1	3	0	0
sp|P18760|COF1_MOUSE	1	1	1	2	1	2
sp|P0DN34|NDUB1_MOUSE	1	2	1	2	1	1
tr|B1Q450|B1Q450_MOUSE	1	3	0	0	0	0
sp|P62862|RS30_MOUSE	1	1	1	2	1	2
sp|Q9D1G3|HHATL_MOUSE	0	0	1	2	1	3
sp|O70433|FHL2_MOUSE	1	2	1	2	0	0
sp|P14115|RL27A_MOUSE	1	2	1	2	0	0
sp|P26883|FKB1A_MOUSE	1	1	1	1	1	2
sp|Q9CXW4|RL11_MOUSE	1	2	1	1	0	0
sp|P97447|FHL1_MOUSE	1	1	1	2	1	2
sp|Q9ET01|PYGL_MOUSE	0	0	1	2	1	1
KV2A7_MOUSE||	1	2	0	0	1	3
sp|Q8C0M9|ASGL1_MOUSE	0	0	1	2	0	0
sp|Q9WU78|PDC6I_MOUSE	0	0	1	2	1	1
sp|P15105|GLNA_MOUSE	0	0	1	1	0	0
sp|Q7TMM9|TBB2A_MOUSE	1	2	1	1	1	1
sp|Q9D0J8|PTMS_MOUSE	1	1	1	1	1	1
sp|P67984|RL22_MOUSE	1	3	1	1	0	0
sp|P13541|MYH3_MOUSE	0	0	1	1	1	1
sp|Q9JKL4|NDUF3_MOUSE	0	0	1	2	0	0
sp|P00342|LDHC_MOUSE	1	2	0	0	1	1
sp|P62900|RL31_MOUSE	0	0	1	1	1	1
sp|P21300|ALD1_MOUSE	0	0	1	1	0	0
sp|Q6P069|SORCN_MOUSE	1	2	0	0	1	1
sp|Q6PIU9|YJ005_MOUSE	1	3	1	1	1	1
sp|Q6ZWV7|RL35_MOUSE	1	1	1	1	1	2
sp|Q9D819|IPYR_MOUSE	1	2	1	1	1	1
sp|Q64522|H2A2B_MOUSE	0	0	1	1	1	1
sp|Q91V64|ISOC1_MOUSE	0	0	0	0	1	2
sp|Q9DC77|SMPX_MOUSE	0	0	1	1	1	1
sp|Q6W8Q3|PC4L1_MOUSE	1	2	0	0	1	2
sp|P54775|PRS6B_MOUSE	0	0	1	2	0	0
sp|P63325|RS10_MOUSE	1	2	1	1	0	0
sp|Q9D1X0|NOL3_MOUSE	1	1	1	2	0	0
sp|Q9JJL8|SYSM_MOUSE	0	0	1	2	0	0
sp|Q8VBT0|TMX1_MOUSE	0	0	0	0	1	1

sp|Q8R1B4|EIF3C_MOUSE	0	0	1	1	0	0
sp|Q6P1F6|2ABA_MOUSE	0	0	1	1	0	0
sp|Q9CQV1|TIM16_MOUSE	1	2	1	1	0	0
sp|Q9CQF3|CPSF5_MOUSE	1	1	1	2	1	1
sp|Q3UV70|PDP1_MOUSE	0	0	0	0	1	2
sp|Q8VE22|RT23_MOUSE	0	0	1	2	1	1
sp|Q9Z172|SUMO3_MOUSE	1	1	1	1	0	0
sp|P28738|KIF5C_MOUSE	0	0	0	0	1	1
sp|P63168|DYL1_MOUSE	1	1	1	1	1	1
sp|O08677|KNG1_MOUSE	1	1	1	1	0	0
sp|P51859|HDGF_MOUSE	0	0	1	1	1	1
tr|Q9CR06|Q9CR06_MOUSE	0	0	1	1	1	1
sp|P26043|RADI_MOUSE	1	1	0	0	1	2
sp|Q61990|PCBP2_MOUSE	0	0	1	2	1	1
sp|P68037|UB2L3_MOUSE	0	0	1	2	0	0
sp|Q9WTX5|SKP1_MOUSE	0	0	1	2	0	0
tr|Q91XL1|Q91XL1_MOUSE	0	0	1	1	1	1
sp|Q61595|KTN1_MOUSE	1	1	1	1	0	0
tr|Q678L1|Q678L1_MOUSE	0	0	1	1	1	1
sp|P49443|PPM1A_MOUSE	1	2	0	0	1	1
tr|Q8R5L1|Q8R5L1_MOUSE	1	2	0	0	1	1
sp|Q02788|CO6A2_MOUSE	1	1	1	1	0	0
sp|Q9DAK9|PHP14_MOUSE	0	0	1	1	0	0
sp|O55023|IMPA1_MOUSE	0	0	1	1	0	0
tr|Q3TFD0|Q3TFD0_MOUSE	1	1	0	0	0	0
sp|P59999|ARPC4_MOUSE	1	1	1	1	0	0
tr|D3Z494|D3Z494_MOUSE	0	0	0	0	1	1
sp|P15331|PERI_MOUSE	0	0	1	1	1	1
sp|P62852|RS25_MOUSE	1	1	1	1	0	0
sp|P46656|ADX_MOUSE	0	0	1	1	0	0
sp|Q4VAE3|TMM65_MOUSE	0	0	1	1	0	0
tr|F8WGM2|F8WGM2_MOUSE	0	0	1	2	0	0
tr|E9Q3H6|E9Q3H6_MOUSE	1	1	0	0	0	0
sp|O08716|FABP9_MOUSE	1	3	0	0	0	0
sp|Q8R404|MIC13_MOUSE	1	1	0	0	0	0
sp|P18242|CATD_MOUSE	0	0	1	1	0	0
sp|P68254|1433T_MOUSE	0	0	1	1	0	0
sp|P62821|RAB1A_MOUSE	0	0	0	0	1	1
sp|A2AUC9|KLH41_MOUSE	0	0	1	1	0	0
sp|Q9CR57|RL14_MOUSE	0	0	0	0	1	1
sp|Q9CYR0|SSBP_MOUSE	0	0	0	0	1	1
sp|Q8BGC4|PTGR3_MOUSE	0	0	1	1	0	0
sp|Q9D8W5|PSD12_MOUSE	0	0	1	1	0	0
sp|Q9DCZ1|GMPR1_MOUSE	1	1	0	0	0	0
sp|P35762|CD81_MOUSE	1	2	0	0	0	0

sp|Q9CY58|PAIRB_MOUSE	0	0	1	1	0	0
sp|Q8K310|MATR3_MOUSE	0	0	1	1	0	0
sp|Q9D880|TIM50_MOUSE	0	0	1	1	1	1
sp|Q9ER88|RT29_MOUSE	0	0	1	1	0	0
sp|Q9D1A2|CNDP2_MOUSE	0	0	1	1	1	1
sp|Q5SWU9|ACACA_MOUSE	1	2	0	0	0	0
sp|Q9CXU9|EIF1B_MOUSE	0	0	1	2	0	0
sp|P36552|HEM6_MOUSE	1	2	0	0	0	0
tr|Q99LF8|Q99LF8_MOUSE	0	0	1	2	0	0
sp|Q9CQF4|CF203_MOUSE	1	1	0	0	1	1
sp|Q9WV98|TIM9_MOUSE	0	0	0	0	1	1
sp|P53994|RAB2A_MOUSE	1	1	0	0	1	1
sp|Q9JJV2|PROF2_MOUSE	1	1	1	1	0	0
sp|Q9R0P9|UCHL1_MOUSE	1	1	0	0	0	0
sp|Q9WUA3|PFKAP_MOUSE	0	0	1	1	0	0
tr|E9PZ43|E9PZ43_MOUSE	1	1	1	1	0	0
sp|Q66JS6|EI3JB_MOUSE	1	1	0	0	1	1
sp|P51885|LUM_MOUSE	0	0	0	0	1	2
sp|Q9CRD2|EMC2_MOUSE	0	0	1	1	1	1
sp|Q9D967|MGDP1_MOUSE	1	1	0	0	1	1
tr|Q9D9G7|Q9D9G7_MOUSE	1	1	0	0	1	1
sp|P31254|UBA1Y_MOUSE	0	0	1	1	0	0
sp|P11798|KCC2A_MOUSE	1	2	0	0	0	0
sp|P04186|CFAB_MOUSE	0	0	0	0	1	1
sp|P97427|DPYL1_MOUSE	1	1	0	0	1	1
sp|P62320|SMD3_MOUSE	0	0	0	0	1	1
sp|Q9Z2W0|DNPEP_MOUSE	0	0	1	2	0	0
sp|Q6ZWN5|RS9_MOUSE	0	0	1	1	1	1
sp|Q9D1R9|RL34_MOUSE	0	0	1	2	0	0
sp|Q9WVA2|TIM8A_MOUSE	0	0	1	1	1	1
sp|Q9JJC6|RIPL1_MOUSE	0	0	0	0	1	1
sp|Q60649|CLPB_MOUSE	1	2	0	0	0	0
sp|Q8C3X2|CC90B_MOUSE	1	1	0	0	1	1
sp|Q8K0Z7|TACO1_MOUSE	0	0	1	1	0	0
tr|A0A0A6YXW6|A0A0A6YXW6_MOUSE	0	0	0	0	1	1
sp|Q8K1B8|URP2_MOUSE	0	0	1	2	0	0
sp|Q05816|FABP5_MOUSE	0	0	0	0	1	1
sp|O35685|NUDC_MOUSE	0	0	1	1	0	0
sp|Q69ZN6|GNPTA_MOUSE	0	0	0	0	1	2
tr|E9PUD2|E9PUD2_MOUSE	1	1	0	0	0	0
sp|Q80W21|GSTM7_MOUSE	0	0	0	0	1	1
sp|Q80XU3|NUCKS_MOUSE	1	1	0	0	0	0
sp|Q9R1P1|PSB3_MOUSE	0	0	0	0	1	1
sp|P12382|PFKAL_MOUSE	0	0	1	1	0	0
sp|P21278|GNA11_MOUSE	0	0	1	1	0	0

sp|Q7TNC4|LC7L2_MOUSE	0	0	1	1	0	0
sp|P40124|CAP1_MOUSE	1	1	0	0	0	0
sp|P26638|SYSC_MOUSE	0	0	0	0	1	1
sp|P56380|AP4A_MOUSE	0	0	1	1	0	0
sp|Q9D1G1|RAB1B_MOUSE	1	1	0	0	0	0
sp|Q6WVG3|KCD12_MOUSE	0	0	1	1	0	0
sp|Q9CXY6|ILF2_MOUSE	0	0	1	1	0	0
sp|Q61292|LAMB2_MOUSE	1	1	0	0	0	0
sp|Q99JB8|PACN3_MOUSE	1	1	0	0	0	0
sp|P35278|RAB5C_MOUSE	0	0	0	0	1	1
sp|O09167|RL21_MOUSE	1	1	0	0	0	0
sp|Q8C129|LCAP_MOUSE	1	1	0	0	0	0
sp|Q9JKB1|UCHL3_MOUSE	1	1	0	0	0	0
tr|Q8C6Y2|Q8C6Y2_MOUSE	0	0	1	1	0	0
sp|Q569Z5|DDX46_MOUSE	1	1	0	0	0	0
sp|P16381|DDX3L_MOUSE	1	1	0	0	0	0
sp|Q9JMA1|UBP14_MOUSE	0	0	0	0	1	1
sp|P01736|TVB3_MOUSE	1	1	0	0	0	0
sp|P56376|ACYP1_MOUSE	0	0	0	0	1	1
sp|P17225|PTBP1_MOUSE	0	0	1	1	0	0
sp|P43275|H11_MOUSE	1	1	0	0	0	0
sp|P31786|ACBP_MOUSE	1	1	0	0	0	0
sp|E9PZQ0|RYR1_MOUSE	0	0	1	1	0	0
sp|Q9D924|ISCA1_MOUSE	1	1	0	0	0	0
sp|O08997|ATOX1_MOUSE	0	0	1	1	0	0
sp|Q9DBG9|TX1B3_MOUSE	1	1	0	0	0	0
sp|P25444|RS2_MOUSE	0	0	1	1	0	0
sp|Q501J6|DDX17_MOUSE	1	1	0	0	0	0
sp|P62073|TIM10_MOUSE	0	0	1	1	0	0
sp|P04919|B3AT_MOUSE	0	0	0	0	1	1
sp|Q8CGY6|UN45B_MOUSE	1	1	0	0	0	0
sp|Q99MR9|PPR3A_MOUSE	0	0	1	1	0	0
sp|Q8C0L0|TMX4_MOUSE	1	1	0	0	0	0
sp|Q9CZD3|GARS_MOUSE	1	1	0	0	0	0
sp|Q62523|ZYX_MOUSE	1	1	0	0	0	0
sp|Q61656|DDX5_MOUSE	0	0	0	0	1	1
sp|Q9CR24|NUDT8_MOUSE	0	0	1	1	0	0
sp|Q9WVL6|EXTL3_MOUSE	0	0	0	0	1	1
sp|Q9CQR2|RS21_MOUSE	0	0	0	0	1	1
sp|Q8K3A0|HSC20_MOUSE	0	0	0	0	1	1
sp|Q91V79|FITM1_MOUSE	0	0	1	1	0	0
sp|Q8JZQ9|EIF3B_MOUSE	0	0	1	1	0	0
sp|P32233|DRG1_MOUSE	1	1	0	0	0	0
sp|Q8VHX6|FLNC_MOUSE	0	0	1	1	0	0
sp|Q3V1D3|AMPD1_MOUSE	1	1	0	0	0	0

sp|Q9DBH5|LMAN2_MOUSE	0	0	1	1	0	0
sp|P48758|CBR1_MOUSE	1	1	0	0	0	0
sp|Q9EQK5|MVP_MOUSE	1	1	0	0	0	0
sp|Q61390|TCPW_MOUSE	0	0	1	1	0	0
sp|Q99N87|RT05_MOUSE	1	1	0	0	0	0
sp|Q9JI75|NQO2_MOUSE	0	0	0	0	1	1
sp|Q9JJZ2|TBA8_MOUSE	1	1	0	0	0	0
sp|Q9Z2Z6|MCAT_MOUSE	0	0	0	0	1	1
sp|P51637|CAV3_MOUSE	1	1	0	0	0	0
sp|Q61704|ITIH3_MOUSE	0	0	1	1	0	0
sp|Q99P72|RTN4_MOUSE	1	1	0	0	0	0
sp|Q8VDM4|PSMD2_MOUSE	1	1	0	0	0	0
sp|A3KMP2|TTC38_MOUSE	1	1	0	0	0	0
sp|P54728|RD23B_MOUSE	0	0	1	1	0	0
sp|Q9EQZ6|RPGF4_MOUSE	0	0	1	1	0	0
sp|Q6PFD6|KI18B_MOUSE	0	0	0	0	1	1
sp|Q9CQW2|ARL8B_MOUSE	0	0	1	1	0	0
sp|P70414|NAC1_MOUSE	0	0	1	1	0	0
sp|Q91VI7|RINI_MOUSE	0	0	1	1	0	0
sp|Q8C0R9|LRRD1_MOUSE	0	0	0	0	1	1
tr|A0A1D5RLZ3|A0A1D5RLZ3_MOUSE	0	0	1	1	0	0
sp|P47962|RL5_MOUSE	0	0	1	1	0	0
sp|Q61702|ITIH1_MOUSE	0	0	1	1	0	0
sp|P36993|PPM1B_MOUSE	0	0	1	1	0	0
sp|Q8R4W6|PCOC2_MOUSE	0	0	1	1	0	0
sp|Q8R1S0|COQ6_MOUSE	0	0	0	0	1	1
sp|B2RVL6|ZCH24_MOUSE	0	0	1	1	0	0
tr|B7ZCI2|B7ZCI2_MOUSE	0	0	0	0	1	1
sp|O88878|ZFAN5_MOUSE	0	0	0	0	1	1
sp|P30115|GSTA3_MOUSE	0	0	1	1	0	0
sp|Q6PCX9|TRI37_MOUSE	0	0	1	1	0	0
sp|P31230|AIMP1_MOUSE	0	0	1	1	0	0
sp|Q9JIP4|PANX1_MOUSE	1	1	0	0	0	0

Supplementary Table 7 Results of mass spectrometry of Set 3 RNA pull-down experiment (>50KDa)

Note 1: Data represented overall Protein scores generated by MASCOT to reflect the confidence of protein identification (https://www.matrixscience.com/help/scoring_help.html)

Note 2: Proteins in the were separated using electrophoresis. Proteins >50 KDa and < 50KDa were sent for mass spectrometry separately. This table presents the results of protein >50KDa.

Accession	CARDINAL pull-down	Control pull-down
O88569	80	0
Q8VEK3	8985	8469
P09405	8819	445
P49312	110	93
Q8BG05	305	236
Q99020	196	159
P61979	8721	2665
Q8R081	7318	352
Q60668	193	142
Q8VEE4	2737	2571
Q05920	2586	1911
Q9Z130	166	130
P62960	1123	204
P63017	2436	2219
P17225	6234	738
Q9JKB3	1069	464
Q9D0E1	4858	5580
P11103	850	1231
Q9CX86	0	0
P60710	136	47
P63260	136	47
P29341	2627	1790
P68033	51	0
P62737	51	0
Q921F4	2801	109
Q9EPU0	1750	996
P42669	0	0
Q6A0A9	1644	325
P04104	622	687
Q8VIJ6	2928	334
P20029	1670	1174
P02535	667	893
Q9DBR1	2125	317
P26369	1769	748
P03975	1123	890
Q9JII5	48	0
P56959	2430	1902
Q61656	2301	1049

Q99K48	2884	1013
P20152	1450	666
O70133	1879	1201
P62806	0	0
Q61879	1688	329
Q8C2Q3	906	1091
Q91VM5	0	0
E9PZJ8	1275	728
Q7TSC1	1622	659
Q6IFZ6	509	0
Q62167	1396	705
Q9WV02	0	0
Q497V5	589	131
Q61584	1559	1081
O35295	0	0
Q3TLH4	784	597
Q00PI9	1222	853
Q7TMK9	2475	237
Q60865	1418	681
Q501J6	1241	674
P38647	893	622
P05213	1175	0
Q61545	1171	765
P68373	0	432
Q569Z6	559	314
Q91VR5	1137	395
P56480	630	421
P99024	1142	704
Q8VH51	1385	427
Q9Z204	0	0
Q922U2	359	254
Q9CZU3	601	269
O35737	904	749
Q03265	926	764
P10126	511	424
P70333	780	743
P97855	2773	1880
Q8VDD5	1008	150
Q5SUQ9	359	217
Q3TTY5	219	229
Q9DB77	0	0
Q99LF4	760	545
Q8VHK9	1426	415
Q64012	0	0
Q8R3C6	912	170

Q02566	892	260
Q9Z2X1	308	205
Q3U9G9	394	233
P47963	0	0
P52479	577	643
Q61990	155	0
P60335	417	44
P97379	877	599
Q921F2	90	30
Q8VED5	254	147
Q8K019	544	147
Q60749	437	182
Q8K310	729	422
Q8BHI4	0	39
Q8BTI8	663	110
Q91W50	1163	61
Q9CXY6	0	0
Q9JIK5	347	40
P17182	58	0
P70372	0	0
P62631	270	265
O35286	479	208
Q5SYD0	468	45
Q5SSW2	150	70
P62862	0	0
P62867	0	0
Q9QWL7	0	163
Q6IFX2	72	157
Q61781	0	0
Q3TEA8	171	555
Q6P4T2	540	190
Q68FD5	435	382
Q3TJZ6	494	289
Q9DCV7	0	0
Q9CQ71	0	0
G5E870	140	153
E9Q7G0	126	0
Q8VC70	0	0
P35922	858	589
Q9Z1X4	780	125
P53564	301	483
O88477	833	78
P10922	0	0
O70157	163	84
Q8BSY0	228	40

Q921M3	523	310
Q7TPV4	237	396
O88532	392	176
A6PWY4	227	144
Q3UKC1	244	0
Q9DB20	0	0
P11499	476	0
Q8R151	398	139
Q8BHD7	1484	156
Q920Q6	0	0
Q5SF07	654	113
P31001	323	182
Q5F2E7	401	367
Q8VDF2	529	136
Q60737	0	0
P54071	0	0
O55143	528	156
Q8BG81	282	155
Q9CYA6	524	47
Q5SFM8	229	147
Q8BI72	306	107
Q7TQH0	527	506
Q05512	560	93
Q91W59	131	186
O88700	114	217
Q8BJL0	178	74
Q91VC3	29	0
Q9D883	0	0
P62242	0	0
Q3TWW8	60	44
Q8C9B9	300	54
Q99NB9	389	225
Q8BMS1	379	114
Q9D8Z1	0	0
P07724	90	119
Q91WR3	307	22
Q8K3G5	163	148
Q9WVR4	598	243
Q8BMK4	413	544
A2AIV2	372	26
Q9WTI7	344	0
Q9JLZ6	103	95
D3YXK2	317	123
Q7TPR4	93	0
P97386	37	103

Q3UYV9	278	43
Q9CR57	0	0
Q8C854	1297	1148
P07901	339	0
P62892	0	0
P61514	0	0
Q8BFR5	0	0
Q8CH25	276	151
Q6PFR5	0	0
O08810	358	65
Q9QXN3	260	138
Q6ZWV3	0	0
P27659	0	0
Q9JIX8	155	79
P62827	0	0
Q61033	454	196
P14869	0	0
Q8K2X3	0	0
Q99PV0	587	256
P63038	364	59
Q8VDN2	216	128
Q9QWT9	170	96
O70338	0	0
P53986	0	34
P32067	152	0
O35326	0	0
P58252	62	33
Q99PU8	175	91
Q02053	49	0
Q60972	118	115
Q60973	118	115
P47911	0	0
Q8VC52	0	0
Q99MN9	81	506
Q8CJG0	116	0
Q03141	274	58
P62983	140	86
P62984	140	86
P0CG49	140	86
P0CG50	140	86
Q80UM7	76	62
Q7TPD0	171	23
P15331	592	108
Q9Z0H1	114	48
Q8K363	110	144

Q3UPF5	204	0
Q8BHN5	112	0
E9Q5K9	206	0
Q8CGU1	0	0
Q6ZWY9	0	0
Q8CGP0	0	0
Q8CGP1	0	0
Q8CGP2	0	0
Q9D2U9	0	0
P10853	0	0
P10854	0	0
Q64475	0	0
Q64478	0	0
Q64524	0	0
Q64525	0	0
E9Q557	154	0
P25444	66	0
P16858	27	0
P10404	153	0
Q922R8	55	0
Q9D4G9	47	26
P05064	0	0
Q8K0L9	182	56
Q61029	231	106
Q9CQ69	0	0
Q99KI0	83	32
P25976	62	25
Q99JX7	154	56
Q99LH1	165	0
Q91ZA3	69	817
B1AZI6	89	41
P18572	0	50
Q9R1C6	60	0
Q8QZY9	0	0
P14824	47	70
O88291	216	91
O88735	41	0
Q8K1N4	45	40
P49718	157	0
P43274	0	49
P13864	249	52
Q922H2	0	0
P51881	0	30
O08917	0	0
P08752	0	0

Q8BKF1	131	40
P70318	0	0
P23249	257	0
Q9QZM2	0	0
P63325	0	0
Q91YN9	0	0
P70168	104	0
Q9CQE8	0	0
Q6ZQJ5	89	0
P14602	0	0
Q8BL97	0	0
Q9D8E6	256	78
Q8VDM6	120	57
Q99JR5	58	25
Q02257	100	33
Q99LC3	0	0
P27641	30	137
P05132	0	0
P14094	46	0
P09055	42	46
Q99MR8	74	317
P47753	0	0
Q925I1	253	53
Q08288	0	0
P97863	83	69
Q8K3J1	0	0
Q91W39	182	105
Q9CZS1	131	0
P55096	61	30
Q6PB66	165	31
Q9EPL8	0	0
Q6A068	143	18
Q62351	23	0
Q8K4Q6	0	0
Q9JIF0	0	0
P16546	151	39
Q91Z49	0	0
Q61753	22	49
P00405	0	0
Q6NS46	55	28
Q8K2I9	0	0
P35601	0	40
P14115	0	0
Q9WUA3	177	43
P11276	87	47

Q9CY58	108	0
Q7TNC4	0	0
Q9D6R2	0	0
Q8BVY0	187	162
Q9QXK3	51	0
Q9QZE5	51	0
Q61329	35	37
Q91YQ5	86	78
P62245	0	0
O35643	0	0
P62996	0	0
P39447	72	40
P42582	0	0
Q99JB2	0	0
Q7TPW1	84	0
Q6PHZ5	83	0
E9PVA8	49	58
Q62261	196	46
P13705	68	0
Q99PL5	128	29
P17809	0	0
Q8BWW4	217	0
Q8BYH7	0	0
P26040	35	0
P26041	35	0
P26043	35	0
Q9CZD3	35	0
Q8CAQ8	175	53
Q9DBR0	66	0
P11152	103	18
Q8BX17	145	33
Q3US41	0	0
Q8K0G8	0	0
Q8VDJ3	1434	0
P02088	0	49
P02089	0	49
P02104	0	0
Q9D0F6	0	0
P46467	0	0
Q9ESZ8	237	0
P21981	85	49
Q3UKJ7	128	22
P14873	47	0
P62702	0	0
Q80TV8	148	0

P35550	0	0
O35682	0	0
O54724	0	29
Q99J62	0	0
P07356	0	0
Q9Z1G4	75	0
Q9CR60	0	0
Q9D824	316	79
P35700	0	0
P06745	129	25
Q60597	0	0
Q9D0B0	0	0
P14211	0	0
Q9CQF3	0	0
Q99KP6	147	53
P26039	42	0
Q9EQP2	124	48
Q6A065	85	104
P35564	126	0
P50516	0	0
O35691	56	0
Q8C3F2	169	18
B2RY56	74	39
Q9ER69	170	0
E9Q5G3	101	0
Q8VDW0	0	0
Q9Z1N5	34	0
Q9CQD1	0	0
P35278	0	0
P61021	0	0
Q8K409	0	0
P09103	47	0
Q3TDQ1	102	66
Q91YE6	36	0
Q8K4Z5	148	25
Q76MZ3	55	0
P09411	0	0
Q52KI8	40	0
P19324	0	0
Q9JJI8	0	0
Q80VP2	0	0
P14206	0	0
P43488	27	23
Q7SIG6	26	26
P14131	0	0

Q6PCM2	134	0
Q922Q8	0	0
Q9DC69	0	0
Q9JIF7	44	0
B2RRE7	54	39
Q7TT45	0	0
Q99K70	0	0
P46737	26	25
Q8JZX4	0	0
O70373	0	0
Q8CGC6	29	0
P57776	0	0
Q8C5N3	68	17
P27773	94	27
P68181	0	0
Q9JJ28	68	25
Q6LBE8	0	0
Q6LBF0	0	0
P02301	0	0
P68433	0	0
P84228	0	0
P84244	0	0
Q9Z110	72	25
Q6PDQ2	141	78
Q8R1M0	0	0
Q9JJD0	0	0
O35083	0	0
Q9CXF4	36	0
Q9D023	0	0
Q8CIE6	54	26
Q0P678	39	26
P84089	0	0
P62962	0	0
P54823	108	86
Q62448	21	0
Q61468	21	23
Q9Z1W9	51	0
P80315	0	0
Q66JY6	0	0
Q91WJ7	32	0
Q9EPU4	285	77
Q9Z1Z2	0	0
Q9Z0R9	0	0
Q8C779	0	0
Q91Z31	3342	0

Q3U0V1	694	36
Q6NXH9	534	0
Q9Z0H4	340	0
Q8BGZ7	315	249
Q8BH59	254	86
Q8VDP4	248	0
P70182	237	96
Q9Z321	218	0
P08113	217	0
Q6ZQ58	213	45
P28659	191	0
Q91V81	190	33
Q8R326	185	0
Q80YR5	177	0
Q9CW46	173	0
Q9R226	165	73
Q9Z0X1	164	61
Q6NVF9	162	0
Q62318	162	0
Q9D554	160	52
Q8BTV2	153	18
Q61595	150	48
Q9JKF1	148	0
Q99LC2	139	0
Q8BK35	135	30
Q5SWU9	131	241
P70403	126	192
Q64337	122	24
P43247	121	29
Q91WJ8	119	0
Q62376	119	54
Q9DBG3	119	0
Q8BP71	116	29
Q9R190	110	120
Q61414	108	0
Q5SRY7	107	35
Q8CH18	98	0
Q3UEB3	98	0
Q922P9	96	59
Q9Z108	95	28
Q9DBG6	95	0
P57780	93	0
Q9Z1M8	93	32
Q8BKT7	89	0
Q9QXK7	89	0

Q8CFQ3	86	382
Q80WJ7	86	0
P70288	84	25
Q6P5D8	82	0
Q6PCN7	79	0
P54728	78	0
Q7TPM1	77	0
Q6NZN0	76	52
Q9D6Z1	75	0
P47738	74	26
O70305	73	32
Q923D5	72	0
Q61464	72	0
Q99MR6	70	0
Q8K2B3	70	0
P17426	70	0
Q8K2F8	67	0
Q9Z0U1	67	0
Q99LI7	67	0
Q08369	66	64
Q64213	66	26
Q9JKR6	65	38
Q6URW6	64	0
Q924X2	64	0
Q9D0G0	64	0
Q3URQ0	63	0
Q9R210	62	30
O08811	62	0
P48962	60	0
Q8CGC4	60	0
Q07832	59	0
Q6NZJ6	59	0
O35218	58	0
Q8CFG0	58	25
Q9JHI7	57	0
Q8BMF4	57	0
P02340	57	0
Q91W18	56	0
Q8BTM8	56	0
E9PZM4	56	0
O08582	55	0
Q8R3N6	54	0
Q9DCD2	53	0
P28656	53	0
Q9D8N0	53	57

Q9QXS1	53	0
Q5SUF2	52	0
Q01853	52	0
Q80U58	51	49
Q9ERH4	51	64
Q91YR7	51	0
Q920A7	51	0
Q99JY9	51	0
P68134	51	0
Q61510	50	0
Q8BK67	50	33
Q8VEG4	50	0
Q9QY40	49	26
Q8BZR9	49	0
Q6KCD5	49	0
P47857	48	0
O35114	48	0
Q8VEJ9	48	0
Q9CSH3	48	0
P97742	48	0
Q91YT7	47	0
P59326	47	0
P23116	47	0
Q99ME2	46	23
Q9JLV1	46	35
P54099	45	111
Q60598	45	34
Q99L45	44	0
Q8C8U0	44	0
P40694	44	0
O35492	43	0
Q91VX2	43	0
Q9CSN1	43	24
Q9CS00	43	0
Q8BGD9	42	0
Q9JJY4	42	0
Q07139	42	0
P97868	41	0
P43029	41	0
Q3V1H1	39	0
Q99MU3	39	0
P60122	39	0
P08775	38	0
Q9ESX5	38	0
Q9ESL4	37	0

Q63918	37	0
P30999	37	0
Q8BH04	37	0
Q8K4P0	36	0
P48678	35	0
Q8BLF1	35	0
Q6NZF1	35	0
Q9D952	35	0
P25206	35	0
P97479	35	0
Q68FH0	35	0
Q922B2	35	0
Q9WTY1	35	0
P58501	34	48
Q9EQM6	33	0
Q9D0I9	33	0
Q9JKN6	33	0
Q8R0T2	33	0
Q3UTJ2	32	0
Q6IQX8	32	0
Q99JF8	32	0
Q8C3P7	32	0
Q8BP47	32	0
Q6NV83	32	0
Q8CGZ0	32	0
Q14C51	32	0
Q99104	32	0
Q61301	32	0
Q9DC23	31	0
Q8BMD6	31	0
P56960	31	0
Q62203	31	37
P62814	31	22
Q05793	31	0
Q99K43	30	27
Q8K203	30	85
Q80X50	30	36
O70551	30	0
Q8BJA3	30	79
Q8BYK6	29	0
Q8BKC5	28	0
Q9D0K2	28	0
Q99M28	28	0
P80318	28	24
Q62181	28	0

Q9QYH6	28	0
Q5HZJ0	28	0
Q3ULD5	27	165
Q9R0L7	27	0
Q9Z0N1	27	0
Q9Z0N2	27	0
Q8BGS1	27	0
C0HKD8	26	0
C0HKD9	26	0
Q9JLI8	26	0
P11983	26	0
Q9DBA9	26	0
Q07133	26	49
P58242	26	0
Q8K4G5	26	0
Q61398	26	0
Q03963	26	0
O08749	25	0
Q8JZN5	25	27
Q91YK8	25	0
P97789	24	0
P24547	23	24
Q7TMY4	23	0
Q91VN6	23	0
O35343	23	0
Q569Z5	22	0
Q8CGY8	22	0
Q8CI11	22	0
Q6ZPZ3	21	0
P11438	21	0
Q8BT07	21	0
Q9D0L8	21	0
Q3UFY0	20	0
P52431	20	0
P19096	18	0
Q3TIX9	17	0
Q8R361	16	0
Q6RHW0	14	0
P54276	0	318
P52480	0	143
P23475	0	86
Q9D2G2	0	60
O54734	0	60
Q9CQN1	0	59
P26443	0	59

Q812E0	0	56
Q75NR7	0	53
P15864	0	49
P43277	0	49
Q8VE97	0	49
Q8BZ20	0	47
O88874	0	39
P02468	0	39
Q8K3Z9	0	38
Q9Z1J1	0	37
Q00417	0	37
Q924A0	0	37
P27782	0	37
Q64516	0	36
Q91VD9	0	34
Q9Z2D6	0	34
Q6NVF4	0	30
Q9JJ43	0	29
Q8BGH2	0	28
Q8JZR0	0	28
P41216	0	28
Q5SXY1	0	27
Q6PAC3	0	24
Q9EP53	0	24
Q9WVM1	0	22
P31266	0	20
Q9JIY2	0	20
Q6P5B0	0	19
Q3U1J4	0	18
P53657	0	17
Q9Z1Q9	0	17
Q9CWL2	0	13
Q8VCW8	0	13

Supplementary Table 8 Results of mass spectrometry of Set 3 RNA pull-down experiment (<50KDa)

Note 1: Data represented overall Protein scores generated by MASCOT to reflect the confidence of protein identification (https://www.matrixscience.com/help/scoring_help.html)

Note 2: Proteins in the were separated using electrophoresis. Proteins >50 KDa and < 50KDa were sent for mass spectrometry separately. This table presents the results of protein <50KDa.

Accession	CARDINAL pull-down	Control pull-down
O88569	10505	10924
P49312	7149	6400
Q8BG05	6954	6453
Q99020	4141	5088
P62960	4001	2173
Q60668	3204	2914
Q9JKB3	2758	1747
Q9CX86	2252	2467
P60710	2635	1195
P63260	2635	1195
P62737	2542	1238
Q9Z130	1892	2014
Q61990	3521	416
P60335	5272	303
P57722	2677	0
Q91VM5	1347	680
Q9WV02	1085	644
P25444	972	742
P62702	1217	914
O89086	1371	1149
P11031	493	535
P42669	851	1241
P62908	1322	759
P62806	598	289
Q9WUB3	0	0
P04104	1263	547
P61979	1496	171
P63325	362	602
Q9JII5	822	862
P62270	520	686
O35295	360	723
Q8VEK3	830	489
P70372	1735	886
Q6PDM2	743	387
P62754	505	665
P03975	455	399
P51881	751	572
P02535	811	499

Q62193	493	569
O08583	886	583
P09405	762	238
Q6ZWY9	635	624
Q8CGP1	635	0
Q8CGP2	635	624
P10853	635	624
P10854	635	624
Q64475	635	624
Q64478	635	624
Q64525	635	624
P62281	372	342
Q8BP67	172	474
P48962	667	626
P62751	259	872
Q3TWW8	534	190
Q8BHI4	390	463
Q6ZWN5	497	538
Q64012	662	480
Q9CXY6	931	338
Q9CQE8	606	455
Q8BL97	406	0
P47963	215	118
Q9Z204	873	578
P16858	450	293
P15864	440	770
P43277	0	0
P43274	467	698
P40630	242	377
P84104	351	173
Q6PFR5	342	176
Q3TTY5	567	146
O35326	606	211
Q922U2	676	183
Q9CQ71	165	176
Q9CYR0	194	392
Q9CXW4	234	236
P62301	357	218
P14115	229	246
P70318	1324	71
P60824	281	292
P62849	310	433
Q8VC70	183	269
P62862	172	178
P62867	172	178

Q6ZWV3	378	157
Q60930	309	283
P62242	285	165
Q9DB77	307	213
P62852	128	265
P17182	469	272
Q9D883	321	164
P54071	271	130
P14131	217	145
Q8K2X3	226	91
Q9CY57	166	153
Q8VED5	447	98
Q9WVB0	476	0
Q9DB20	152	249
Q9Z1Z2	824	96
Q6ZWV7	131	234
Q8R081	267	0
Q91VR2	376	175
P12970	201	176
Q60737	301	244
P60867	137	115
Q921F2	928	419
Q6IFX2	180	116
Q8BJ90	152	53
P56959	210	245
P14869	395	213
P10922	69	153
Q9CPR4	208	294
Q8BG81	112	65
P10126	171	154
Q91VC3	312	168
Q8VC52	303	0
Q9R0U0	108	72
P35980	191	177
P62264	205	177
P29391	89	134
P49945	89	0
P47754	197	46
O35737	194	190
Q9QWL7	0	109
P61255	71	265
Q9Z2X1	325	197
P27048	188	84
P63163	188	84
Q3THE2	84	108

P47753	135	56
Q9CWX4	127	0
P32067	427	77
P97461	47	50
Q8BWL5	0	120
P62900	163	195
P62996	246	80
P47911	332	163
P42582	198	82
Q9D8Z1	124	118
P62892	68	118
P20152	139	96
P61358	148	118
Q60931	181	120
P14148	193	80
P35550	84	55
P62830	78	95
P97351	261	212
Q9D0B0	101	0
Q91Z49	149	62
P51174	118	38
Q9CZU6	108	0
P19324	95	0
Q9CQT2	64	72
P35979	105	102
P0C0S6	0	65
Q3THW5	0	65
P02088	31	0
P60843	110	85
Q9D8M4	130	256
P62911	55	56
P27659	208	103
P51410	64	107
Q99LC5	136	48
Q9CXC3	32	78
P61027	87	99
Q99M28	105	78
Q8QZY9	131	38
Q5U4D9	55	67
O70325	54	41
P53026	182	101
P09541	56	66
Q920Q6	213	360
Q6LBE8	49	32
Q6LBF0	49	32

P02301	49	32
P68433	49	32
P84228	49	32
P84244	49	32
P07724	79	98
Q9CQ19	82	0
O09167	168	81
Q9CQ69	90	71
P62918	125	103
P05064	50	0
P35486	76	40
P47757	59	57
P42125	66	40
Q60605	58	46
Q91YN9	76	71
Q9CZX8	48	168
Q9QVP4	0	18
O08917	107	50
P29387	38	0
Q9D8E6	183	85
Q8BFR5	126	71
Q9D8B3	86	66
P09528	43	69
O70338	26	75
P45376	0	31
O35682	30	41
P62245	88	65
Q9CR57	79	0
P68254	94	0
Q922H2	81	66
Q9D6R2	28	52
P57784	176	74
P62137	58	53
P97447	62	0
P14602	60	49
P62821	0	135
P63101	131	62
Q9JIF0	148	41
Q9D051	122	140
P61514	92	45
P51150	30	139
P26369	94	57
P53986	92	67
P08752	177	53
P62717	40	57

P09411	86	52
Q9D0M3	98	74
P35700	82	0
P62827	68	0
Q61187	0	0
Q91XB0	39	126
P61205	66	83
P84078	66	83
P84084	66	83
Q8BVW3	0	0
P57776	58	47
P62962	36	0
Q62093	982	304
Q9JLZ6	112	54
O08573	65	0
Q99LB0	57	0
Q5SSK3	101	60
Q9CPR5	42	32
Q61425	42	0
Q9DCU6	42	36
P17742	54	51
Q99KV1	0	57
P62843	142	52
P17751	61	47
P08249	178	61
Q99LC3	59	50
P05132	52	62
P00405	61	50
Q922Q8	67	46
Q6PE01	117	48
Q9CR62	127	85
O70503	83	43
P05213	60	33
P68369	60	33
P68373	60	33
Q9DCW4	76	102
Q9JM76	46	32
Q8K4Q6	29	51
Q9CQF3	191	45
P61750	0	0
P29758	28	0
Q9QZM2	0	26
D3Z4I3	113	102
Q62176	113	102
P68181	33	33

P27671	0	0
P29341	91	0
Q9WTX2	118	28
Q9DC70	48	64
Q8VEE4	29	31
Q9DC28	158	0
A2ADA5	60	71
Q99J47	33	0
Q9D7S7	0	0
Q7TNC4	58	46
Q9CQ22	0	0
P99024	0	0
P17225	627	0
Q99J62	0	43
P14873	0	52
Q60634	88	0
Q8R2G4	0	31
Q9CZ13	0	0
P17809	57	0
Q08288	47	63
Q62186	22	70
P18572	0	27
P14152	0	0
Q99JY0	25	36
Q9CR68	47	72
P63037	74	52
Q9CZU3	0	0
Q9CQI7	58	105
Q62189	0	0
P62317	43	80
P06151	52	48
P63017	72	37
P45952	46	0
Q91WN1	0	179
Q8QZT1	35	0
E9PVA8	44	32
P21107	45	48
P35293	0	0
Q9CQD1	41	24
P35278	41	24
P61021	41	24
Q9WV55	0	60
Q8VEM8	127	48
P46638	44	44
O08807	42	0

P62315	0	23
Q9D0R8	0	103
Q99JP7	0	0
O70133	57	44
Q3TUH1	0	39
P57724	203	0
P62855	31	38
Q9CWZ3	30	34
Q9WUK4	65	27
Q8BX10	74	57
Q80VD1	55	0
Q9JJD0	44	0
Q99N93	100	44
P16045	0	0
Q9JIK9	27	0
P68040	29	0
P41105	0	0
Q9D0G0	0	0
Q01853	0	0
Q9JJI8	46	76
P97863	52	26
P62983	32	38
P62984	32	38
P0CG49	32	0
P0CG50	32	0
P63276	84	91
Q9DB34	0	47
Q9D0D4	0	28
Q8VBV3	41	0
Q9ESW4	25	0
Q9WTX5	0	0
P62320	27	0
Q9D7K2	0	31
Q5SUQ9	33	28
P83882	0	0
Q9CQ10	0	0
P56480	0	0
Q7SIG6	29	0
Q3TSG4	23	0
O08756	48	36
Q8JZU2	0	0
Q9R0P6	0	0
Q9DBJ1	45	63
Q9CY27	74	76
Q9D384	56	0

Q8VDP6	33	0
P01901	32	44
P03991	32	44
P04223	32	44
P14426	32	44
P14428	32	44
Q8K072	54	0
Q91V12	0	0
P47955	40	33
P49813	74	43
P10833	0	0
P14206	74	46
P70404	26	0
Q8BYI8	24	21
Q02257	68	0
P84089	0	34
Q921F4	64	0
Q3UIU2	0	0
Q6IFZ6	1017	0
P52912	655	0
P50446	617	0
Q9DCV7	346	0
Q61414	299	0
P28659	271	0
P19001	231	0
Q61781	202	0
Q9D0F9	162	0
Q8BHD7	146	0
Q9Z2K1	132	0
Q91W59	111	0
Q91Z31	111	0
Q9D0E1	101	137
Q91Z92	98	0
Q7TMK9	96	0
Q80VL1	94	0
C0HKE1	94	99
C0HKE2	94	99
C0HKE3	94	99
C0HKE4	94	99
C0HKE5	94	99
C0HKE6	94	99
C0HKE7	94	99
C0HKE8	94	99
C0HKE9	94	99
Q6GSS7	94	99

Q8BFU2	94	99
Q8CGP5	94	99
Q8CGP6	94	99
Q8CGP7	94	99
Q8R1M2	94	99
Q64523	94	99
Q9CZM2	90	0
Q8VH51	80	0
Q8C7Q4	78	0
Q9DCT2	78	81
Q91W39	74	0
E9Q557	70	0
P99027	70	0
Q9DBC7	68	0
Q8C2Q3	68	0
P97376	68	0
Q9CXW2	66	0
P61620	66	31
Q99JI4	64	25
P11499	60	0
Q9WV32	59	0
Q60759	59	0
Q9QYR6	56	0
Q9CYH6	56	27
P62267	56	74
P67984	56	49
Q9D1G1	56	86
Q66JY6	55	48
O08579	54	35
Q9DCT8	54	25
Q9QZH3	53	76
Q8CD10	51	0
Q60865	51	38
O35129	50	83
Q9DC69	49	96
Q8BHE0	48	0
P97315	47	45
Q9CRB2	47	0
Q91V04	47	0
Q9CS42	46	0
Q9D7G0	46	0
P58771	45	0
P58774	45	0
Q791T5	44	27
Q61743	43	0

Q99K48	43	0
Q9Z127	43	0
Q9QYJ0	42	0
Q9DBR1	42	0
Q91VR5	41	0
P19253	41	0
Q99JB2	40	52
Q6T707	40	0
Q99PL7	40	0
P13011	40	0
P13516	40	0
Q8BHS6	40	0
Q99M87	40	0
Q61656	39	0
O55143	39	0
Q6GYP7	39	0
Q9DC71	39	0
P62874	38	0
P62880	38	0
Q8BH95	37	28
Q8JZM0	37	48
Q9CRA8	36	0
Q9CWT2	36	24
Q8BI72	35	0
A2A8U2	34	0
Q61545	33	72
P21278	33	0
P21279	33	0
P30677	33	0
Q921I9	32	0
Q03265	32	30
P32233	32	0
O70152	32	31
O35730	32	0
Q9ESX5	31	0
P51863	31	0
Q9QZ06	31	0
P62889	31	50
P02089	31	0
P02104	31	0
Q569Z6	31	0
Q8BK72	31	0
Q80ZS3	30	32
Q9D1R9	30	30
P47962	30	0

Q7TSF1	30	0
Q61495	30	0
Q80UW8	29	0
Q8JZX4	29	0
Q8C1B7	29	0
Q8CHH9	29	0
Q791V5	29	43
O08992	29	26
Q99J72	29	28
Q9Z1D1	29	0
O55142	29	0
Q9D0I8	28	0
P62482	28	0
Q9JL15	27	0
P62305	27	0
Q9ER88	26	0
Q8VIJ6	26	0
Q69ZQ2	26	94
Q9D6X6	26	0
P62082	26	39
Q8BGV7	26	0
Q922M3	26	0
O70479	26	0
P46737	26	0
Q8VE80	26	0
P62311	25	0
Q91WD5	25	0
O08547	25	0
Q9JKY0	25	0
Q9D023	25	37
Q8R1M0	25	0
Q9D287	24	72
Q8BP71	24	0
Q9JJ43	24	0
P59999	24	27
Q8C570	24	42
P62334	24	0
Q9D7N3	24	0
P01942	23	0
O88667	23	25
Q920S3	23	0
Q9D0M0	23	0
Q6ZWX6	23	0
Q9CQM9	23	0
B9EJA2	23	0

Q9D0F6	23	30
Q2VIS4	22	0
O88477	22	0
Q920L1	22	0
Q8BFV2	20	0
Q9CR60	20	19
Q06185	19	0
Q8BTV2	18	0
P10404	17	0
P97855	17	26
P52432	17	0
P28658	17	0
Q8K310	16	0
P68033	0	1264
P10630	0	85
P43275	0	80
P06745	0	69
P62071	0	68
P53994	0	66
Q9D880	0	60
Q9QY76	0	60
Q9DCF9	0	60
Q9DCS9	0	50
P11103	0	45
P01895	0	44
P01896	0	44
P01897	0	44
P01898	0	44
P01899	0	44
P01900	0	44
P14429	0	44
P14430	0	44
Q8C854	0	43
P05977	0	43
Q921L3	0	40
Q3US41	0	40
Q8K0G8	0	40
Q9D937	0	40
P50518	0	36
Q9CQL1	0	33
P61327	0	33
P61226	0	32
Q9JLR1	0	31
Q04841	0	30
Q921W0	0	28

O35286	0	28
Q8VEH3	0	28
Q9CQW2	0	28
Q8K409	0	25
P61161	0	25
P00397	0	23
Q7M732	0	22
P51655	0	21
P48545	0	21

Supplementary Table 9 List of DRG1 interacting proteins

Note: Data in each column represented numbers of total peptides that matched to each protein

Description	Peptides matched in Ad-HA-Drg1 group	Peptide matched in Ad-GFP group	Subgroup
60S ribosomal protein L35 OS=Rattus norvegicus OX=10116 GN=Rpl35 PE=1 SV=3	19	5	Ribosome component
60S ribosomal protein L3 OS=Rattus norvegicus OX=10116 GN=Rpl3 PE=1 SV=3	2	0	Ribosome component
60S ribosomal protein L13 OS=Rattus norvegicus OX=10116 GN=Rpl13 PE=1 SV=2	53	0	Ribosome component
60S ribosomal protein L27a OS=Rattus norvegicus OX=10116 GN=RGD1562402 PE=3 SV=1	22	5	Ribosome component
60S ribosomal protein L29 OS=Rattus norvegicus OX=10116 PE=3 SV=1	3	0	Ribosome component
60S ribosomal protein L18 OS=Rattus norvegicus OX=10116 GN=Rpl18 PE=1 SV=1	8	0	Ribosome component
60S ribosomal protein L39 OS=Rattus norvegicus OX=10116 GN=Rpl39 PE=1 SV=1	2	0	Ribosome component
60S ribosomal protein L36 OS=Rattus norvegicus OX=10116 GN=Rpl36 PE=3 SV=1	3	0	Ribosome component
60S ribosomal protein L13a OS=Rattus norvegicus OX=10116 PE=3 SV=1	9	0	Ribosome component
Rpl7 protein (Fragment) OS=Rattus norvegicus OX=10116 GN=Rpl7 PE=2 SV=1	4	0	Ribosome component
Large subunit ribosomal protein L36a, isoform CRA_a OS=Rattus norvegicus OX=10116 GN=Rpl36a PE=2 SV=1	5	0	Ribosome component
40S ribosomal protein S8 OS=Rattus norvegicus OX=10116 GN=Rps8 PE=2 SV=1	6	0	Ribosome component
60S ribosomal protein L21 OS=Rattus norvegicus OX=10116 PE=3 SV=1	14	0	Ribosome component
40S ribosomal protein S15a OS=Rattus norvegicus OX=10116 GN=LOC100909878 PE=3 SV=1	6	0	Ribosome component
60S ribosomal protein L38 OS=Rattus norvegicus OX=10116 PE=3 SV=2	2	0	Ribosome component
40S ribosomal protein S4 OS=Rattus norvegicus OX=10116 GN=Rps4y2 PE=1 SV=1	32	0	Ribosome component
60S ribosomal protein L8 OS=Rattus norvegicus OX=10116 PE=3 SV=1	1	0	Ribosome component
60S ribosomal protein L34 OS=Rattus norvegicus OX=10116 GN=Rpl34 PE=3 SV=1	2	0	Ribosome component
60S ribosomal protein L19 OS=Rattus norvegicus OX=10116 PE=3 SV=2	1	0	Ribosome component

40S ribosomal protein S6 OS=Rattus norvegicus OX=10116 GN=Rps6 PE=1 SV=1	12	5	Ribosome component
40S ribosomal protein S30 OS=Rattus norvegicus OX=10116 GN=LOC100360647 PE=1 SV=1	8	1	Ribosome component
60S ribosomal protein L28 OS=Rattus norvegicus OX=10116 GN=Rpl28 PE=2 SV=1	10	0	Ribosome component
60S ribosomal protein L4 OS=Rattus norvegicus OX=10116 GN=Rpl4 PE=1 SV=1	5	0	Ribosome component
40S ribosomal protein S27a OS=Rattus norvegicus OX=10116 GN=Rps27a PE=2 SV=1	14	0	Ribosome component
Heat shock cognate 71 kDa protein OS=Rattus norvegicus OX=10116 GN=Hspa8 PE=1 SV=1	44	0	Ribosome interacting protein
ADP/ATP translocase 1 OS=Rattus norvegicus OX=10116 GN=Slc25a4 PE=1 SV=3	15	0	Ribosome interacting protein
RNA cytidine acetyltransferase OS=Rattus norvegicus OX=10116 GN=Nat10 PE=1 SV=1	5	0	Ribosome interacting protein
Spectrin alpha chain, non-erythrocytic 1 OS=Rattus norvegicus OX=10116 GN=Sptan1 PE=1 SV=1	4	0	Ribosome interacting protein
NOP14 nucleolar protein OS=Rattus norvegicus OX=10116 GN=Nop14 PE=1 SV=1	2	0	Ribosome interacting protein
PRP4 pre-mRNA-processing factor 4 homolog OS=Rattus norvegicus OX=10116 GN=Prpf4b PE=1 SV=1	2	0	Ribosome interacting protein
Fructose-bisphosphate aldolase OS=Rattus norvegicus OX=10116 PE=3 SV=1	3	0	Ribosome interacting protein
H1.2 linker histone, cluster member OS=Rattus norvegicus OX=10116 GN=Hist1h1c PE=1 SV=1	8	3	Ribosome interacting protein
LUC7-like (S. cerevisiae) OS=Rattus norvegicus OX=10116 GN=Luc7l PE=2 SV=1	3	0	Ribosome interacting protein
RCG34104, isoform CRA_a OS=Rattus norvegicus OX=10116 GN=Tsr1 PE=1 SV=1	4	1	Ribosome interacting protein
Ribosomal RNA-processing 12 homolog OS=Rattus norvegicus OX=10116 GN=Rrp12 PE=1 SV=1	17	4	Ribosome interacting protein

tRNA methyltransferase 10 homolog C OS=Rattus norvegicus OX=10116 GN=Trmt10c PE=2 SV=1	3	1	tRNA/rRNA modification enzyme
rRNA adenine N(6)-methyltransferase OS=Rattus norvegicus OX=10116 GN=Dimt1 PE=2 SV=1	1	0	tRNA/rRNA modification enzyme
BUD23, rRNA methyltransferase and ribosome maturation factor OS=Rattus norvegicus OX=10116 GN=Bud23 PE=1 SV=1	2	0	tRNA/rRNA modification enzyme
Histone H1.5 OS=Rattus norvegicus OX=10116 GN=H1-5 PE=1 SV=1	2	0	Other
Histone H2A type 1-C OS=Rattus norvegicus OX=10116 PE=1 SV=2	11	0	Other
Calmodulin-1 OS=Rattus norvegicus OX=10116 GN=Calm1 PE=1 SV=1	2	0	Other
Deoxyribonuclease-1-like 1 OS=Rattus norvegicus OX=10116 GN=Dnase1l1 PE=2 SV=1	1	0	Other
39S ribosomal protein L38, mitochondrial OS=Rattus norvegicus OX=10116 GN=Mrpl38 PE=2 SV=2	2	0	Other
Ankycorbin OS=Rattus norvegicus OX=10116 GN=Rai14 PE=1 SV=2	1	0	Other
Junction plakoglobin OS=Rattus norvegicus OX=10116 GN=Jup PE=1 SV=1	7	3	Other
Zinc finger CCCH domain-containing protein 15 OS=Rattus norvegicus OX=10116 GN=Zc3h15 PE=1 SV=1	23	0	Other
Phosphorylase b kinase regulatory subunit OS=Rattus norvegicus OX=10116 GN=Phka1 PE=1 SV=1	2	0	Other
Prelamin-A/C OS=Rattus norvegicus OX=10116 GN=Lmna PE=1 SV=1	5	0	Other
Myomegalin OS=Rattus norvegicus OX=10116 GN=Pde4dip PE=1 SV=1	4	0	Other
Nebulette OS=Rattus norvegicus OX=10116 GN=Nebl PE=1 SV=1	3	0	Other
GMP reductase OS=Rattus norvegicus OX=10116 GN=Gmpr2 PE=1 SV=1	1	0	Other
Tropomyosin 1, alpha, isoform CRA_i OS=Rattus norvegicus OX=10116 GN=Tpm1 PE=1 SV=1	7	0	Other
Prolyl endopeptidase-like OS=Rattus norvegicus OX=10116 GN=Prepl PE=1 SV=1	6	0	Other
HECT-type E3 ubiquitin transferase OS=Rattus norvegicus OX=10116 GN=Nedd4l PE=1 SV=1	7	0	Other

F-actin-capping protein subunit beta OS=Rattus norvegicus OX=10116 GN=Capzb PE=1 SV=1	1	0	Other
Serine and arginine repetitive matrix 1 OS=Rattus norvegicus OX=10116 GN=Srrm1 PE=1 SV=1	5	2	Other
Cytochrome c oxidase subunit 2 (Fragment) OS=Rattus exulans OX=34854 GN=COX2 PE=3 SV=1	3	0	Other
Bag2 protein OS=Rattus norvegicus OX=10116 GN=Bag2 PE=2 SV=1	3	0	Other
NTF2-related export protein 1 (Predicted), isoform CRA_a OS=Rattus norvegicus OX=10116 GN=Nxt1 PE=2 SV=1	2	0	Other
Nexilin (Fragment) OS=Rattus norvegicus OX=10116 PE=2 SV=1	13	0	Other
DLST protein OS=Rattus norvegicus OX=10116 GN=DLST PE=2 SV=1	2	0	Other
MSL complex subunit 1 OS=Rattus norvegicus OX=10116 GN=Msl1 PE=1 SV=1	2	0	Other
DIRAS family GTPase 2 OS=Rattus norvegicus OX=10116 GN=Diras2 PE=4 SV=1	2	0	Other
Uncharacterized protein OS=Rattus norvegicus OX=10116 PE=3 SV=1	3	0	Other
Treacle ribosome biogenesis factor 1 OS=Rattus norvegicus OX=10116 GN=Tcof1 PE=1 SV=2	6	0	Other
Uncharacterized protein OS=Rattus norvegicus OX=10116 PE=3 SV=1	6	0	Other
Heterogeneous nuclear ribonucleoprotein A3-like OS=Rattus norvegicus OX=10116 GN=LOC100911361 PE=1 SV=3	2	0	Other
Fibroblast activation protein, alpha OS=Rattus norvegicus OX=10116 GN=Fap PE=4 SV=3	1	0	Other
Desmoplakin OS=Rattus norvegicus OX=10116 GN=Dsp PE=1 SV=1	31	11	Other
Ribosome-releasing factor 2, mitochondrial OS=Rattus norvegicus OX=10116 GN=Gfm2 PE=1 SV=2	3	0	Other
Myosin heavy chain 14 OS=Rattus norvegicus OX=10116 GN=Myh14 PE=1 SV=1	21	0	Other
Dmx-like 2 OS=Rattus norvegicus OX=10116 GN=Dmxl2 PE=1 SV=3	5	0	Other
Ecm29 proteasome adaptor and scaffold OS=Rattus norvegicus OX=10116 GN=Ecpas PE=1 SV=3	2	0	Other
Calsequestrin OS=Rattus norvegicus OX=10116 GN=Casq2 PE=1 SV=2	1	0	Other

SON DNA and RNA-binding protein OS=Rattus norvegicus OX=10116 GN=Son PE=1 SV=2	1	0	Other
RB-binding protein 6, ubiquitin ligase OS=Rattus norvegicus OX=10116 GN=Rbbp6 PE=1 SV=1	1	0	Other
RCG44245 OS=Rattus norvegicus OX=10116 GN=Zfp449 PE=3 SV=1	3	0	Other
C3-beta-c OS=Rattus norvegicus OX=10116 GN=C3 PE=1 SV=1	10	0	Other
Citrate synthase (Fragment) OS=Rattus norvegicus OX=10116 GN=CS PE=2 SV=1	4	0	Other
Developmentally-regulated GTP-binding protein 1 OS=Rattus norvegicus OX=10116 GN=Drg1 PE=1 SV=1	59	0	Other

Supplementary Table 10 Primer sequences for RT-qPCR

Species	Gene name	Forward primer (5'→3')	Reverse primer (3'→5')
mouse	Cardinal	AGAAACCTCTGAATGCCCAG	GATGTGATGCTTTTGTTCCTCG
mouse	Nppa	CACAGATCTGATGGATTTCAAGA	CCTCATCTTCTACCGGCATC
mouse	Nppb	GTCAGTCGTTTGGGCTGTAAC	AGACCCAGGCAGAGTCAGAA
mouse	Acta1	GCCCATCTATGAGGGCTATG	AATCTCACGTTCAGCTGTGG
mouse	Fbn1	CCTTCCTGTGGCTCCAGAT	GCTGCCCCCATTCATACA
mouse	Fn	CTTTGTGGTCTCATGGGTCTC	AGCAGGTCAGGAATGTTCAC
mouse	β-actin	GTGACGTTGACATCCGTAAAGA	GCCGGACTCATCGTACTCC
mouse	Gapdh	AGGTCGGTGTGAACGGATTTG	TGTAGACCATGTAGTTGAGGTCA
mouse	18S	TCCGACCATAAACGATGCCG	CAATCTGTCAATCCTGTCCGTGTC
mouse	Drg1	TCGAAGAGAACTCATCACTCCTAA	CTGTCTTGGCCACATCAAAA
mouse	Mhrt	GAGCATTTGGGGATGGTATAC	TCTGCTTCATTGCCTCTGTTT
mouse	Neat1	TGGCCCCTTTTGTTCATTAGC	TGGAAGGCCATTGTTTCAGG
mouse	Chaer	TCCAATGAGGGAAGCGAAGC	GTCCGATGCCAGTTCCAGTT
mouse	U6	GTGCTCGCTTCGGCAGCA	GGAACGCTTCACGAATTTGC
mouse	28S	GGTAGCCAAATGCCTCGTCAT	CCCTTGGCTGTGGTTTCG
mouse	Hprt	TGGCCCTCTGTGTGCTCAA	TGATCATTACAGTAGCTCTTCAGTCTGA
mouse	Myocd	GCAAGGGCAGAAACAGGTC	ATCTGAGCAGTTGGAATGGAC
rat	Nppa	CAACACAGATCTGATGGATTTCA	CCTCATCTTCTACCGGCATC
rat	Nppb	GTCAGTCGCTTGGGCTGT	CCAGAGCTGGGGAAAGAAG
rat	Acta1	AGCTATGAGCTGCCTGACG	GATCCCCGCAGACTCCATA
rat	Drg1	CATCTCTGCTCATCACCGCT	CTGGTAACTGGCCTTTGGGT
Human	CARDINAL	CCGTTTAACAAGATGCAACAGT	CATGGTGCTTTTCACTTCAGC
Human	β-actin	CCAACCGCGAGAAGATGA	CCAGAGGCGTACAGGGATAG

Supplementary Methods

Generation of *Cardinal*-KO mice

Cardinal-KO mice were generated by Biocytogen. In brief, mRNA of *Cas9* and 2 sgRNA (5’: GTATACGCTACCCTCCTGTT AGG and 3’: GAGGCCTCATTGACTAATGG AGG) were injected into the single cell fertilized eggs of C57BL/6 mice. The injected fertilized eggs were then transplanted into the surrogate female mice. F0 generation were obtained after 21 days of pregnancy. Genotyping was performed using DNA extracted from tail tips. F0 mice with a positive genotype were mated to wild-type mice and gave birth to the F1 generation. *Cardinal*-HET mice were crossed to generate *Cardinal*-KO mice. *Cardinal*-HET and WT were used as controls for *Cardinal*-KO mice. Primers for genotyping were:

Common_forward: CGATGGGTCAGCAACAACAAAACGG

WT_reverse: CTCAGGCAACCTGTCAAACGTGAAG

KO_reverse: ACCCTGGAGTGCAATGGCTACTTTC

In total, 9 founders were obtained in the experiment to generate the *Cardinal* mutant mouse line; we obtained mice demonstrating germline transmission (F1) from 6 (out of 9) founders. Offspring from 2 founders were used in this study and the progeny of both lines showed a similar phenotype.

AAV9 generation and injection

AAV9 generation and injection have been described previously (1). Briefly, *GFP*,

full-length *Cardinal,* and *Cardinal-as* were separately cloned into the ITR-containing AAV plasmid (Penn Vector Core P1967) harboring the chicken cardiac TNT promoter to generate pAAV9-cTnT-*Cardinal*, pAAV9-cTnT-*Cardinal-as* (control) and pAAV9-cTnT-*GFP* (control). AAV9 was packaged in HEK293T cells with AAV9: Rep-Cap and pHelper (pAd deltaF6, Penn Vector Core), then purified and concentrated by gradient centrifugation (2). AAV9 titer was determined by quantitative PCR. AAV9 virus (5×10^9 viral genomes [vg]/g) was injected into postnatal day 1 WT pups by subcutaneous injection.

Measurement of cardiac function by echocardiography

Echocardiography was performed as previously described (3) using a Visual Sonics Vevo 2100 Imaging System (Visual Sonics) with a 40-MHz MicroScan transducer (model MS-550D). Mice were lightly anesthetized with isoflurane (2.5% isoflurane for induction and 0.5% for maintenance). Recovery from anesthesia was achieved by reducing the isoflurane concentration with monitoring of the heart rate until stabilized above 500 bpm. Heart rate and LV dimensions, including diastolic and systolic wall thicknesses and LV end-diastolic and end-systolic chamber dimensions, were measured from 2D short-axis views under M-mode tracings at the level of the papillary muscle. LV mass and functional parameters, such as percentage of fraction shortening and ejection fraction, were calculated using the above primary measurements and accompanying software.

H&E staining, Sirius red/Fast green collagen staining, and Wheat Germ Agglutinin (WGA) staining

The staining was performed as previously described (3). Mouse heart tissues were dissected out, rinsed with PBS, and fixed in 4% PFA (pH 8.0) overnight. After dehydration through a series of ethanol baths, samples were embedded in paraffin wax according to standard laboratory procedures. 5 μm sections were stained with H&E. For Sirius red/Fast green collagen staining, sections were fixed with prewarmed Bouin's solution at 55°C for 1 hour and then washed in running water. Sections were first stained in 0.1% fast green solution for 10 minutes and then washed with 1% acetic acid for 2 minutes. After washing in tap water, sections were stained in 0.1% Sirius solution for 30 minutes. After staining, sections were dehydrated and cleared with xylene. The images were examined with a light microscope and quantified with ImageJ software.

For cell area quantification, heart sections were deparaffined and then incubated with fluorescein conjugated WGA (5μg/mL, ThermoFisher, W11262) at 4°C overnight. Cell membranes were visualized with a fluorescence microscope (Olympus; IX71). The cross-sectional area of cardiomyocytes was calculated with ImageJ.

Polysome profiling

HL-1 cells were cultured as previously described (4). When they reached 90% confluence, cells were pretreated with 100μg/mL cycloheximide for 5 minutes. After incubation, they were washed once with ice-cold PBS, then scraped off the culture dish

in PBS with 100μg/mL cycloheximide. Cells were pelleted by centrifuge at 1000 rpm (ThermoFisher, Sorvall ST 16R) at 4°C for 5 minutes. PBS was removed and cells were lysed in 1.2mL Polysome Extraction Buffer (50 mM MOPS, 15mM $MgCl_2$, 150mM NaCl, 100μg/mL cycloheximide, 0.5% Triton X-100, 1mg/mL heparin, 5ul/mL RNAsin [Promega], cOmplete protease inhibitor cock tail, 1mM benzamidine). After incubation for 10 minutes on ice, lysate was centrifuged for 13,000 g, 4°C, 10 minutes. The supernatant was collected for subsequent procedures.

A linear sucrose gradient of 10%-50% was made with Polysome Buffer (50mM MOPS, 15mM $MgCl_2$, 150mM NaCl, 100μg/mL cycloheximide) using a Density Gradient Fractionation System (BR-188, BRANDEL). The supernatant was loaded on top of the sucrose gradient, and centrifuged for 36,000 rpm (Beckman Coulter, Optima XPN-100) at 4°C for 150 minutes. The OD254 value along the gradient was captured by the Fractionation System; ribosome-free, 40S, 60S, monosome, and polysome fractions were collected according to the polysome profiling curve. RNA from each fraction was isolated using TRIzol (Invitrogen) as previously described (5). Equal volumes of each fraction were directly subjected to boiling with protein loading buffer and then western blotting.

Isolation and culture of adult cardiomyocytes

Adult cardiomyocytes were isolated by Langendorff retrograde perfusion (6). After dissecting from adult mice, hearts were submerged in Perfusion Buffer (113 mM NaCl, 4.7 mM KCl, 0.6 mM KH_2PO_4, 0.6 mM Na_2HPO_4, 1.2 mM $MgSO_4$, 10 mM Na-HEPES,

12 mM $NaHCO_3$, 10 mM $KHCO_3$, 30 mM taurine, 10 mM BDM, and 5.5 mM glucose, pH 7.25) to allow pumping out of any remaining blood. The aorta was lightly slipped onto an aortic cannula; the aorta was then clamped and ligated on the cannula. The heart was perfused with Digestion Buffer (1mg/mL type II collagenase [Worthington] in Perfusion Buffer) for approximately 40 minutes. The heart was pulled out from the cannula and transferred to a 60mm dish containing 3mL of fresh Digestion Buffer. Aorta, atria, great vessels, and any remaining connective tissue were trimmed. The ventricles were teased into small pieces with fine-tip forceps. Heart pieces were gently pipetted up and down for about 20 times and transferred to 5mL Stop Buffer (0.5% BSA in Perfusion Buffer) in a conical tube. The conical tube was rotated up and down several times to ensure effective stoppage. The solution was filtered through a 100μm cell strainer to remove any remaining tissue pieces. The filtered cell suspension was centrifuged for 5 minutes at 300 rpm (ThermoFisher, Fresco™ 21). Supernatant was removed and the pellets were resuspended using Calcium Solution I (12.5 μM $CaCl_2$ in Stop Buffer). The centrifugation and resuspension steps were repeated 3 times using Calcium Solution II to IV (100, 400, and 900μM $CaCl_2$ in Stop Buffer). These serial centrifugations separate cardiomyocytes from non-cardiomyocytes. After the last centrifugation, supernatant was removed and cardiomyocytes pellets were resuspended in Culture Medium (10 mM BDM, 0.5% BSA in M199 culture medium [Sigma-Aldrich]). Cells were plated on culture plates precoated with Laminin (2μg/cm^2) at 4°C overnight. The plate was placed in the cell culture incubator for 40 minutes to allow cardiomyocyte attachment, then culture medium was removed. The attached cells were

washed once with Culture Medium to remove any remaining unattached cells or debris. Fresh Culture medium with or without phenylephrine (PE) was added and the plate was returned to the incubator. Cardiomyocytes from *Cardinal*-HET mice were used as control for cardiomyocytes from *Cardinal*-KO mice.

Isolation and culture of neonatal rat ventricular cardiomyocytes (NRVCs)

Isolation and culture of NRVCs was performed as previously described (7). In brief, 1-3 day old neonatal Sprague-Dawley rats were decapitated using sterile scissors, and the chest was opened to harvest the heart. After trimming of atria, large vessels, and connective tissue, hearts were cut into pieces; NRVCs were isolated by repeated enzymatic dissociation (0.08% trypsin) at 37°C. Digestion was stopped by adding an equal amount of DMEM containing 10% FBS. All isolated cells were pre-plated for 1 h to remove noncardiomyocytes. Nonadherent cardiomyocytes were then plated on 0.5% gelatin-coated plates and cultured in DMEM (ThermoFisher, C11995500BT), supplemented with sodium pyruvate (1×), GlutaMAX (1×), and 10% FBS with antibiotics. 18 hours after plating, culture medium was changed to FBS-free medium for starvation. RNA interference or adenovirus infection was performed at the beginning of starvation. RNA interference was performed using Lipofectamine RNAiMAX (Invitrogen, 13778150) according to the protocol provided by the manufacturer. Adenovirus was added at a multiplicity of infection (MOI) of 10. After starvation for 24 hours, culture medium was replaced by fresh FBS-free medium with or without 50μM PE. Protein or RNA was harvested at the indicated time points. For IF

staining, cells were fixed 48 hours after stimulation. Sequence of siRNAs used in this study are listed below:

si-Drg1: GGUUGAAGGAAACAGAGUU, si-NC: UUCUCCGAACGUGUCACGU.

These siRNAs were produced by GenePharma.

Culture and transfection of 293T

293T cells were cultured in DMEM (ThermoFisher, C11995500BT) with 10% FBS, and passaged using 0.25% Trypsin (Gibco, 25200072). Transfection of 293T cells was performed using polyethylenimine (PEI) when cells reached 70-80% confluence. PEI and plasmid DNA were mixed in a 4:1 ratio in DMEM and incubated for 30 minutes at room temperature. The mixture was added to cultured cells drop by drop. Protein or RNA was harvested 48 hours after transfection.

Gene expression manipulation in HL-1 cells

HL-1 cells were cultured following the instructions provided by the manufacturer of Claycomb Medium (Sigma-Aldrich, 51800C). In brief, cells were cultured in Claycomb medium with 10% FBS, 0.1mM norepinephrine, and 2mM L-glutamine, and passaged using 0.05% Trypsin (Gibco, 25200072). RNA interference in HL-1 cells was performed by Lipofactamine RNAiMAX (Invitrogen, 13778150) according to the protocol provided by the manufacturer. The siRNA sequence for *Drg1* knock-down was GCAUUCUGGCUGAAUAUAATT, and the sequence for the negative control was UUCUCCGAACGUGUCACGU.

Lentivirus was used to generate a HL-1 cell line with stable knock-down of *Cardinal*. Sequences of short hairpin RNA were cloned into the plasmid pLKO.1 (Addgene, 8453). The recombinant pLKO.1 plasmids, pCMV-VSV-G (Addgene, 8454), and pCMV delta R8.2 (Addgene, 12263) were co-transfected into 293T cells using PEI. Media containing transfection reagents was discarded 8 hours after transfection, and replaced with fresh culture media. The culture media containing lentivirus was collected 48 hours after transfection and stored at -80℃. When HL-1 cells reached 80% confluence, 500μl of lentivirus media was added to every 2ml HL-1 culture media. HL-1 cells were cultured with lentivirus at 37℃ for 48 hours. Culture media with lentivirus was replaced by fresh culture media supplemented with 1 μg/mL of puromycin for selection. These cells were cultured at 37℃ for 72 hours to establish the HL-1 line with stable knock-down of *Cardinal*. Control lines were generated similarly. The shRNA sequence for *Cardinal* knock-down was:

CCGGGGGCTAGTTTCTGATAGTGAACTCGAGTTCACTATCAGAAACTAGCC
CTTTTTGAATT.

The shRNA sequence for the negative control was:

CCGGTTCTCCGAACGTGTCACGTCTCGAGACGTGACACGTTCGGAGAATTT
TTGAATT.

Single-molecule RNA FISH

Single-molecule RNA FISH was performed using the RNAscope™ 2.5 HD Assay – RED kit (Cat No.: 322350) with the probe RNAscope® Probe- Mm-Gm12295 (Cat

No: 832421) according to the protocol provided by the manufacturer. Experiment RNAscope® Probe- Mm-*Neat1* (Cat No: 440351) was performed in parallel as a positive control. After FISH, immunofluorescent (IF) of myosin heavy chain by MF20 antibody (Developmental Studies Hybridoma Bank) was performed to visualize cardiomyocytes. The protocol provided by the RNAscope manufacturer for IF staining following RNA FISH on plated cells was used.

Preparation of nuclear and cytoplasmic RNAs

The nuclear and cytoplasmic portion of HL-1 cells and adult cardiomyocytes were separated using the PARIS™ Kit (Invitrogen, AM1921). The RNA extracted from these fractions was used for reverse transcription and RT-qPCR.

Preparation of adenovirus

Adenovirus was prepared using the AdMAX system. The full-length sequence of *Cardinal* was cloned into the shuttle vector with a minimal CMV promoter. The empty shuttle vector was used to produce control virus. Shuttle vector and backbone vector were co-transfected to 293A cells. After amplification, adenovirus was collected, purified, and concentrated by gradient centrifugation with the ViraTrap Adenovirus Purification Miniprep Kit, according to the manufacturer's instructions (Biomiga; V1160).

Immunofluorescent staining

NRVCs were fixed in 4% PFA at room temperature for 15 minutes, washed by PBS, permeabilized and blocked in PBS containing 0.1% Triton X-100 and 10% FBS for 1 hour, incubated in anti-α-actinin (1:200, Abcam, ab9465) diluted in PBS with 0.1% Triton X-100 and 10% FBS at 4°C overnight. After washing with PBST (0.1% Tween 20), cells were incubated in anti-mouse secondary antibodies conjugated with Alexa 488 (1:1,000, ThermoFisher, A-11029) or Alexa 594 (1:1,000, ThermoFisher, A-11032), together with DAPI (0.1 mg/mL, Sigma, D9542) in PBST for 1 h. Images were captured by a fluorescence microscope (Olympus; IX71) and analyzed by ImageJ.

FlUorescent Non–Canonical Amino acid Tagging (FUNCAT) assay

FUNCAT (8) was performed using the Click-iT® HPG Alexa Fluor® Protein Synthesis Assay Kit (Invitrogen, C10429) following the protocol provided by the manufacturer. In brief, cells were incubated with 50μM homopropargylglycine (HPG) in L-methionine-free medium for 30 minutes. Newly synthesized proteins within this period incorporated HPG into the polypeptide chain. After fixation and permeabilization, HPG on newly synthesized protein was labeled by fluorescein conjugated azide by a "Click Reaction". After additional nuclear staining with DAPI, images were captured using a fluorescence microscope (Olympus; IX71).

Identification of interacting proteins using RNA pull-down and Mass Spectrometry

The first two sets of pull-down experiments used *Cardinal* RNA; for controls,

Cardinal-as, as well as lncRNA *Nppa-as* and *Linc-p21* (two unrelated lncRNAs), were used. These RNAs were *in vitro* transcribed using the MEGAscript™ T7 Kit (Invitrogen, AM1333) and purified by MEGAclear™ Transcription Clean-Up Kit (Invitrogen, AM1908). The RNA was labeled by desthiobiotin with the Pierce™ RNA 3' End Desthiobiotinylation Kit (Thermo Scientific, 20163). RNA pull-down with the biotinylated RNA probes and heart lysates were performed using Pierce™ Magnetic RNA-Protein Pull-Down Kit (Thermo Scientific, 20164).

For the third set of *Cardinal* pull-down reactions, a biotinylated single-stranded DNA fragment was synthesized from the *Cardinal* complementary (i.e., non-coding) strand by BGI Genomics. A biotinylated single stranded sense DNA fragment was used as a control probe. HL-1 cells were lysed with Lysis Buffer (50mM Tris[pH7.4], 150mM NaCl, 1% NP-40, 0.25% sodium deoxycholate) supplemented with cOmplete protease inhibitor cocktail (Roche) and 5uL/mL RNAsin (Promega). The lysate was centrifuged at 12,000 rpm (ThermoFisher, Sorvall ST 16R) at 4°C for 10 minutes. The supernatant was incubated with 2μg of the DNA probe at 4°C overnight with rotation. Streptavidin conjugated magnetic beads (Pierce, 88816) were then added to the solution, and incubated at 4°C with rotation for 2 hours. The supernatant was removed and beads were washed with Lysis Buffer and protein was eluted by boiling with protein loading buffer.

The samples from the first 2 sets of pull-down experiments were sent to Taplin Mass Spectrometry Facility (Harvard) for mass spectrometry. After digestion by trypsin, samples were loaded on a nano-scale reverse-phase HPLC capillary column. The

eluted peptides were subjected to electrospray ionization and then analyzed with an LTQ Velos ion-trap mass spectrometer. Peptide sequences were determined by matching the acquired fragmentation pattern with protein databases using the software program, SEQUEST.

The samples from the third set of pull-down experiment were sent to BGI genomics for analysis by mass spectrometry. Proteins were digested by trypsin and separated by the UltiMate3000 RSLCnano ultra-high performance liquid system. The peptides were then analyzed by the Thermo ScientificTM Q ExactiveTM mass spectrometer. Data Dependent Acquisition (DDA) procedure was used for data acquisition. The resulting MS/MS data were processed using MASCOT2.3.0. Tandem mass spectra were searched against uniprot20160315. Trypsin was specified as the cleavage enzyme allowing up to 2 missing cleavages. Mass error was set to 15 ppm for precursor ions and 20 mmu for fragment ions. Carbamidomethyl on Cys were specified as fixed modification, Gln->pyro-Glu (N-term Q) and oxidation on Met was specified as variable modification. Peptide ion score was set > 20.

Cardinal and *Cardinal*-as pull-down in 293T cells was performed using the same procedures. The proteins were subjected to western blotting instead of mass spectrometry. The sequences of DNA probes were:

anti-*Cardinal*-1:

Biotin-CATTCCTTGAGAACCTCCTCCTGTCCTTCCCTGTGTTTCCTGCCCTTTCACTATCAGAAACTAG

CCCACT

anti-*Cardinal*-2:

Biotin-TATCAGGTGACCTCACAAATCATATGAACATTACCTCATTAACCCAGGATGCTTACTACGGCTA
AGGCAT

anti-*Cardinal*-3:

Biotin-TGAGAAGGTCCACCATTGACCTGTAGTTTGCTCAGCCAGTGACTCAGATTCTTAGCCCTTTCCT
AGTCTC

anti-*Cardinal-as*-1:

Biotin-ATGCCTTAGCCGTAGTAAGCATCCTGGGTTAATGAGGTAATGTTCATATGATTTGTGAGGTCAC
CTGATA

anti-*Cardinal-as*-2:

Biotin-GAGACTAGGAAAGGGCTAAGAATCTGAGTCACTGGCTGAGCAAACTACAGGTCAATGGTGGACCTT
CTCA

and anti-*Cardinal-as*-3:

Biotin-AGTGGGCTAGTTTCTGATAGTGAAAGGGCAGGAAACACAGGGAAGGACAGGAGGAGGTTCTCAAG
GAATG

RNA immunoprecipitation

293T cells were harvested using Lysis Buffer (50mM Tris[pH7.4], 150mM NaCl, 1% NP-40, 0.25% sodium deoxycholate) supplemented with cOmplete protease inhibitor cocktail (Roche) and 5uL/mL RNAsin (Promega). The lysate was centrifuged at 12,000 rpm (ThermoFisher, Sorvall ST 16R) at 4° for 10 minutes. The supernatant was incubated with anti-HA magnetic beads (Thermo Scientific, 88837) at 4°C with rotation for 2 hours. The supernatant was removed, then beads were washed with Lysis

Buffer 3 times. RNA attached to beads was isolated using TRIzol (Invitrogen) and used for reverse transcription and RT-qPCR.

Co-immunoprecipitation

Cells were harvested using Lysis Buffer (50mM Tris[pH7.4], 150mM NaCl, 1% NP-40, 0.25% sodium deoxycholate) supplemented with cOmplete protease inhibitor cocktail (Roche). The lysate was centrifuged at 12,000 rpm (ThermoFisher, Sorvall ST 16R) at 4° for 10 minutes. 10μl supernatant was kept as input. The remaining supernatant was incubated with anti-HA magnetic beads (Thermo Scientific, 88837) or anti-FLAG magnetic beads (Thermo Scientific, A36797) at 4°C with rotation for 2 hours. Supernatant was removed, and beads were washed 3 times with Lysis Buffer. The protein attached to the beads were eluted by boiling with protein loading buffer and subjected to western blotting and mass spectrometry (BGI genomics).

For endogenous co-immunoprecipitation, cells were harvested using Lysis Buffer with cOmplete protease inhibitor cocktail, followed by centrifugation. 10μl supernatant was kept as input. The remaining supernatant and corresponding antibodies were incubated with rotation at 4°C overnight. Protein A/G PLUS-Agarose beads were then added to the solution, which was incubated with rotation at 4°C for 1 hour. The agarose beads were pelleted by centrifugation at 3000 rpm (ThermoFisher, Sorvall ST 16R) at 4°C for 1 minute. Supernatant was removed, and beads were washed 3 times with Lysis Buffer. Protein attached to beads was eluted by boiling with protein loading buffer and subjected to western blotting.

Northern blotting

Northern blotting was performed as previously described (9, 10). 10 μg of total RNA was loaded in each lane. DNA probes were amplified with following primers:

Cardinal-F: AGAAACCTCTGAATGCCCAG

Cardinal-R: GATGTGATGCTTTTGTTCCTCG

Gapdh-F: AGGTCGGTGTGAACGGATTTG

Gapdh-R: TGTAGACCATGTAGTTGAGGTCA

Probes were labeled with α-^{32}P-dATP (NEN, Boston, MA) using a random primed DNA labeling kit (Boehringer Mannheim, Indianapolis, IN). The hybridization was performed at 68°C overnight in ExpressHyb hybridization solution (Clontech, Palo Alto, CA). The filter was then washed twice in 2× SSC, 0.05% SDS at room temperature for 30 minutes each, followed by 30 minutes at 50°C in 0.1× SSC, 0.1% SDS. The filter was then imaged with a phosphorimager (GE Healthcare).

RNA isolation and RT-qPCR

RNA was isolated from cells or tissues using TRIzol Reagent (Invitrogen). For RT-qPCR, 1μg of RNA was reversed-transcribed to cDNA using PrimeScript RT Reagent Kit (TAKARA) in a 20μL reaction system. In each analysis, 0.1μL of cDNA solution was used for RT-qPCR using TB Green® Premix Ex Taq™ II (TAKARA).*Gapdh*, *β-actin*, or *18s* was used as internal control. Sequences of primers for RT-qPCR is summarized in Suppl. Table 10.

Western blotting

Protein in cells or tissues was extracted using RIPA buffer (Beyotime) with cOmplete protease inhibitor cocktail (Roche) and denatured at 95°C for 10 minutes. Samples were subsequently analyzed by SDS-PAGE and transferred to 0.45 μm PVDF membranes. After blocking with 5% BSA in Tris-buffered saline with Tween-20 (TBST) for 1 hour at room temperature, the membrane was incubated with solution containing primary antibody at 4°C overnight, washed by TBST, incubated with corresponding secondary antibody at room temperature for 1 hour, and washed again with TBST. Specific protein bands were visualized using the Immobilon Western chemiluminescent HRP substrate (Millipore; WBKLS0500) by ImageQuant LAS4000 Mini (GE Healthcare).

Primary antibodies used: anti-RPL35 (1:1000, Signalway Antibody, 34357), anti-GAPDH (1:5000, Proteintech, 60004), anti-puromycin (1:5000, Millipore, MABE343), anti-HA (1:1000, Cell Signaling Technology, 3724S), anti-DRG1 (1:200, Santacruz, sc-390030), anti-DRG1 (1:1000, Invitrogen, PA5-76585), anti-FLGA (1:1000, Proteintech, 20543-1-AP), anti-ZC3H15/DFRP1 (1:1000, Invitrogen, PA5-56639).

Secondary antibodies used: anti-mouse IgG (1:5000, Proteintech, SA00001-1), anti-rabbit IgG (1:5000, SA00001-2).

Analysis of RNA-seq data

Total RNA from the apex of heart was isolated using TRIzol (Invitrogen) and poly

A-selected libraries were generated with the TruSeq RNA Library Preparation Kit (Illumina). The RNA-sequencing was processed by GENEWIZ using an Illumina platform (paired-ended, 150bp read-length). FASTQ files were extracted, and sequencing adapters and low-quality reads were removed by Cutadapt (v.2.3). Quality of the processed FASTQ files were checked by FastQC (Babraham Bioinformatics), then aligned to the mouse genome (Esembl GRCm38 genome obtained from GENCODE) using HISAT2 (v.2.1.0). Heatmaps were generated with the "pheatmap" package in R (v.4.3.1) and principal component analysis (PCA) plot was generated by the "factoextra" package. EdgeR (v.3.26.1) was used to identify differentially expressed genes. Gene Set Enrichment Analysis was performed with the GSEA software (v.4.2.3). Deposited Gene Expression Omnibus (GEO) RNA-seq genomic data (GSE210985) are available and contained in the Source Data.

Analysis of Ribo-seq data

Heart tissues were snap frozen in liquid nitrogen and ground to power in liquid nitrogen with a mortar and pestle, then dissolved in 400μL of lysis buffer. Cells were triturated ten times through a 26-G needle. The lysate was centrifuged at 20,000 g for 10min at 4℃, and the supernatant was collected. To prepare ribosome footprints (RFs), 7.5μL of RNase I and 5μL of DNase I were added to 300μL of lysate to incubate for 45 min at room temperature with gentle mixing on a Nutator mixer. Nuclease digestion was stopped by adding 10μL of SUPERase·In RNase inhibitor. 100μL of lysate was added to a size exclusion column (illustra MicroSpin S-400 HR Columns; GE

Healthcare; catalog no. 27-5140-01), centrifuged at 600 g for 2 min, and eluted with 10μL 10% SDS. RFs were isolated from the solution using the RNA Clean and Concentrator-25 kit (Zymo Research;R1017). rRNA was removed using DNA probes complementary to rRNA sequences, which were then digested by RNase H and DNase I. Ribo-seq libraries were constructed using NEBNext® Multiple Small RNA Library Prep Set for Illumina® (catalog no.E7300S, E7300L). The 140-160bp size PCR products were enriched to generate a cDNA library and sequenced using Illumina HiSeqTM 2500 by Gene Denovo Biotechnology Co. (Guangzhou, China).

After raw Ribo-seq data were obtained, Cutadapt was used to remove adaptors and low-quality reads. Bowtie2 was used to remove rRNA and tRNA sequences. STAR was used to align the remaining reads to the genome. RiboCode was used to assure 3nt periodicity of the mapped reads and quantify read counts mapped to coding sequence regions of genes. Fold-changes of genes between samples were calculated by EdgeR.

The raw sequence data reported in this paper have been deposited to the Genome Sequence Archive in National Genomics Data Center, China National Center for Bioinformation / Beijing Institute of Genomics, Chinese Academy of Sciences (GSA: CRA014575) and are publicly accessible at https://ngdc.cncb.ac.cn/gsa.

Analysis of publicly available sequencing data

Publicly available RNA-seq data were analyzed using the same pipeline described above. For Ribo-seq data, in order to assess the association of lncRNA with ribosome instead of translation, Ribo-seq reads were analyzed directly without evaluating

periodicity, and read counts quantification were not limited to the coding sequence regions using StringTie. For Chromatin Immunoprecipitation (CHIP)-seq data, Bowtie2 was used for genome alignment. Bam files or Bigwig files generated during the analysis pipeline were used for visualization in IGV (v.2.11.2) software.

Mass spectrometry for evaluation of proteomic alteration

To evaluate proteomic alteration, label-free mass spectrometry was performed by TPM BIO. Heart tissues were snap frozen in liquid nitrogen and ground to power in liquid nitrogen. Four volumes of lysis buffer (1% Triton X-100, 1% protease inhibitor cocktail) were added to the powder, followed by sonication for three minutes on ice using a high intensity ultrasonic processor. The remaining debris was removed by centrifugation at 12,000 g at 4 °C for 10 min. The supernatant was collected and the protein concentration was determined using a BCA kit according to the manufacturer's instructions. TCA was slowly added to the samples until a final concentration of 20% (m/v) to precipitate protein. After incubation for 2 hours, the precipitate was collected by centrifugation at 4500 g for 5 min at 4°C. The protein sample was then redissolved in 200 mM TEAB and ultrasonically dispersed, after washing 3 times with acetone. Trypsin was added at 1:50 trypsin-to-protein mass ratio and incubated overnight. The sample was reduced with 5 mM dithiothreitol for 30 min at 56 °C and alkylated with 11 mM iodoacetamide for 15 min at room temperature in darkness. Finally, the peptides were desalted by Strata X SPE column. The tryptic peptides were dissolved in solvent A, directly loaded onto a home-made reversed-phase analytical column and separated

by gradients using an Easy-nLC1000 UHPLC system. The peptides were subjected to capillary source followed by timsTOF Pro mass spectrometry. Precursors and fragments were analyzed at the TOF detector. The resulting MS/MS data were processed using MaxQuant search engine (v.1.6.15.0). Tandem mass spectra were searched against Mus_musculus_10090_SP_20230103.fasta (17132 entries) concatenated with reverse decoy and contaminants database. Trypsin/P was specified as cleavage enzyme allowing up to 2 missing cleavages. Minimal peptide length was set as 7 and maximal number of modification per peptide was set as 5. The mass tolerance for precursor ions was set as 20 ppm in first search and 20 ppm in main search, and the mass tolerance for fragment ions was set as 20 ppm. Carbamidomethyl on Cys was specified as fixed modification, and acetylation on protein N-terminal and oxidation on Met were specified as variable modifications. False discovery rate (FDR) of protein, peptide and PSM was adjusted to < 1%. For comparison between KO;TAC and Ctrl;TAC groups, fold change was calculated as the ratio of mean of label-free quantification (LFQ) intensities. The difference was tested using a T test with the Benjamini-Hochberg correction. Any protein with fold change > 1 and false discovery rate (FDR) < 0.05 was regarded as up-regulated. The mass spectrometry proteomics data have been deposited to the ProteomeXchange Consortium via the iProX partner repository with the dataset identifier PXD048782: https://proteomecentral.proteomexchange.org

Reference

1. Guo H, Lu YW, Lin Z, Huang ZP, Liu J, Wang Y, et al. Intercalated disc protein

Xinbeta is required for Hippo-YAP signaling in the heart. *Nature communications.* 2020;11(1):4666.

2. Ding J, Lin ZQ, Jiang JM, Seidman CE, Seidman JG, Pu WT, et al. Preparation of rAAV9 to Overexpress or Knockdown Genes in Mouse Hearts. *J Vis Exp.* 2016;(118)(118):54787.
3. Huang ZP, Kataoka M, Chen J, Wu G, Ding J, Nie M, et al. Cardiomyocyte-enriched protein CIP protects against pathophysiological stresses and regulates cardiac homeostasis. *J Clin Invest.* 2015;125(11):4122-34.
4. Claycomb WC, Lanson NA, Jr., Stallworth BS, Egeland DB, Delcarpio JB, Bahinski A, et al. HL-1 cells: a cardiac muscle cell line that contracts and retains phenotypic characteristics of the adult cardiomyocyte. *Proc Natl Acad Sci U S A.* 1998;95(6):2979-84.
5. Panda AC, Martindale JL, and Gorospe M. Polysome Fractionation to Analyze mRNA Distribution Profiles. *Bio Protoc.* 2017;7(3):e2126.
6. Li D, Wu J, Bai Y, Zhao X, and Liu L. Isolation and culture of adult mouse cardiomyocytes for cell signaling and in vitro cardiac hypertrophy. *J Vis Exp.* 2014;(87):51357.
7. Yan Y, Tang R, Li B, Cheng L, Ye S, Yang T, et al. The cardiac translational landscape reveals that micropeptides are new players involved in cardiomyocyte hypertrophy. *Mol Ther.* 2021;29(7):2253-67.
8. Dieterich DC, Hodas JJ, Gouzer G, Shadrin IY, Ngo JT, Triller A, et al. In situ visualization and dynamics of newly synthesized proteins in rat hippocampal neurons. *Nat Neurosci.* 2010;13(7):897-905.
9. Wang D, Chang PS, Wang Z, Sutherland L, Richardson JA, Small E, et al. Activation of cardiac gene expression by myocardin, a transcriptional cofactor for serum response factor. *Cell.* 2001;105(7):851-62.
10. Wang DZ, Reiter RS, Lin JL, Wang Q, Williams HS, Krob SL, et al. Requirement of a novel gene, Xin, in cardiac morphogenesis. *Development.* 1999;126(6):1281-94.
