# Supplementary material for: The long noncoding RNA CARDINAL attenuates cardiac hypertrophy by modulating protein translation
Source: J Clin Invest. 2024 May 14;134(13):e169112. doi: 10.1172/JCI169112 (PMC11213465; doi:10.1172/JCI169112)

Figure 1F

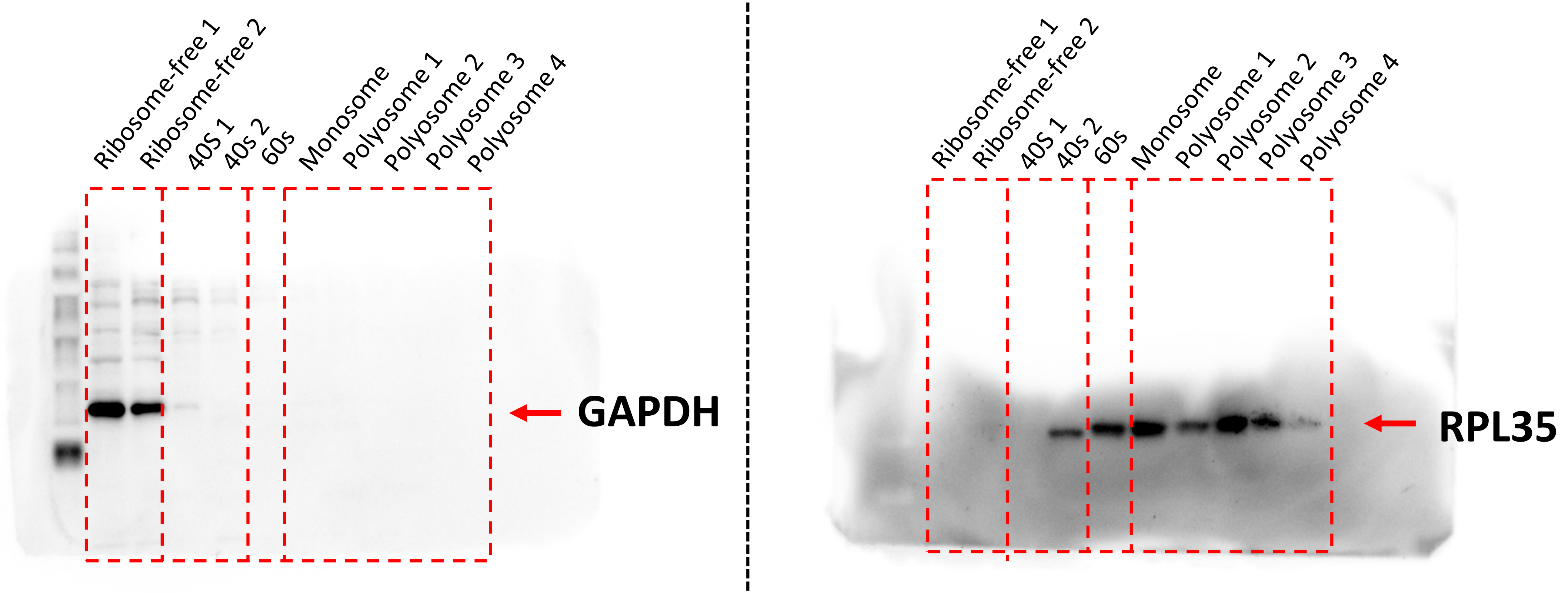
Ribosome-free 1
Ribosome-free 2
40S 1
40s 2
60s
Monosome
Polyosome 1
Polyosome 2
Polyosome 3
Polyosome 4
GAPDH
Ribosome-free 1
Ribosome-free 2
40S 1
40s 2
60s
Monosome
Polyosome 1
Polyosome 2
Polyosome 3
Polyosome 4
RPL35

Figure 1H

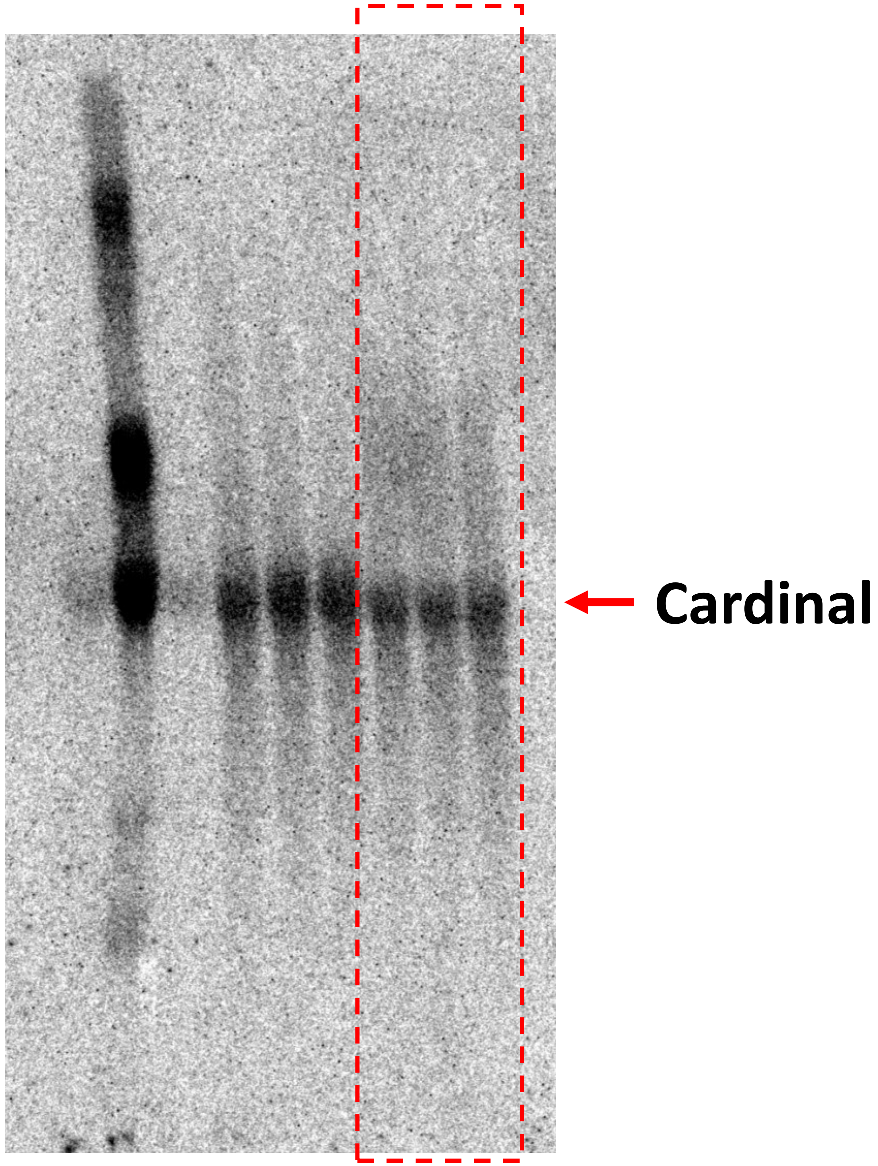

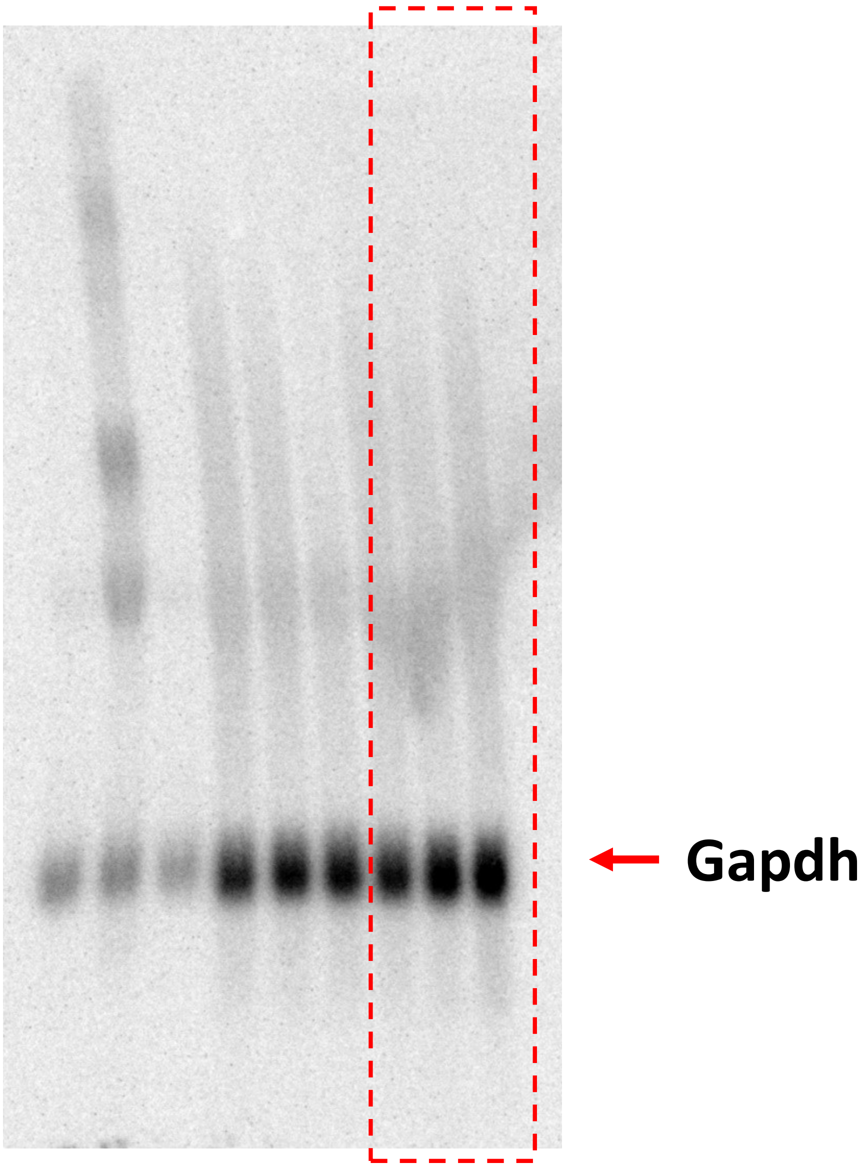

Figure 2B

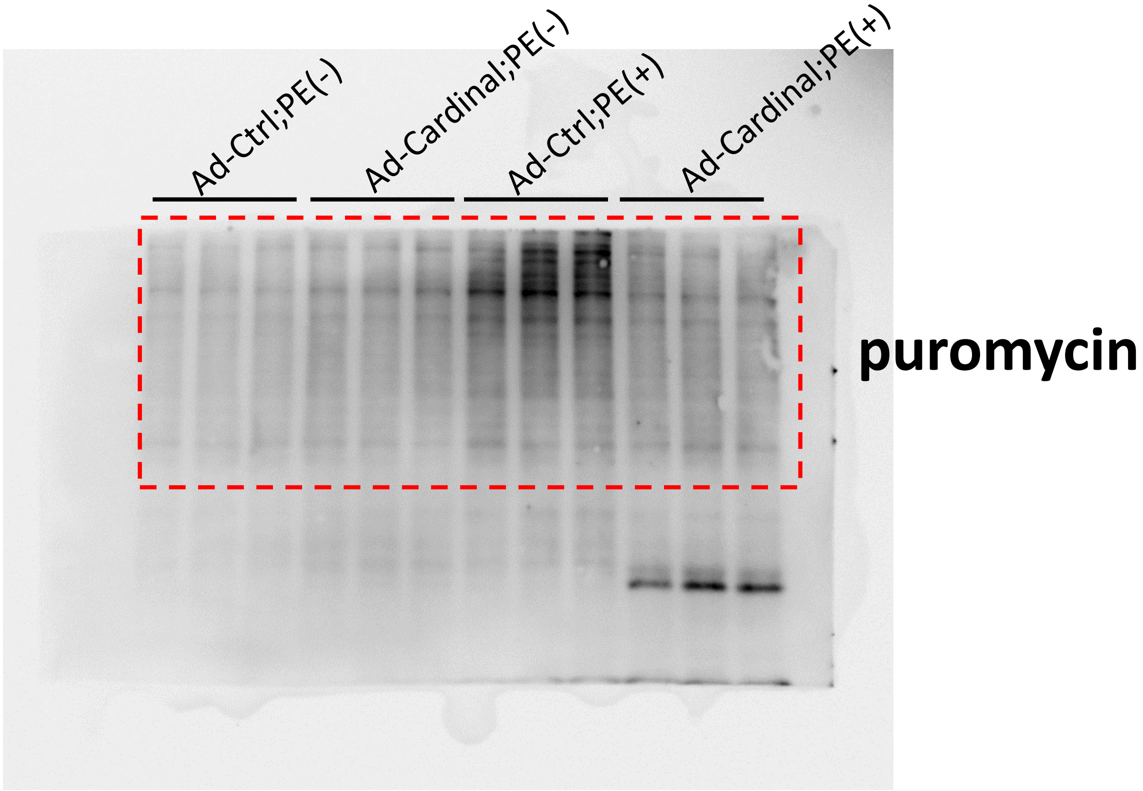

Ad-Ctrl;PE(-)
Ad-Cardinal;PE(-)
Ad-Ctrl;PE(+)
Ad-Cardinal;PE(+)
puromycin

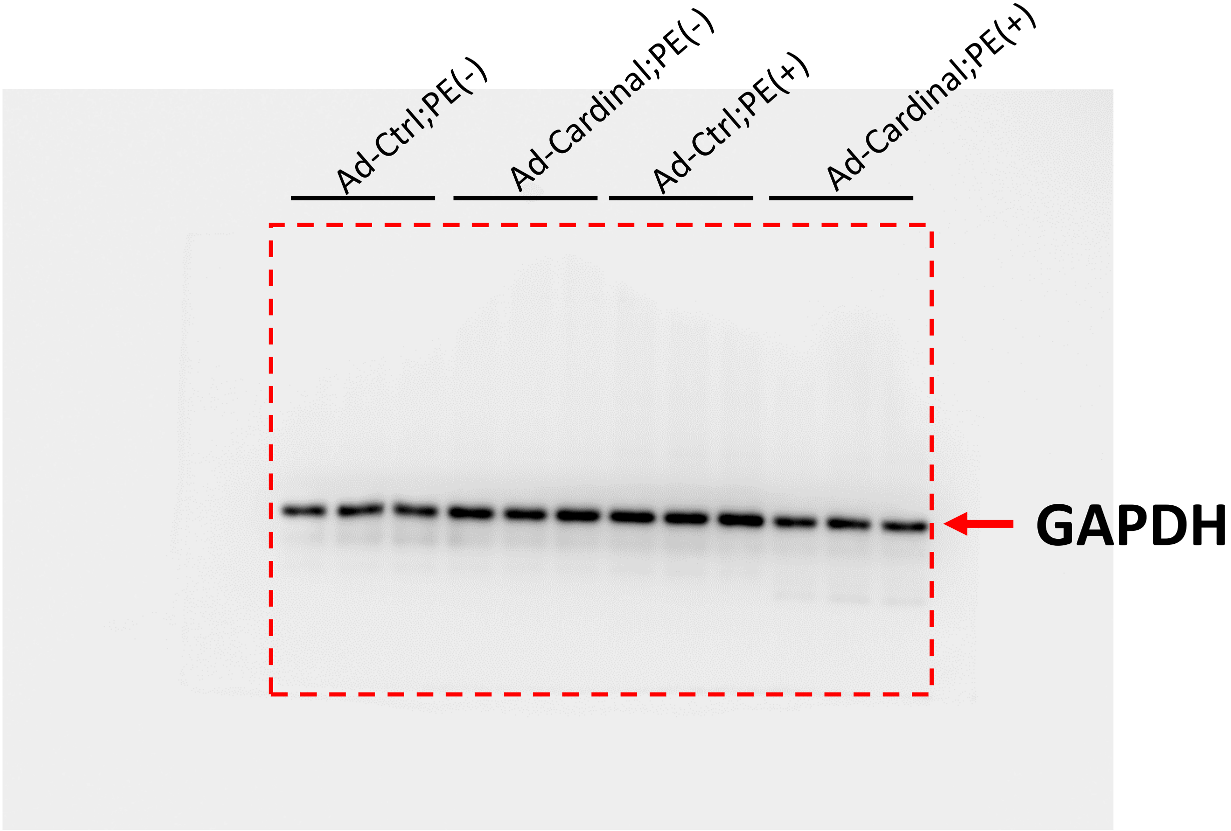

Ad-Ctrl;PE(-)
Ad-Cardinal;PE(-)
Ad-Ctrl;PE(+)
Ad-Cardinal;PE(+)
GAPDH

Figure 2F

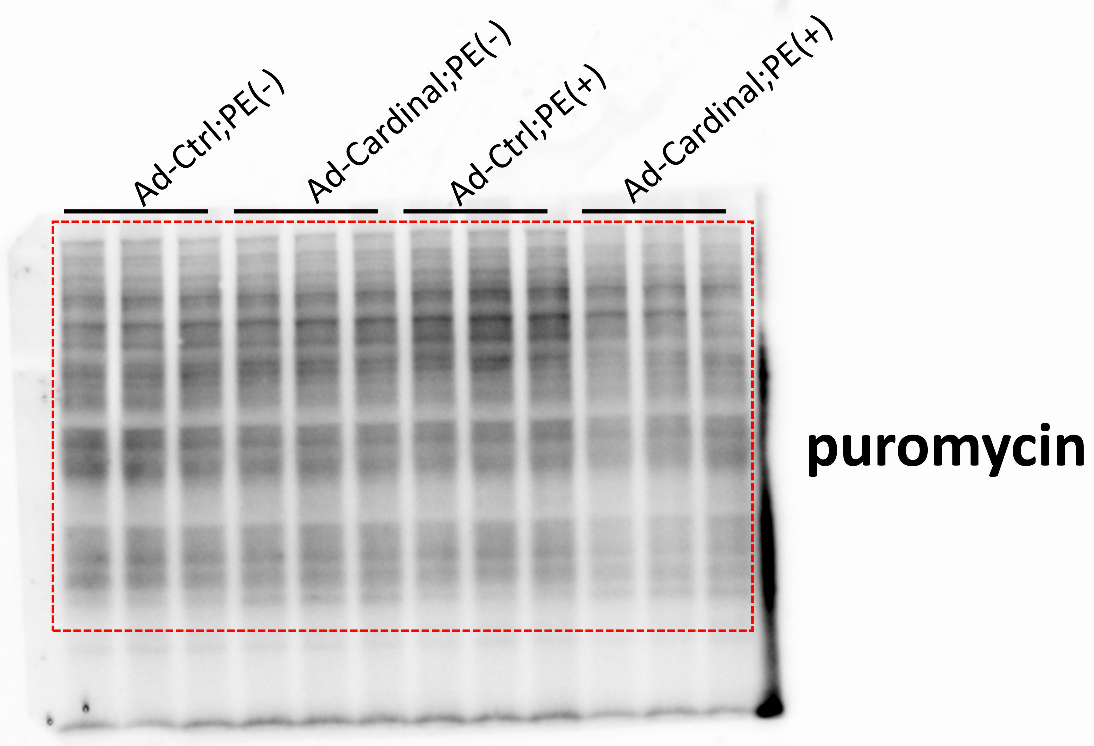
Ad-Ctrl;PE(-)
Ad-Cardinal;PE(-)
Ad-Ctrl;PE(+)
Ad-Cardinal;PE(+)
puromycin

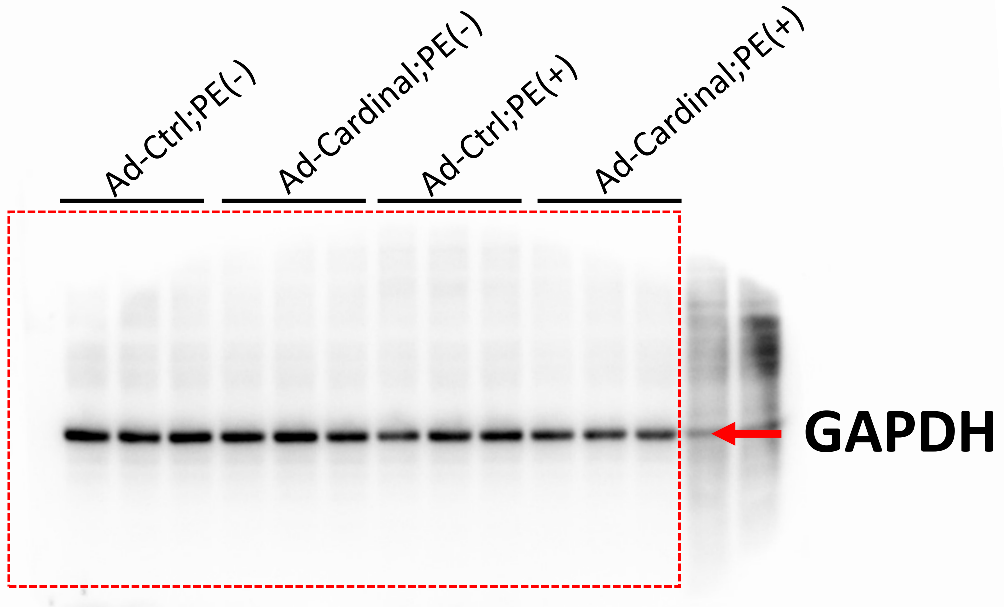
Ad-Ctrl;PE(-)
Ad-Cardinal;PE(-)
Ad-Ctrl;PE(+)
Ad-Cardinal;PE(+)
GAPDH

Figure 4J

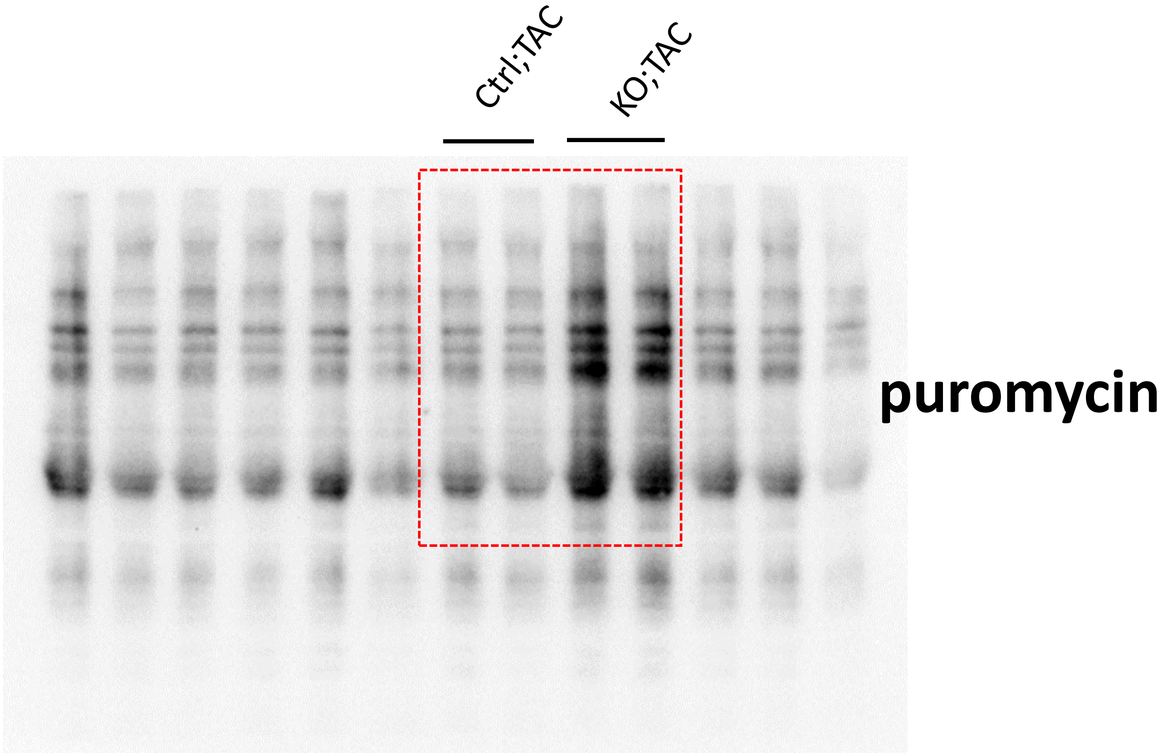

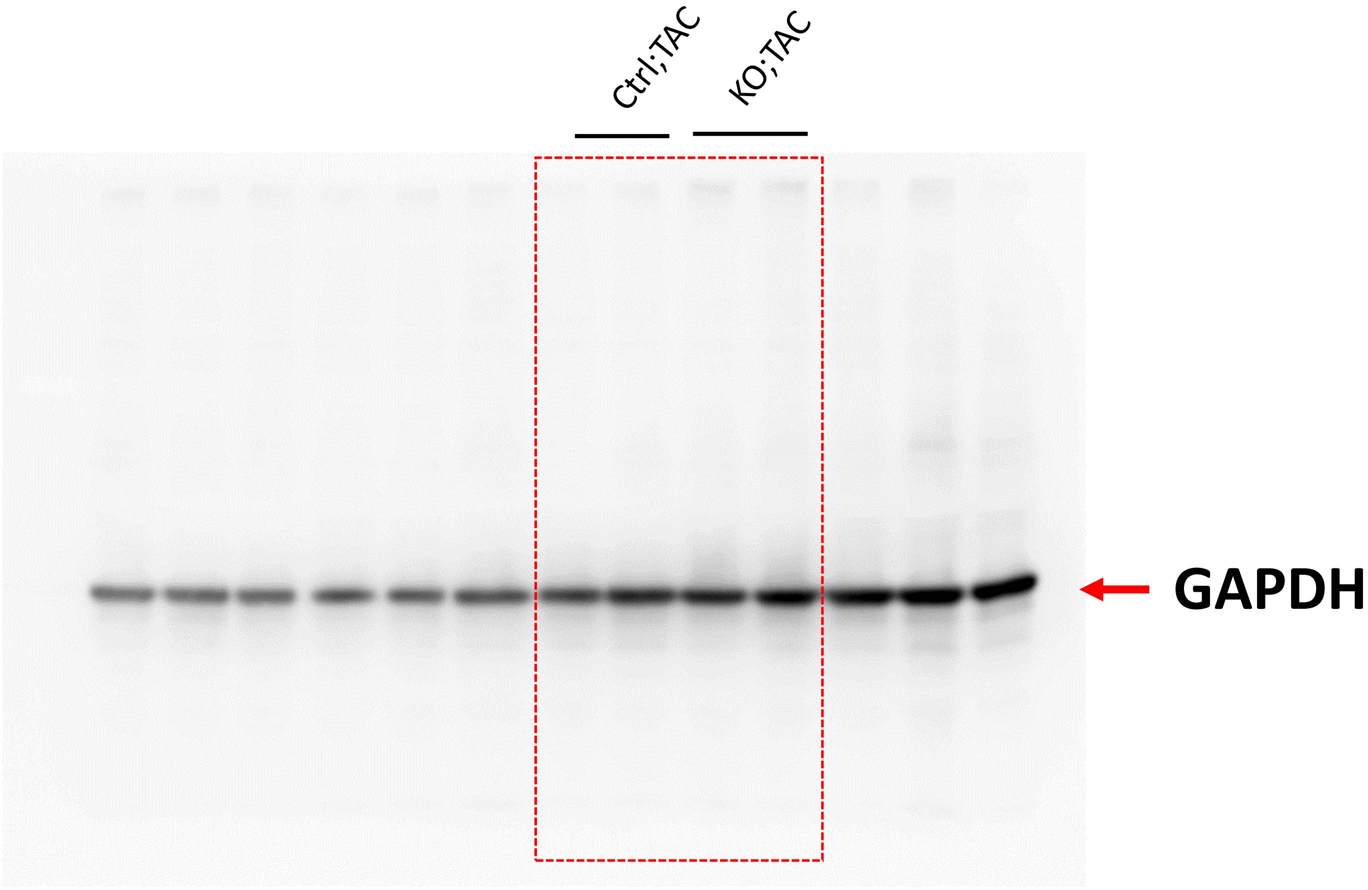

Figure 4L

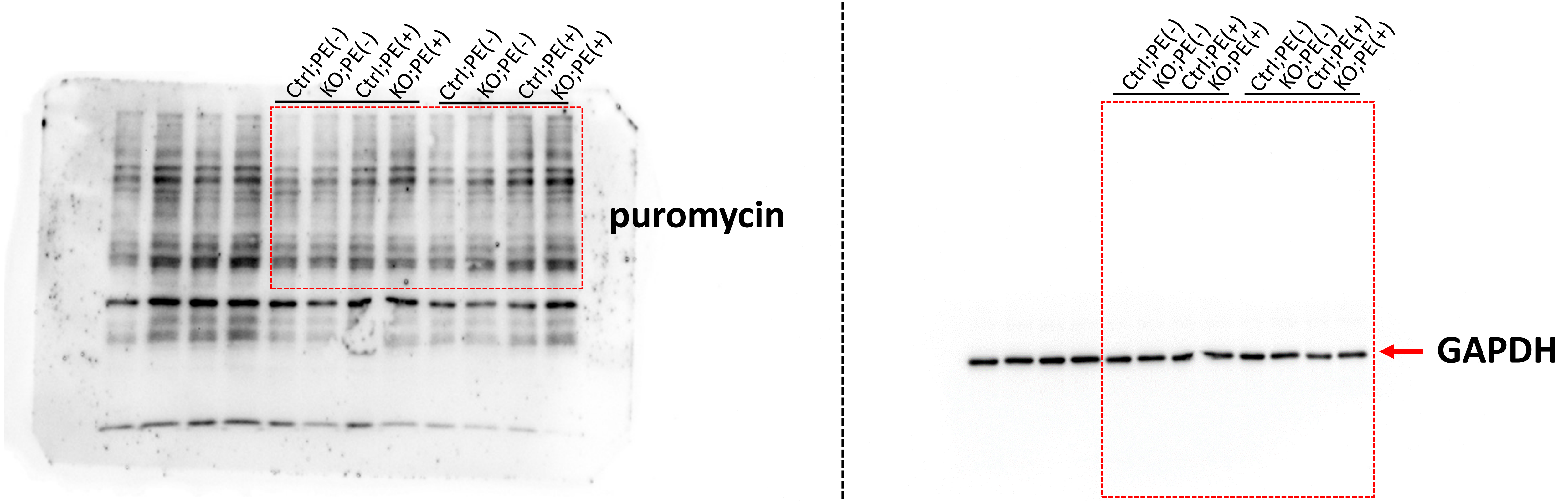
Ctrl;PE(-)
KO;PE(-)
Ctrl;PE(+)
KO;PE(+)
Ctrl;PE(-)
KO;PE(-)
Ctrl;PE(+)
KO;PE(+)
puromycin
Ctrl;PE(-)
KO;PE(-)
Ctrl;PE(+)
KO;PE(+)
Ctrl;PE(-)
KO;PE(-)
Ctrl;PE(+)
KO;PE(+)
GAPDH

Figure 6D

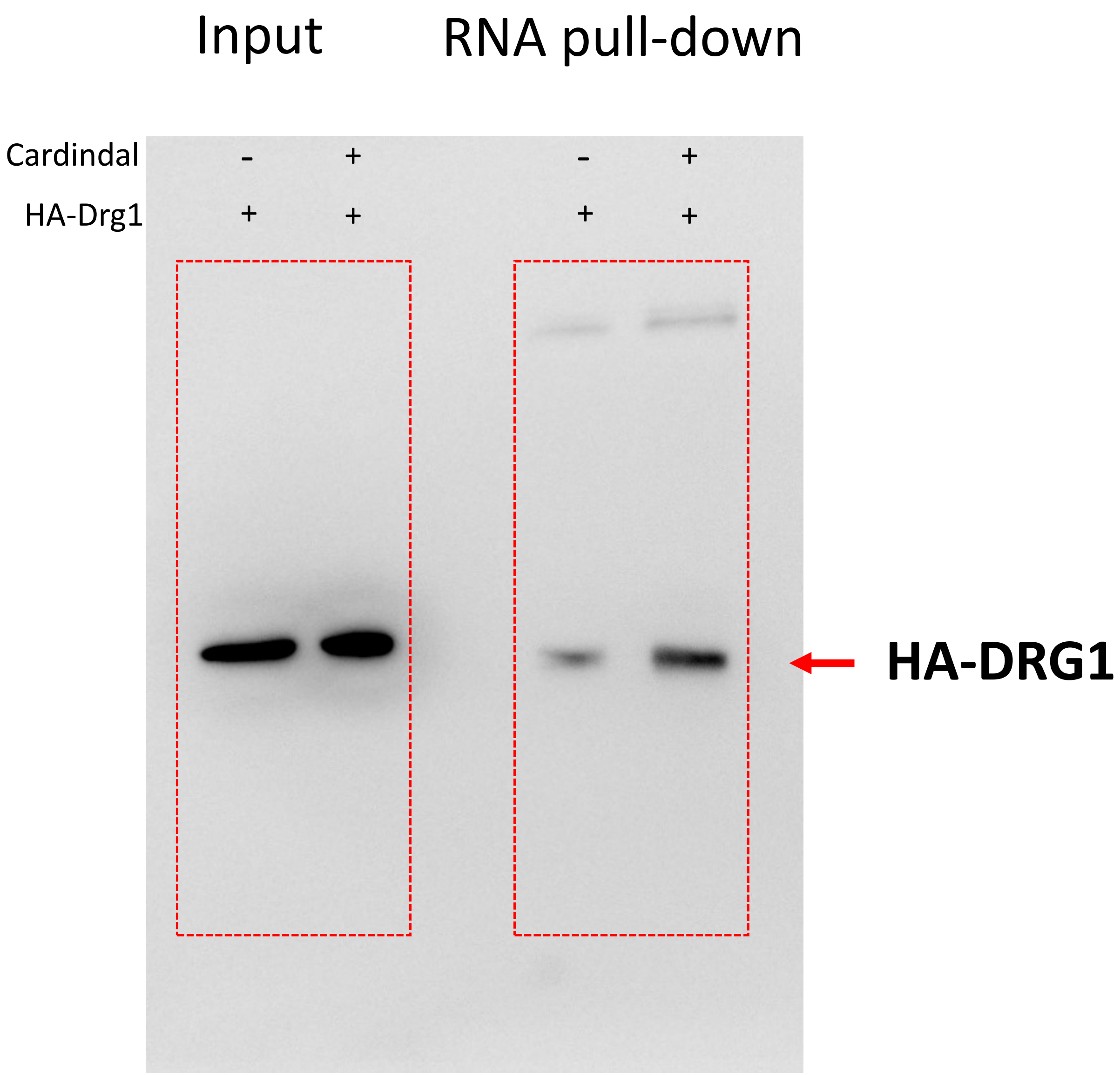

Figure 6G

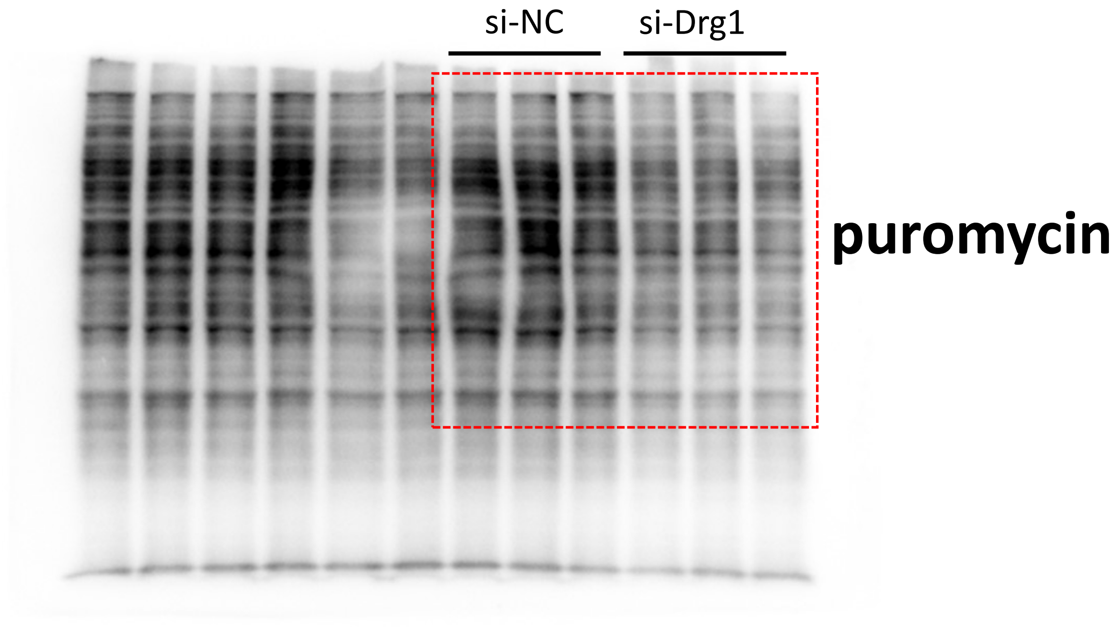

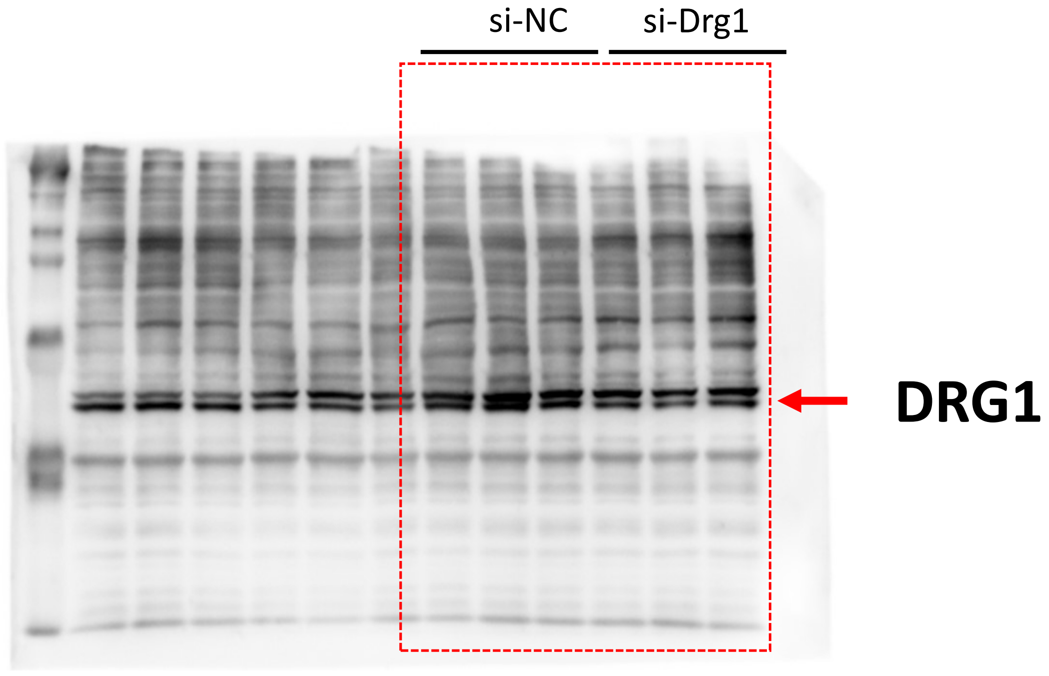

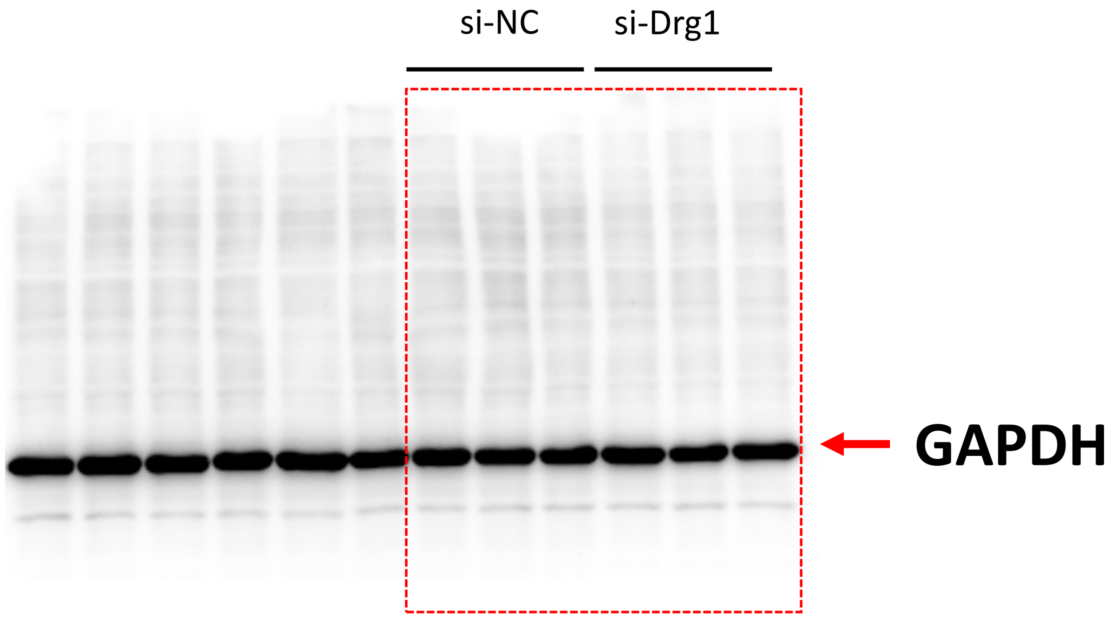

Figure 6I

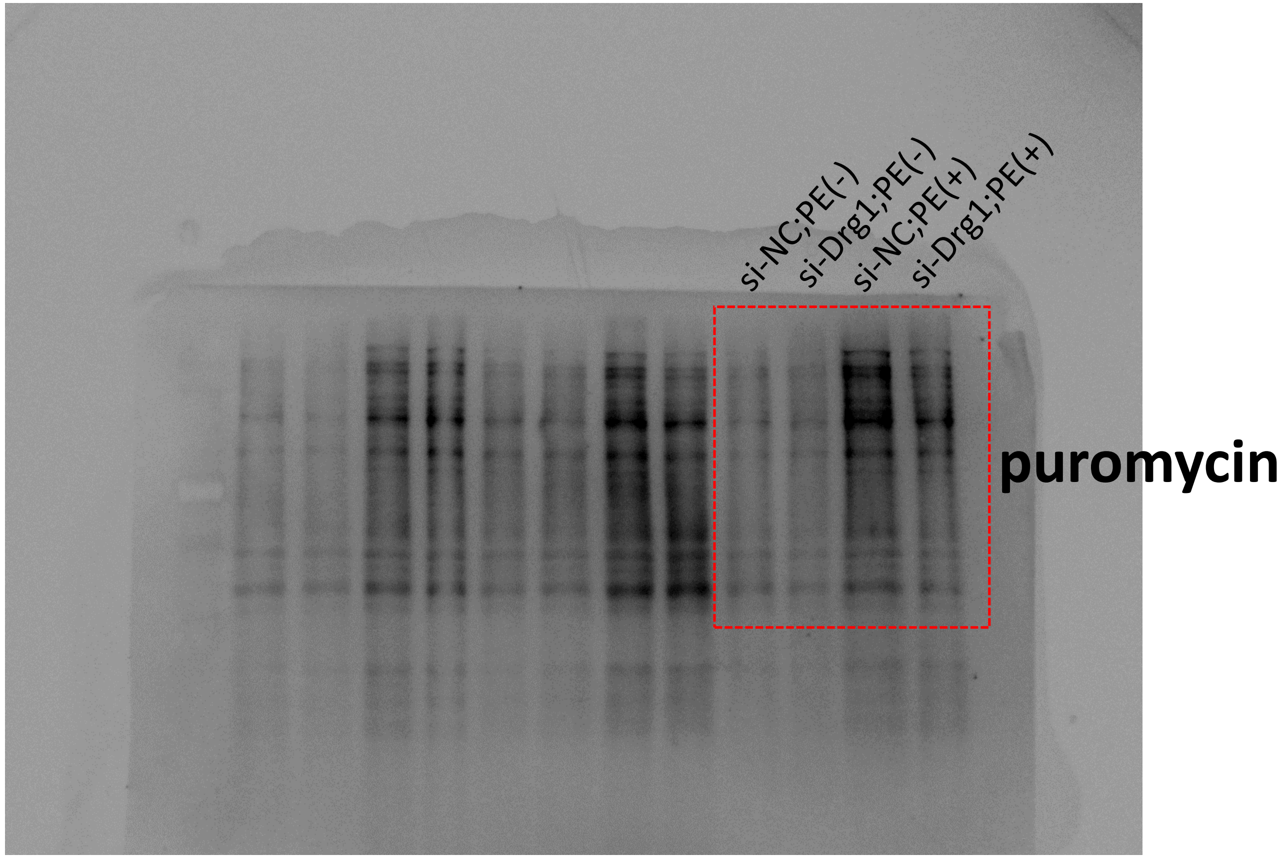
si-NC;PE(-)
si-Drg1;PE(-)
si-NC;PE(+)
si-Drg1;PE(+)
puromycin

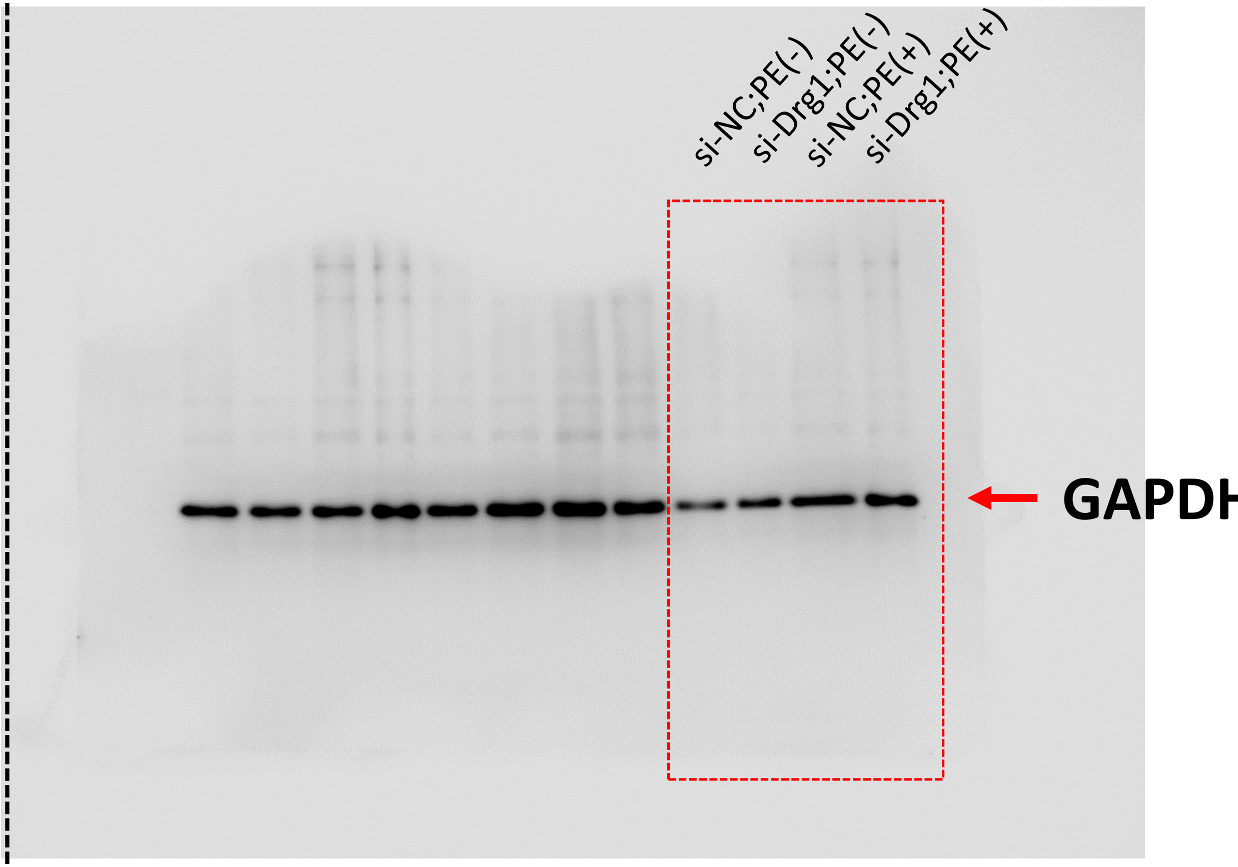
si-NC;PE(-)
si-Drg1;PE(-)
si-NC;PE(+)
si-Drg1;PE(+)
GAPDH

Figure 7A

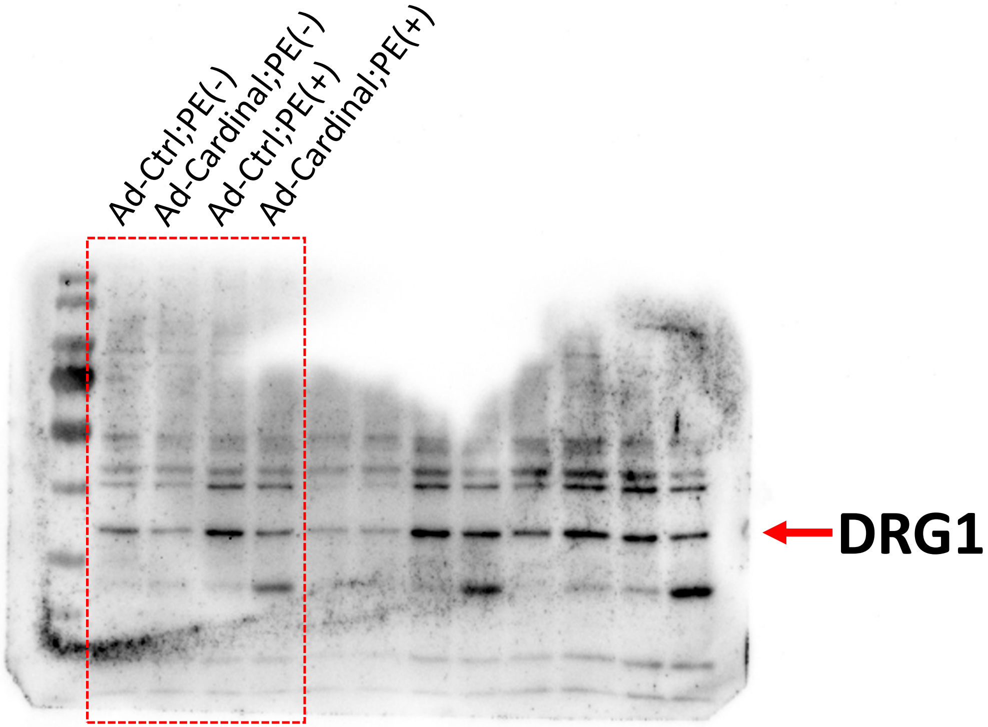

Ad-Ctrl;PE(-)
Ad-Cardinal;PE(-)
Ad-Ctrl;PE(+)
Ad-Cardinal;PE(+)
DRG1

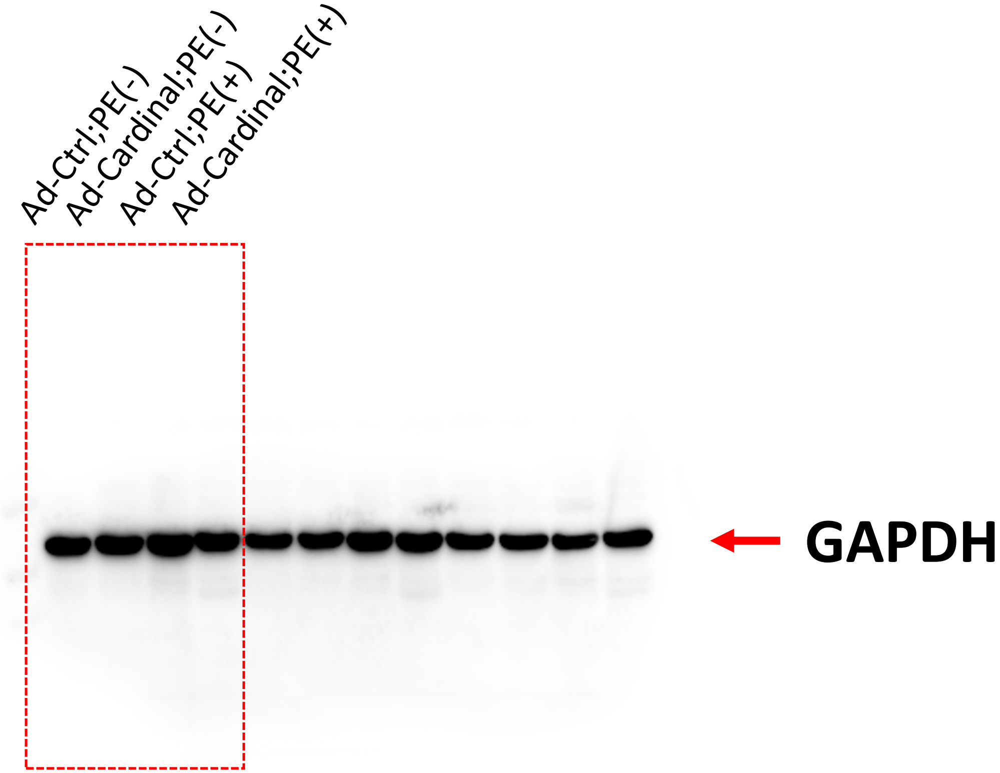

Ad-Ctrl;PE(-)
Ad-Cardinal;PE(-)
Ad-Ctrl;PE(+)
Ad-Cardinal;PE(+)
GAPDH

Figure 7D

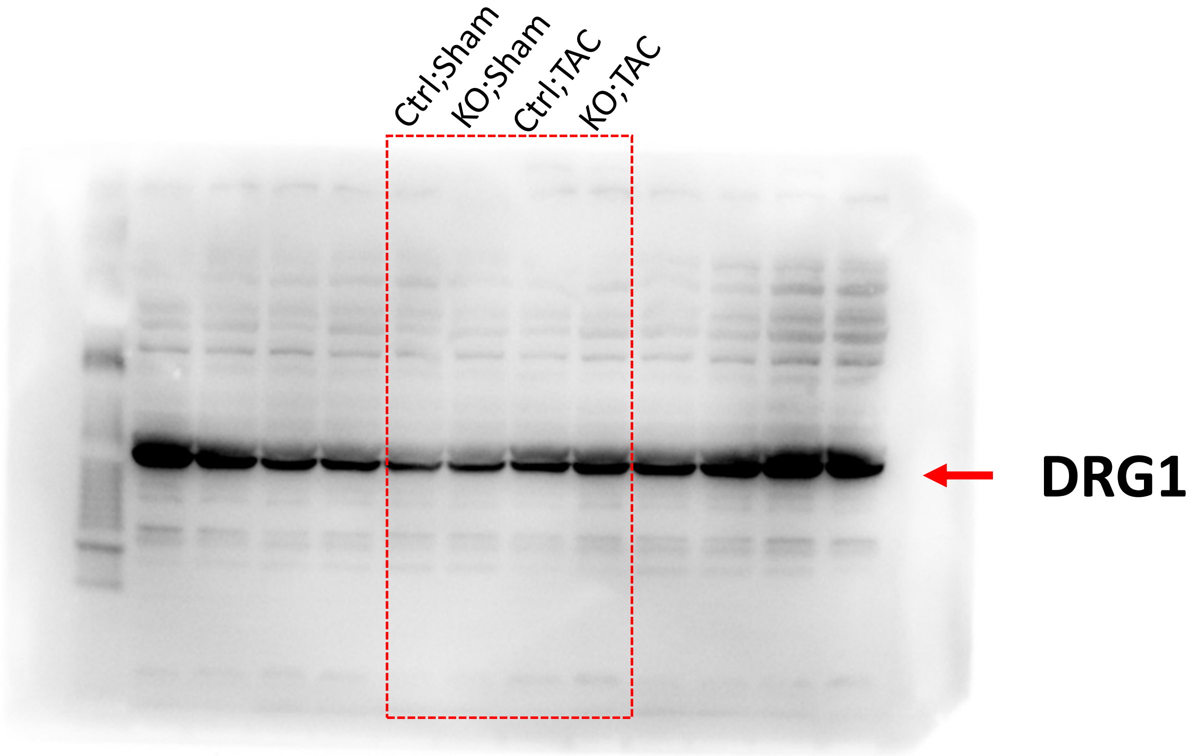

Ctrl;Sham
KO;Sham
Ctrl;TAC
KO;TAC
DRG1

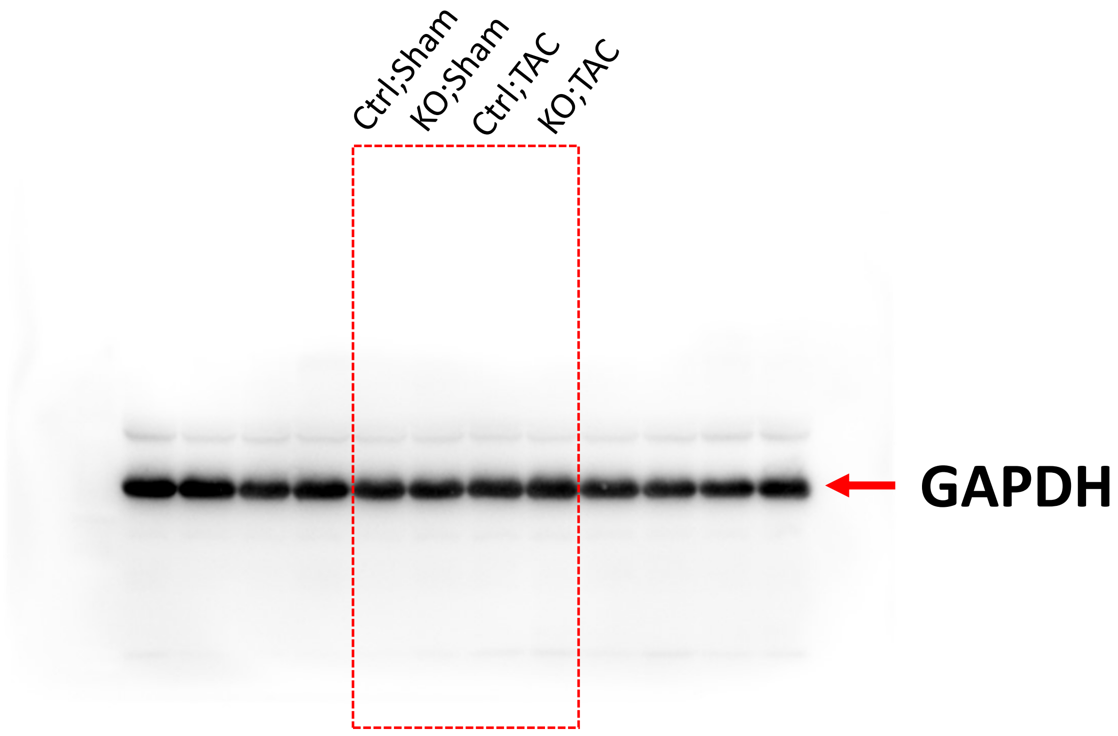

Ctrl;Sham
KO;Sham
Ctrl;TAC
KO;TAC
GAPDH

Figure 7G

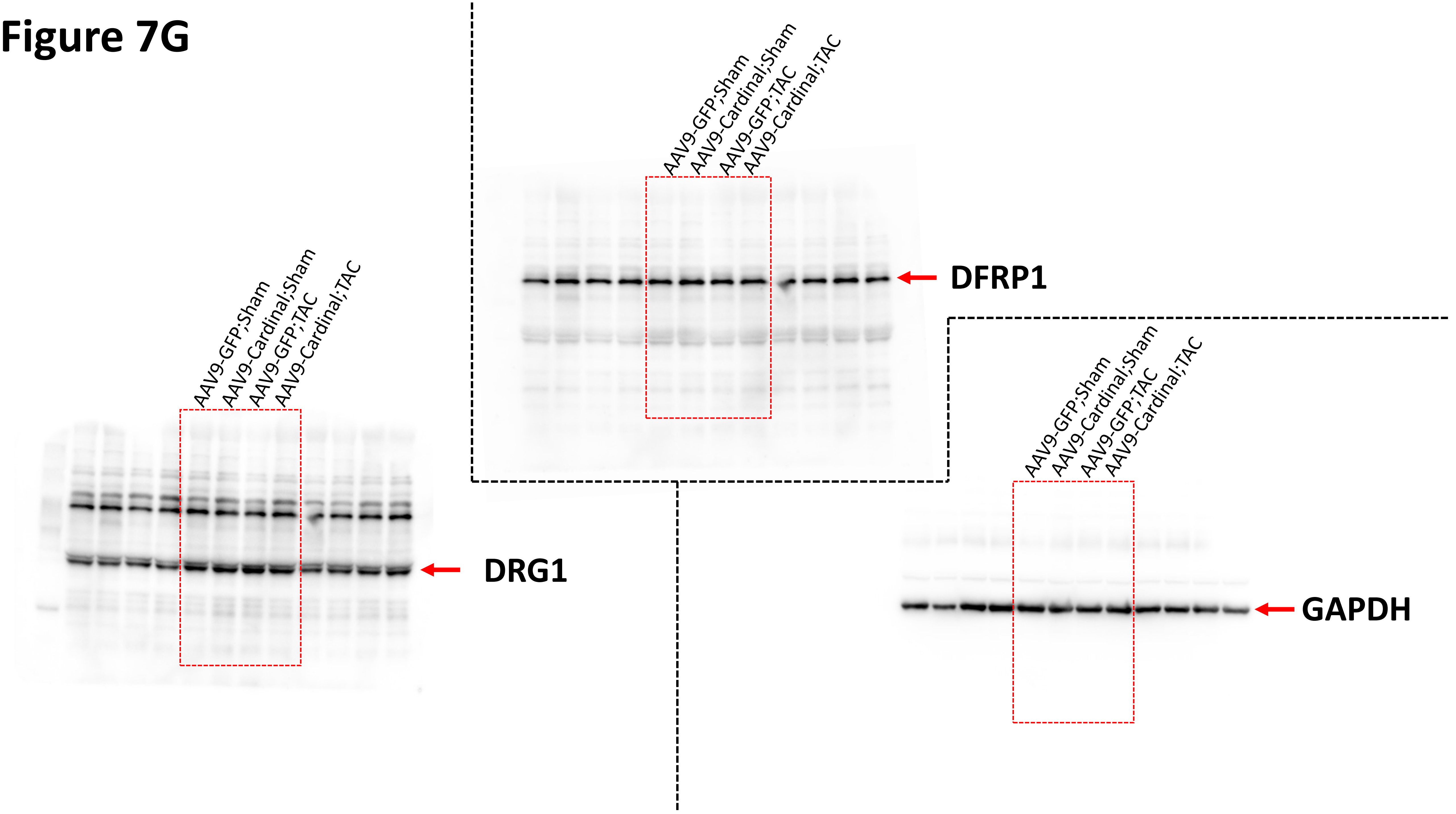
AAV9-GFP;Sham
AAV9-Cardinal;Sham
AAV9-GFP;TAC
AAV9-Cardinal;TAC
DFRP1
AAV9-GFP;Sham
AAV9-Cardinal;Sham
AAV9-GFP;TAC
AAV9-Cardinal;TAC
DRG1
AAV9-GFP;Sham
AAV9-Cardinal;Sham
AAV9-GFP;TAC
AAV9-Cardinal;TAC
GAPDH

Figure 7I

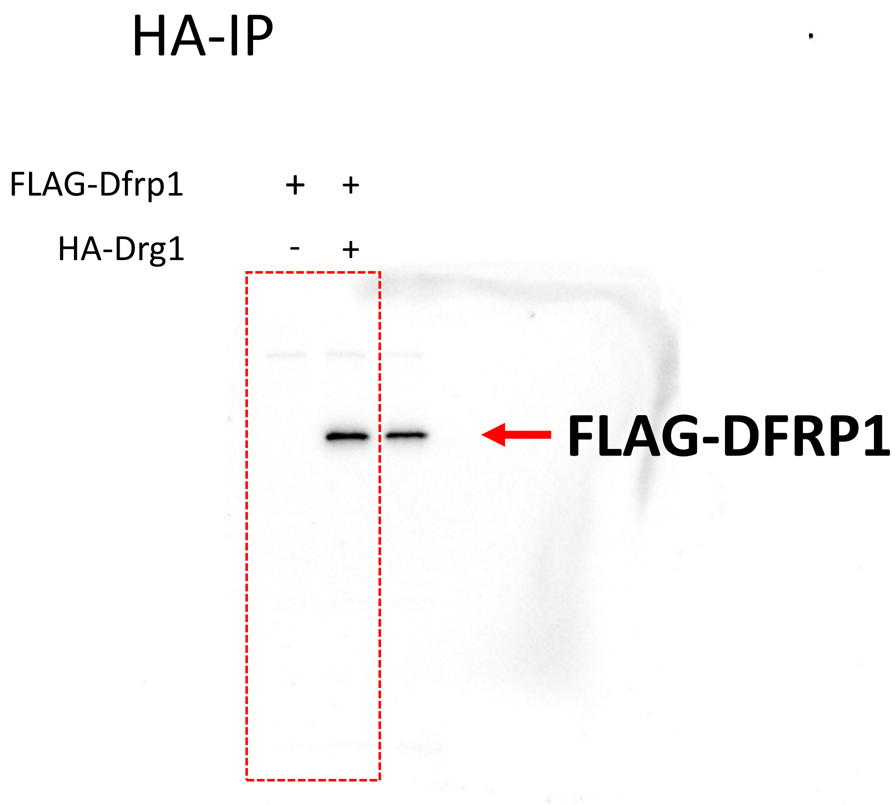
HA-IP
FLAG-Dfrp1 + +
HA-Drg1 - +
FLAG-DFRP1

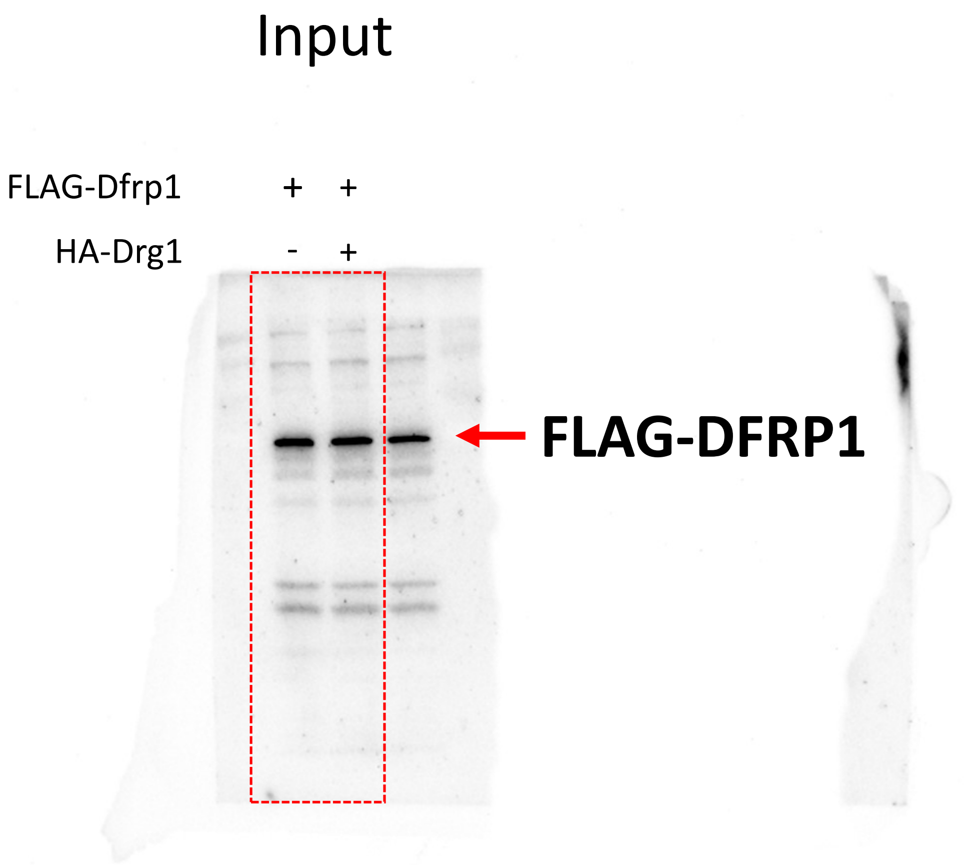
Input
FLAG-Dfrp1 + +
HA-Drg1 - +
FLAG-DFRP1

Figure 7J

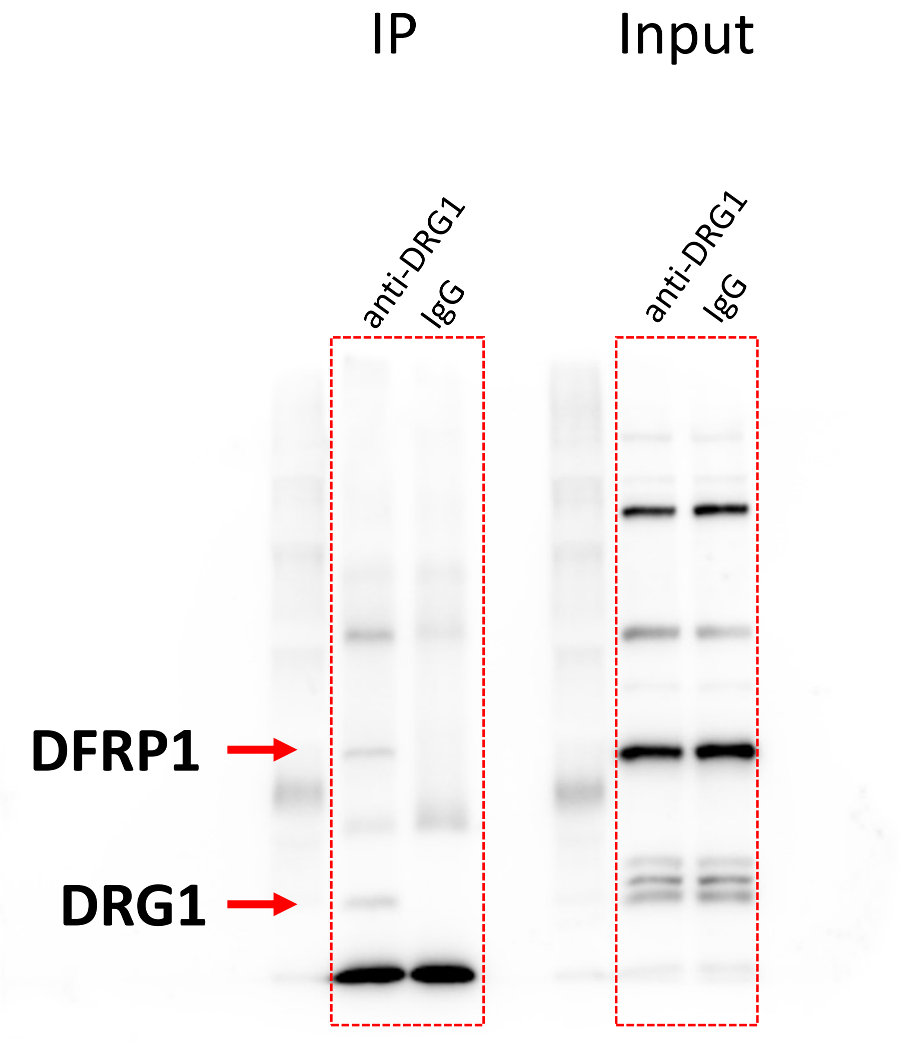

Figure 7K

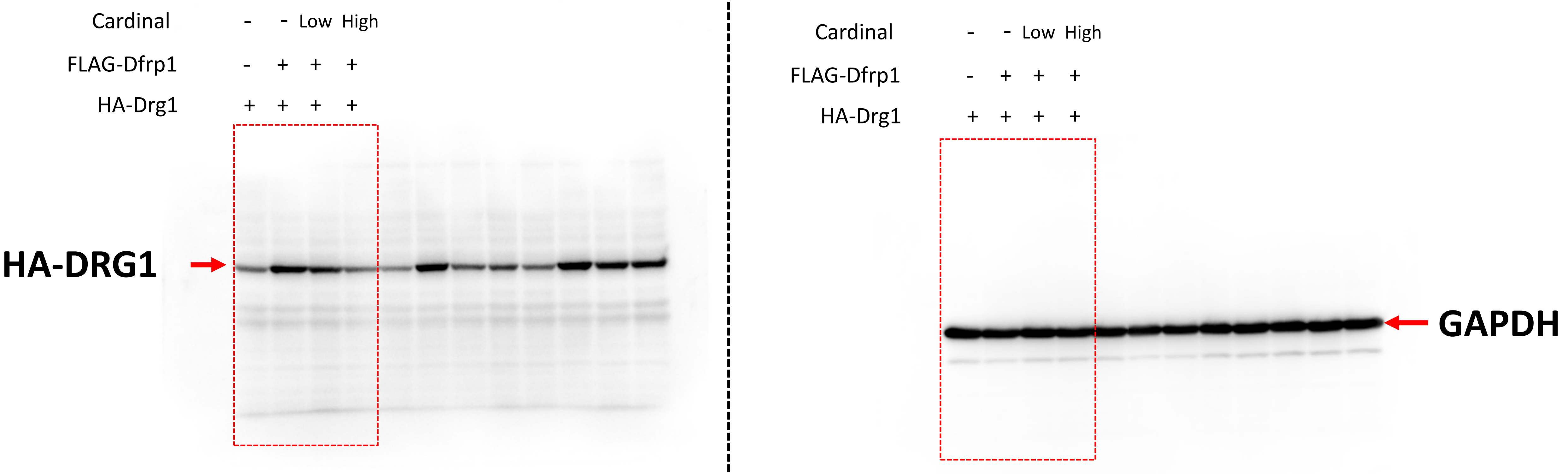
Cardinal - - Low High
FLAG-Dfrp1 - + + +
HA-Drg1 + + + +
HA-DRG1
Cardinal - - Low High
FLAG-Dfrp1 - + + +
HA-Drg1 + + + +
GAPDH

Figure 7L

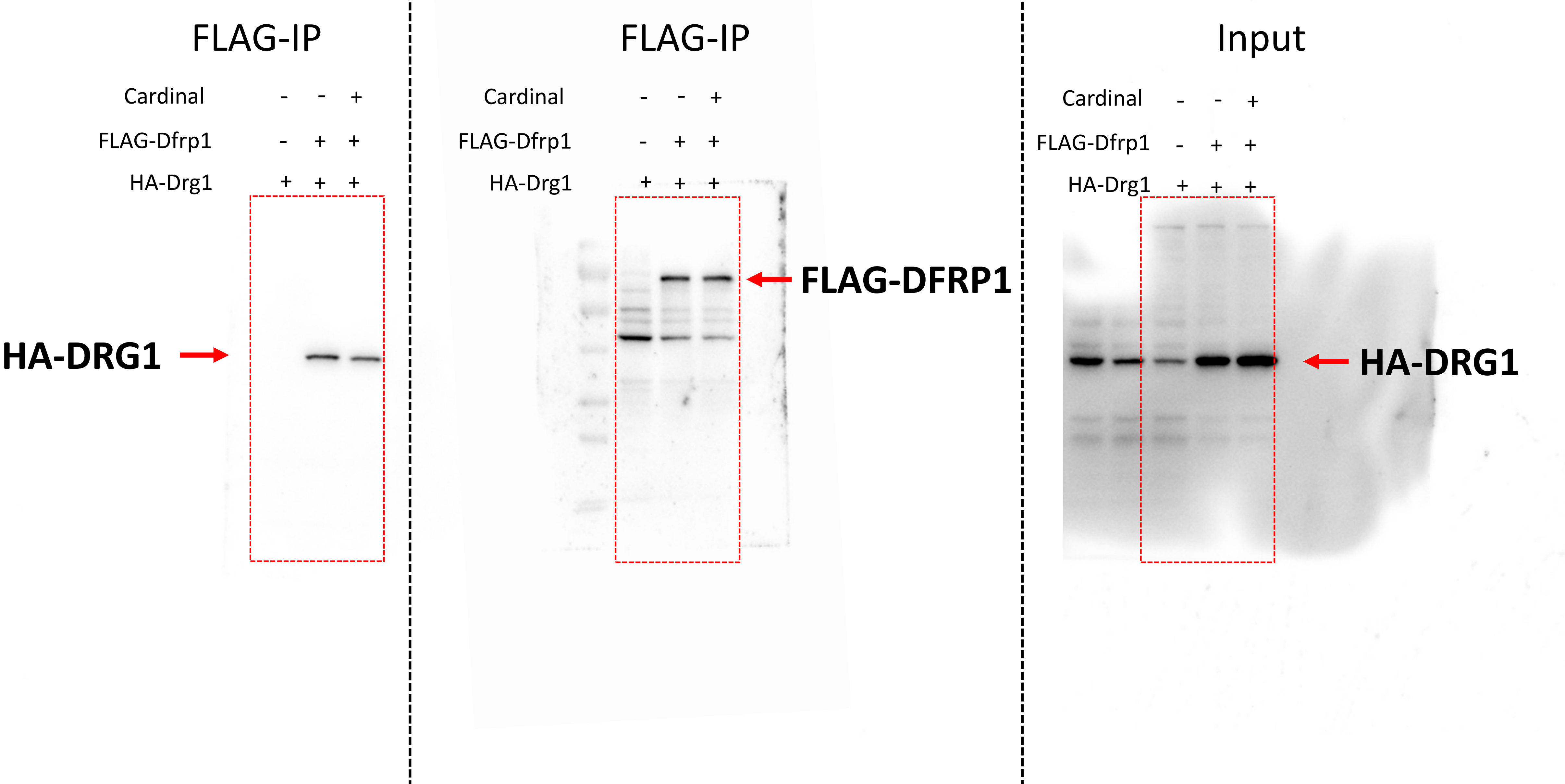
FLAG-IP
Cardinal - - +
FLAG-Dfrp1 - + +
HA-Drg1 + + +
HA-DRG1
FLAG-IP
Cardinal - - +
FLAG-Dfrp1 - + +
HA-Drg1 + + +
FLAG-DFRP1
Input
Cardinal - - +
FLAG-Dfrp1 - + +
HA-Drg1 + + +
HA-DRG1

Figure 7N

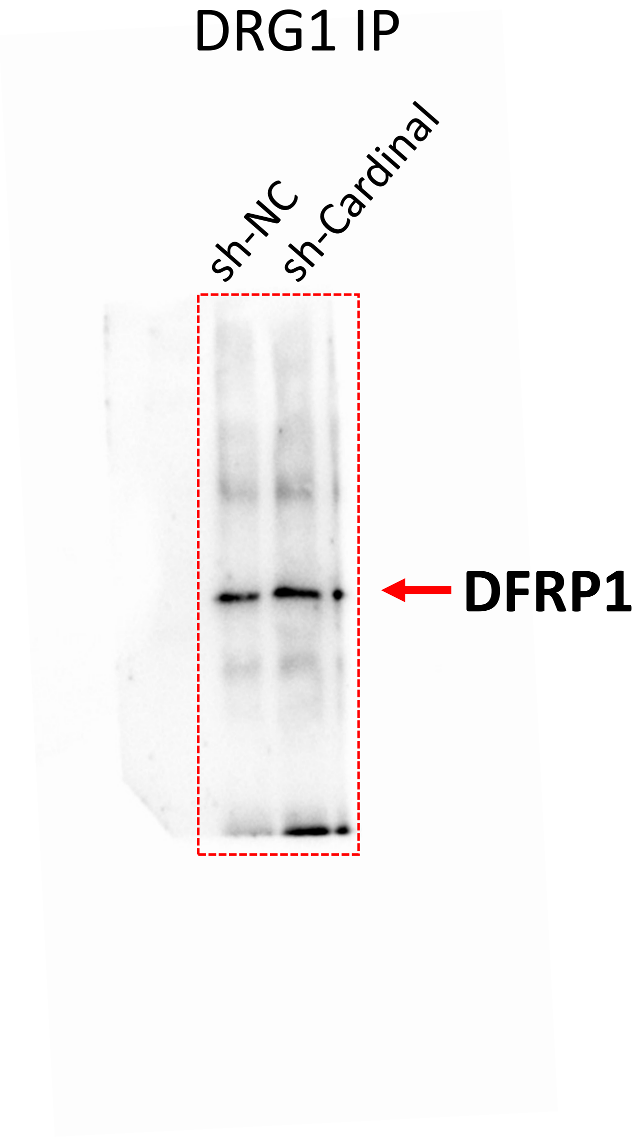

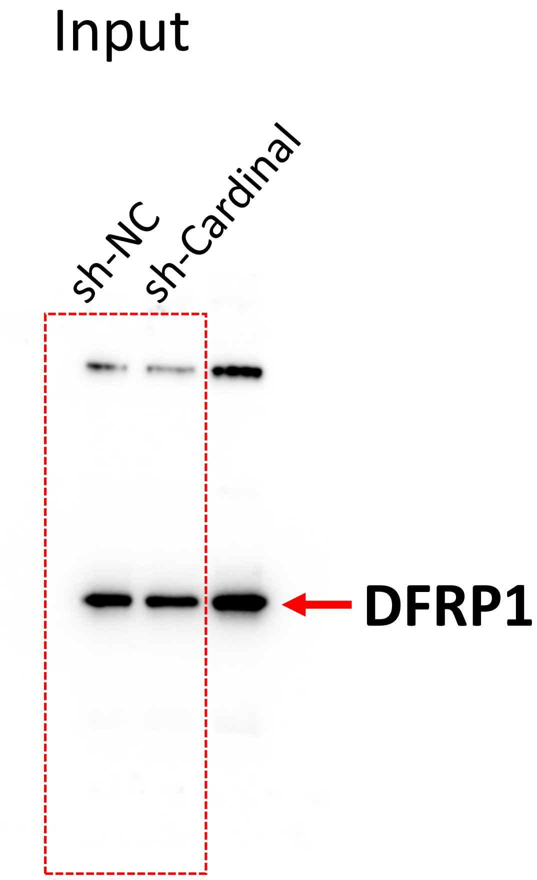

Figure 7J

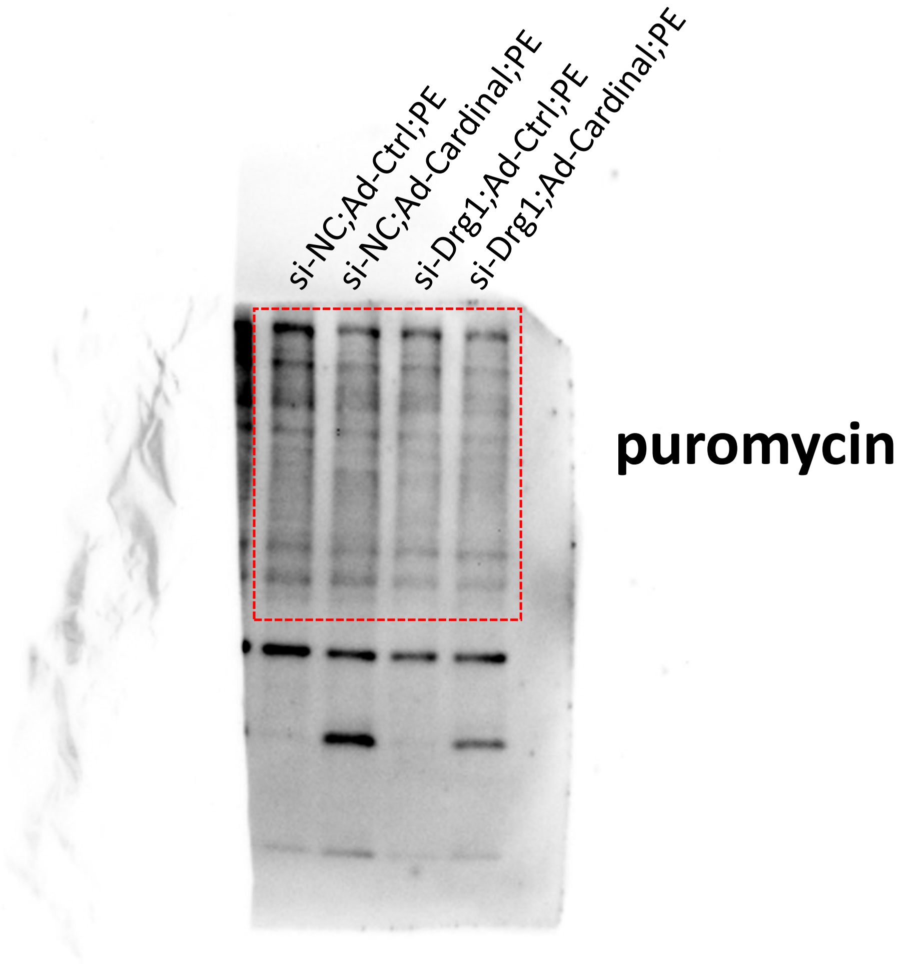
si-NC;Ad-Ctrl;PE
si-NC;Ad-Cardinal;PE
si-Drg1;Ad-Ctrl;PE
si-Drg1;Ad-Cardinal;PE
puromycin

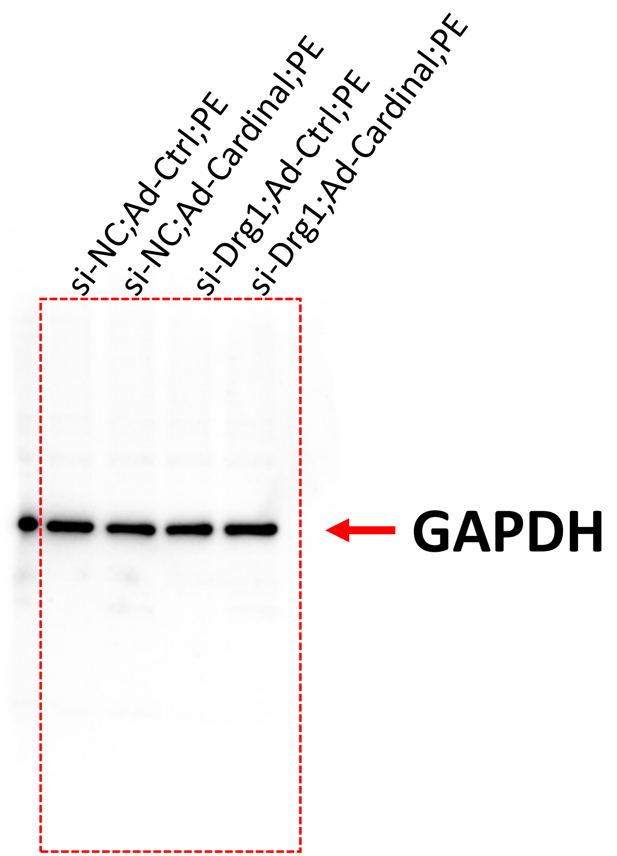
si-NC;Ad-Ctrl;PE
si-NC;Ad-Cardinal;PE
si-Drg1;Ad-Ctrl;PE
si-Drg1;Ad-Cardinal;PE
GAPDH

Supplementary Figure 11A

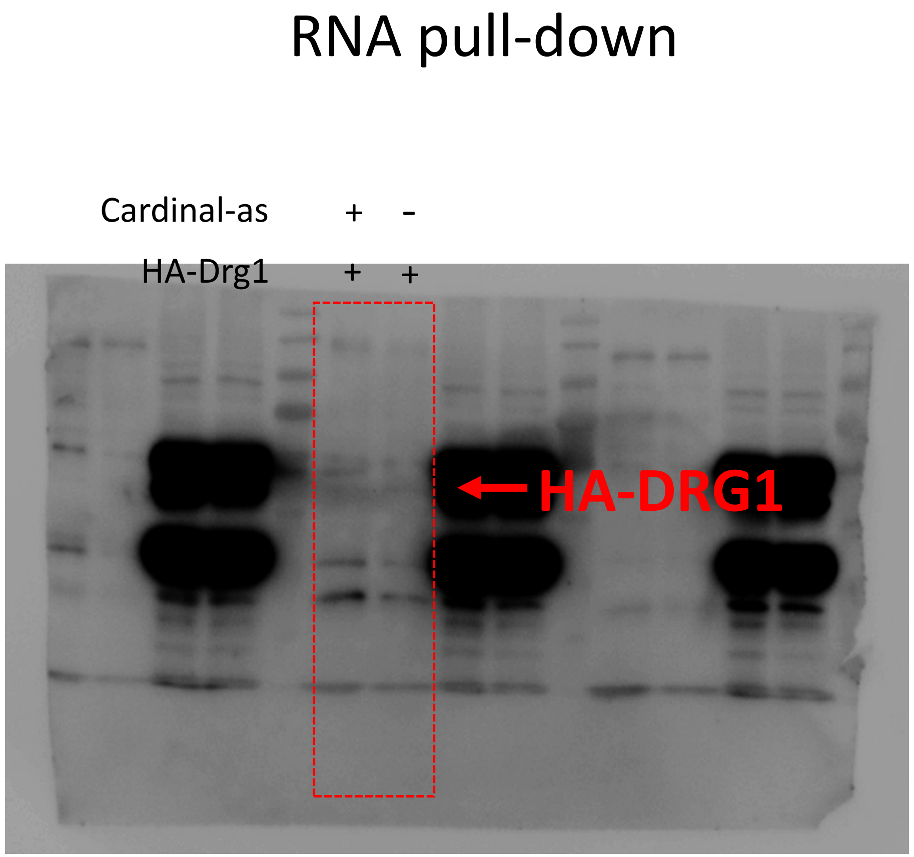

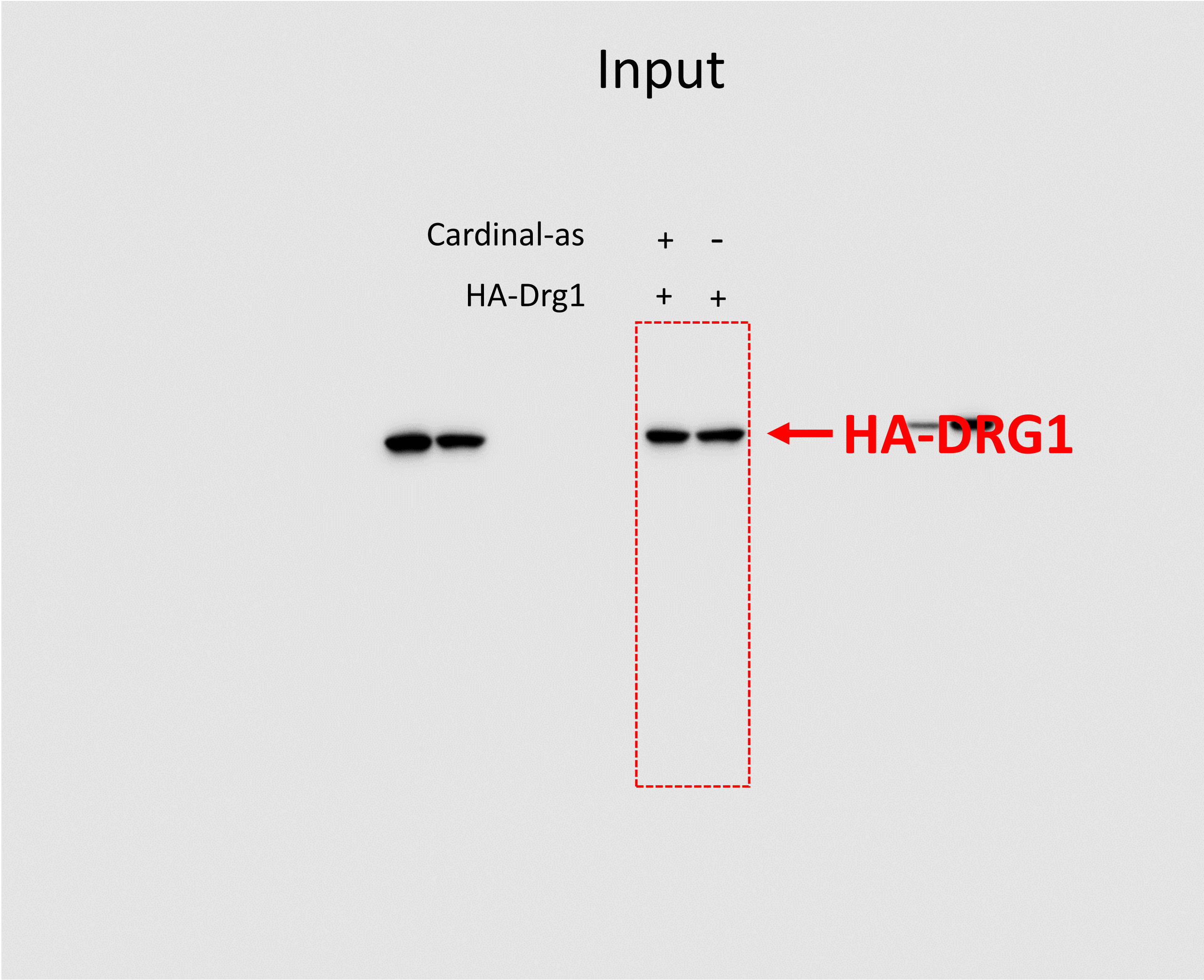

Supplementary Figure 11B

RNA pull-down

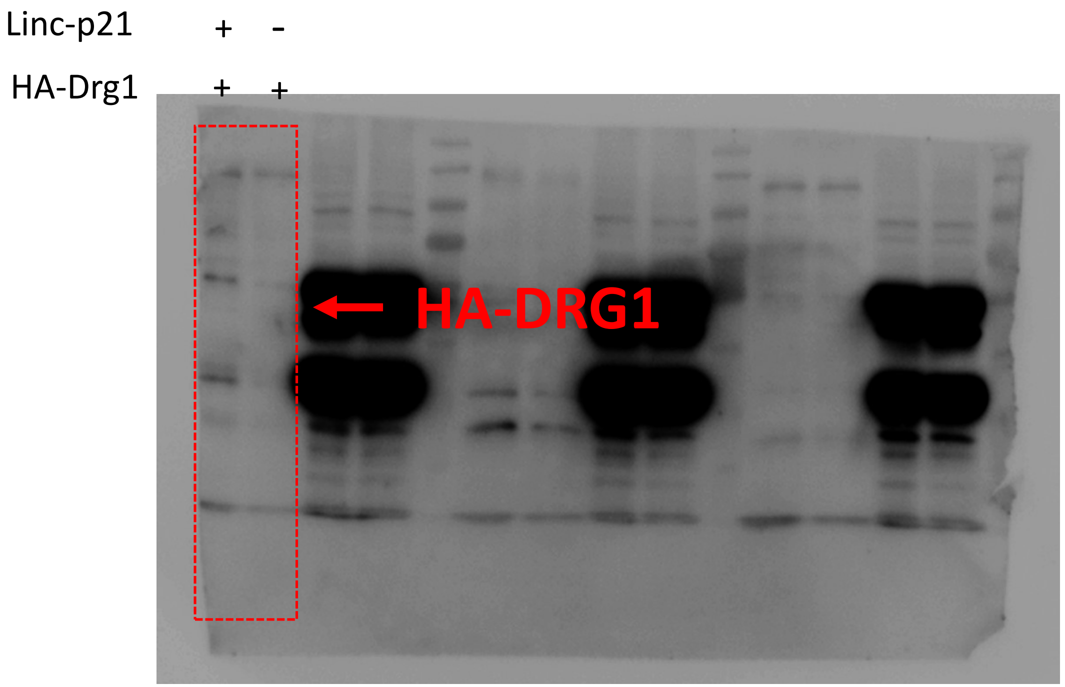

Input

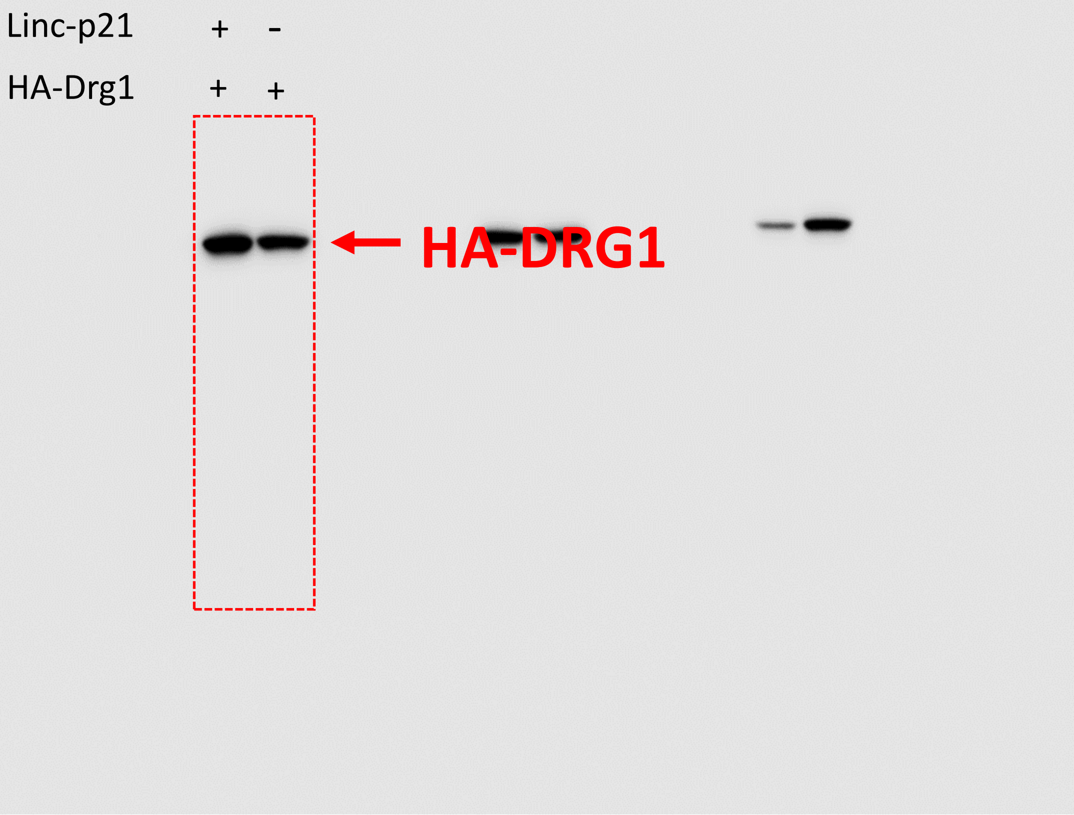

Supplementary Figure 11C

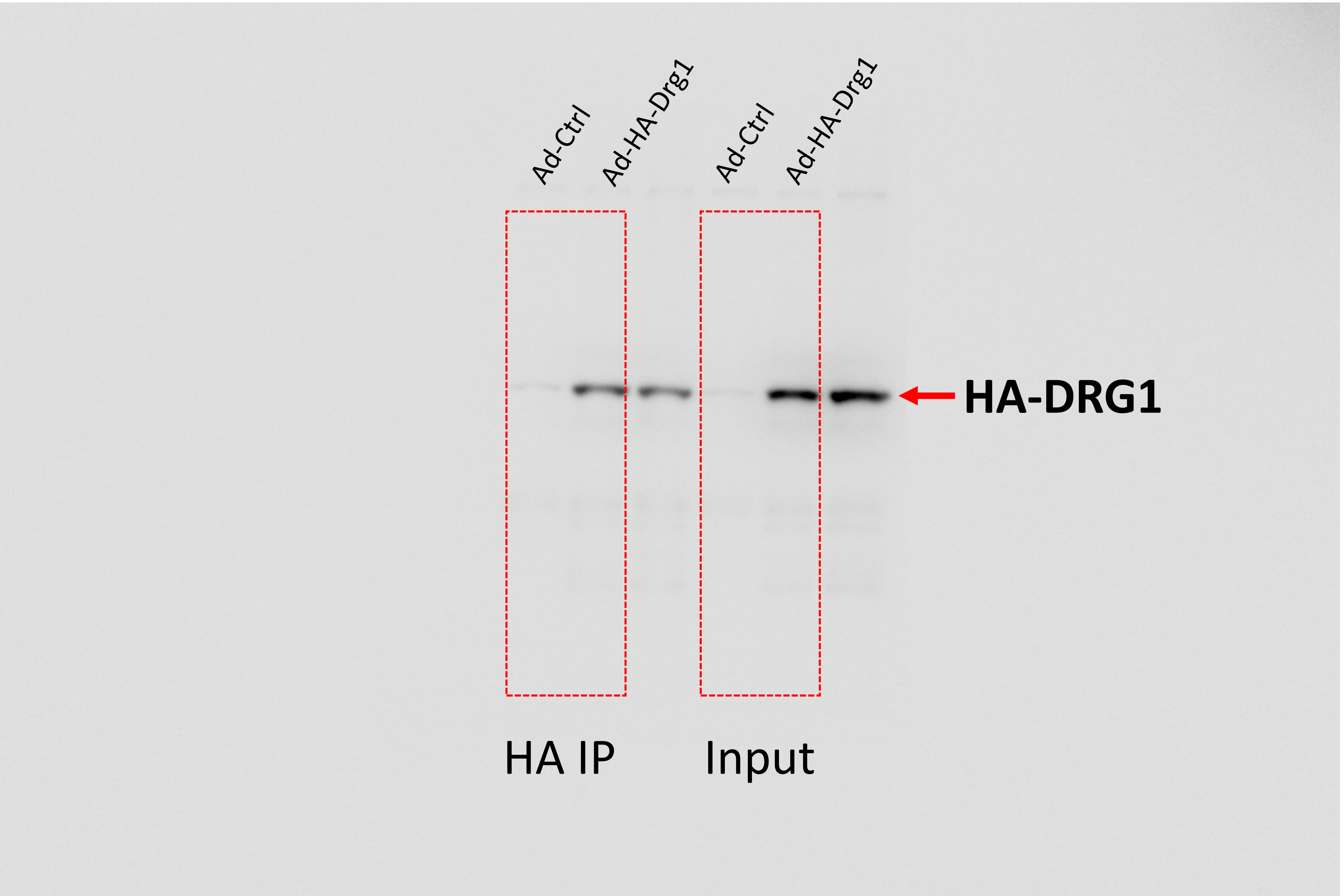

Supplementary Figure 12A

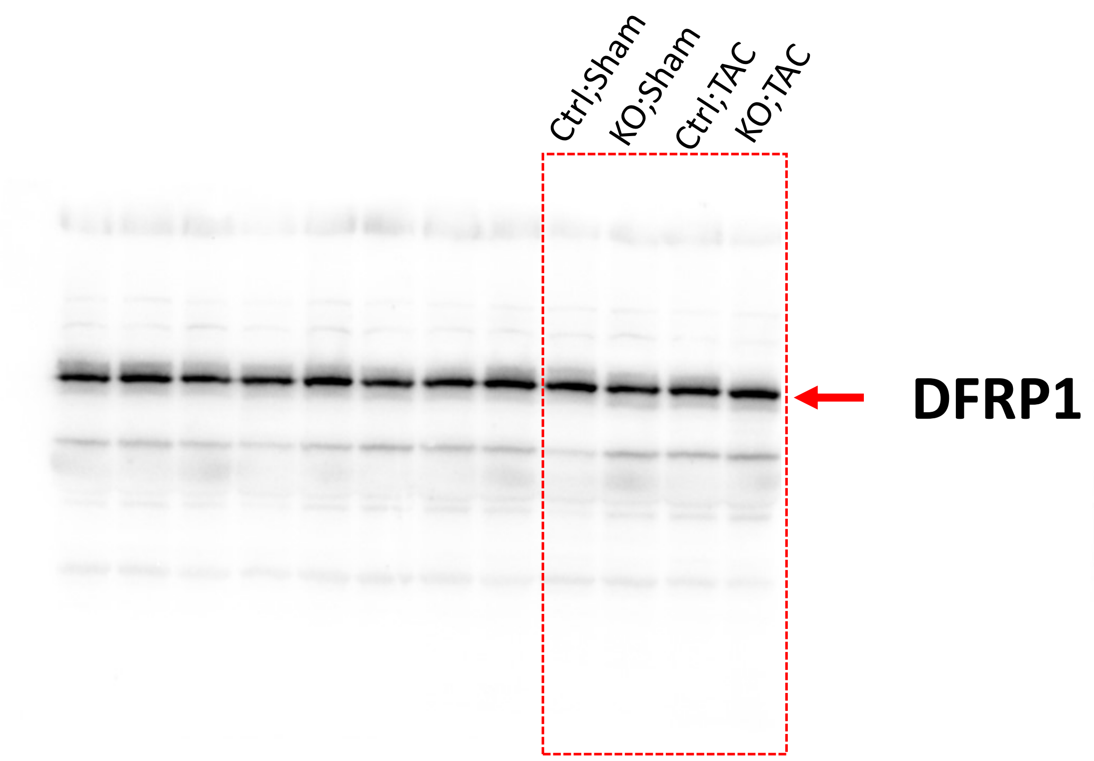

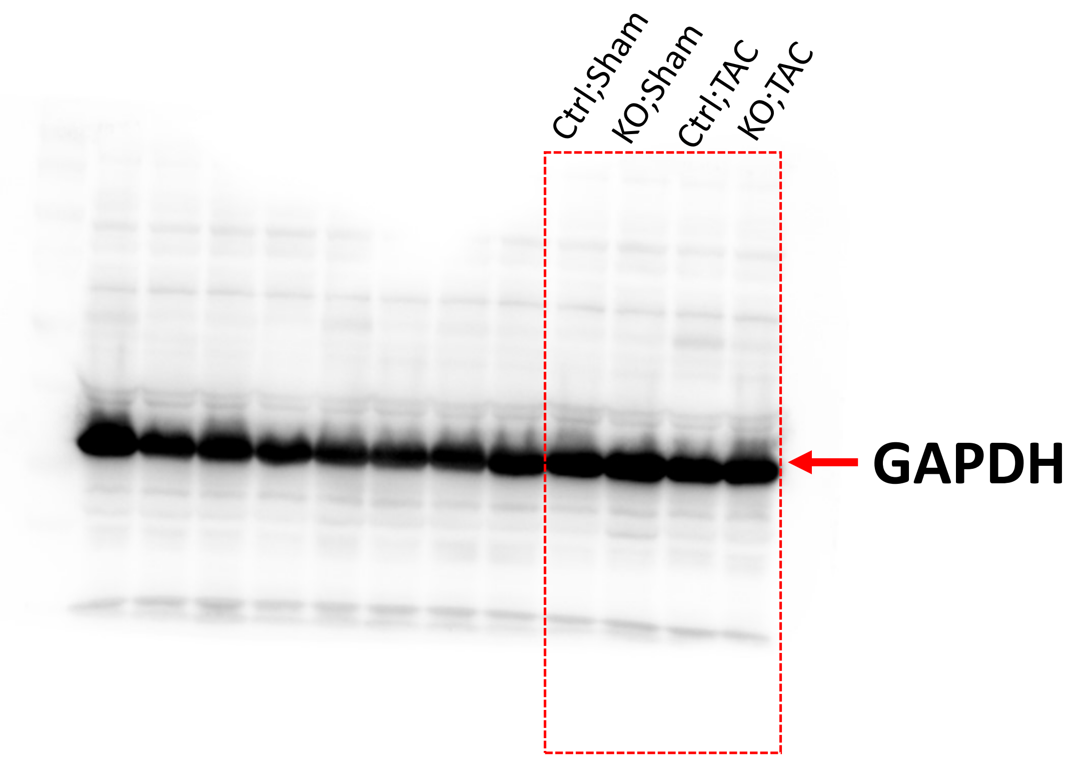

Supplementary Figure 12D

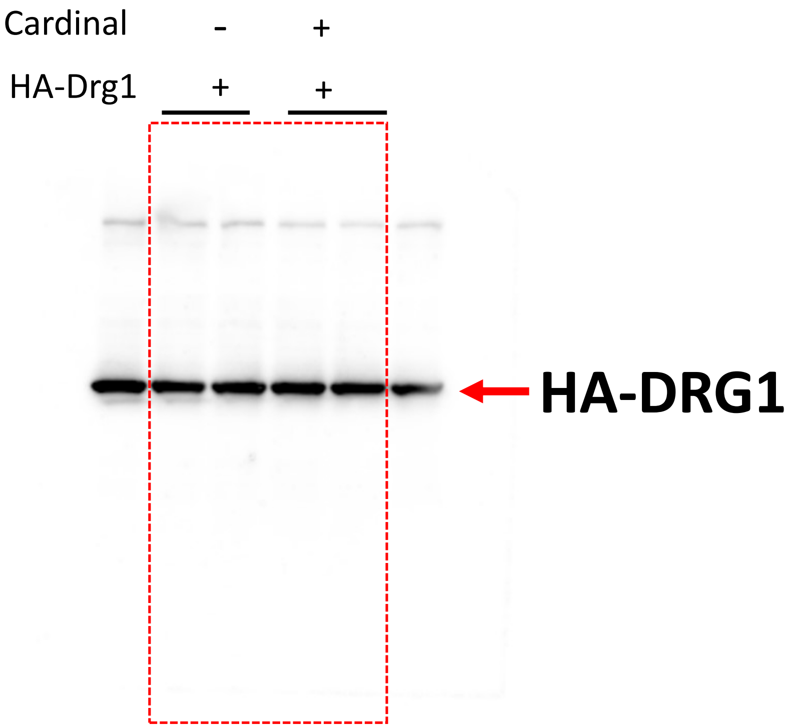

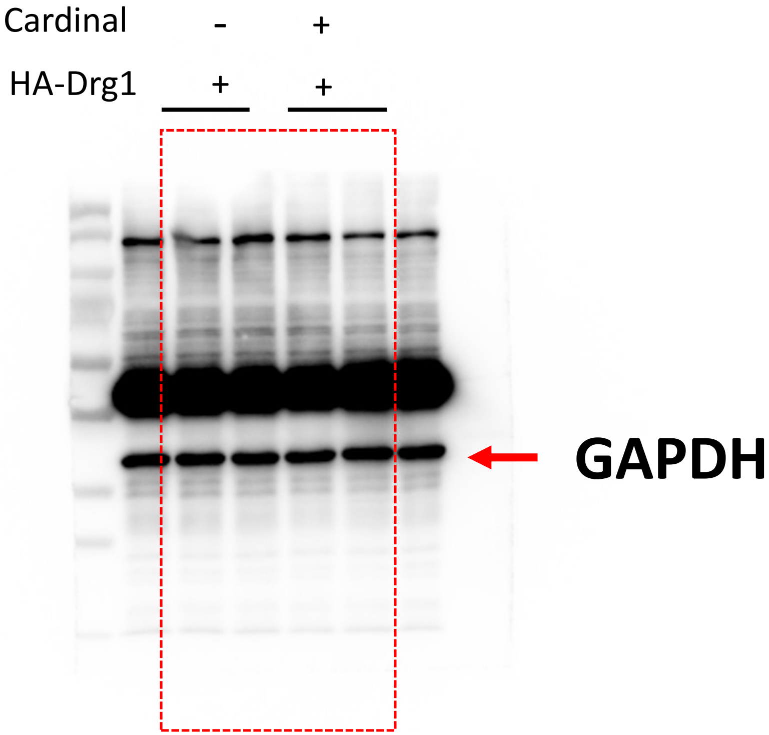

Supplementary Figure 12E

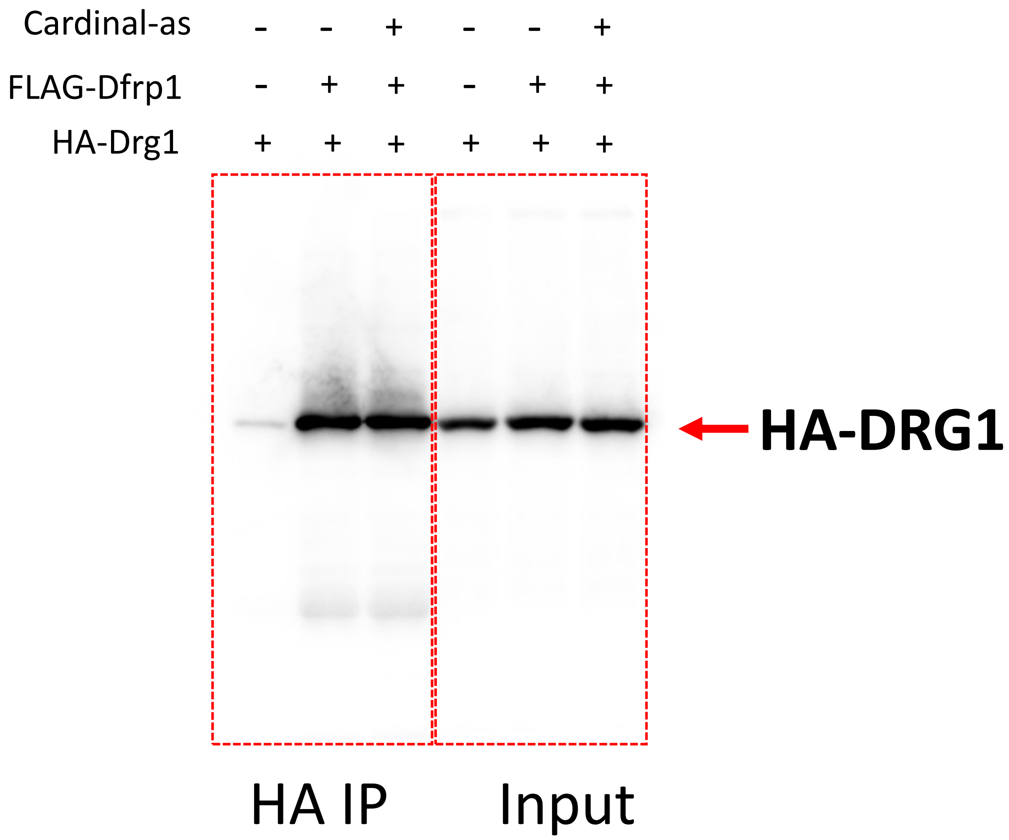

Supplement: Unedited blot and gel images [file jci-134-169112-s237.pdf]
